# Variation in responses to temperature across admixed genotypes of *Populus trichocarpa* × *P. balsamifera* predict geographic shifts in regions where hybrids are favored

**DOI:** 10.1101/2025.05.16.654548

**Published:** 2025-05-22

**Authors:** Alayna Mead, Joie R. Beasley-Bennett, Andrew Bleich, Dylan Fischer, Shelby Flint, Julie Golightly, Sara K. Klopf, Mason W. Kulbaba, Jesse R. Lasky, Jared M. LeBoldus, David B. Lowry, Nora Mitchell, Emily Moran, Jason P. Sexton, Kelsey L. Søndreli, Baxter Worthing, Michelle Zavala-Paez, Matthew C. Fitzpatrick, Jason Holliday, Stephen Keller, Jill A. Hamilton

**Affiliations:** 1 Pennsylvania State University; 2 Oregon State University; 3 Michigan State University; 4 The Evergreen State College; 5 Southwest Minnesota State University; 6 Salisbury University; 7 Virginia Tech; 8 St. Mary’s University; 9 University of Wisconsin - Eau Claire; 10 University of California Merced; 11 University of Vermont; 12 University of Maryland Center for Environmental Science; 13 Virginia Tech University

**Keywords:** climate change, hybrid zone, plasticity, *Populus*, provenance trial, climate response function

## Abstract

In a rapidly changing environment, predicting changes in the growth and survival of local populations can inform conservation and management. Plastic responses vary as a result of genetic differentiation within and among species, so accurate rangewide predictions require characterization of genotype-specific reaction norms across the continuum of historic and future climate conditions comprising a species’ range. Natural hybrid zones can give rise to novel recombinant genotypes associated with high phenotypic variability, further increasing the variance of plastic responses within the ranges of the hybridizing species. Experiments that plant replicated genotypes across a range of environments can characterize genotype-specific reaction norms; identify genetic, geographic, and climatic factors affecting variation in climate responses; and make predictions of climate responses across complex genetic and geographic landscapes. The North American hybrid zone of *Populus trichocarpa* and *P. balsamifera* represents a natural system in which reaction norms are likely to vary with underlying genetic variation that has been shaped by climate, geography, and introgression. Here, we leverage a dataset containing 45 clonal genotypes of varying ancestry from this natural hybrid zone, planted across 17 replicated common garden experiments spanning a broad climatic range, including sites warmer than the natural species ranges. Growth and mortality were measured over two years, enabling us to model reaction norms for each genotype across these tested environments. Genomic variation associated with species ancestry and northern/southern regions significantly influenced growth across environments, with genotypic variation in reaction norms reflecting a trade-off between cold tolerance and growth. Using modeled reaction norms for each genotype, we predicted that genotypes with more *P. trichocarpa* ancestry may gain an advantage under warmer climates. Spatial shifts of the hybrid zone could facilitate the spread of beneficial alleles into novel climates. These results highlight that genotypic variation in responses to temperature will have landscape-level effects.

## Introduction

Predicting the phenotypic responses of populations to changing climates and how they vary across species ranges is essential for conserving and restoring native ecosystems. Predicted changes in fitness between current and future climates can be used to identify populations most at risk, or to identify populations resilient to change which could be valuable seed sources for assisted gene flow and restoration (Aitken and Whitlock 2013; Aitken and Bemmels 2016). Phenotypes expressed in the field arise from the genotypic variation underlying phenotypic traits (G), the plastic response to the environment (E), and genotypic variation in response to the environment (GxE) (Des Marais et al. 2013). Each of these factors vary across complex and changing landscapes. By characterizing reaction norms, the set of phenotypes expressed across a range of environments, it is possible to predict organisms’ responses to the variable environments they inhabit and understand the selective forces that may be shaping plasticity (Via et al. 1995; Arnold et al. 2019). Reaction norms can vary within species, resulting in part from selection imposed by spatially varying climates (Des Marais et al. 2013; Ikeda et al. 2017; Rehfeldt et al. 2018; Patsiou et al. 2020). Predicting genotype-specific responses will be particularly important for species with large ranges, which may exhibit variation in reaction norms due to the combined influence of geography, population demography, and climate (Cooper et al. 2019, 2022; Van Nuland et al. 2020). This is particularly true where glacial refugia have shaped species’ demographic history and connectivity (Woolbright et al. 2014; Love et al. 2023; Bolte et al. 2024). However, because of the difficulty of phenotyping multiple genotypes across many environments, many methods for modeling a species’ response to climate change ignore genotypic variation, and instead consider only the climate envelope of the extant species range (Capblancq et al. 2020). Furthermore, regions within a species’ range will vary in the rate, magnitude, and nature of climate change (e.g, increases or decreases in precipitation), making it important to understand localized climate responses. To accurately predict local phenotypic responses to changing climates, it is necessary to characterize genotype-specific reaction norms across the continuum of historic and future climate conditions comprising a species’ range.

Common garden experiments and provenance trials are invaluable tools for quantifying intraspecific variation in phenotypic plasticity and for predicting phenotypic responses to current and future environments (O’Neill et al. 2008; Wang et al. 2010; Leites et al. 2012; Fischer et al. 2014a; Grady et al. 2015; Browne et al. 2019; Leites and Benito Garzón 2023; Ye et al. 2023; Hord et al. 2025). Capturing variation in reaction norms varies across genetic and climatic gradients is critical and can be accomplished by repeated planting of genotypes across multiple environments. Genotype-specific reaction norms modeled across continuous environments can be used to identify genotypes or loci associated with optimized fitness or yield across varying environmental conditions; however, such studies have been limited to a handful of species (Gray et al. 2011; Arnold et al. 2019; Lowry et al. 2019; Patsiou et al. 2020; VanWallendael et al. 2022; Li et al. 2024). Because plasticity results from adaptation to climate as well as demographic history, it may be possible to predict the reaction norms of unmeasured genotypes if they vary as a function of climate of origin (O’Neill et al. 2008; Wang et al. 2010; Rehfeldt et al. 2018) or genetic variation. When genomic data are available in addition to phenotypic and climate data collected in common garden experiments, predictions of phenotypic responses can be improved (Mahony et al. 2020; Archambeau et al. 2022; Putra et al. 2023; Li et al. 2024). Therefore, incorporating genetic information into characterization of reaction norms will enable more accurate predictions of rangewide climate responses.

Natural hybrid zones are ideal systems for disentangling the effects of adaptation and demographic history on phenotypes, including reaction norms, because multiple generations of backcrossing can result in novel recombinant genotypes associated with high phenotypic variability (Janes and Hamilton 2017). Furthermore, hybrid zones can increase the genetic variation available for adaptation to rapidly changing climates and allow movement of adaptive loci from one species into another via introgression (Janes and Hamilton 2017; Suarez-Gonzalez et al. 2018b; Kremer and Hipp 2020; Buck et al. 2023; Hord et al. 2025). Comparing responses to the environment across admixed genotypes can inform how existing genetic variation within hybrid zones underlies fitness differences across environments. Hybrid zones can also be used to monitor responses to climate change (Taylor et al. 2015), with documented geographic shifts in some zones in response to changing environments (Billerman et al. 2016; Wielstra 2019; Alexander et al. 2022). If reaction norms depart from the intraspecific pattern across a hybrid zone, climate change may alter regions where particular species, genotypes, or even genes are favored (Hord et al. 2025).

**Figure 1.**
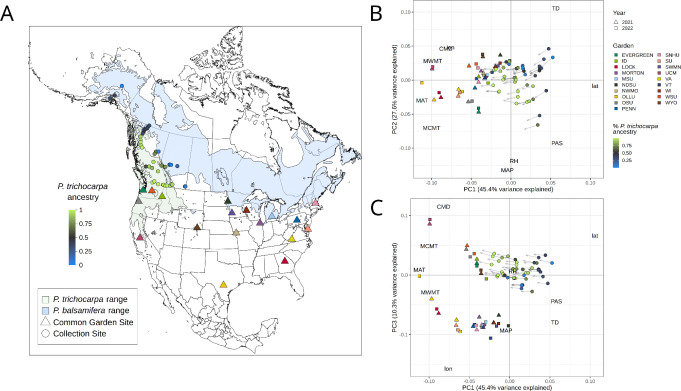
(A) Map of sampled genotype localities (circles) and common garden sites (triangles) in relation to the ranges of both species (Little 1971); color of sampled genotypes indicates species ancestry calculated from ADMIXTURE at K=2. (B-C) PCAs of past and future climates across collection sites and garden sites. Circles represent the historic climate (1961–1990) of each genotype’s provenance, colored by species ancestry, and arrowheads represent the predicted climate for 2041–2070 under an ensemble of 13 GCMs. Triangles and squares represent the climate at common garden sites for 2021 and 2021. Text indicates the loadings of climate and geographic variables on each axis, with abbreviations as follows: CMD, climatic moisture deficit; lat, latitude; lon, longitude; MAP, mean annual precipitation, MAT, mean annual temperature; MCMT, mean coldest month temperature; MWMT, mean warmest month temperature, PAS, precipitation as snow; RH, relative humidity; TD, temperature difference, or continentality. Loadings of climate variables have been downscaled to allow visibility of sites.

North American *Populus* is a model system for forest trees due to their small genomes, ease of clonal propagation, and development as a biofuel feedstock (Jansson and Douglas 2007; Sannigrahi et al. 2010; Porth and El-Kassaby 2015), yet like many non-model trees, its genetic variation is shaped by interactions between climate, geography, and interspecific introgression. *Populus* species occupy heterogeneous landscapes, often forming multi-species hybrid zones that are a source of novel recombinant genetic variation (Suarez-Gonzalez et al. 2016, 2018a; Chhatre et al. 2018; Bolte et al. 2024). These factors make *Populus* an ideal system for quantifying intraspecific variation in climate responses and predicting landscape-scale changes in fitness. Here, we focus on the North American hybrid zone between *P. trichocarpa*, a western species spanning latitudes from Alaska to California, and *P. balsamifera*, which occurs transcontinentally throughout the boreal regions of the contiguous United States, Canada and Alaska (Figure 1A). Populations within the hybrid zone exhibit genetic and phenotypic differentiation, likely resulting from varying demographic history as well as adaptation to wide-ranging biotic and abiotic environments (Keller et al. 2010, 2011, 2012; Slavov et al. 2012; Evans et al. 2014; Geraldes et al. 2014; McKown et al. 2014a, c,b; Zhou et al. 2014; Fitzpatrick and Keller 2015; Holliday et al. 2016; Chhetri et al. 2019; Zhang et al. 2019; Fitzpatrick et al. 2021). Furthermore, rates of gene flow across the hybrid zone vary in part due to geographic barriers, resulting in spatial differences in population differentiation (Bolte et al. 2024).

We leveraged the *P. trichocarpa* × *P. balsamifera* hybrid zone to determine how genetic and environmental parameters interact to influence genotype-specific reaction norms, ultimately using our predictions to model changes to hybrid zone composition in the context of global change. We used a series of replicated provenance trials of clonal genotypes of *P. trichocarpa*, *P. balsamifera*, and their hybrids, which we planted in 17 gardens across the United States that span a wide range of environments, including many warmer than the climate of origin. We tested three hypotheses: 1) *Populus* hybrids display heritable differences in adaptation to climate, which manifest as genotype-specific responses to climatic variation. We measured fitness-related traits in each garden to determine how genetic background and the environment interact to determine fitness in this system, quantifying the effect of genetic structure (genotype effect, G), climate (environmental effect, E) and how the response to climate varies by genotype (G×E). A significant genotype × environment interaction could suggest that the response to climate is mediated by local adaptation to the climate of origin. 2) Variation in reaction norms are predictable based on a continuous gradient of genomic ancestry, enabling us to predict where the hybrid zone is favored to move under future climates. We used whole-genome sequence data to characterize multivariate genetic structure, including both species ancestry and intraspecific variation, and predicted its effect on phenotypic responses among genotypes. Finally, we used genotype-specific reaction norms to predict phenotypic responses to climate change across the landscape of the *P. trichocarpa* x *P. balsamifera* hybrid zone. 3) Inclusion of genetic information in phenotypic response models improves their predictions due to inherent genetic structure contributing to variation in reaction norms. We tested whether climate of origin can serve as a proxy for adaptive genetic structure, circumventing the need for genetic data. Using this experiment, we show that increased temperatures could shift where genotypes with different levels of admixture are favored, illustrating the importance of combining genomic data with common garden experiments to predict rangewide responses to temperature.

## Methods

### Field collections and propagation

In 2020 we established 17 common gardens containing clonal replicates of 48 poplar genotypes, including *P. trichocarpa*, *P. balsamifera*, and admixed individuals. Genotypes were established from dormant branches collected between October 2019 and March 2020 from natural populations spanning five transects that traverse the natural hybrid zone of *P. trichocarpa* and *P. balsamifera* and span latitudes from Alaska to southern British Columbia and Alberta (Figure 1). Dormant vegetative cuttings were transported to Virginia Tech (Critz, VA, USA) for propagation, as described in Bolte et al. (2024). Briefly, cuttings were exposed to a 30-second ZeroTol 2.0 fungal dip treatment, dipped in Garden Safe Take Root hormone (0.1% indole-3-butyric acid), rooted into a standard rooting mix, and placed on a mist bench for 38 days following which clonal genotypes were shipped and planted into common garden sites.

### Common garden design

Seventeen common gardens were established at colleges, universities, and arboreta across the United States in fall 2020, including: The Evergreen State College, WA; University of Idaho; Lockerly Arboretum, GA; Morton Arboretum, IL; Michigan State University; North Dakota State University; Northwest Missouri State Arboretum; Oregon State University; Our Lady of the Lake University, TX; Pennsylvania State University; Salisbury University, MD; Southwest Minnesota State University; University of California, Merced; Virginia Tech University; University of Wisconsin - Eau Claire; Washington State University; and University of Wyoming (Figure 1A). Garden sites span a range of environments and include the southern range of both *P. trichocarpa* and *P. balsamifera*, as well as warmer regions to the south of their native ranges, enabling us to predict responses to novel climates (Figure 1B). Each garden included two blocks, with each genotype replicated in each block. In total, the design included 48 genotypes × 17 gardens × 2 blocks × 1 genotype/block. Each garden had a minimum of 94 trees, for a total of 1656 individuals included across all gardens. Phenotypic data were collected during the growing season for three years: 2021–2023. All 17 gardens were evaluated in the first year, but high mortality and changing local phenotyping capacity at some sites impacted the total number of gardens assessed, with fifteen and twelve sites evaluated in 2022 and 2023, respectively.

As part of the same experiment, three additional common gardens were established in March 2020. These gardens included 544 genotypes, each with three replicated individuals planted in three blocks (1 replicate per block), located at North Dakota State University (NDSU), Virginia Tech University (VA), and the University of Vermont (VT). We designated the 17 smaller gardens as “mini” gardens, and the three larger gardens as “maxi” gardens. All 48 genotypes included in the mini gardens were also planted in the maxi gardens. In this study, our analysis focuses on the mini gardens, which allows us to compare phenotypes and fitness metrics across a wide range of environments while controlling for genetic effects by using replicated clonal genotypes; we use the maxi gardens for model evaluation (see statistical methods below).

### Phenotypic data

Height was measured each year (2021–2023) prior to bud flush and after budset. Using these data, annual growth increment was calculated per year based on the height accumulated during the growing season (pre-bud flush height subtracted from post-budset height). Some gardens experienced herbivory, disease, and accidental mechanical damage leading to height measurements decreasing from bud flush to bud set in some individuals. As these negative growth increments likely represent the consequences of measurement error or herbivory, they were removed prior to analyses. Given the frequency of herbivory, it is likely that some growth increment values are underestimates of potential cumulative growth.

### Climate data

Climate data were extracted from ClimateNA (Wang et al. 2016; AdaptWest Project 2022) for genotype provenances (locations of origin) and common garden environments using terra 1.8–21 (Hijmans 2025) in R version 4.4.2 (R Core Team 2024). For provenances, we extracted historical climate data for the 30-year period from 1961–1990. We focused on average historical climate data because variation in climate response is partially the result of selection associated with climate of origin over the lifespan of the collected adult trees. For garden climates, we extracted the average climate data associated with each year of data collection, starting when the seedlings were planted in 2020. To identify climatic variables contributing to variation in growth across gardens, we fit LOESS curves of each climate variable against growth increment measurements using the loess function in base R. We selected mean coldest month temperature (MCMT) for use in statistical modeling because it corresponded to variation in performance across gardens and because the range of MCMT values at the common garden sites encompassed climates for most natural populations (see Results), enabling us to predict growth and mortality responses to MCMT within much of the native range of the two poplar species. We did not use precipitation variables in statistical modeling because most gardens were irrigated during the first year and some drier gardens (OLLU, SWMN, and UCM) continued irrigation in later years to ensure survival, limiting the selective effect of natural precipitation variation across gardens.

### Genomic data

Whole-genome sequence data associated with each genotype and previously described in Bolte et al. (2024) was used in phenotypic prediction across common gardens. DNA extraction, sequencing, and variant filtration are described in Supplementary Methods and filtering scripts are available at https://github.com/alaynamead/poplar_hybrid_vcf_filtering. A total of 334,657 variable sites were used to characterize genetic variation and admixture. Bolte et al. (2024) identified three distinct lineages in this hybrid zone: *P. balsamifera*, coastal *P. trichocarpa*, and a separate interior *P. trichocarpa* lineage originating from an ancient admixture event between the two species. We incorporated this genetic variation into our predictions using the PC scores of each genotype from a PCA of the 334,657 SNPs created with vegan 2.6–8 in R (Oksanen et al. 2024), defining genetic structure as variation in genomic PC space. PC1 separates *P. balsamifera* and the two *P. trichocarpa* lineages, PC2 separates all three lineages, and PC3 separates the northern (Alaska and Cassiar) and southern (Chilcotin, Jasper, and Crowsnest) transects (Figure S4). To visualize how responses varied by species ancestry across the repeated contact zones, we assigned species ancestry for each genotype using the proportions based on K=2 from the ancestry estimation software ADMIXTURE (Alexander et al. 2009). Across 48 genotypes, three genotypes were genetic outliers, possibly as a result of mixed ancestry with additional *Populus* species. These individuals were removed from the analyses, leaving a total of 45 genotypes for statistical modeling.

### Statistical model

We fit a linear mixed-effects model to evaluate the effects of garden climate, home climate, genetic structure and their interactions (G×E) on growth across the common gardens and to predict responses to future climates. We defined the environment as the MCMT of the common garden climate for the year of measurement, and we defined home climate as the historical average of MCMT at the provenance of each genotype. Genotypes may vary in their responses to climate as a result of local adaptation to their climate of origin and as a result of neutral genetic variation. Given this, we accounted for these sources of variation using different model variables. Previous studies have tested how climate of origin determines response to the environment within a species, essentially using climate of origin as a proxy for genotype to model G×E, assuming that much of the response is explained by adaptation to the local environment of origin (O’Neill et al. 2008; Wang et al. 2010). However, this approach does not account for variation in responses that result from other processes, such as neutral evolution resulting from historic demographic processes, selection for recent but not long-term climate patterns, or novel recombinants due to hybridization. We include genetic structure and climate of origin in the model to account for these multiple interacting factors that may influence the response to the environment.

We included data from the first two years of measurement (2021 and 2022) across 17 and 14 gardens, respectively, analyzing 2768 measurements of 1610 individual trees. We excluded 2023 data because gardens at the upper and lower temperature extremes were lost due to high mortality (Figure S1), and climatic extremes are important to provide “anchor points” to produce biologically realistic response curves (Wang et al. 2006). However, we did compare the model fit for each year individually and observed similar effect directions across all three years (Supplementary Figure S10). Individuals with a negative growth increment, attributed to herbivory or measurement error, were excluded from the statistical modeling (137 individuals in 2021 and 97 in 2022).

We included survival and yearly growth increment as two measures of performance within the same model by fitting a zero-inflated Gaussian model using glmmTMB 1.1.11 (Brooks et al. 2017) in R version 4.5.0 (R Core Team 2024). We set the growth of all trees marked dead at the end of each growing season as 0, resulting in a large number of zero values (381 trees in 2021 and 343 trees in 2022, 27% of the total). This zero-inflated model assumes that values of zero can have two origins: “sampling” zeros that are part of the distribution of growth increment (i.e., some individuals survived but had growth at or near zero) and “structural” zeros due to mortality (Hu et al. 2011; Brooks et al. 2017). This allowed us to evaluate how two separate fitness-associated traits, growth and mortality, respond to MCMT. Together their combined effect can be considered a proxy for overall fitness, incorporating both the probability of mortality and the predicted growth for surviving individuals. We report phenotypic predictions for three model components: the zero-inflated model representing mortality, the conditional model representing growth, and the overall model combining the two.

The full model included the fixed effects of garden MCMT, home MCMT, their square terms (to account for the observed quadratic response associated with a fitness optimum, Figure S2 and S3), and all interactions between garden and home MCMT and their square terms (indicating that the response to garden temperature varies based on temperature of origin). We also included genetic information as a fixed effect using the values for genetic PC1, PC2, and PC3 (Figure S4). MCMT was negatively correlated with genetic PC1 (R = −0.69), consistent with the climate niches associated with the two species, but was only weakly correlated with PC2 (R=−0.22) and PC3 (−0.40), so including multiple PCs enables us to identify variation in responses resulting from neutral genetic structure or adaptation to other climate variables. To test for G×E, we included interaction effects between garden climate and its square term with all genetic PCs. We also included the random effects of individual, genotype, year, and block nested within garden to account for non-independence of multiple observations of the same individual, genotype and garden site. The same formula was used for the zero-inflated portion of the model to test the effect of each factor on mortality. To improve model convergence, we scaled the numeric variables using the R function scale without centering them and retained the scaling factor used for each variable to back-transform scaled values to actual values. Growth increment was log-transformed to account for a distribution skewed towards lower values. Using each individual as a separate observation, we fit the following model in using the glmmTMB function, accounting for home MCMT, garden MCMT and their interactions and square terms; as well as genetic PCs 1–3 and their interaction terms with garden MCMT and its square term.

Model 1: $$\text{log}\;\left(\text{growth}\;\text{increment}\;+1\right)∼\;\text{garden}\;\text{MCMT}\;\times \;\text{home}\;\text{MCMT}\;+\;\text{garden}\;{\text{MCMT}}^{2}\times \text{home}\;{\text{MCMT}}^{2}+\;\text{garden}\;{\text{MCMT}}^{2}\times \text{home}\;\text{MCMT}\;+\;\text{garden}\;\text{MCMT}\;\times \;\text{home}\;{\text{MCMT}}^{2}+\text{genetic}\;\text{PC}1\;\times \;\text{garden}\;\text{MCMT}\;+\;\text{genetic}\;\text{PC}2\;\times \;\text{garden}\;\text{MCMT}\;+\;\text{genetic}\;\text{PC}3\;\times \;\text{garden}\;\text{MCMT}\;+\;\text{genetic}\;\text{PC}1\;\times \;\text{garden}\;{\text{MCMT}}^{2}+\text{genetic}\;\text{PC}2\;\times \;\text{garden}\;{\text{MCMT}}^{2}+\;\text{genetic}\;\text{PC}3\;\times \;\text{garden}\;{\text{MCMT}}^{2}+\;(1\;|\;\text{genotype})\;+\;(1\;|\;\text{garden}/\text{block})\;+\;(1\;|\;\text{year})\;+\;(1\;|\;\text{individual})$$

The random intercepts of genotype, block nested within garden, year, and individual tree were included and are indicated by parentheses. The significance of each term was evaluated using the tab_model function from the R package sjPlot 2.8.17 (Lüdecke 2024), which uses a type II Wald chi square test.

### Model evaluation

To evaluate whether the model can accurately predict poplar phenotypic response to environmental conditions, we compared its predictions of growth and mortality to actual measures in two ways. First, we removed one of the garden environments from the dataset, trained the model on the remaining 16 gardens, and used the fitted model to predict height in the garden that was excluded (hereafter referred to as leave-one-out cross validation). Second, we trained the model using all 17 gardens and then predicted growth increment of 544 genotypes in the three maxi gardens, two of which also have a mini garden site that was included in the training data (Virginia and North Dakota) and one that does not have a mini garden (University of Vermont). 500 of these genotypes were novel genotypes not evaluated within the mini gardens. We predicted heights for individuals based on the full model using the predict function from the glmmTMB package. To quantify the model’s predictive ability for each year, we calculated the Pearson correlation between the actual heights and the predicted heights (conditional model) as well as the predicted heights when the probability of mortality was included (overall model). To calculate predictive ability for the conditional model, we removed the individuals with a measured height of zero (which is accounted for in the zero-inflated portion of the model). We also evaluated model predictive ability with and without the random effects of garden, block, and genotype. Including random effects (using option re.form=NULL in the predict function) accounts for the varying intercepts associated with each group, while excluding them and setting all random effects to zero (re.form = NA) makes predictions based on the fixed effects of climate and genetics only. When the model was used to predict growth in genotypes or gardens not included in the training set, a new random effect was predicted.

We also used leave-one-out cross-validation to compare the performance of three different models with different combinations of genetic and climatic information. If most of the variation among genotypes in response to garden environments as estimated by MCMT is explained by local adaptation, provenance MCMT could serve as a proxy for genetic structure, enabling genotype-specific predictions of responses to temperature without the need for genetic data. To determine whether genetic PCs or home temperature increased the predictive power of the model, we tested simplified versions of the full model described above: a genetics-only model with home temperature excluded to test the predictive power of the genetic PCs, and a temperature-only model with genetic information excluded to test the predictive power of home MCMT (Table 1). We trained the three models on datasets with each garden excluded and predicted responses in the excluded garden for 2021 and 2022, evaluating models using the Pearson correlation between actual and predicted heights as described above.

### Predicting the response to temperature

We used the above model to predict each genotype’s norm of reaction; i.e. the response to a continuous temperature gradient, allowing us to characterize responses to temperature across the hybrid zone. We predicted the height and mortality probability of each genotype across a range of 100 equally-spaced MCMT values from −23.9 to 9.8 °C, encompassing both historic 30-year home climates (−23.9 to −3.8 °C) and yearly garden climates ( −16.5 to 9.8 °C). Phenotypic predictions should be more reliable for the warmer climatic range included in the common gardens, where phenotypic data was collected, than for the coldest parts of the home range. If so, this would increase confidence in our ability to predict responses to warming climates. We predicted the phenotypic response of each genotype across this climatic range of MCMT, accounting for genotype-specific responses with the genetic PCs and the home MCMT of that genotype. The glmmTMB predict function was used, ignoring the random effects of genotype, block, and garden and predicting the overall response to temperature rather than the genotype- or garden-specific response (option re.form = NA). Predictions were made for three phenotypes: the conditional model reflecting growth increment, the zero-inflated model predicting the probability of mortality, and the overall model incorporating both.

We estimated fitness changes under future values of MCMT using the norm of reaction generated from the model to predict the growth, mortality, and combined fitness of each genotype at its provenance under historic (1961–1980) and future values of MCMT. Future MCMT values were predicted from an ensemble containing 13 general circulation models (GCMs) from the CMIP6 database, generated from ClimateNA (Wang et al. 2016) and available at AdaptWest (AdaptWest Project 2022). We used the time period of 2041–2070 under the shared socioeconomic pathway (SSP) 2–4.5, an intermediate scenario in which emissions rise until mid-century, then decline (IPCC 2023). To predict how the relative fitness of *P. balsamifera*, *P. trichocarpa*, and hybrid genotypes may change under future values of MCMT, we predicted which genotypes would have the best performance (highest overall fitness combining growth and survival) across the species ranges and regions with common gardens. For each grid cell, we extracted MCMT values for past and future climates, and identified the genotype with the highest predicted fitness at that temperature.

## Results

### Variation in growth among gardens

Growth increment varied widely among gardens (Figure S1), with the highest growth occurring at WSU (Washington) and SU (Maryland) gardens, and the lowest growth occurring at the upper and lower winter temperature extremes, particularly at OLLU (Texas), UCM (California), OSU (Oregon), and NDSU (North Dakota). Genotypes typically reached their greatest height at intermediate temperatures warmer than those of their climate of origin, with height decreasing as MCMT increased or decreased (Figure S1 and S2), suggesting that MCMT exerts selection pressure on poplars, as previously found in high-latitude tree species (Leites et al. 2012, 2019; Yeaman et al. 2016; Rehfeldt et al. 2018; Mahony et al. 2020).

### Factors predicting growth and mortality

**Figure 2.**
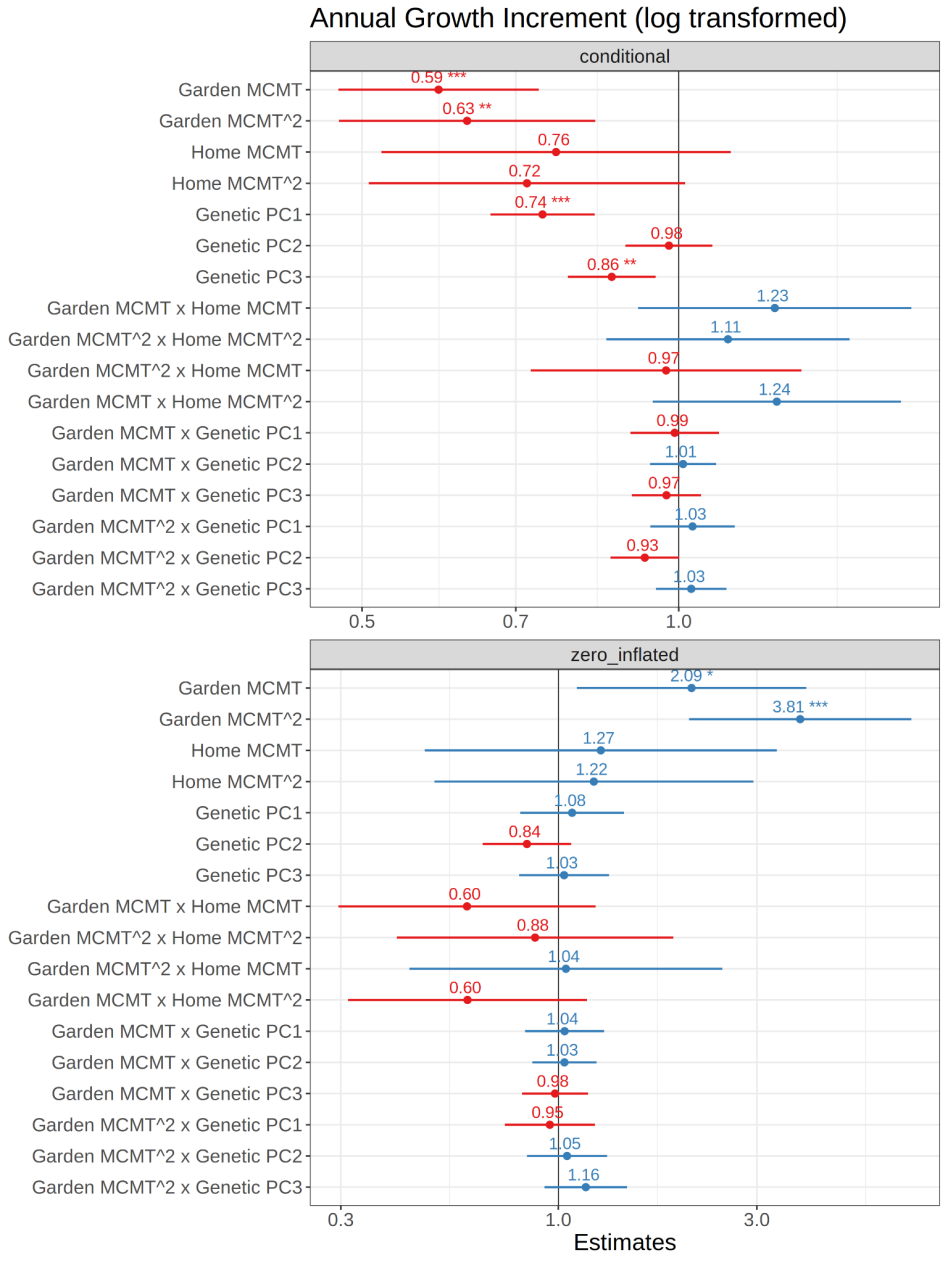
Forest plot of model coefficients using sjPlot (Lüdecke 2024); with an estimate of one indicating no effect, and estimates significantly greater or less than one indicating a positive and negative effect, respectively. Coefficients are standardized to allow comparison when interaction effects are included. Top plot shows results for the conditional model (growth increment) and bottom plot shows the estimates for the zero-inflated model (mortality); the effects for the two models are reversed because the zero-inflated model predicts the probability of mortality, which is negatively correlated with growth. P-values are indicated as follows: *<0.05, **<0.01, ***<0.001.

We evaluated the effect size and significance of each model factor for the conditional model, representing growth, and for the zero-inflated model, representing the probability of mortality (Figure 2 and Table S1). The two models had similar effects, indicating that the same factors associated with increased growth were also associated with low mortality. MCMT of the common garden site and its square term had the strongest effect on growth (Figure 2 and Table S1). Both had negative effects, indicating decreased growth in climates warmer or colder than the optimum temperature. Intra- and interspecific genetic structure represented by PC1 and PC3 both had significant effects on growth, although with a smaller effect size than garden temperature. Individuals with greater *P. trichocarpa* ancestry (PC1) and from the three southernmost transects (PC3) had higher growth on average. PC2, which explains genetic structure within *P. trichocarpa* (Figure S4), was not statistically significant, suggesting that genetic differences between interior and coastal *P. trichocarpa* did not significantly affect growth. Home temperature and its interaction with garden temperature did not significantly affect growth, suggesting that responses to MCMT were better explained by genetic structure than by MCMT of origin. While no genotype × environment interaction terms were significant at the p<0.05 level, Garden MCMT^2^ × Genetic PC2 was near significant (p=0.052, Table S1) and was significant when only measurements from 2021 were included in the model (Figure S10), suggesting a weak effect of variation in the temperature response curve between two *P. trichocarpa* lineages during first year growth (Figure S4). Overall, our results show that growth is primarily determined by temperature effects. Genetics also contributed to variation in growth, which suggests the response to temperature is partially determined by species ancestry and latitudinal variation within both species. Similarly, in the zero-inflated model, garden temperature significantly affected the probability of mortality, with increased mortality in sites at the higher and lower temperature extremes. Genetic structure, home MCMT, and their interactions with garden MCMT were not significant, suggesting that mortality probability was primarily driven by planting site temperature, with limited variation among genotypes.

### Evaluation of model predictive ability

We evaluated the predictive ability of the full model that included garden climate, climate of origin, genetic structure, and their interactions (GxE) (Table 1) using the Pearson correlation between the actual and predicted heights for all individuals. Both models generally performed well in predicting the response to climate: correlation between actual and predicted heights when random effects were included in the prediction was 0.795 for the overall model, and 0.809 for the conditional model (Figure S6). When random effects were not incorporated, predictive ability decreased to 0.631 and 0.538 for the overall and conditional model, respectively (Figure S6), indicating there were garden-specific effects on plant response that were not accounted for by garden MCMT, and/or genotype-specific effects that were not accounted for by home MCMT or the genetic PCs, and year-specific effects. However, the model consistently underpredicted actual growth increment values, particularly for the tallest trees. For this reason we focus on the relative differences in growth among genotypes and gardens rather than predicting specific yearly growth increments.

We also evaluated models by testing their predictive power for environments and genotypes not included in the input “training” dataset, using two methods. First, leave-one-out cross validation was performed for each garden; we report prediction ability for each garden for the conditional model with random effects of garden and genotype incorporated into the predictions. Prediction ability (Pearson’s correlation between observed and predicted heights, with dead individuals excluded) varied widely among gardens (Figure S7), with the average being 0.49 for 2021 and 0.38 for 2022, the highest correlation being 0.67 for OSU in 2022 (Oregon) and the lowest correlation being −0.8 for NDSU in 2022 (North Dakota). Most sites with poor predictions were sites with high mortality (NDSU, OLLU, LOCK, UCM, and OSU had >40% mortality in the first year). Other sites with low prediction ability included SWMN (r=0.058 in 2021,) and WI (r=0.4 in 2021, −0.09 in 2022), which are two sites having low MCMT (Figure S2) and therefore may provide unique information on responses to lower temperatures.

**Figure 3.**
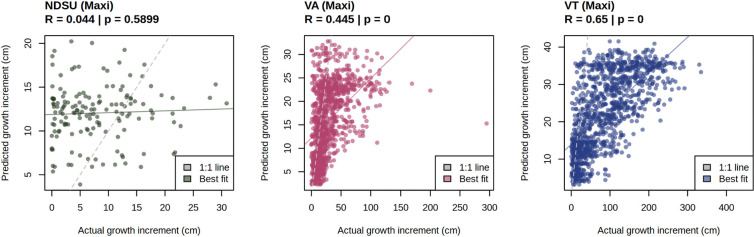
Performance of model predictions in the maxi gardens, predicting growth across 544 genotypes (500 novel genotypes) and one novel environment (VT) using a model trained on all 17 mini gardens. Models were evaluated by comparing predicted and actual height for the conditional model with no random effects for garden and genotype. As the conditional portion of the model predicts growth rather than mortality, individuals that did not survive were removed from the model. R values and p-values are given for the Pearson correlation between actual and predicted height.

Second, we predicted growth for our three ‘maxi’ common garden experiments, which included 500 novel genotypes and one novel site using the model trained on all 17 mini gardens (Figure 3). Prediction ability was low for NDSU, likely due to high mortality (Pearson’s r=0.04 for the conditional model). However, prediction ability for the VA and VT predictions was relatively high. For the conditional model VA had correlation between actual and predicted values of 0.45 and VT 0.65 (Figure 3), and when dead individuals were included in the overall model VA had an prediction ability of 0.48 and VT 0.53 (Figure S8). However, as with the leave-one-garden-out tests, the model underpredicted height. Taken together, these results suggest that for environments with low mortality, the model can predict relative performance for both novel genotypes and novel environments outside of the training dataset, but is limited in its prediction of specific growth increments.

### The role of genetics in phenotypic prediction across environments

**Figure 4.**
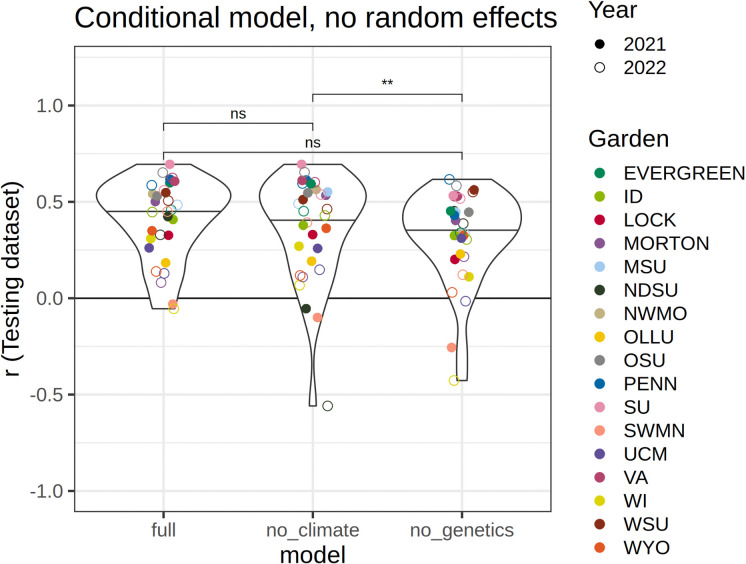
Comparison of model performance among the full model and those excluding provenance MCMT or genetic PCs (Table 1), estimated as the correlation between predicted and observed growth increments for each garden and year predicted using the leave-one-out models, in which heights were predicted for a single year and garden using a model trained on other gardens. The model including genetic data without home temperature data resulted in better predictive ability than the model with temperature data alone. Other comparisons with the overall model and with random effects included are shown in Figure S9. ** indicates p ≤0.01, “ns” indicates not significant.

When predicting growth and overall performance for each garden and year using leave-one-out cross validation, the climate-only model that excluded genetic information had worse performance than the genetics-only model (p=0.0039) and on average had lower performance than the full model, but did not significantly differ from it (p = 0.061) (Table 1, Figure 4). These results show that genetic information improved predictions when climate data was excluded, but that models with either genetics or climate data missing performed similarly to the full model. When random effects of garden and genotype were included in the predictions, all models performed equally well (Figure S9). This is likely because all genotypes were included in the training datasets allowing the intercept of each genotype to be estimated, improving predictions of their growth in a novel environment without explicitly including genetic or climate information.

**Figure 5.**
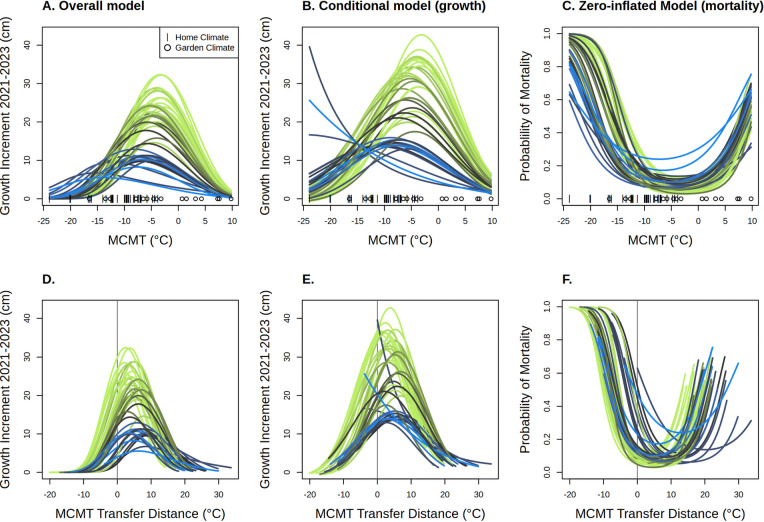
Model predictions for each genotype’s growth and mortality response to temperature, with each genotype indicated as a separate line colored by species ancestry at K=2, with green indicating *P. trichocarpa* and blue indicating *P. balsamifera*. Predictions for the overall model (A and D) incorporate both growth and the probability of mortality, predictions for the conditional model (B and E) only predict growth, ignoring the probability of zeros arising from other processes (mortality), and predictions for the zero-inflated model (C and F) predict the probability of zeros arising from mortality. A-C: Responses across the range of mean coldest month temperature (MCMT) values at garden and home climates. Actual values of home climates (|) and garden climates (circle) used for model training are shown on the x-axis. D-E. Responses based on distance from climate of origin (garden - home MCMT); positive values indicate a warmer climate (higher coldest month temperature) and negative values indicate a colder climate. If genotypes perform best in environments similar to their home environment, growth should be highest and mortality should be lowest when the transfer distance is 0.

Using the full model trained on all 17 gardens, we predicted the response of each genotype to a range of temperatures (Figure 5). Our model predicts that genotypes with more *P. trichocarpa* ancestry have higher growth and overall performance in warmer environments (MCMT ≈ −14 to 10 °C) compared to *P. balsamifera* genotypes, but that genotypes with more *P. balsamifera* ancestry have higher growth in colder climates, consistent with the climatic preferences of the two species (Figure 5A–B). Two majority-*P. balsamifera* genotypes were predicted to have biologically unrealistic reaction norms, with growth increasing exponentially with decreased winter temperatures (Figure 5B). These modeled growth responses likely reflect a preference for very cold environments that are not well-represented in our gardens, and when the effects of growth and mortality are combined, these genotypes show realistic response curves with optima at relatively low temperatures. Across species ancestries, mortality is predicted to increase at the colder and warmer extremes, with a greater probability of mortality under extreme cold climates than warmer climates (Figure 5C). As with predictions of growth, mortality responses predict that *P. balsamifera* is more cold tolerant, with increased probability of mortality *P. trichocarpa* genotypes occurring at less extreme cold temperatures than for *P. balsamifera* (Figure 5C). Likewise, the probability of mortality for *P. balsamifera* generally increases with warmer temperatures more than *P. trichocarpa*. Combining the growth and mortality predictions as an overall fitness proxy, genotypes with a majority *P. trichocarpa* ancestry have consistently higher fitness except in the coldest climates, where majority-ancestry *P. balsamifera* genotypes are predicted to outperform them (Figure 5A). The overall maximum growth of *P. balsamifera* genotypes is lower than that of *P. trichocarpa*, which has previously been reported to be a faster-growing species. Admixed genotypes with majority *P. trichocarpa* ancestry had maximum heights intermediate to the two parental species, while admixed majority-*P. balsamifera* genotypes had responses more similar to parental *P. balsamifera* genotypes. For most genotypes, the highest growth and lowest mortality occurred in climates warmer than their climate of origin (Figure 5).

### Rangewide projections of future fitness changes

**Figure 6.**
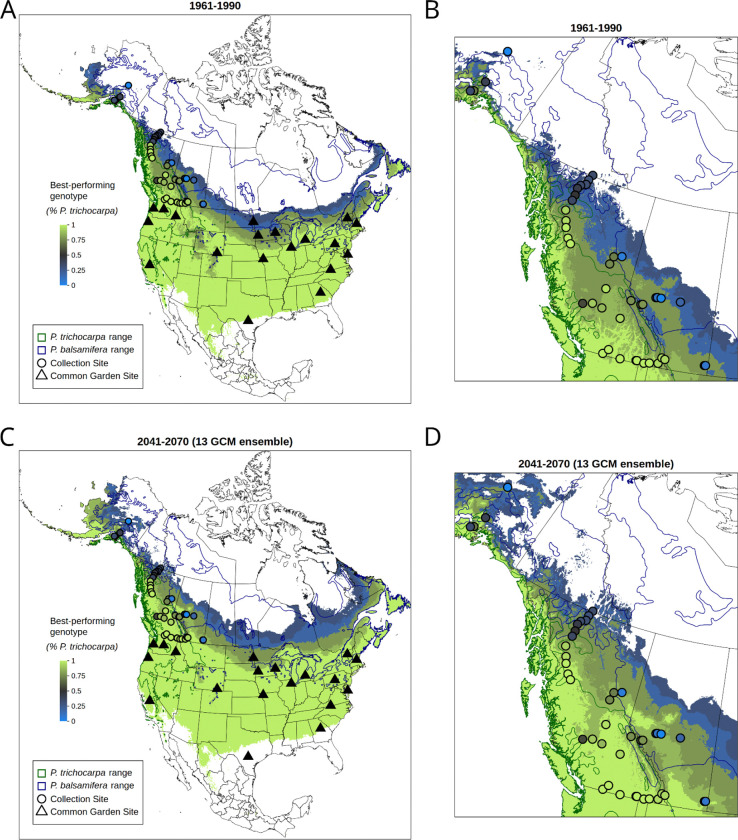
Maps showing the species ancestry of the studied genotype which is predicted to have highest fitness (as measured by growth and mortality) in that location under historic (A and B) and future climate (C and D), indicated by the color of the base layer. A and C show predictions for the species ranges and studied locations, and B and D show the same predictions for the sampled hybrid zone region. Regions with MCMT values outside of the range measured in the common gardens (−13.05 to 10.85 °C) are masked and colored white. Actual ancestry of collected genotypes are shown as circles, and locations of common gardens are shown as triangles. Species ranges are shown as dark blue and green outlines (Little 1971). As MCMT increases, we predict that genotypes with higher *P. trichocarpa* ancestry may be able to outcompete genotypes with higher *P. balsamifera* ancestry in some portions of the *P. balsamifera range*, favoring a northeastern shift of the *P. trichocarpa* range and the hybrid zone and into historically colder, more continental regions.

Our model predicts increased growth and survival under climates with MCMT temperatures warmer than the climate of origin (Figure 5D–F and Figure S11). However, we are limited in our ability to predict responses to multivariate changes in climate. Here, we focus on relative performance of genotypes rather than absolute changes in performance metrics. Predictions of the best-performing genotype under historic values of MCMT generally follows previously-described species ranges; with *P. trichocarpa* occurring along the west coast of North America and *P. balsamifera* occurring in the more northern and interior regions of the continent (Figure 6). Hybrid genotypes are predicted to outcompete parental species in intermediate regions of the contact zone, particularly in central British Columbia, where sampled genotypes exhibited high levels of admixture. Under future climates (2041–2070 for a “middle-of-the-road” emissions scenario), the model predicts shifts in the best-performing genotype following the two species’ climatic preferences: the regions suitable for *P. trichocarpa* and its backcrosses will shift northward as climate warms (Figure 6). For example, interior sites where majority-*P. balsamifera* genotypes were sampled are predicted to become more favorable to increased *P. trichocarpa* ancestry, which could favor introgression of *P. trichocarpa* genes into these regions.

## Discussion

Using 17 common gardens with *Populus* genotypes which originated from a hybrid zone spanning a broad temperature range, we evaluated the genotype-specific response to warming winter temperatures and predicted future responses across the hybrid zone. We found that 1) Fitness metrics and reaction norms varied among genotypes due to species ancestry and region of origin, consistent with a trade-off between cold tolerance and growth potential in warmer environments, 2) hybrids displayed reaction norms and temperature optima intermediate between their parental species, suggesting that warming temperatures could favor movement of the hybrid zone, and 3) a significant variance in growth explained by genetic structure illustrates the importance of genetic variation in predicting responses to climate change.

### Question 1: Do genotypes from different climates vary in fitness responses across environments?

Local adaptation to climate can shape not only traits, but their reaction norms across environments, contributing to variation in plastic responses within and across species (Patsiou et al. 2020). We found that poplar growth and survival across common garden environments was predicted by species ancestry and by genetic differences associated with a northern and southern region within each species (Figure 2), illustrating that genetic variation will contribute to spatially varying responses to warming temperatures (Patsiou et al. 2020; Martinez del Castillo et al. 2024). Performance traits varied along a continuous ancestry gradient between the two parental species, consistent with their climatic niches. In general, individuals with greater *P. trichocarpa* ancestry had greater overall growth, but these genotypes experienced lower growth and increased mortality in cold, continental environments typical of the *P. balsamifera* range (Figure 5). In addition, genotypes from the three southernmost transects had higher maximum heights than those from the two northern transects (as described by genetic PC3). This is consistent with a tradeoff between growth potential and cold tolerance often observed in temperate or boreal tree species, likely enabling *P. balsamifera* to outcompete *P. trichocarpa* in colder regions (Leites et al. 2012; Menon et al. 2015; Rehfeldt et al. 2018). While no G×E terms were significant in the model based on two years of data, the effect of garden MCMT^2^ × genetic PC2 was near significant (p = 0.052) (Figure 2, Table S1) and was significant when only measurements from 2021 were included in the model (Figure S10). This result suggests a weak effect of genetic variation between two *P. trichocarpa* lineages on the plastic response to winter temperatures, which may be partially explained by maternal effects expressed in the first year of growth. Additionally, optimum temperatures varied for each genotype, with *P. trichocarpa* individuals reaching maximum heights in warmer environments than *P. balsamifera* (Figure 5). This suggests that selection associated with minimum temperatures may be acting to produce different plastic responses across the range of both species and their hybrid zone. While the majority of *P. balsamifera* genotypes had lower overall growth, their climatic range appeared to be wider (Figure 5), perhaps reflecting adaptation to more continental climates with larger annual temperature ranges (Figure 1). Generally, the response of hybrid genotypes was intermediate between the parental species; however, some hybrid genotypes have reduced probability of mortality at lower temperatures (Figure 5), suggesting that introgression could promote increased cold hardiness (Hamilton et al. 2013). However, the effects of species ancestry and geography may be difficult to disentangle, as genotypes from the coldest part of the sampled range (Alaska and northern British Columbia) are admixed (Figure S4).

In this study, we find that most genotypes reach their fitness optima in environments that are warmer than their climate of origin (Figure 6), and that genotypes planted at sites having MCMT values similar to their home site did not always perform better than other, nonlocal genotypes (Figure S12). Taken together, these results suggest genotypes may not be locally adapted to winter temperatures alone when considering either the “home vs away” or “local vs foreign” criteria (Kawecki and Ebert 2004). This decoupling between the physiological optimum, the climate in which genotypes reach their maximum growth rate, and the ecological optimum, the climate in which they exist in the ecosystem, has been previously observed for temperate tree species (Rehfeldt et al. 2018). Higher growth rates in non-local environments does not necessarily indicate a lack of local adaptation (Kawecki and Ebert 2004), but provides important context for predicting responses to climate change, which often assume optimal performance in local climates. Instead, we found that most genotypes, when in gardens with temperatures similar to their climate of origin, were outperformed by a small number of primarily *P. trichocarpa* genotypes. These genotypes may be ideal for planting as part of restoration projects using assisted gene flow or for biofuel production across diverse environments, particularly under increasingly variable and unpredictable climate futures (Sannigrahi et al. 2010; Porth and El-Kassaby 2015; Mahoney et al. 2019). However, if increased growth corresponds to a lack of cold tolerance or lower survival and fecundity (Leites et al. 2012, 2019; Yeaman et al. 2016; Rehfeldt et al. 2018; Mahony et al. 2020), caution is warranted when planting in locations susceptible to cold stress. Further work comparing the cold tolerance of these genotypes could determine the lower temperature limits where they are likely to be successful. Regardless of the mechanisms underlying genetic variation in fitness proxies, the large differences in responses to temperature illustrate the importance and benefit of measuring reaction norms across wide geographic regions within and among species.

Our predictions of increased growth under warming climates for these *Populus* genotypes raises the question: is active conservation and management of poplar in this region necessary, or should we prioritize species more vulnerable to increased temperatures? However, predictions of increased growth should be treated with caution, as they only represent aboveground height and mortality in the first two years of growth and exclude reproductive measures of fitness and belowground biomass (Fischer et al. 2007), which could vary between the species given the presence of *P. balsamifera* in drier environments (Figure 1B). Additionally, we only modeled growth over two growing seasons, in relatively warm climates and with irrigation during the first year, limiting the selective response to cold or drought stresses that genotypes may experience in their home environments. Major selective weather events may occur only rarely; for example, an unusually cold winter could select against genotypes with high growth and low cold tolerance, favoring cold-tolerant genotypes over longer timescales (Rehfeldt et al. 2018; Lowry et al. 2019). In this case, our dataset may underestimate the benefit that *P. balsamifera* has in colder regions. Similarly, if warming winters are accompanied by summer heat waves, the benefits of warming we observe may not persist long-term in wild populations. We also did not consider biotic factors such as competition with more cold-hardy conifers or pathogen pressure that may exclude *Populus* from warmer climates (La Mantia et al. 2013; Suarez-Gonzalez et al. 2018a), or interactions with belowground communities (Fischer et al. 2014b). If biotic interactions shift along with climate change, overall fitness could decrease even if higher temperatures favor faster aboveground growth. For example, pressure from leaf rust (*Melampsora* spp.) may be higher in warmer, more southern regions of the *P. trichocarpa* range (La Mantia et al. 2013; McKown et al. 2014; Suarez-Gonzalez et al. 2018). Lastly, our predictions of the response to climate are limited by the training data used to create the model – in this case, the climatic range of the common gardens (Rogers and Holland 2021). Because the gardens were largely planted to the south and in warmer climates than the climate of origin, most temperature variables (including multivariate PC axes) had little overlap between home and garden climates, limiting our ability to make predictions for growth and mortality in the colder historic climates of origin. We choose to use MCMT as the predictor variable because of its biological importance in past studies (Leites et al. 2012, 2019; Yeaman et al. 2016; Rehfeldt et al. 2018; Mahony et al. 2020) and because garden climates included temperatures matching historic home climates for both *P. balsamifera* and *P. trichocarpa* genotypes (Figure 1B–C, Figure 5). However, we lack common gardens matching the coldest collection sites, limiting our predictions for the multivariate home environments experienced by natural populations, including some genotypes studied here. Given these limitations, testing how native poplar populations are responding to climate change *in situ* could complement common garden studies (Sharma et al. 2022; Astigarraga et al. 2024; Martinez del Castillo et al. 2024).

### Question 2: How will warming temperatures alter the geography of the two species and their hybrid zone?

Geographic variation in the magnitude of climate change and differences in phenotypic plasticity among genotypes could alter regions where certain genomic backgrounds outcompete others, potentially leading to range shifts for the two species and their hybrid zone. While we predict that local poplar populations across the sampled region could experience increased growth and decreased mortality from increased winter temperatures, they must contend with competition from non-local genotypes that may have greater fitness under novel climates than local genotypes. Species ranges are already beginning to shift in response to climate change (Parmesan 2006; Bell et al. 2014; Astigarraga et al. 2024) and multi-species hybrid zones may also shift (Taylor et al. 2014, 2015; Billerman et al. 2016; Hamilton and Miller 2016; Wielstra 2019; Alexander et al. 2022). If dispersal of poplar genotypes is fast enough to track changes in climate, regions where particular genomic backgrounds dominate may shift under climate change. For example, under higher minimum winter temperatures, *P. balsamifera* may be outcompeted by less cold-tolerant but faster growing genotypes with higher *P. trichocarpa* ancestry (Figure 6). However, such shifts in genetic composition would rely on gene flow or migration being fast enough to track climate change, either through dispersal of genotypes or seeds or wind-dispersed pollen (Corlett and Westcott 2013). While *Populus* can likely disperse over long distances via wind dispersal or via river-dispersed vegetative material (Kling and Ackerly 2021), geographic barriers to gene flow persist across the hybrid zone, particularly in mountainous regions (Bolte et al. 2024). Our model predicts that parental *P. trichocarpa* genotypes may begin to outcompete *P. trichocarpa* backcrosses in central British Columbia, a region with high gene flow (Bolte et al. 2024), so this region may see shifts in the genetic composition of poplars (Figure 6). Conversely, while the regions where *P. trichocarpa* ancestry is favored are predicted to shift to the northeast in Alberta, the Rocky Mountains are a barrier to gene flow that may prevent their dispersal (Bolte et al. 2024). Our predictions in colder regions, including the *P. balsamifera* range, are likely limited because our dataset includes few parental *P. balsamifera* genotypes and we did not measure growth in gardens with cold climates matching this part of the *P. balsamifera* range: as a result, it predicts backcrossed *P. balsamifera* genotypes would perform better than parental *P. balsamifera* in northern, interior North America within the core of the species distribution (Figure 6). Because the reaction norms of parental and backcrossed *P. balsamifera* are very similar (Figure 5A), we interpret these as regions where increased *P. balsamifera* ancestry is beneficial, not necessarily that backcrosses will be significantly more successful than parental genotypes.

Spatial changes in the location of the hybrid zone could provide an opportunity for adaptive introgression to occur, enabling individual alleles and novel allelic combinations to track climate change. Ancient and contemporary introgression can allow species to persist as environments change (Kremer and Hipp 2020; Leroy et al. 2020; O’Donnell et al. 2021; Buck et al. 2023; Yang et al. 2023). The presence of admixed genotypes in intermediate climates in this *Populus* hybrid zone and the reaction norms that are intermediate between the two parental species are consistent with a the bounded hybrid superiority model, in which hybrids have higher fitness than either parental species in intermediate climates (Hamilton et al. 2013; De La Torre et al. 2014; Bolte et al. 2024). Movement of the *P. trichocarpa* x *P. balsamifera* hybrid zone could allow genes to move across species boundaries at new contact zones, increasing the levels of standing genetic variation in these regions and facilitating introgression of alleles that are beneficial under novel climates into new regions.

### Question 3: What information is needed to predict climate change responses in trees?

Like generations of provenance studies, we find that the environment has a large effect on tree performance (O’Neill et al. 2008; Wang et al. 2010; Leites et al. 2012; Browne et al. 2019; Leites and Benito Garzón 2023; Ye et al. 2023). Understanding how growth and survival varies across species, populations, and environments will be essential to predicting shifts in the performance and carbon sequestration abilities of trees under changing climates. Because the temperature of the planting site had the greatest effect in determining growth, common garden experiments will continue to be essential to predicting phenotypic responses to the environment. We also show that a model including genetic PCs but not climate of origin significantly improved predictions of growth compared to a model containing only home and garden climates (Figure 5), suggesting that universal transfer functions for predicting tree growth (O’Neill et al. 2008; Wang et al. 2010), could benefit from genetic information, as found by other studies (Mahony et al. 2020; Archambeau et al. 2022; Putra et al. 2023; Li et al. 2024). Furthermore, the model that included genetic information but not climate of origin predicted growth equally as well as the full model, suggesting genetic structure may be used to effectively model responses to climate in this system. Genetic isolation-by-environment is stronger than isolation-by-distance in this system (Bolte et al. 2024), suggesting environmental adaptation drives much of the observed genetic structure. Conversely, in species with high gene flow and less structured populations, adaptation to climate may be the primary factor contributing to intraspecific variation, and genetic structure may not be as effective in predicting responses.

## Conclusions

Across 17 common garden experiments spanning a wide climatic range, the performance of *Populus* genotypes was explained by genetic variation associated with species ancestry and origin from northern or southern regions. Genotypes with greater *P. trichocarpa* ancestry and from southern regions had higher growth potential, but at a cost of decreased growth and increased mortality in cold regions typical of *P. balsamifera*. By characterizing genotype-specific reaction norms, we were able to predict which genotypes should have the highest performance in historic and future climates across the hybrid zone, identifying regions where the contact zone is favored to shift under warming winter temperatures. If migration and gene flow is able to track climate change, a moving hybrid zone could facilitate adaptive introgression of alleles beneficial under novel climates, enabling poplar populations to persist in their ecosystems even as their genetic makeup may change. This study shows the large effect that environment has on performance, illustrating the continued value that common garden experiments will have in predicting organisms’ responses to climate change. Furthermore, using genomic data, we were able to characterize how genetic structure shapes varying responses within and among species, enabling predictions of climate change responses to take into account the genetic variation in traits that exists in natural populations.

## Supplementary Material

Supplement 1

Supplement 2

Supplement 3

Supplement 4

Supplement 5

## Figures and Tables

**Table 1.** Factors included in each model used for testing whether home climate can be a proxy for genetic structure in modeling the response to environment

	E: Garden MCMT (quadratic)	G: Genetic PCs 1–3	GxE (genotype x garden climate interaction)	Home MCMT as a proxy for G (quadratic)	Home MCMT x E
Full	x	x	x	x	x
Genetics only	x	x	x		
Climate only	x			x	x

## Data Availability

Scripts for filtering genomic data are available at github.com/alaynamead/poplar_hybrid_vcf_filtering. Analysis scripts are available at github.com/alaynamead/popup_poplar_reaction_norms and corresponding R markdown files with script outputs are provided as supplementary material. Github repositories will be archived at Zenodo upon manuscript acceptance. Data from this study is included as supplementary material and will be archived in Dryad upon manuscript acceptance.

## References

[R1] AdaptWest Project. 2022. Gridded current and projected climate data for North America at 1km resolution, generated using the ClimateNA v7.30 software (Wang T. , 2022). Available at adaptwest.databasin.org.

[R2] Aitken S. N., and Bemmels J. B.. 2016. Time to get moving: assisted gene flow of forest trees. Evolutionary Applications 9:271–290. 27087852 10.1111/eva.12293 PMC4780373

[R3] Aitken S. N., and Whitlock M. C.. 2013. Assisted gene flow to facilitate local adaptation to climate change. Annu. Rev. Ecol. Evol. Syst 44:367–88.

[R4] Alexander A., Robbins M. B., Holmes J., Moyle R. G., and Peterson A. T.. 2022. Limited movement of an avian hybrid zone in relation to regional variation in magnitude of climate change. Molecular Ecology 31:6634–6648. 36210655 10.1111/mec.16727 PMC9729445

[R5] Alexander D. H., Novembre J., and Lange K.. 2009. Fast model-based estimation of ancestry in unrelated individuals. Genome Res 19:1655–1664. 19648217 10.1101/gr.094052.109 PMC2752134

[R6] Archambeau J., Benito Garzón M., Barraquand F., de Miguel M., Plomion C., and González-Martínez S. C.. 2022. Combining Climatic and Genomic Data Improves Range-Wide Tree Height Growth Prediction in a Forest Tree. The American Naturalist 200:E141–E159. The University of Chicago Press. 10.1086/720619 36150196

[R7] Arnold P. A., Kruuk L. E. B., and Nicotra A. B.. 2019. How to analyse plant phenotypic plasticity in response to a changing climate. New Phytologist 222:1235–1241. 30632169 10.1111/nph.15656

[R8] Astigarraga J., Esquivel-Muelbert A., Ruiz-Benito P., Rodríguez-Sánchez F., Zavala M. A., Vilà-Cabrera A., Schelhaas M.-J., Kunstler G., Woodall C. W., Cienciala E., Dahlgren J., Govaere L., König L. A., Lehtonen A., Talarczyk A., Liu D., and Pugh T. A. M.. 2024. Relative decline in density of Northern Hemisphere tree species in warm and arid regions of their climate niches. Proceedings of the National Academy of Sciences 121:e2314899121. Proceedings of the National Academy of Sciences. 10.1073/pnas.2314899121 PMC11252807 38954552

[R9] Bell D. M., Bradford J. B., and Lauenroth W. K.. 2014. Early indicators of change: divergent climate envelopes between tree life stages imply range shifts in the western United States. Global Ecology and Biogeography 23:168–180.

[R10] Billerman S. M., Murphy M. A., and Carling M. D.. 2016. Changing climate mediates sapsucker (Aves: Sphyrapicus) hybrid zone movement. Ecology and Evolution 6:7976–7990. 27878070 10.1002/ece3.2507 PMC5108250

[R11] Bolte C. E., Phannareth T., Zavala-Paez M., Sutara B. N., Can M. F., Fitzpatrick M. C., Holliday J. A., Keller S. R., and Hamilton J. A.. 2024. Genomic insights into hybrid zone formation: The role of climate, landscape, and demography in the emergence of a novel hybrid lineage. Molecular Ecology 33:e17430. 38867593 10.1111/mec.17430

[R12] Brooks M. E., Kristensen K., van Benthem K. J., Magnusson A., Berg C. W., Nielsen A., Skaug H. J., Mächler M., and Bolker B. M.. 2017. glmmTMB Balances Speed and Flexibility Among Packages for Zero-inflated Generalized Linear Mixed Modeling. The R Journal 9:378–400.

[R13] Browne L., Wright J. W., Fitz-Gibbon S., Gugger P. F., and Sork V. L.. 2019. Adaptational lag to temperature in valley oak (Quercus lobata) can be mitigated by genome-informed assisted gene flow. PNAS 116:25179–25185. National Academy of Sciences. 31767740 10.1073/pnas.1908771116 PMC6911187

[R14] Buck R., Ortega-Del Vecchyo D., Gehring C., Michelson R., Flores-Rentería D., Klein B., Whipple A. V., and Flores-Rentería L.. 2023. Sequential hybridization may have facilitated ecological transitions in the Southwestern pinyon pine syngameon. New Phytologist 237:2435–2449. 36251538 10.1111/nph.18543

[R15] Capblancq T., Fitzpatrick M. C., Bay R. A., Exposito-Alonso M., and Keller S. R.. 2020. Genomic Prediction of (Mal)Adaptation Across Current and Future Climatic Landscapes. Annual Review of Ecology, Evolution, and Systematics 51:245–269.

[R16] Chhatre V. E., Evans L. M., DiFazio S. P., and Keller S. R.. 2018. Adaptive introgression and maintenance of a trispecies hybrid complex in range-edge populations of Populus. Molecular Ecology 27:4820–4838. 30071141 10.1111/mec.14820

[R17] Chhetri H. B., Macaya-Sanz D., Kainer D., Biswal A. K., Evans L. M., Chen J.-G., Collins C., Hunt K., Mohanty S. S., Rosenstiel T., Ryno D., Winkeler K., Yang X., Jacobson D., Mohnen D., Muchero W., Strauss S. H., Tschaplinski T. J., Tuskan G. A., and DiFazio S. P.. 2019. Multitrait genome-wide association analysis of Populus trichocarpa identifies key polymorphisms controlling morphological and physiological traits. New Phytologist 223:293–309. 30843213 10.1111/nph.15777

[R18] Cooper H. F., Best R. J., Andrews L. V., Corbin J. P. M., Garthwaite I., Grady K. C., Gehring C. A., Hultine K. R., Whitham T. G., and Allan G. J.. 2022. Evidence of climate-driven selection on tree traits and trait plasticity across the climatic range of a riparian foundation species. Molecular Ecology 31:5024–5040. 35947510 10.1111/mec.16645

[R19] Cooper H. F., Grady K. C., Cowan J. A., Best R. J., Allan G. J., and Whitham T. G.. 2019. Genotypic variation in phenological plasticity: Reciprocal common gardens reveal adaptive responses to warmer springs but not to fall frost. Global Change Biology 25:187–200. 30346108 10.1111/gcb.14494

[R20] Corlett R. T., and Westcott D. A.. 2013. Will plant movements keep up with climate change? Trends in Ecology & Evolution 28:482–488. Elsevier. 23721732 10.1016/j.tree.2013.04.003

[R21] De La Torre A. R., Wang T., Jaquish B., and Aitken S. N.. 2014. Adaptation and exogenous selection in a Picea glauca × Picea engelmannii hybrid zone: implications for forest management under climate change. New Phytologist 201:687–699. 24200028 10.1111/nph.12540 PMC4285121

[R22] Des Marais D. L., Hernandez K. M., and Juenger T. E.. 2013. Genotype-by-environment interaction and plasticity: exploring genomic responses of plants to the abiotic environment. Annual Review of Ecology, Evolution, and Systematics 44:5–29.

[R23] Evans L. M., Slavov G. T., Rodgers-Melnick E., Martin J., Ranjan P., Muchero W., Brunner A. M., Schackwitz W., Gunter L., Chen J.-G., Tuskan G. A., and DiFazio S. P.. 2014. Population genomics of *Populus trichocarpa* identifies signatures of selection and adaptive trait associations. Nature Genetics 46:1089–1096. 25151358 10.1038/ng.3075

[R24] Fischer D. G., Chapman S. K., Classen A. T., Gehring C. A., Grady K. C., Schweitzer J. A., and Whitham T. G.. 2014a. Plant genetic effects on soils under climate change. Plant Soil 379:1–19.

[R25] Fischer D. G., Chapman S. K., Classen A. T., Gehring C. A., Grady K. C., Schweitzer J. A., and Whitham T. G.. 2014b. Plant genetic effects on soils under climate change. Plant Soil 379:1–19.

[R26] Fischer D. G., Hart S. C., LeRoy C. J., and Whitham T. G.. 2007. Variation in below-ground carbon fluxes along a Populus hybridization gradient. New Phytologist 176:415–425. 17888120 10.1111/j.1469-8137.2007.02167.x

[R27] Fitzpatrick M. C., Chhatre V. E., Soolanayakanahally R. Y., and Keller S. R.. 2021. Experimental support for genomic prediction of climate maladaptation using the machine learning approach Gradient Forests. Molecular Ecology Resources 21:2749–2765. 33683822 10.1111/1755-0998.13374

[R28] Fitzpatrick M. C., and Keller S. R.. 2015. Ecological genomics meets community-level modelling of biodiversity: mapping the genomic landscape of current and future environmental adaptation. Ecology Letters 18:1–16. 25270536 10.1111/ele.12376

[R29] Geraldes A., Farzaneh N., Grassa C. J., McKown A. D., Guy R. D., Mansfield S. D., Douglas C. J., and Cronk Q. C. B.. 2014. Landscape Genomics of Populus Trichocarpa: The Role of Hybridization, Limited Gene Flow, and Natural Selection in Shaping Patterns of Population Structure. Evolution 68:3260–3280. 25065449 10.1111/evo.12497

[R30] Grady K. C., Kolb T. E., Ikeda D. H., and Whitham T. G.. 2015. A bridge too far: cold and pathogen constraints to assisted migration of riparian forests. Restoration Ecology 23:811–820.

[R31] Gray L. K., Gylander T., Mbogga M. S., Chen P., and Hamann A.. 2011. Assisted migration to address climate change: recommendations for aspen reforestation in western Canada. Ecological Applications 21:1591–1603. 21830704 10.1890/10-1054.1

[R32] Hamilton J. A., Lexer C., and Aitken S. N.. 2013. Genomic and phenotypic architecture of a spruce hybrid zone (Picea sitchensis × P. glauca). Molecular Ecology 22:827–841. 22967172 10.1111/mec.12007

[R33] Hamilton J. A., and Miller J. M.. 2016. Adaptive introgression as a resource for management and genetic conservation in a changing climate. Conservation Biology 30:33–41. 26096581 10.1111/cobi.12574

[R34] Hijmans R. J. 2025. terra: Spatial Data Analysis.

[R35] Holliday J. A., Zhou L., Bawa R., Zhang M., and Oubida R. W.. 2016. Evidence for extensive parallelism but divergent genomic architecture of adaptation along altitudinal and latitudinal gradients in Populus trichocarpa. New Phytologist 209:1240–1251. 26372471 10.1111/nph.13643

[R36] Hord A. M., Fischer D. G., Schweitzer J. A., LeRoy C. J., Whitham T. G., and Bailey J. K.. 2025. Hybrid introgression as a mechanism of rapid evolution and resilience to climate change in a riparian tree species. Communications Biology. 10.1038/s42003-025-08410-3 PMC12331986 40775051

[R37] Hu M.-C., Pavlicova M., and Nunes E. V.. 2011. Zero-Inflated and Hurdle Models of Count Data with Extra Zeros: Examples from an HIV-Risk Reduction Intervention Trial. The American Journal of Drug and Alcohol Abuse 37:367–375. Taylor & Francis. 21854279 10.3109/00952990.2011.597280 PMC3238139

[R38] Ikeda D. H., Max T. L., Allan G. J., Lau M. K., Shuster S. M., and Whitham T. G.. 2017. Genetically informed ecological niche models improve climate change predictions. Global Change Biology 23:164–176. 27543682 10.1111/gcb.13470

[R39] IPCC. 2023. Climate Change 2023: Synthesis Report. Contribution of Working Groups I, II and III to the Sixth Assessment Report of the Intergovernmental Panel on Climate Change. , doi: 10.59327/IPCC/AR6-978929169164.

[R40] Janes J. K., and Hamilton J. A.. 2017. Mixing It Up: The Role of Hybridization in Forest Management and Conservation under Climate Change. Forests 8:237. Multidisciplinary Digital Publishing Institute.

[R41] Jansson S., and Douglas C. J.. 2007. Populus: A Model System for Plant Biology. Annual Review of Plant Biology 58:435–458. Annual Reviews. 10.1146/annurev.arplant.58.032806.103956 17280524

[R42] Kawecki T. J., and Ebert D.. 2004. Conceptual issues in local adaptation. Ecology Letters 7:1225–1241.

[R43] Keller S. R., Levsen N., Olson M. S., and Tiffin P.. 2012. Local Adaptation in the Flowering-Time Gene Network of Balsam Poplar, Populus balsamifera L. Molecular Biology and Evolution 29:3143–3152. 22513286 10.1093/molbev/mss121

[R44] Keller S. R., Olson M. S., Silim S., Schroeder W., and Tiffin P.. 2010. Genomic diversity, population structure, and migration following rapid range expansion in the Balsam Poplar, Populus balsamifera. Molecular Ecology 19:1212–1226. 20163548 10.1111/j.1365-294X.2010.04546.x

[R45] Keller S. R., Soolanayakanahally R. Y., Guy R. D., Silim S. N., Olson M. S., and Tiffin P.. 2011. Climate-driven local adaptation of ecophysiology and phenology in balsam poplar, Populus balsamifera L. (Salicaceae). American Journal of Botany 98:99–108. 21613088 10.3732/ajb.1000317

[R46] Kling M. M., and Ackerly D. D.. 2021. Global wind patterns shape genetic differentiation, asymmetric gene flow, and genetic diversity in trees. Proceedings of the National Academy of Sciences 118:e2017317118. Proceedings of the National Academy of Sciences. 10.1073/pnas.2017317118 PMC8092467 33875589

[R47] Kremer A., and Hipp A. L.. 2020. Oaks: an evolutionary success story. New Phytologist 226:987–1011. 31630400 10.1111/nph.16274 PMC7166131

[R48] La Mantia J., Klápště J., El-Kassaby Y. A., Azam S., Guy R. D., Douglas C. J., Mansfield S. D., and Hamelin R.. 2013. Association Analysis Identifies Melampsora ×columbiana Poplar Leaf Rust Resistance SNPs. PLOS ONE 8:e78423. Public Library of Science. 24236018 10.1371/journal.pone.0078423 PMC3827267

[R49] Leites L., and Benito Garzón M.. 2023. Forest tree species adaptation to climate across biomes: Building on the legacy of ecological genetics to anticipate responses to climate change. Global Change Biology 29:4711–4730. 37029765 10.1111/gcb.16711

[R50] Leites L. P., Rehfeldt G. E., and Steiner K. C.. 2019. Adaptation to climate in five eastern North America broadleaf deciduous species: Growth clines and evidence of the growth-cold tolerance trade-off. Perspectives in Plant Ecology, Evolution and Systematics 37:64–72.

[R51] Leites L. P., Robinson A. P., Rehfeldt G. E., Marshall J. D., and Crookston N. L.. 2012. Height-growth response to climatic changes differs among populations of Douglas-fir: a novel analysis of historic data. Ecological Applications 22:154–165. 22471081 10.1890/11-0150.1

[R52] Leroy T., Rougemont Q., Dupouey J.-L., Bodénès C., Lalanne C., Belser C., Labadie K., Le Provost G., Aury J.-M., Kremer A., and Plomion C.. 2020. Massive postglacial gene flow between European white oaks uncovered genes underlying species barriers. New Phytologist 226:1183–1197. 31264219 10.1111/nph.16039 PMC7166129

[R53] Li F., Gates D. J., Buckler E. S., Hufford M. B., Janzen G. M., Rellán-Álvarez R., Rodríguez-Zapata F., Navarro J. A. R., Sawers R. J. H., Snodgrass S. J., Sonder K., Willcox M. C., Hearne S. J., Ross-Ibarra J., and Runcie D. E.. 2024. The utility of environmental data from traditional varieties for climate-adaptive maize breeding. bioRxiv 2024.09.19.613351. Cold Spring Harbor Laboratory.

[R54] Little E. L. 1971. Atlas of United States trees. U.S. Department of Agriculture, Forest Service, Washington, DC.

[R55] Love S. J., Schweitzer J. A., Woolbright S. A., and Bailey J. K.. 2023. Sky Islands Are a Global Tool for Predicting the Ecological and Evolutionary Consequences of Climate Change. Annual Review of Ecology, Evolution, and Systematics 54:219–236. Annual Reviews.

[R56] Lowry D. B., Lovell J. T., Zhang L., Bonnette J., Fay P. A., Mitchell R. B., Lloyd-Reilley J., Boe A. R., Wu Y., Rouquette F. M., Wynia R. L., Weng X., Behrman K. D., Healey A., Barry K., Lipzen A., Bauer D., Sharma A., Jenkins J., Schmutz J., Fritschi F. B., and Juenger T. E.. 2019. QTL × environment interactions underlie adaptive divergence in switchgrass across a large latitudinal gradient. Proceedings of the National Academy of Sciences 116:12933–12941. Proceedings of the National Academy of Sciences. 10.1073/pnas.1821543116 PMC6600931 31182579

[R57] Lüdecke D. 2024. sjPlot: Data Visualization for Statistics in Social Science.

[R58] Mahoney S. M., Mike J. B., Parker J. M., Lassiter L. S., and Whitham T. G.. 2019. Selection for genetics-based architecture traits in a native cottonwood negatively affects invasive tamarisk in a restoration field trial. Restoration Ecology 27:15–22.

[R59] Mahony C. R., MacLachlan I. R., Lind B. M., Yoder J. B., Wang T., and Aitken S. N.. 2020. Evaluating genomic data for management of local adaptation in a changing climate: A lodgepole pine case study. Evolutionary Applications 13:116–131. 31892947 10.1111/eva.12871 PMC6935591

[R60] Martinez del Castillo E., Torbenson M. C. A., Reinig F., Tejedor E., de Luis M., and Esper J.. 2024. Contrasting Future Growth of Norway Spruce and Scots Pine Forests Under Warming Climate. Global Change Biology 30:e17580. 39548695 10.1111/gcb.17580

[R61] McKown A. D., Guy R. D., Klápště J., Geraldes A., Friedmann M., Cronk Q. C. B., El-Kassaby Y. A., Mansfield S. D., and Douglas C. J.. 2014a. Geographical and environmental gradients shape phenotypic trait variation and genetic structure in *Populus trichocarpa*. New Phytologist 201:1263–1276. 24491114 10.1111/nph.12601

[R62] McKown A. D., Guy R. D., Quamme L., Klápště J., Mantia J. L., Constabel C. P., El-Kassaby Y. A., Hamelin R. C., Zifkin M., and Azam M. S.. 2014b. Association genetics, geography and ecophysiology link stomatal patterning in *Populus trichocarpa* with carbon gain and disease resistance trade-offs. Molecular Ecology 23:5771–5790. 25319679 10.1111/mec.12969

[R63] McKown A. D., Klápště J., Guy R. D., Geraldes A., Porth I., Hannemann J., Friedmann M., Muchero W., Tuskan G. A., Ehlting J., Cronk Q. C. B., El-Kassaby Y. A., Mansfield S. D., and Douglas C. J.. 2014c. Genome-wide association implicates numerous genes underlying ecological trait variation in natural populations of *Populus trichocarpa*. New Phytologist 203:535–553. 24750093 10.1111/nph.12815

[R64] Menon M., Barnes W. J., and Olson M. S.. 2015. Population genetics of freeze tolerance among natural populations of *Populus balsamifera* across the growing season. New Phytologist 207:710–722. 25809016 10.1111/nph.13381

[R65] O’Donnell S. T., Fitz-Gibbon S. T., and Sork V. L.. 2021. Ancient Introgression Between Distantly Related White Oaks (Quercus sect. Quercus) Shows Evidence of Climate-Associated Asymmetric Gene Exchange. Journal of Heredity 112:663–670. 34508641 10.1093/jhered/esab053

[R66] Oksanen J., Simpson G. L., Blanchet F. G., Kindt R., Legendre P., Minchin P. R., O’Hara R. B., Solymos P., Stevens M. H. H., Szoecs E., Wagner H., Barbour M., Bedward M., Bolker B., Borcard D., Carvalho G., Chirico M., Caceres M. D., Durand S., Evangelista H. B. A., FitzJohn R., Friendly M., Furneaux B., Hannigan G., Hill M. O., Lahti L., McGlinn D., Ouellette M.-H., Cunha E. R., Smith T., Stier A., Braak C. J. F. T., and Weedon J.. 2024. vegan: Community Ecology Package.

[R67] O’Neill G. A., Hamann A., and Wang T.. 2008. Accounting for population variation improves estimates of the impact of climate change on species’ growth and distribution. Journal of Applied Ecology 45:1040–1049.

[R68] Parmesan C. 2006. Ecological and Evolutionary Responses to Recent Climate Change. Annual Review of Ecology, Evolution, and Systematics 37:637–669.

[R69] Patsiou T. S., Shestakova T. A., Klein T., di Matteo G., Sbay H., Chambel M. R., Zas R., and Voltas J.. 2020. Intraspecific responses to climate reveal nonintuitive warming impacts on a widespread thermophilic conifer. New Phytologist 228:525–540. 32402106 10.1111/nph.16656

[R70] Porth I., and El-Kassaby Y. A.. 2015. Using Populus as a lignocellulosic feedstock for bioethanol. Biotechnology Journal 10:510–524. 25676392 10.1002/biot.201400194

[R71] Putra A. R., Yen J. D. L., and Fournier-Level A.. 2023. Forecasting trait responses in novel environments to aid seed provenancing under climate change. Molecular Ecology Resources 23:565–580. 36308465 10.1111/1755-0998.13728

[R72] R Core Team. 2024. R: A Language and Environment for Statistical Computing. R Foundation for Statistical Computing, Vienna, Austria.

[R73] Rehfeldt G. E., Leites L. P., Joyce D. G., and Weiskittel A. R.. 2018. Role of population genetics in guiding ecological responses to climate. Global Change Biology 24:858–868. 28862811 10.1111/gcb.13883

[R74] Rogers A. R., and Holland J. B.. 2021. Environment-specific genomic prediction ability in maize using environmental covariates depends on environmental similarity to training data. G3 (Bethesda) 12:jkab440. 10.1093/g3journal/jkab440 PMC9245610 35100364

[R75] Sannigrahi P., Ragauskas A. J., and Tuskan G. A.. 2010. Poplar as a feedstock for biofuels: A review of compositional characteristics. Biofuels, Bioproducts and Biorefining 4:209–226.

[R76] Sharma S., Andrus R., Bergeron Y., Bogdziewicz M., Bragg D. C., Brockway D., Cleavitt N. L., Courbaud B., Das A. J., Dietze M., Fahey T. J., Franklin J. F., Gilbert G. S., Greenberg C. H., Guo Q., Hille Ris Lambers J., Ibanez I., Johnstone J. F., Kilner C. L., Knops J. M. H., Koenig W. D., Kunstler G., LaMontagne J. M., Macias D., Moran E., Myers J. A., Parmenter R., Pearse I. S., Poulton-Kamakura R., Redmond M. D., Reid C. D., Rodman K. C., Scher C. L., Schlesinger W. H., Steele M. A., Stephenson N. L., Swenson J. J., Swift M., Veblen T. T., Whipple A. V., Whitham T. G., Wion A. P., Woodall C. W., Zlotin R., and Clark J. S.. 2022. North American tree migration paced by climate in the West, lagging in the East. Proceedings of the National Academy of Sciences 119:e2116691118. Proceedings of the National Academy of Sciences. 10.1073/pnas.2116691118 PMC8784119 34983867

[R77] Slavov G. T., DiFazio S. P., Martin J., Schackwitz W., Muchero W., Rodgers-Melnick E., Lipphardt M. F., Pennacchio C. P., Hellsten U., Pennacchio L. A., Gunter L. E., Ranjan P., Vining K., Pomraning K. R., Wilhelm L. J., Pellegrini M., Mockler T. C., Freitag M., Geraldes A., El-Kassaby Y. A., Mansfield S. D., Cronk Q. C. B., Douglas C. J., Strauss S. H., Rokhsar D., and Tuskan G. A.. 2012. Genome resequencing reveals multiscale geographic structure and extensive linkage disequilibrium in the forest tree Populus trichocarpa. New Phytologist 196:713–725. 22861491 10.1111/j.1469-8137.2012.04258.x

[R78] Suarez-Gonzalez A., Hefer C. A., Christe C., Corea O., Lexer C., Cronk Q. C. B., and Douglas C. J.. 2016. Genomic and functional approaches reveal a case of adaptive introgression from Populus balsamifera (balsam poplar) in P. trichocarpa (black cottonwood). Molecular Ecology 25:2427–2442. 26825293 10.1111/mec.13539

[R79] Suarez-Gonzalez A., Hefer C. A., Lexer C., Cronk Q. C. B., and Douglas C. J.. 2018a. Scale and direction of adaptive introgression between black cottonwood (Populus trichocarpa) and balsam poplar (P. balsamifera). Molecular Ecology 27:1667–1680. 29575353 10.1111/mec.14561

[R80] Suarez-Gonzalez A., Lexer C., and Cronk Q. C. B.. 2018b. Adaptive introgression: a plant perspective. Biology Letters 14:20170688. Royal Society. 29540564 10.1098/rsbl.2017.0688 PMC5897607

[R81] Taylor S. A., Larson E. L., and Harrison R. G.. 2015. Hybrid zones: windows on climate change. Trends in Ecology & Evolution 30:398–406. Elsevier. 25982153 10.1016/j.tree.2015.04.010 PMC4794265

[R82] Taylor S. A., White T. A., Hochachka W. M., Ferretti V., Curry R. L., and Lovette I.. 2014. Climate-Mediated Movement of an Avian Hybrid Zone. Current Biology 24:671–676. 24613306 10.1016/j.cub.2014.01.069

[R83] Van Nuland M. E., Vincent J. B., Ware I. M., Mueller L. O., Bayliss S. L. J., Beals K. K., Schweitzer J. A., and Bailey J. K.. 2020. Intraspecific trait variation across elevation predicts a widespread tree species’ climate niche and range limits. Ecology and Evolution 10:3856–3867. 32489616 10.1002/ece3.5969 PMC7244802

[R84] VanWallendael A., Lowry D. B., and Hamilton J. A.. 2022. One hundred years into the study of ecotypes, new advances are being made through large-scale field experiments in perennial plant systems. Current Opinion in Plant Biology 66:102152. 35065527 10.1016/j.pbi.2021.102152

[R85] Via S., Gomulkiewicz R., Jong G. D., Scheiner S. M., Schlichting C. D., and Tienderen P. H. V.. 1995. Adaptive phenotypic plasticity: consensus and controversy. Trends in Ecology & Evolution 10:212–217. Elsevier. 21237012 10.1016/s0169-5347(00)89061-8

[R86] Wang T., Hamann A., Spittlehouse D., and Carroll C.. 2016. Locally downscaled and spatially customizable climate data for historical and future periods for North America. PLOS ONE 11:e0156720. 27275583 10.1371/journal.pone.0156720 PMC4898765

[R87] Wang T., Hamann A., Yanchuk A., O’neill G. A., and Aitken S. N.. 2006. Use of response functions in selecting lodgepole pine populations for future climates. Global Change Biology 12:2404–2416.

[R88] Wang T., O’Neill G. A., and Aitken S. N.. 2010. Integrating environmental and genetic effects to predict responses of tree populations to climate. Ecological Applications 20:153–163. 20349837 10.1890/08-2257.1

[R89] Wielstra B. 2019. Historical hybrid zone movement: More pervasive than appreciated. Journal of Biogeography 46:1300–1305.

[R90] Woolbright S. A., Whitham T. G., Gehring C. A., Allan G. J., and Bailey J. K.. 2014. Climate relicts and their associated communities as natural ecology and evolution laboratories. Trends in Ecology & Evolution 29:406–416. Elsevier. 24932850 10.1016/j.tree.2014.05.003

[R91] Yang N., Wang Y., Liu X., Jin M., Vallebueno-Estrada M., Calfee E., Chen L., Dilkes B. P., Gui S., Fan X., Harper T. K., Kennett D. J., Li W., Lu Y., Ding J., Chen Z., Luo J., Mambakkam S., Menon M., Snodgrass S., Veller C., Wu S., Wu S., Zhuo L., Xiao Y., Yang X., Stitzer M. C., Runcie D., Yan J., and Ross-Ibarra J.. 2023. Two teosintes made modern maize. Science 382:eadg8940. American Association for the Advancement of Science. 38033071 10.1126/science.adg8940

[R92] Ye Z., O’Neill G. A., and Wang T.. 2023. Optimization and validation of universal response functions for interior spruce (Picea glauca, Picea engelmannii, and their hybrids). Forest Ecology and Management 550:121509.

[R93] Yeaman S., Hodgins K. A., Lotterhos K. E., Suren H., Nadeau S., Degner J. C., Nurkowski K. A., Smets P., Wang T., Gray L. K., Liepe K. J., Hamann A., Holliday J. A., Whitlock M. C., Rieseberg L. H., and Aitken S. N.. 2016. Convergent local adaptation to climate in distantly related conifers. Science 353:1431–1433. 27708038 10.1126/science.aaf7812

[R94] Zhang M., Suren H., and Holliday J. A.. 2019. Phenotypic and Genomic Local Adaptation across Latitude and Altitude in Populus trichocarpa. Genome Biology and Evolution 11:2256–2272. 31298685 10.1093/gbe/evz151 PMC6735766

[R95] Zhou L., Bawa R., and Holliday J. A.. 2014. Exome resequencing reveals signatures of demographic and adaptive processes across the genome and range of black cottonwood (Populus trichocarpa). Molecular Ecology 23:2486–2499. 24750333 10.1111/mec.12752

