# Supplementary material for: Variation in responses to temperature across admixed genotypes of Populus trichocarpa × P. balsamifera predict geographic shifts in regions where hybrids are favored
Source: bioRxiv. 2025 May 22:2025.05.16.654548. Preprint. [Version 1] doi: 10.1101/2025.05.16.654548 (PMC12139819; doi:10.1101/2025.05.16.654548)
Supplement: Supplement 1 [file media-1.pdf]

Supplemental Material for "Variation in responses to temperature across admixed genotypes of *Populus trichocarpa* × *P. balsamifera* predict geographic shifts in regions where hybrids are favored"

Supplementary Methods

Genomic data

For each genotype, approximately 100 mg of young leaf tissue was used for genomic DNA extraction with the Qiagen Plant DNeasy kit. Genomic libraries were sequenced using an Illumina NovaSeq 6000, with 64 samples per lane, using paired-end 150 bp reads. Reads were aligned to the *P. trichocarpa* reference genome (v4.0) and variant calling was performed using GATK Haplotype Caller. Within GATK, variants were filtered for quality-by-depth (QD<2), mapping quality (MQ<40), elevated strand bias (FS>40, SOR>3), and differential map quality and positional bias between reference and alternate alleles (MQRankSum<-12.5, ReadPosRankSum<-8). Using bcftools, variants were subset to biallelic SNPs (-m2 -M2 snps) and variants with a minor allele count of 1 (--include 'MAC>1'). VCFtools was used to remove all SNPs with missing data across individuals (max-missing 1.0). Finally, variants were LD-pruned in PLINK using a 10000 bp window size, shifted by 1000 bp, removing variants with a pairwise R^2 value >0.1. After filtering and LD-pruning, a total of 334,657 variable sites were used to characterize genetic variation and admixture.

Supplementary Figures

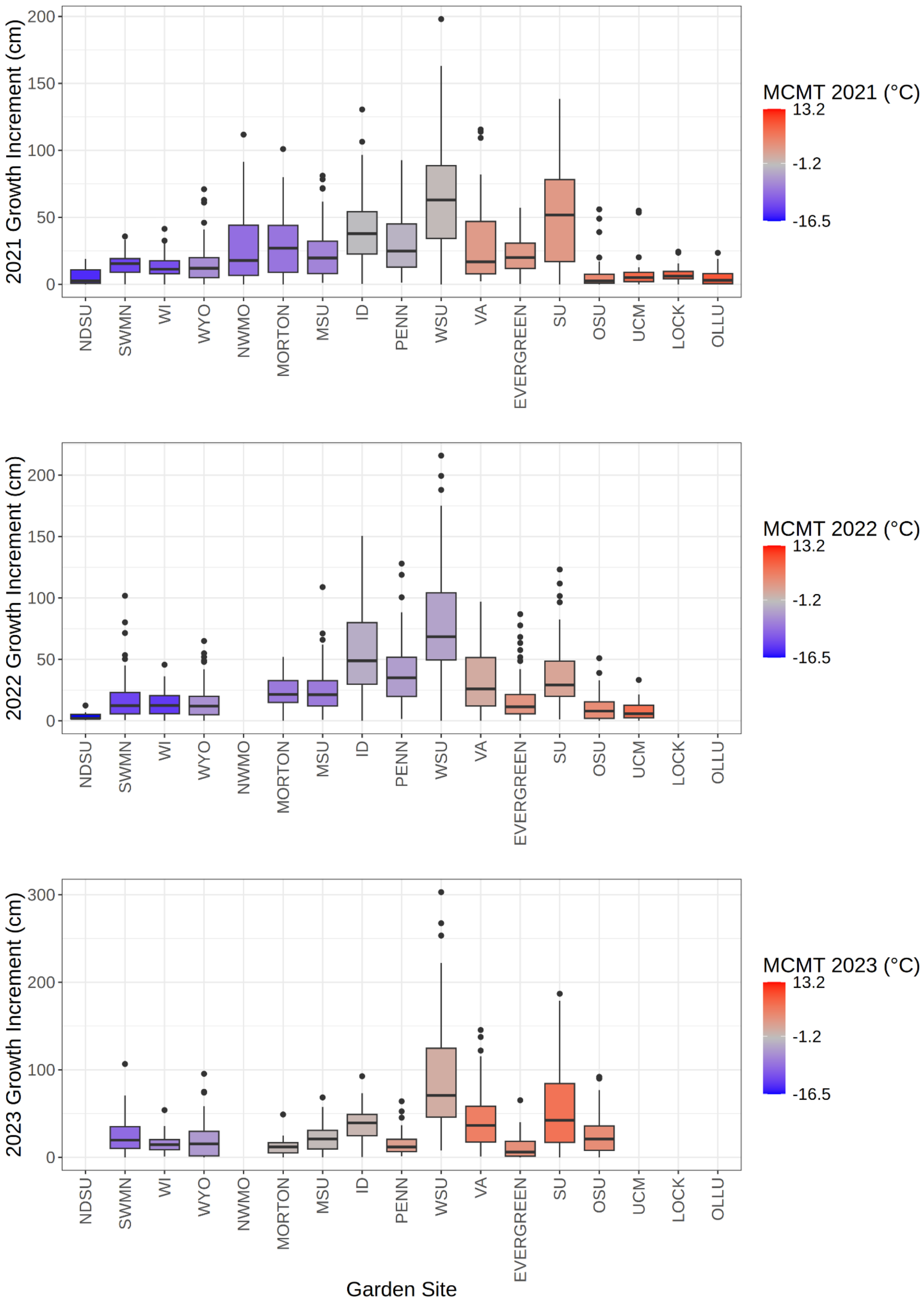
2021 Growth Increment (cm)
MCMT 2021 (°C)
13.2
-1.2
-16.5
2022 Growth Increment (cm)
MCMT 2022 (°C)
13.2
-1.2
-16.5
2023 Growth Increment (cm)
MCMT 2023 (°C)
13.2
-1.2
-16.5
NDSU
SWMN
WI
WYO
NWMO
MORTON
MSU
ID
PENN
WSU
VA
EVERGREEN
SU
OSU
UCM
LOCK
OLLU
Garden Site

Figure S1. Variation in growth increment across gardens for each year. Gardens are ordered along the x-axis by their mean coldest month temperature (MCMT) averaged from 2020-2023. Garden boxplots are colored by the MCMT (°C) of that year.

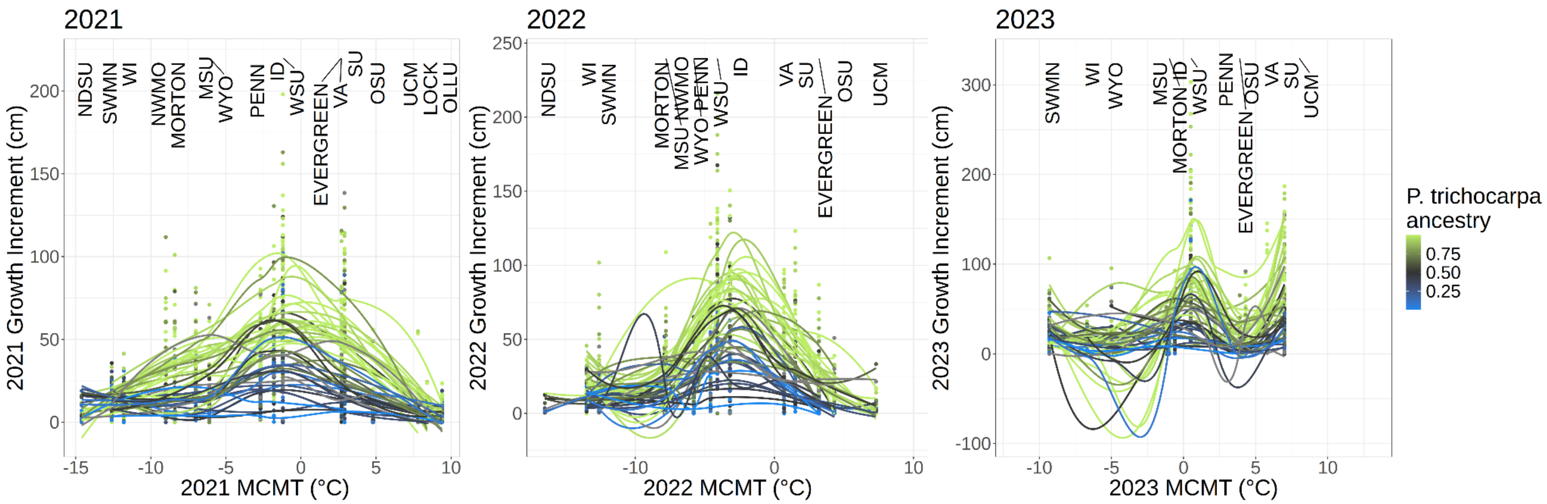

Figure S2. The genotype-specific response of yearly growth increment to garden MCMT across three years (2021-2023). Lines show the response of each genotype and are fitted for each using the loess function in R; colors indicate species ancestry at K=2. Text labels indicate the MCMT value of each garden for that year.

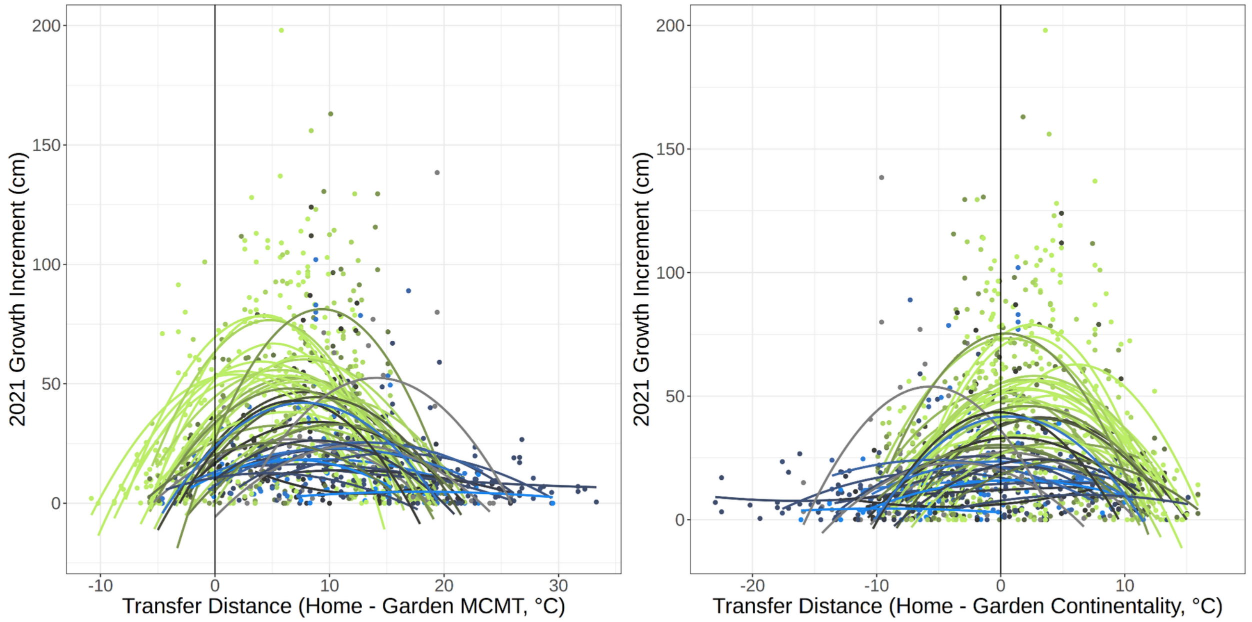

Figure S3. Response of growth increment to transfer distance, or the difference between home and common garden climate in 2021. Lines show the response of each genotype and are fitted using a simple quadratic model in the lm function in R. Line colors indicate the genotype's species ancestry at K=2. The response to continentality transfer distance indicates genotypes generally have the highest growth in environments similar to their climate of origin (transfer distance = 0) while the response to mean coldest month temperature transfer distance indicates genotypes have higher growth in environments that are warmer than their climate of origin.

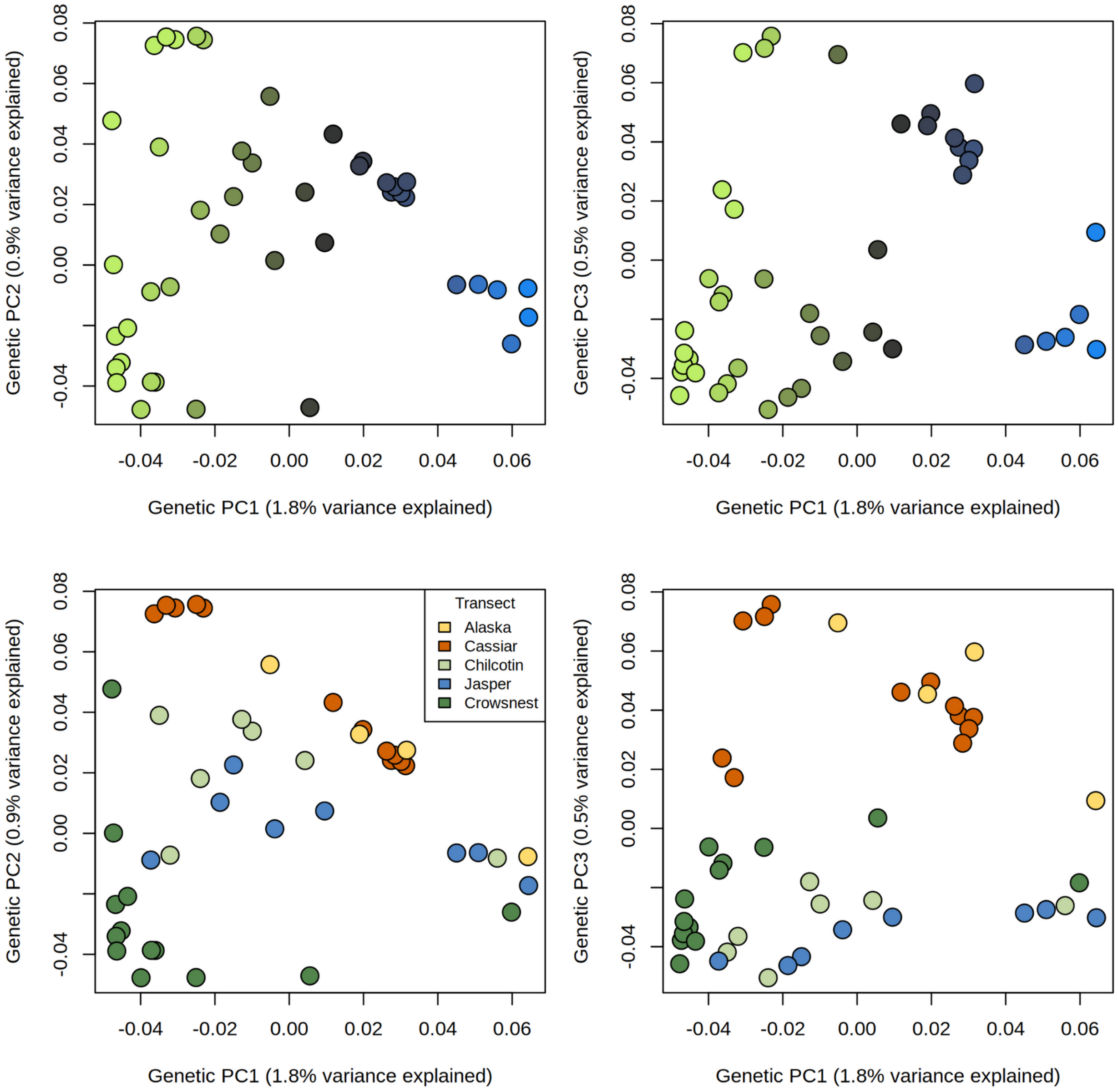

Figure S4. Genetic PCs 1-3 for each genotype, used to estimate the effect of genetic structure on phenotypic responses to climate. Top: colors represent species ancestry at K=2. Bottom: colors represent transect, listed from northernmost (Alaska) to southernmost (Crowsnest).

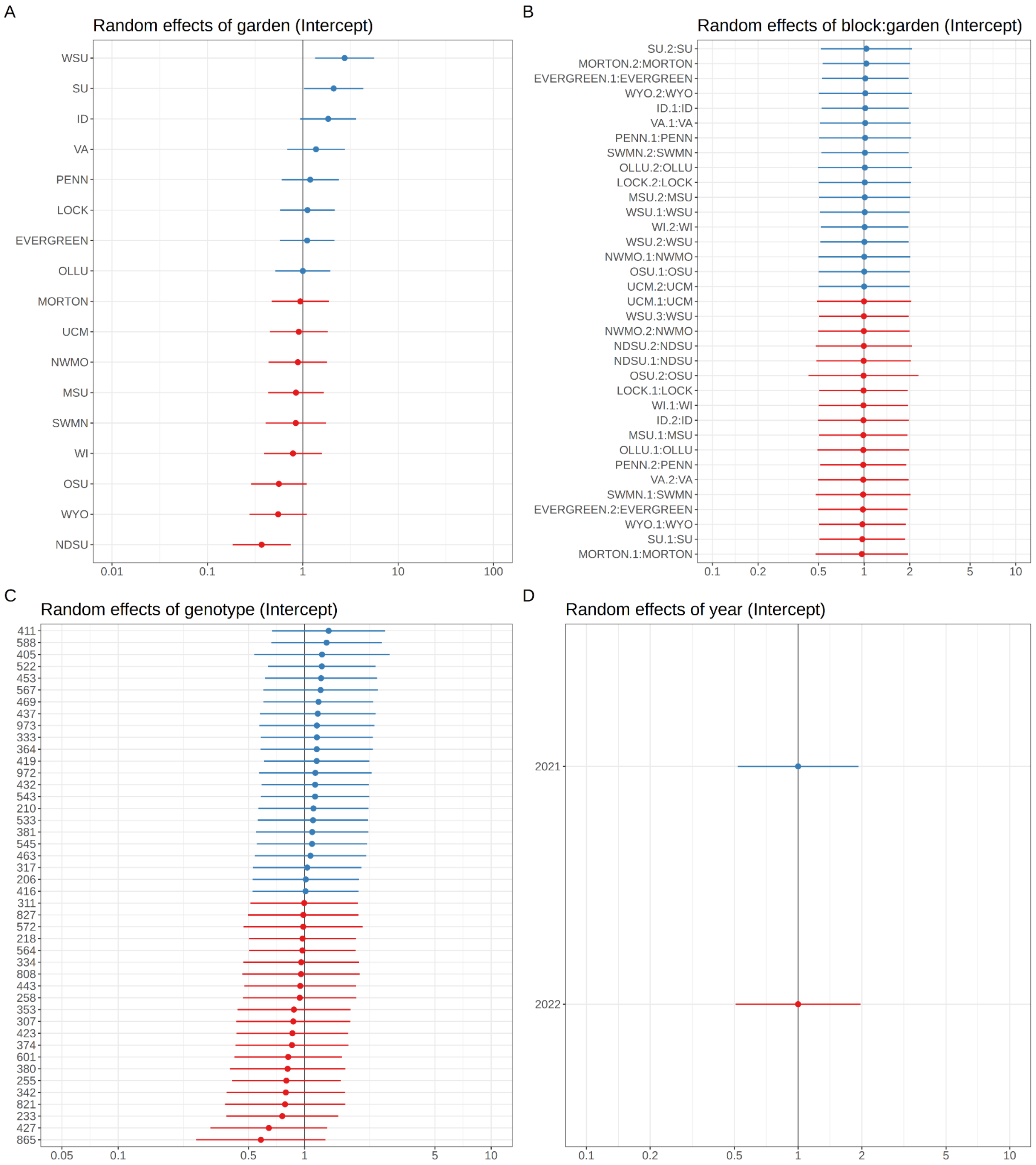

Figure S5. Estimates of model random effects for A) garden site, B) block nested within garden, C) genotype, and D) year.

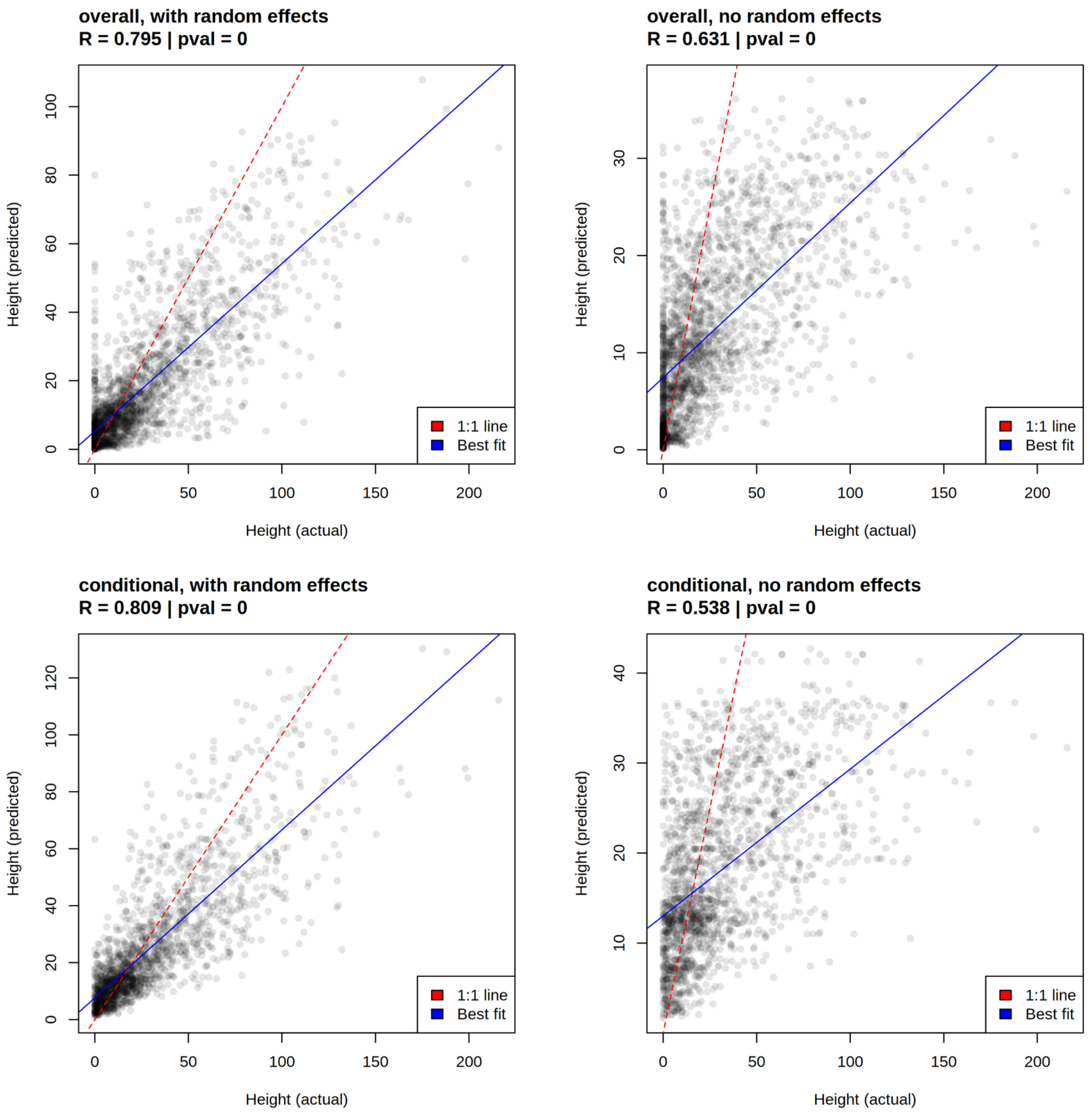

Figure S6. Correlation between actual and predicted growth for individuals across all 17 garden sites and two years, with the Pearson correlation and its p-value, across four categories of predictions from the model. Results are shown for heights predicted from the overall model, including growth and mortality, with dead individuals having a height of zero (top) and for the conditional model representing growth in the surviving individuals (bottom). When the random effects of genotype, garden, and block are included (left) predictions are more closely correlated with actual heights compared to when they were excluded (right). Best fit lines have a lower slope than the 1:1 line, indicating that the model underpredicts height for taller individuals.

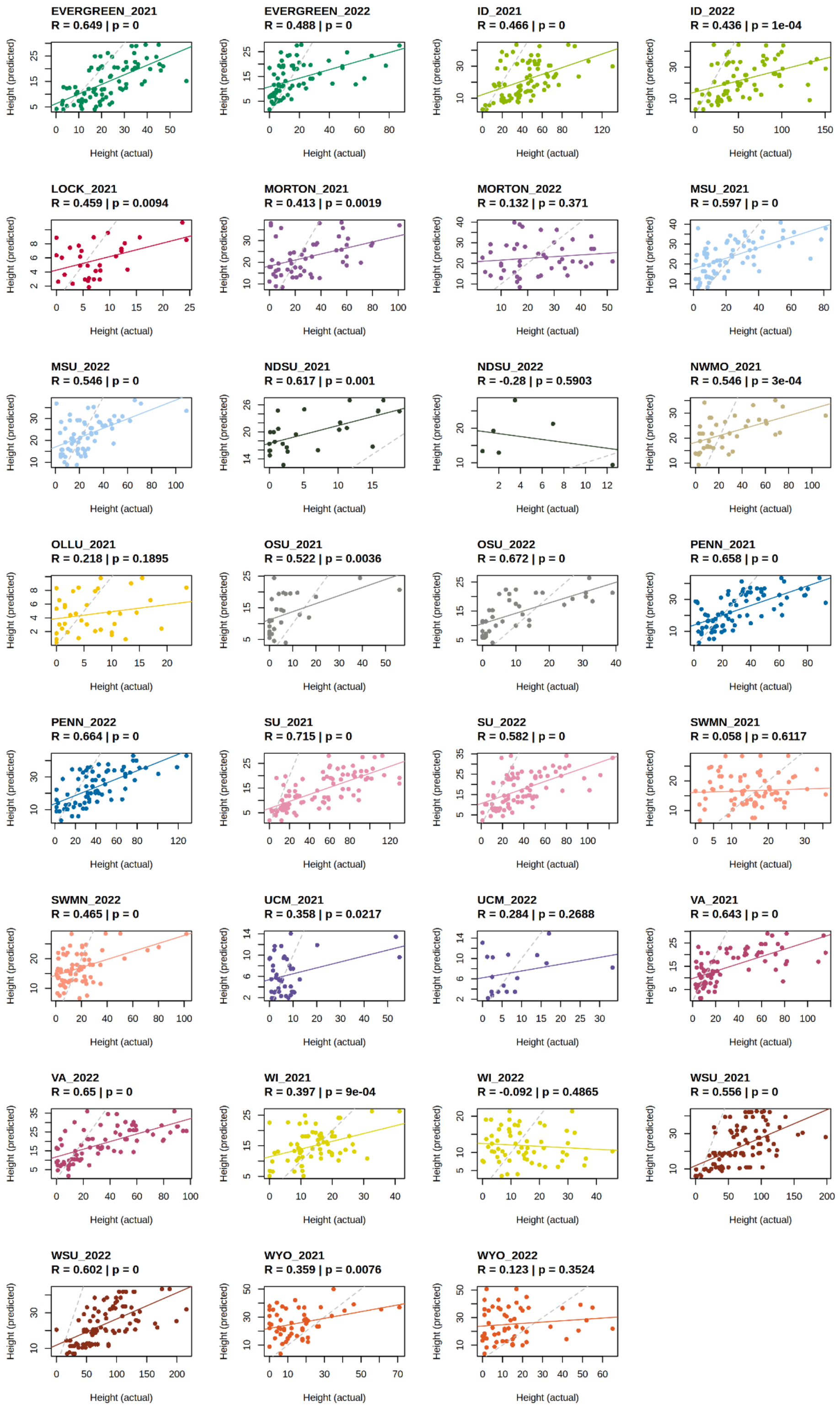
EVERGREEN_2021
R = 0.649 | p = 0
EVERGREEN_2022
R = 0.488 | p = 0
ID_2021
R = 0.466 | p = 0
ID_2022
R = 0.436 | p = 1e-04
LOCK_2021
R = 0.459 | p = 0.0094
MORTON_2021
R = 0.413 | p = 0.0019
MORTON_2022
R = 0.132 | p = 0.371
MSU_2021
R = 0.597 | p = 0
MSU_2022
R = 0.546 | p = 0
NDSU_2021
R = 0.617 | p = 0.001
NDSU_2022
R = -0.28 | p = 0.5903
NWMO_2021
R = 0.546 | p = 3e-04
OLLU_2021
R = 0.218 | p = 0.1895
OSU_2021
R = 0.522 | p = 0.0036
OSU_2022
R = 0.672 | p = 0
PENN_2021
R = 0.658 | p = 0
PENN_2022
R = 0.664 | p = 0
SU_2021
R = 0.715 | p = 0
SU_2022
R = 0.582 | p = 0
SWMN_2021
R = 0.058 | p = 0.6117
SWMN_2022
R = 0.465 | p = 0
UCM_2021
R = 0.358 | p = 0.0217
UCM_2022
R = 0.284 | p = 0.2688
VA_2021
R = 0.643 | p = 0
VA_2022
R = 0.65 | p = 0
WI_2021
R = 0.397 | p = 9e-04
WI_2022
R = -0.092 | p = 0.4865
WSU_2021
R = 0.556 | p = 0
WSU_2022
R = 0.602 | p = 0
WYO_2021
R = 0.359 | p = 0.0076
WYO_2022
R = 0.123 | p = 0.3524
Height (predicted)
Height (actual)

Figure S7. Correlation between actual and predicted growth for each garden and year from leave-one-out cross validation predictions in gardens, in which predictions were made for the garden using a model trained on the other gardens. Pearson R values and p-values for the correlation are shown. Solid lines indicate the best fit line; grey dotted lines indicate the one-to-one line.

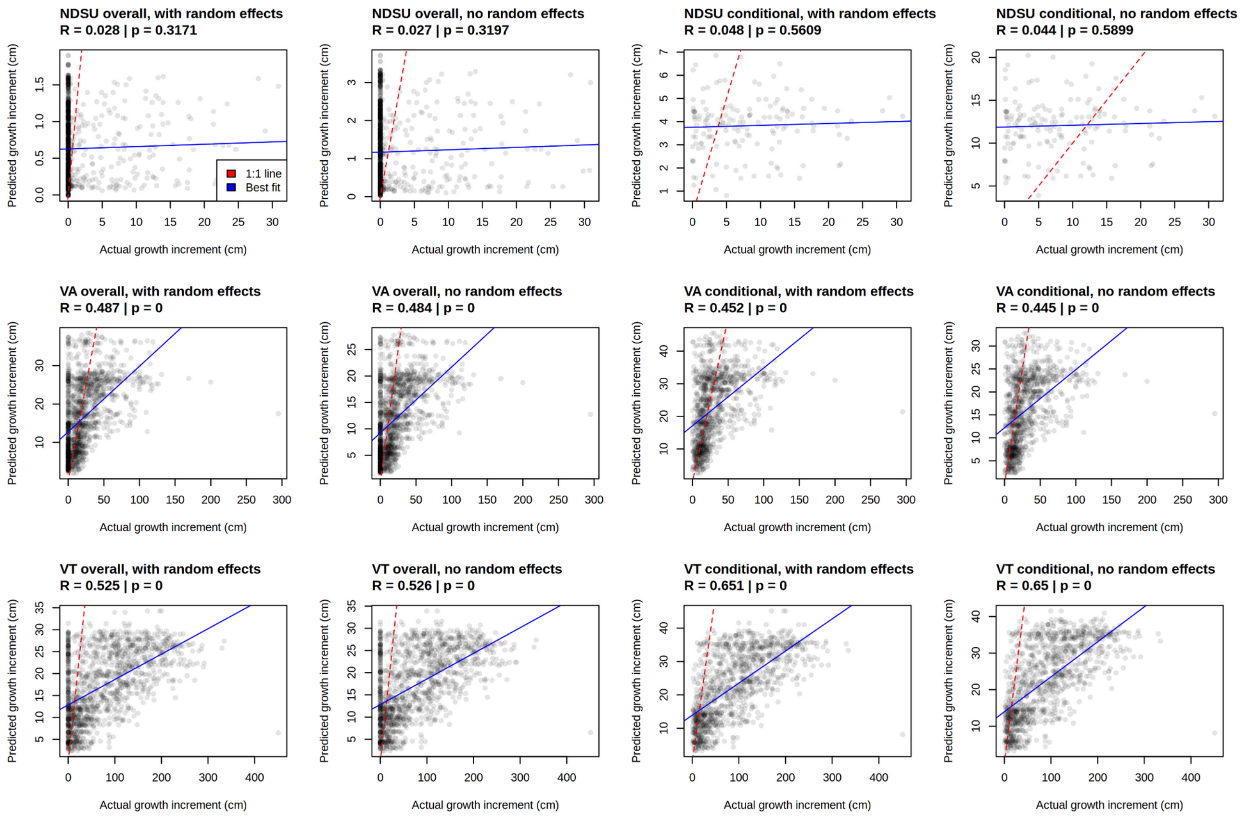

Figure S8. Correlation between actual and predicted growth for individuals across all 17 garden sites, with the Pearson correlation and its p-value, across four categories of predictions from the model (as described in Figure S6).

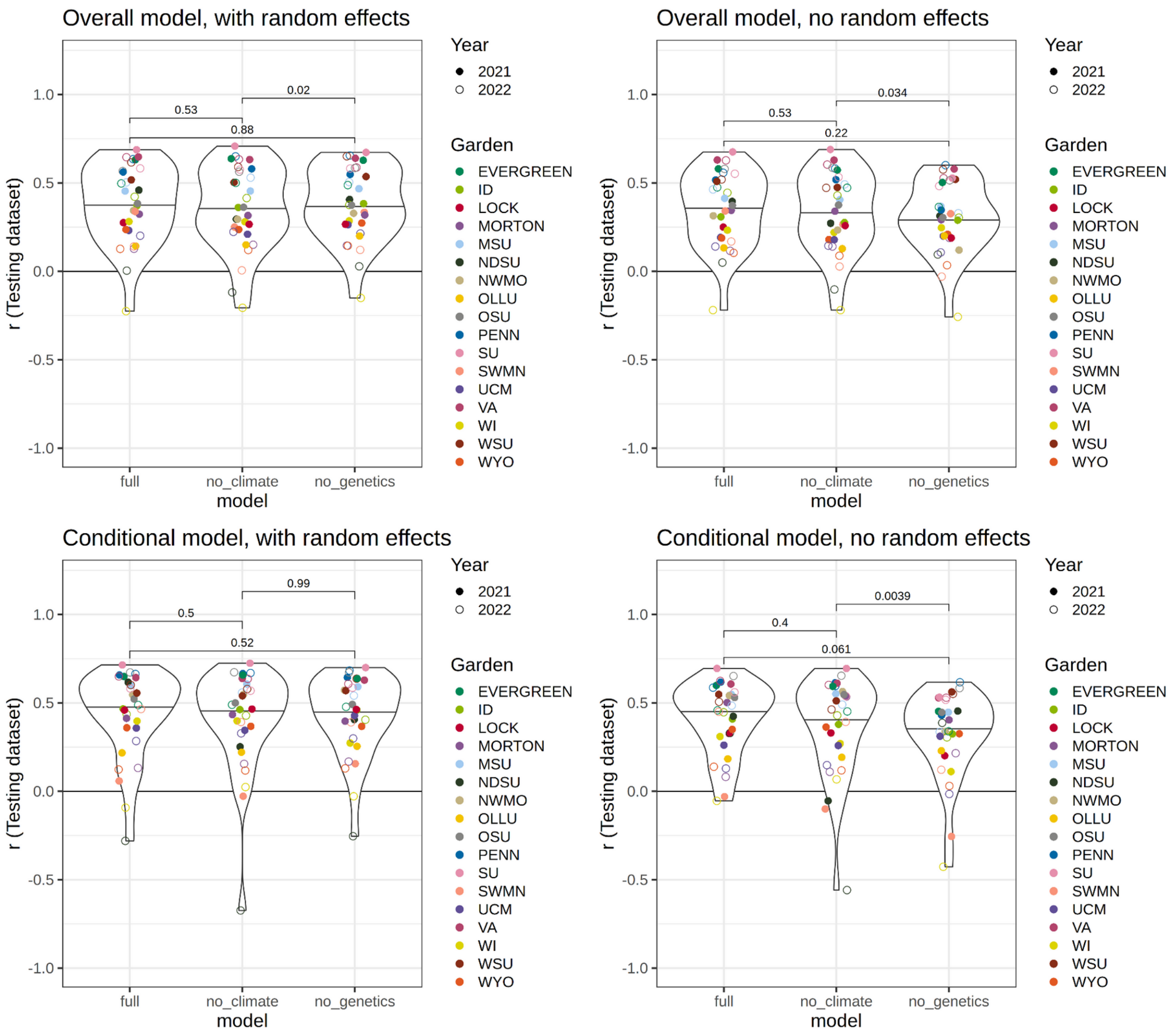

Figure S9. Comparisons of Pearson's correlation r values with and without genetic and home climate information. P-values are given for each pairwise model comparison using a paired Wilcoxon test.

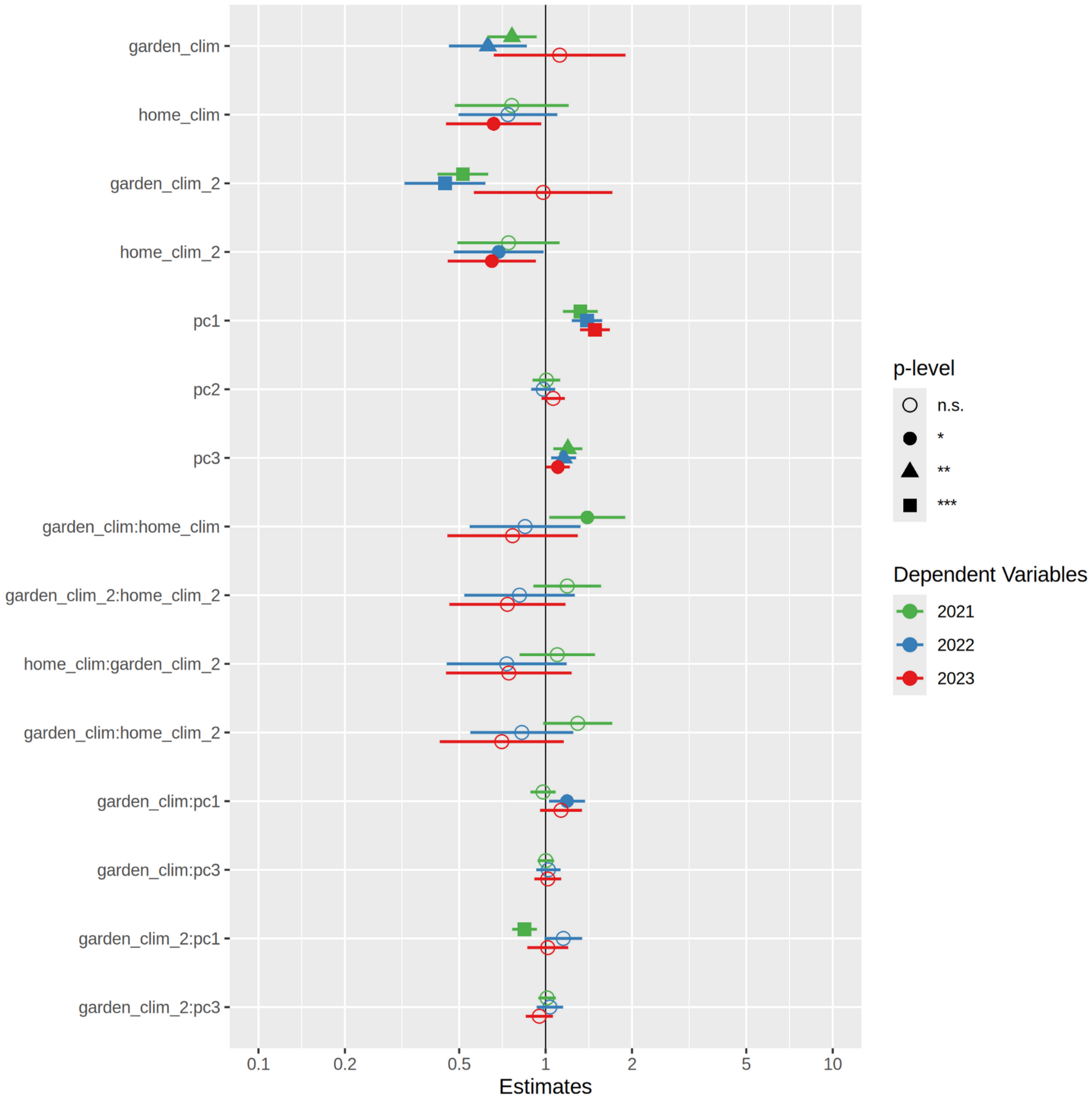

Figure S10. Comparison of model effects across three years of data collection. The model is similar to that shown in Figure 2; however, some interaction effects were dropped to allow convergence of the 2023 model, and the same model was used for 2021 and 2022 datasets for comparison. PCs indicate genetic PCs; home_clim and garden_clim indicate values of MCMT climate, "_2" indicates the square term for climate variables, and ":" indicates interaction effects.

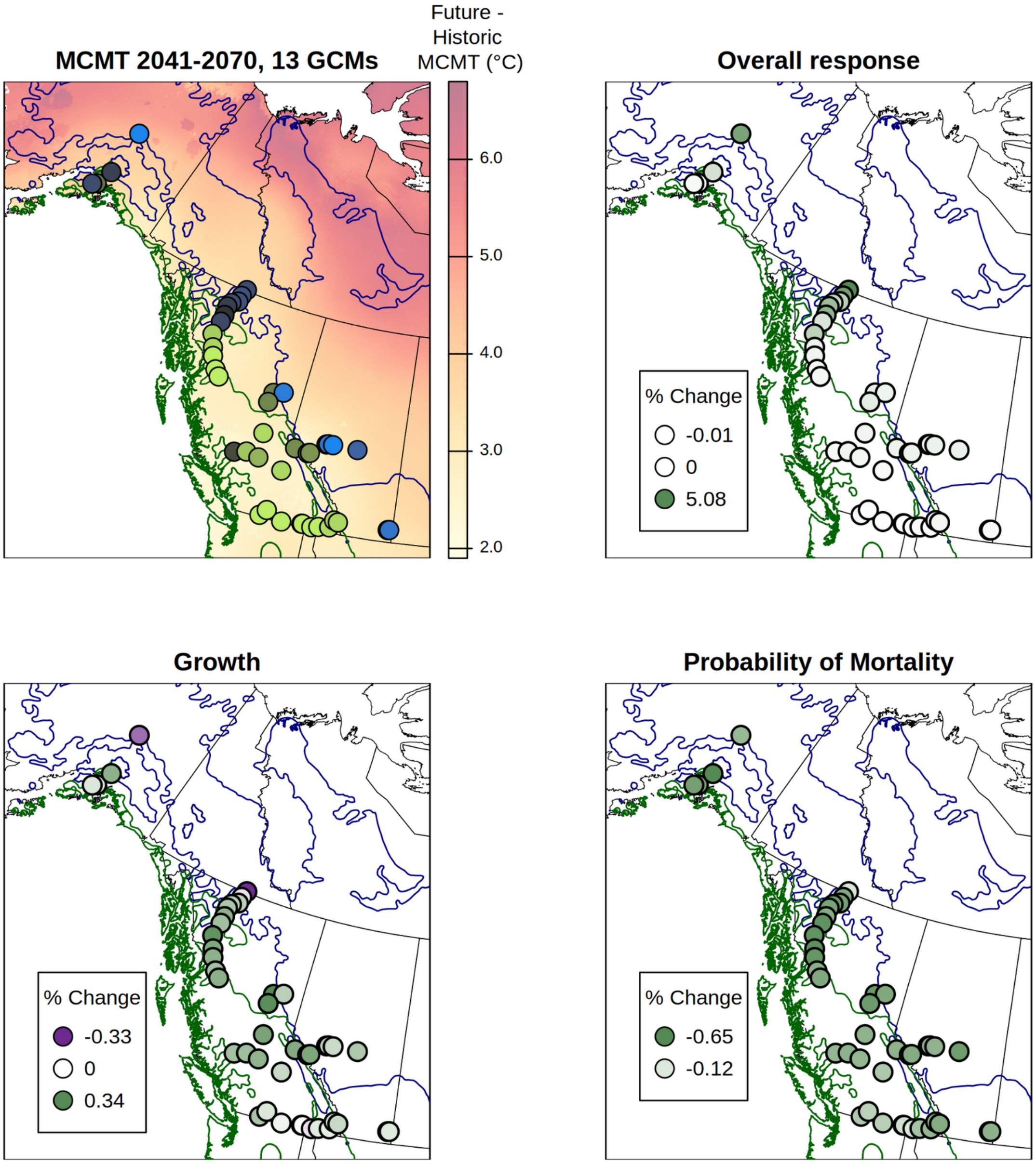

Figure S11. Spatial changes in MCMT and predicted changes in fitness metrics for each genotype as a result of changing climate at its home site, based on their norm of reaction (Figure 5). Green indicates increased fitness, purple indicates decreased fitness, and white indicates no change; minimum and maximum values for each metric are shown in the legend. Predictions are shown for changes in MCMT between the periods of 1961-1990 and 2041-2070 under SSP 2-45.

Figure S12 (attached as a separate file). Predicted reaction norms across values of MCMT as in Figure 5a, with each genotype plotted separately (solid curve) with its MCMT of origin (vertical dotted line), allowing performance to be compared among the "local" genotype (MCMT of origin is the same as MCMT at planting site) and all other genotypes. Under the "local vs foreign" criterion of local adaptation, the genotype originating from a particular environment should outperform other genotypes in that environment (Kawecki and Ebert 2004).

Supplementary Tables

Table S1. Effects of the linear mixed-effect model predicting yearly growth increment: home and garden MCMT, the square terms of MCMT, genetic PCs 1-3, and their interactions. Effects are shown for the conditional, or count, model testing each factor's effect on growth, and for the zero-inflated model testing the effect on the probability of mortality.

**Count Model**						
**Predictors**	**Estimates**	**std. Beta**	**CI**	**standardized CI**	**p**	**std. p**
(Intercept)	2.85	2.73	2.13 – 3.57	2.48 – 2.98	**<0.001**	**<0.001**
Garden MCMT	-0.28	-0.53	-0.83 – 0.28	-0.74 – -0.31	0.33	**<0.001**
Home MCMT	-0.42	-0.27	-1.57 – 0.72	-0.65 – 0.11	0.468	0.169
Garden MCMT^2	-0.83	-0.46	-1.53 – -0.13	-0.74 – -0.18	**0.02**	**0.001**
Home MCMT^2	-0.54	-0.33	-1.14 – 0.06	-0.68 – 0.01	0.077	0.06
Genetic PC1	-0.33	-0.3	-0.46 – -0.21	-0.41 – -0.18	**<0.001**	**<0.001**
Genetic PC2	0.05	-0.02	-0.06 – 0.16	-0.12 – 0.07	0.35	0.66
Genetic PC3	-0.18	-0.15	-0.29 – -0.08	-0.24 – -0.05	**0.001**	**0.003**
Garden MCMT × Home MCMT	0.64	0.21	-0.27 – 1.55	-0.09 – 0.51	0.168	0.168
Garden MCMT^2 × Home MCMT^2	0.23	0.11	-0.33 – 0.79	-0.16 – 0.37	0.427	0.427
Home MCMT × Garden MCMT^2	-0.1	-0.03	-1.19 – 0.98	-0.32 – 0.27	0.855	0.855
Garden MCMT × Home MCMT^2	0.38	0.21	-0.10 – 0.85	-0.06 – 0.49	0.121	0.121
Garden MCMT × Genetic PC1	-0.01	-0.01	-0.12 – 0.10	-0.11 – 0.09	0.86	0.86
Garden MCMT × Genetic PC2	0.01	0.01	-0.07 – 0.09	-0.06 – 0.08	0.796	0.796
Garden MCMT × Genetic PC3	-0.03	-0.03	-0.11 – 0.05	-0.10 – 0.05	0.487	0.487
Garden MCMT^2 × Genetic PC1	0.04	0.03	-0.08 – 0.16	-0.06 – 0.12	0.521	0.521
Garden MCMT^2 × Genetic PC2	-0.1	-0.07	-0.20 – 0.00	-0.15 – 0.00	0.052	0.052
Garden MCMT^2 × Genetic PC3	0.04	0.03	-0.07 – 0.14	-0.05 – 0.10	0.487	0.487
(Intercept)	0.69	0.69	0.65 – 0.73	0.65 – 0.73		
**Zero-Inflated Model**						
**Predictors**	**Estimates**	**std. Beta**	**CI**	**standardized CI**	**p**	**std. p**
(Intercept)	-2.99	-2.03	-5.18 – -0.80	-2.81 – -1.26	**0.008**	**<0.001**
Garden MCMT	0.05	0.74	-1.31 – 1.42	0.10 – 1.37	0.94	**0.023**
Home MCMT	-0.07	0.24	-3.61 – 3.47	-0.74 – 1.21	0.97	0.636

Garden MCMT^2	2.08	1.34	0.11 – 4.05	0.72 – 1.95	**0.039**	**<0.001**
Home MCMT^2	0.15	0.2	-1.68 – 1.97	-0.69 – 1.08	0.876	0.663
Genetic PC1	0.14	0.08	-0.24 – 0.51	-0.21 – 0.36	0.475	0.604
Genetic PC2	-0.21	-0.17	-0.54 – 0.12	-0.42 – 0.07	0.217	0.163
Genetic PC3	-0.11	0.03	-0.44 – 0.22	-0.22 – 0.28	0.506	0.803
Garden MCMT × Home MCMT	-1.54	-0.51	-3.69 – 0.62	-1.22 – 0.21	0.164	0.164
Garden MCMT^2 × Home MCMT^2	-0.27	-0.13	-1.89 – 1.35	-0.89 – 0.64	0.741	0.741
Home MCMT × Garden MCMT^2	0.15	0.04	-3.02 – 3.33	-0.82 – 0.91	0.925	0.925
Garden MCMT × Home MCMT^2	-0.88	-0.5	-2.04 – 0.28	-1.16 – 0.16	0.136	0.136
Garden MCMT × Genetic PC1	0.04	0.03	-0.20 – 0.28	-0.18 – 0.25	0.756	0.756
Garden MCMT × Genetic PC2	0.04	0.03	-0.16 – 0.24	-0.14 – 0.21	0.71	0.71
Garden MCMT × Genetic PC3	-0.02	-0.02	-0.22 – 0.18	-0.20 – 0.16	0.843	0.843
Garden MCMT^2 × Genetic PC1	-0.06	-0.05	-0.39 – 0.27	-0.30 – 0.20	0.711	0.711
Garden MCMT^2 × Genetic PC2	0.07	0.05	-0.24 – 0.37	-0.17 – 0.27	0.671	0.671
Garden MCMT^2 × Genetic PC3	0.2	0.15	-0.10 – 0.50	-0.08 – 0.38	0.192	0.192

Table S2. Effect sizes of the random effects of genotype, garden, block nested within garden, year, and individual and their sample sizes.

**Random Effects**	
σ2	0.69
τ00 genotype	0.05
τ00 block:Garden	0
τ00 Garden	0.24
τ00 year	0
τ00 indiv	0.06
N genotype	44
N block	35
N Garden	17
N year	2
N indiv	1448
Observations	2308
Marginal R2 / Conditional R2	0.348 / NA
AIC	Inf
