# Supplementary material for: Variation in responses to temperature across admixed genotypes of Populus trichocarpa × P. balsamifera predict geographic shifts in regions where hybrids are favored
Source: bioRxiv. 2025 May 22:2025.05.16.654548. Preprint. [Version 1] doi: 10.1101/2025.05.16.654548 (PMC12139819; doi:10.1101/2025.05.16.654548)
Supplement: Supplement 2 [file media-2.pdf]

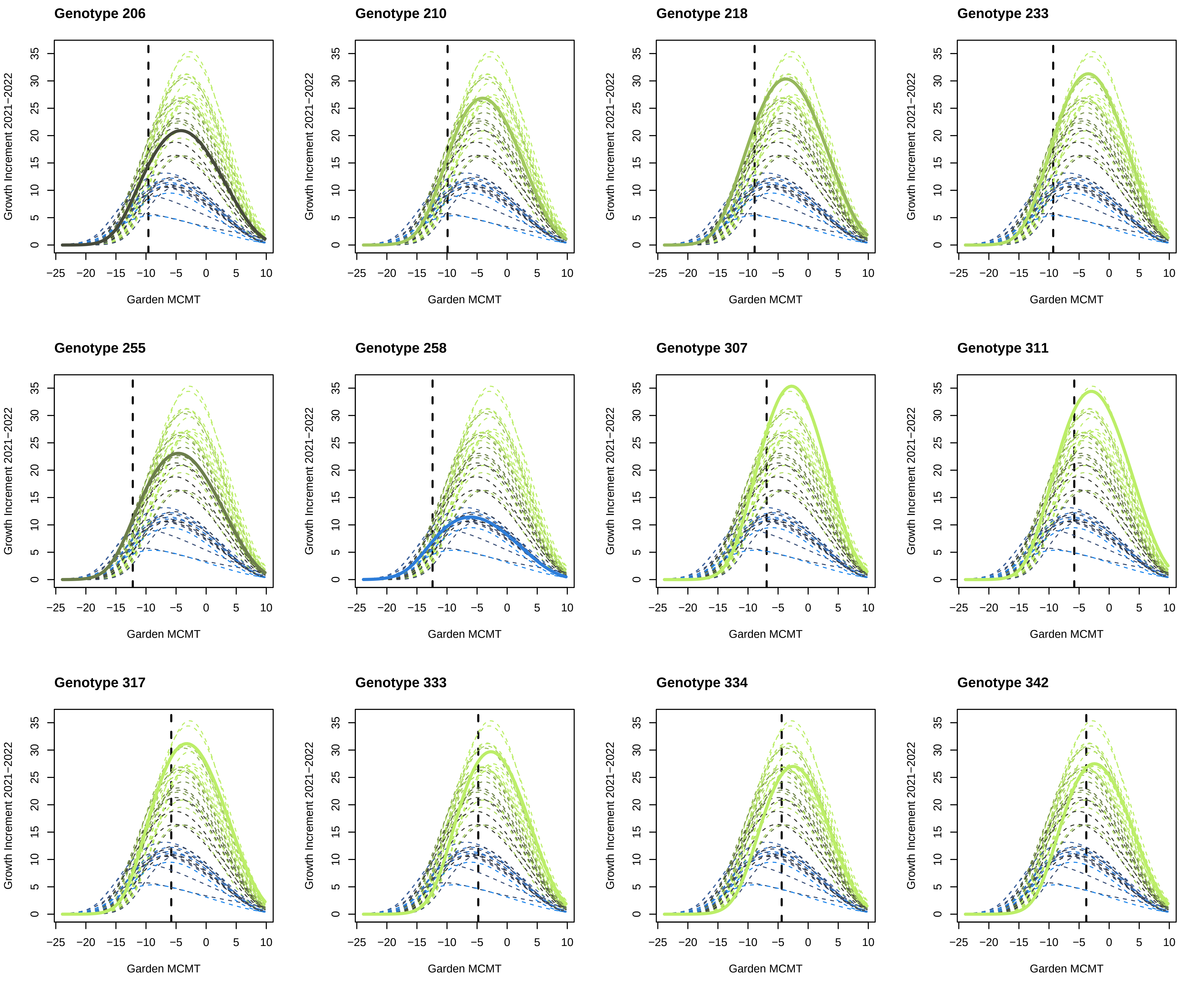
Genotype 206
Genotype 210
Genotype 218
Genotype 233
Genotype 255
Genotype 258
Genotype 307
Genotype 311
Genotype 317
Genotype 333
Genotype 334
Genotype 342
Growth Increment 2021−2022
Garden MCMT
0
5
10
15
20
25
30
35
−25
−20
−15
−10
−5
0
5
10

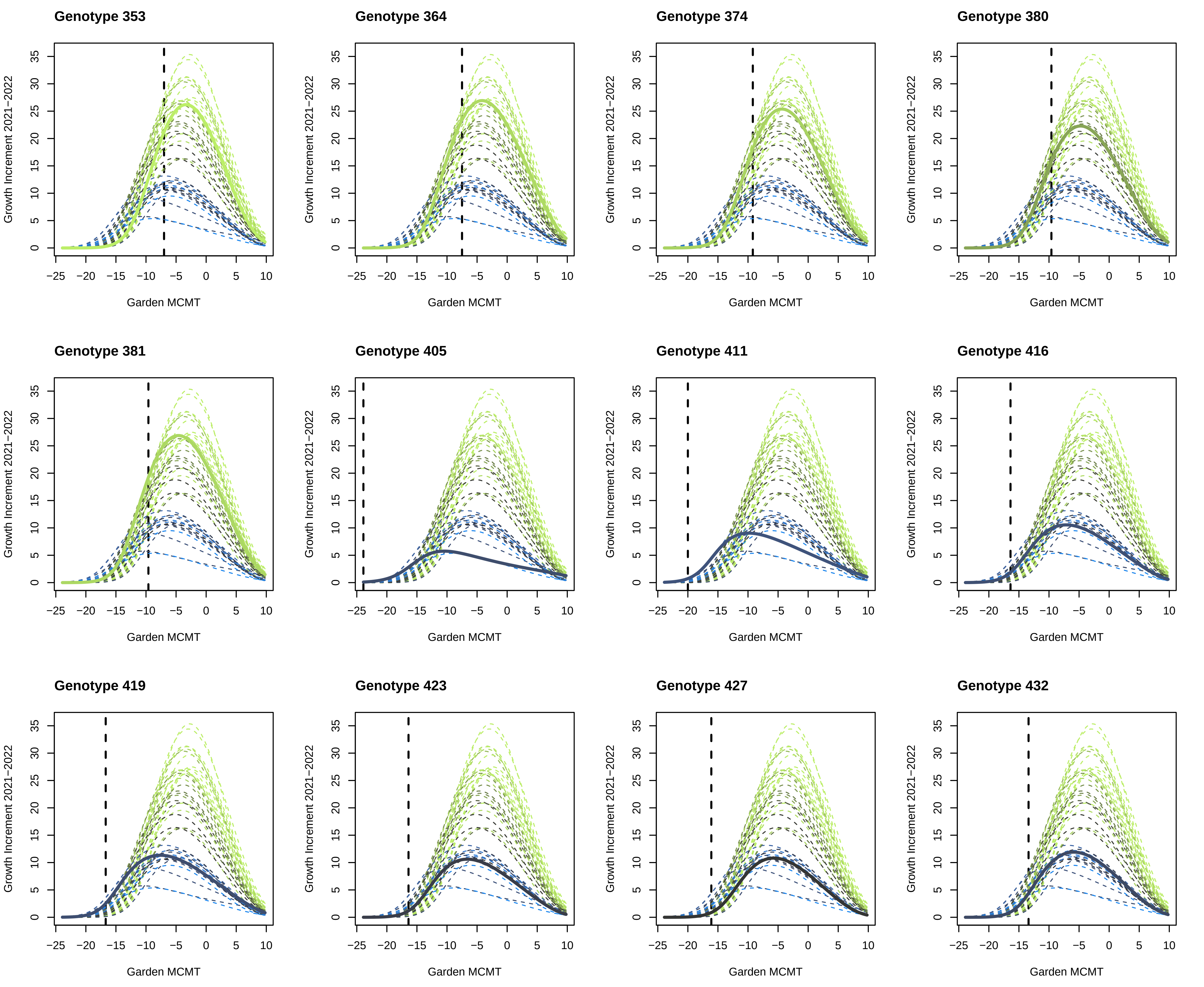
Genotype 353
Genotype 364
Genotype 374
Genotype 380
Genotype 381
Genotype 405
Genotype 411
Genotype 416
Genotype 419
Genotype 423
Genotype 427
Genotype 432
Growth Increment 2021−2022
Garden MCMT
0
5
10
15
20
25
30
35
−25
−20
−15
−10
−5
0
5
10

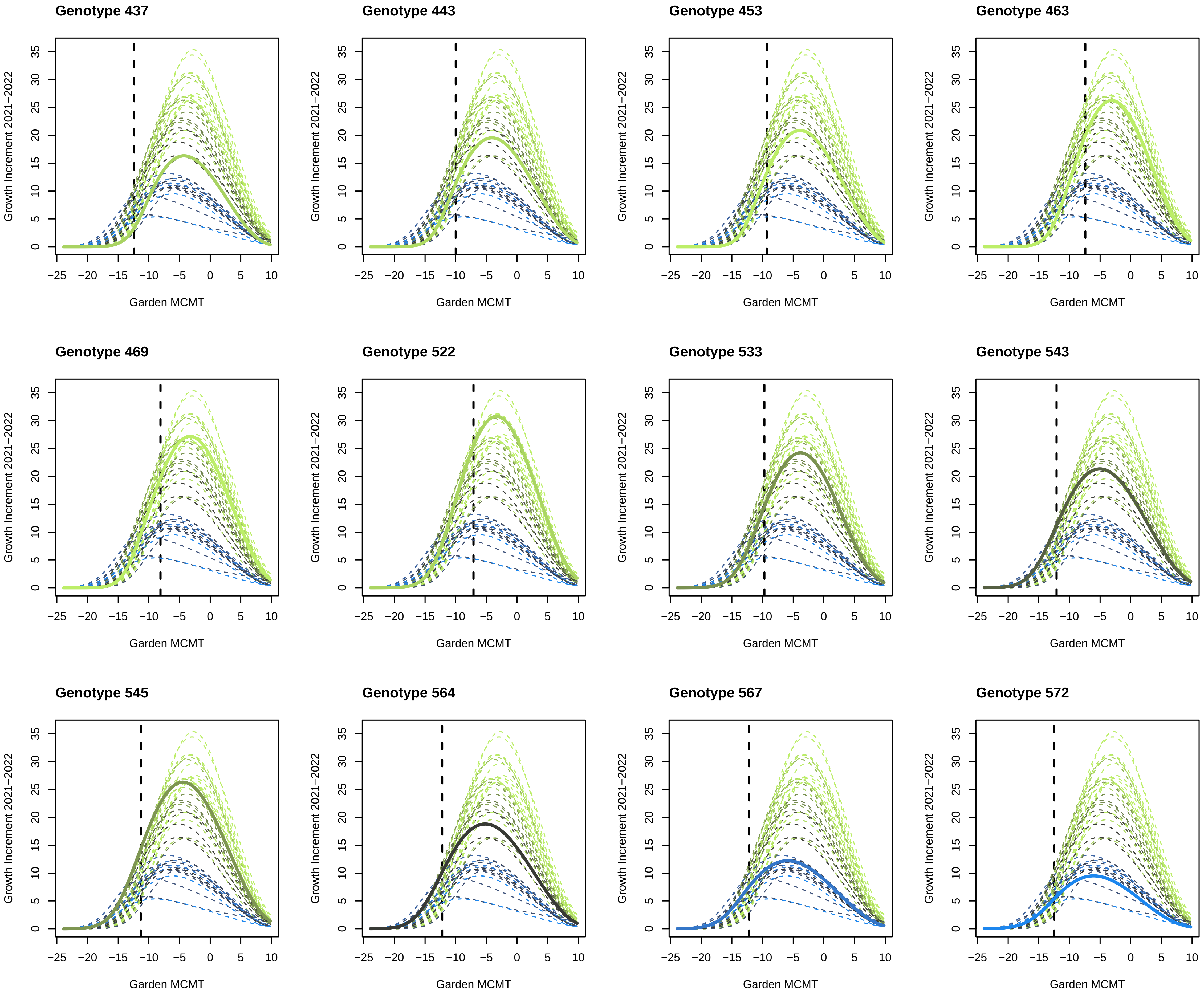

Genotype 437
Genotype 443
Genotype 453
Genotype 463
Genotype 469
Genotype 522
Genotype 533
Genotype 543
Genotype 545
Genotype 564
Genotype 567
Genotype 572
Growth Increment 2021−2022
Garden MCMT

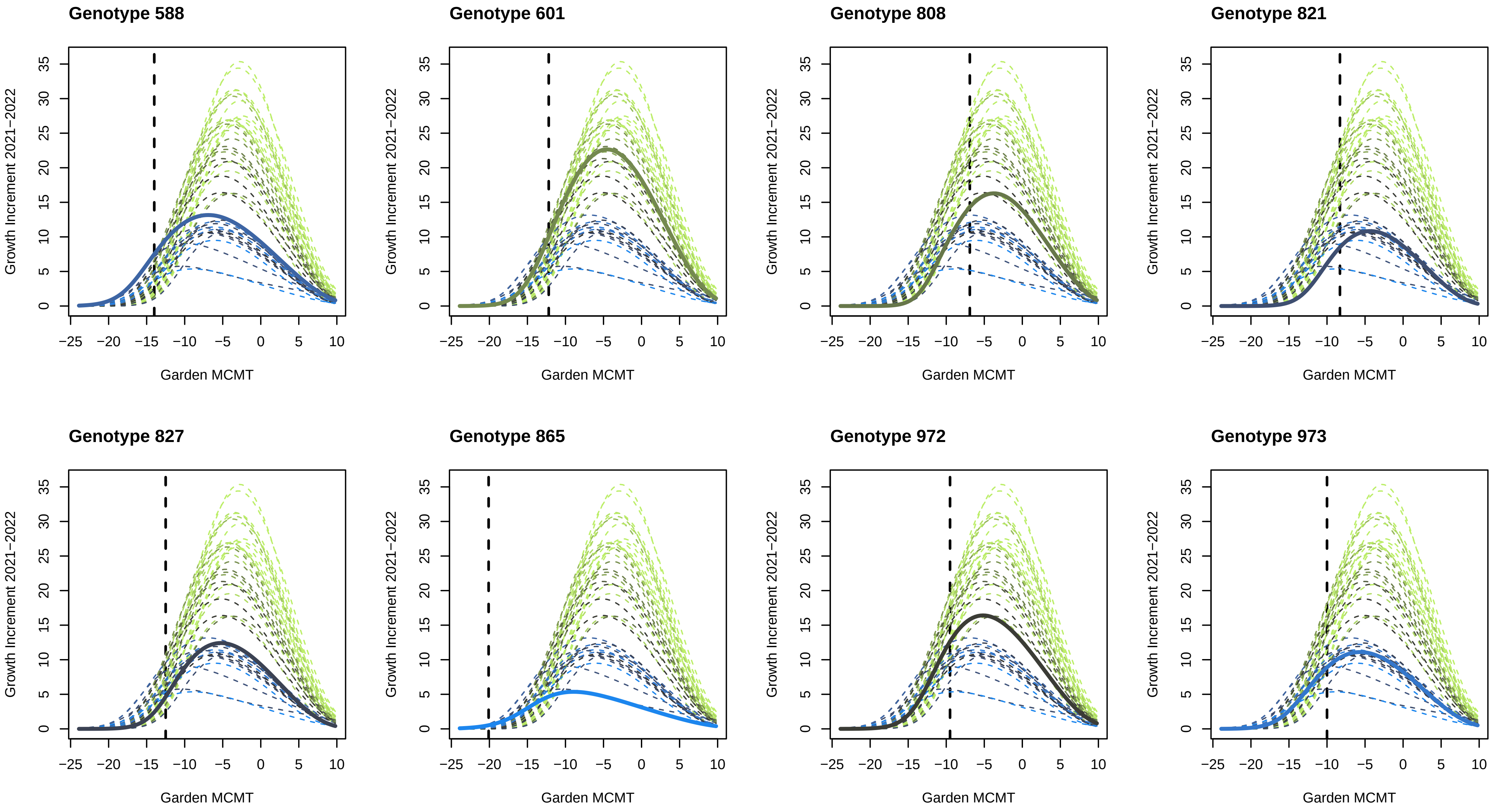
Genotype 588
Genotype 601
Genotype 808
Genotype 821
Genotype 827
Genotype 865
Genotype 972
Genotype 973
Growth Increment 2021−2022
Garden MCMT
0
5
10
15
20
25
30
35
−25
−20
−15
−10
−5
0
5
10
