# Supplementary material for: Variation in responses to temperature across admixed genotypes of Populus trichocarpa × P. balsamifera predict geographic shifts in regions where hybrids are favored
Source: bioRxiv. 2025 May 22:2025.05.16.654548. Preprint. [Version 1] doi: 10.1101/2025.05.16.654548 (PMC12139819; doi:10.1101/2025.05.16.654548)
Supplement: Supplement 3 [file media-3.gz › climate_PCA_garden_provenance_historic_future.html]

Climate PCA


# Climate PCA

#### Alayna Mead

#### 2025-05-13

- 1 Setup
- 2 Reduce climate variables
- 3 PCA
- 4 Plots
  - 4.1 Setup
  - 4.2 Plot with base R
  - 4.3 Plot with ggplot

# 1 Setup

```
library(vegan)
```

```
## Loading required package: permute
```

```
## Loading required package: lattice
```

```
library(viridis) # color palette
```

```
## Loading required package: viridisLite
```

```
library(pals) # color palette
```

```
## 
## Attaching package: 'pals'
```

```
## The following objects are masked from 'package:viridis':
## 
##     cividis, inferno, magma, plasma, turbo, viridis
```

```
## The following objects are masked from 'package:viridisLite':
## 
##     cividis, inferno, magma, plasma, turbo, viridis
```

```
library(psych) # pairs.panels
```

```
## 
## Attaching package: 'psych'
```

```
## The following object is masked from 'package:vegan':
## 
##     pca
```

```
library(ggplot2)
```

```
## 
## Attaching package: 'ggplot2'
```

```
## The following objects are masked from 'package:psych':
## 
##     %+%, alpha
```

```
library(ggnewscale) # multiple color scales
library(ggtext) # for using markdown in legend title 'legend.title = element_markdown()'

# climates in long format - named 'all'
load(file = 'data/clean/546_genotypes_provenance_future_historic_climate_with_gardens_long_format.Rda')
clim <- all
rm(all)

# only need one set of future climates - remove 8 GCMs
clim <- clim[clim$period != "2041_2070_8GCMs",]

# ggplot theme
theme_set(theme_bw(base_size = 18))

sessionInfo()
```

```
## R version 4.5.0 (2025-04-11)
## Platform: x86_64-pc-linux-gnu
## Running under: Arch Linux
## 
## Matrix products: default
## BLAS:   /usr/lib/libblas.so.3.12.0 
## LAPACK: /usr/lib/liblapack.so.3.12.0  LAPACK version 3.12.0
## 
## locale:
##  [1] LC_CTYPE=en_US.UTF-8       LC_NUMERIC=C              
##  [3] LC_TIME=en_US.UTF-8        LC_COLLATE=en_US.UTF-8    
##  [5] LC_MONETARY=en_US.UTF-8    LC_MESSAGES=en_US.UTF-8   
##  [7] LC_PAPER=en_US.UTF-8       LC_NAME=C                 
##  [9] LC_ADDRESS=C               LC_TELEPHONE=C            
## [11] LC_MEASUREMENT=en_US.UTF-8 LC_IDENTIFICATION=C       
## 
## time zone: US/Eastern
## tzcode source: system (glibc)
## 
## attached base packages:
## [1] stats     graphics  grDevices datasets  utils     methods   base     
## 
## other attached packages:
##  [1] ggtext_0.1.2      ggnewscale_0.5.1  ggplot2_3.5.2     psych_2.5.3      
##  [5] pals_1.10         viridis_0.6.5     viridisLite_0.4.2 vegan_2.6-10     
##  [9] lattice_0.22-6    permute_0.9-7    
## 
## loaded via a namespace (and not attached):
##  [1] sass_0.4.10       generics_0.1.3    renv_0.17.3       xml2_1.3.8       
##  [5] digest_0.6.37     magrittr_2.0.3    evaluate_1.0.3    grid_4.5.0       
##  [9] fastmap_1.2.0     maps_3.4.2.1      jsonlite_2.0.0    Matrix_1.7-3     
## [13] gridExtra_2.3     mgcv_1.9-1        scales_1.3.0      jquerylib_0.1.4  
## [17] mnormt_2.1.1      cli_3.6.4         rlang_1.1.6       munsell_0.5.1    
## [21] splines_4.5.0     withr_3.0.2       cachem_1.1.0      yaml_2.3.10      
## [25] tools_4.5.0       parallel_4.5.0    dplyr_1.1.4       colorspace_2.1-1 
## [29] vctrs_0.6.5       mapproj_1.2.11    R6_2.6.1          lifecycle_1.0.4  
## [33] MASS_7.3-65       cluster_2.1.8.1   pkgconfig_2.0.3   pillar_1.10.2    
## [37] bslib_0.9.0       gtable_0.3.6      glue_1.8.0        Rcpp_1.0.14      
## [41] xfun_0.52         tibble_3.2.1      tidyselect_1.2.1  knitr_1.50       
## [45] dichromat_2.0-0.1 htmltools_0.5.8.1 nlme_3.1-168      rmarkdown_2.29   
## [49] compiler_4.5.0    gridtext_0.1.5
```

```
knitr::opts_chunk$set(fig.width = 10, fig.height = 8)
```

# 2 Reduce climate variables

```
# Derived annual variables:
# DD<0              degree-days below 0°C, chilling degree-days
# DD>5              degree-days above 5°C, growing degree-days
# DD<18            degree-days below 18°C, heating degree-days
# DD>18            degree-days above 18°C, cooling degree-days
# NFFD              the number of frost-free days
# FFP                 frost-free period
# bFFP               the day of the year on which FFP begins
# eFFP                the day of the year on which FFP ends
# PAS                 precipitation as snow (mm) between August in previous year and July in current year
# EMT                extreme minimum temperature over 30 years
# EXT                extreme maximum temperature over 30 years
# Eref                 Hargreaves reference evaporation (mm)
# CMD               Hargreaves climatic moisture deficit (mm)
# MAR               mean annual solar radiation (MJ m‐2 d‐1)
# RH                  mean annual relative humidity (%)
# CMI                Hogg’s climate moisture index (mm)
# DD1040 (10<DD<40)    degree-days above 10°C and below 40°C

vars <- c("lat", "lon", "elev", "AHM", "bFFP", "CMD", "CMI", "DD_0", "DD_18", "DD1040", "DD18", "DD5", "eFFP", "EMT", "Eref", "EXT", "FFP", "MAP", "MAR", "MAT", "MCMT", "MSP", "MWMT", "NFFD", "PAS", "RH", "SHM", "TD")

pairs.panels(clim[,vars], scale = T)
```

```
vars <- c("MWMT", "MCMT", "TD", "MAP", "MSP", "SHM", "PAS", "EMT", "CMD", "RH")

pairs.panels(clim[,vars], scale = T)
```

```
# remove EMT

vars <- c("MWMT", "MCMT", "TD", "MAP", "MSP", "SHM", "PAS", "CMD", "RH")

pairs.panels(clim[,vars], scale = T)
```

# 3 PCA

PCA of climate across all home sites and garden sites (both minis and
maxis), 544 genotypes, historic (1961-1990) and future (2041-2070, 13GCM
ensemble) climates, and yearly garden climates for 2020-2023.

Uses rda() funcion from vegan package

```
# reorder so gardens are plotted last
clim <- clim[order(clim$site_type, decreasing = T),]

# add site-period column
clim$site_period <- paste(clim$site_name, clim$period, sep = '_')


# climate variables to use

vars <- c("CMD", "MAP", "MAT", "MCMT", 'MWMT', "PAS", "RH", "TD", "lat", "lon")

# run PCA
rda <- rda(clim[,vars], scale = T)
summary(rda)
```

```
## 
## Call:
## rda(X = clim[, vars], scale = T) 
## 
## Partitioning of correlations:
##               Inertia Proportion
## Total              10          1
## Unconstrained      10          1
## 
## Eigenvalues, and their contribution to the correlations 
## 
## Importance of components:
##                          PC1    PC2    PC3     PC4     PC5     PC6      PC7
## Eigenvalue            4.5419 2.7588 1.0327 0.85492 0.48021 0.19563 0.086291
## Proportion Explained  0.4542 0.2759 0.1033 0.08549 0.04802 0.01956 0.008629
## Cumulative Proportion 0.4542 0.7301 0.8333 0.91883 0.96685 0.98641 0.995043
##                            PC8      PC9      PC10
## Eigenvalue            0.037151 0.012386 3.512e-05
## Proportion Explained  0.003715 0.001239 3.512e-06
## Cumulative Proportion 0.998758 0.999996 1.000e+00
```

```
biplot(rda)
```

```
plot(rda)
```

```
info <- summary(rda)
barplot(info$cont$importance[2,])
```

```
# look at loadings
rda$CA$v
```

```
##              PC1         PC2         PC3         PC4         PC5         PC6
## CMD  -0.32794556  0.18432303  0.45847429 -0.23981506 -0.46933375 -0.37300205
## MAP  -0.01830080 -0.52967231 -0.37276790 -0.03731436 -0.19705325  0.37364357
## MAT  -0.44784465 -0.11936818  0.03476957  0.17028695 -0.10914966  0.14054526
## MCMT -0.39984142 -0.28111065  0.19436269 -0.03829501  0.10626586  0.14832314
## MWMT -0.39406157  0.11216064 -0.11632693  0.39945579 -0.42999590  0.25322812
## PAS   0.23207559 -0.37050773 -0.17660011 -0.44835087 -0.54644018 -0.21952081
## RH    0.01483863 -0.47563961  0.02425598  0.58671481  0.04054783 -0.63097114
## TD    0.22496243  0.42199142 -0.31818126  0.31859114 -0.42222519 -0.01206897
## lat   0.42329262 -0.03396331  0.26435291  0.30189004 -0.12261205  0.11759495
## lon  -0.30418790  0.19204430 -0.62883222 -0.11478943  0.19810215 -0.39382045
##              PC7         PC8           PC9          PC10
## CMD  -0.45261938  0.15050988  0.0620044748  8.432959e-05
## MAP  -0.62173682  0.11112526  0.0376674034  4.734548e-04
## MAT   0.20685242  0.28475016 -0.7742355547 -1.395322e-03
## MCMT  0.22117791  0.03618628  0.3579854990  7.142726e-01
## MWMT  0.25115254 -0.13513864  0.4179730883 -3.923979e-01
## PAS   0.47856811 -0.01577837 -0.0244165148 -1.601509e-04
## RH   -0.06515951 -0.15719087 -0.0005933537 -6.010191e-04
## TD   -0.10141577 -0.13565418 -0.1601017102  5.795136e-01
## lat   0.09742849  0.76557931  0.1848878502  4.308552e-05
## lon   0.02726116  0.48463568  0.1789642550 -1.228071e-04
```

```
barplot(rda$CA$v[,1])
```

```
barplot(rda$CA$v[,2])
```

```
barplot(rda$CA$v[,3])
```

```
# PC1 mostly temperatures/latitude
# PC2 mostly precipitation/continentality
# PC3 mostly CMD/continentality
```

# 4 Plots

## 4.1 Setup

```
# setup colors and shapes for nice plot

# color based on transect or garden
# provenances are colored by genotype ancestry, garden sites each have their own color

clim$gards <-  NA
clim$gards[clim$site_type == 'garden'] <- clim$site_name[clim$site_type == 'garden']

clim$gards <- factor(clim$gards, levels = c("EVERGREEN", "ID", "LOCK", "MORTON", "MSU", "NDSU","NWMO", "OLLU", "OSU", "PENN", "SNHU", "SU", "SWMN", "UCM", "VA", "VT", "WI", "WSU", "WYO"))


# colors for gardens - picking distinguishable colors from transects
kelly(22)
```

```
##  [1] "#F2F3F4" "#222222" "#F3C300" "#875692" "#F38400" "#A1CAF1" "#BE0032"
##  [8] "#C2B280" "#848482" "#008856" "#E68FAC" "#0067A5" "#F99379" "#604E97"
## [15] "#F6A600" "#B3446C" "#DCD300" "#882D17" "#8DB600" "#654522" "#E25822"
## [22] "#2B3D26"
```

```
#col_pal <- c("#000004FF", "#330A5FFF", "#781C6DFF", "#BB3754FF", "#FCB519FF", "#006837", "#f1b300", "#ED6925FF", "#114637", "#285a40", "#18453b", "#ffc82e","#006747", "#0056b8", "#d73f09", "#001e44", "#001e60", "#8a0000", "#3a1807", "#002856", "#e87722", "#2b3e85", "#a60f2d", "#492f24", "#FCFFA4FF")
# remove some colors from output
col_pal <- c("#008856",  "#8DB600", "#BE0032", "#875692", "#A1CAF1", "#2B3D26", "#C2B280", "#F3C300", "#848482", "#0067A5", "#E68FAC", "#F99379", "#604E97", "#B3446C", "#DCD300", "#2b3e85", "#882D17", "#E25822", "#654522")


names(col_pal) <- levels(clim$gards)


# vector with colors
# start with genotype colors
clim$cols <- clim$color_Pt

# add garden colors

for(n in 1:nrow(clim)){
  
  if(clim$site_type[n] == 'garden'){
    clim$cols[n] <- col_pal[clim$gards[n]]
  }
  
}

# shape by year
shapes <- clim$period
shapes[shapes == '2020'] <- 22
shapes[shapes == '2021'] <- 23
shapes[shapes == '2022'] <- 24
shapes[shapes == '2023'] <- 25
shapes[shapes == '1961_1990'] <- 21
shapes[shapes == '2041_2070_13GCMs'] <- 1
shapes <- as.numeric(shapes)
```

## 4.2 Plot with base R

```
# PC1 and PC2

# png(file = 'results/climate/climate_PCA_1-2_transects_and_gardens_4yrs.png', height = 8, width = 10, res = 300, units = 'in')

par(cex.lab = 1.5, mar = c(5,5,3,1))

choices = c(1,2)

plot(rda, choices = choices, type = 'none', xlim = c(-3.5, 4),
     xlab = paste('PC', choices[1], ' (', round(info$cont$importance[2,choices[1]]*100, 1), '% variance explained)', sep = ''),
     ylab = paste('PC', choices[2], ' (', round(info$cont$importance[2,choices[2]]*100, 1), '% variance explained)', sep = ''))
points(rda, choices = choices, display = 'sites', col = clim$cols,  pch = shapes, cex = ifelse(clim$site_type == 'garden', 1.5, 1), lwd = 2)
#text(rda, choices = choices, display = 'sites', col = col, cex = 0.5)
text(rda, choices = choices, display = 'species', col = 'black', cex = 1.5)

legend('topright', pch = c(1,2,3,0,16, 2), col = c(rep('black', 6)), pt.cex = 2, legend = c('2020', '2021', '2022', '2023','1961-1990', '2041-2070'))
```

```
#dev.off()

# PC1 and PC3
# png(file = 'results/climate/climate_PCA_1-3_transects_and_gardens_4yrs.png', height = 8, width = 10, res = 300, units = 'in')

par(cex.lab = 1.5, mar = c(5,5,3,1))

choices = c(1,3)

plot(rda, choices = choices, type = 'none', xlim = c(-3.5, 4),
     xlab = paste('PC', choices[1], ' (', round(info$cont$importance[2,choices[1]]*100, 1), '% variance explained)', sep = ''),
     ylab = paste('PC', choices[2], ' (', round(info$cont$importance[2,choices[2]]*100, 1), '% variance explained)', sep = ''))
points(rda, choices = choices, display = 'sites', col = clim$cols,  pch = shapes, cex = ifelse(clim$site_type == 'garden', 1, 1), lwd = 2)
#text(rda, choices = choices, display = 'sites', col = col, cex = 0.5)
text(rda, choices = choices, display = 'species', col = 'black', cex = 1.5)

legend('topright', pch = c(1,2,3,0,16, 2), col = c(rep('black', 6)), pt.cex = 2, legend = c('2020', '2021', '2022', '2023','1961-1990', '2041-2070'))
```

```
#dev.off()

# PC1 and PC3, version without legend
#png(file = 'results/climate/climate_PCA_1-3_transects_and_gardens_4yrs_nolegend.png', height = 8, width = 10, res = 300, units = 'in')

par(cex.lab = 1.5, mar = c(5,5,3,1))

choices = c(1,3)

plot(rda, choices = choices, type = 'none', xlim = c(-3.5, 4),
     xlab = paste('PC', choices[1], ' (', round(info$cont$importance[2,choices[1]]*100, 1), '% variance explained)', sep = ''),
     ylab = paste('PC', choices[2], ' (', round(info$cont$importance[2,choices[2]]*100, 1), '% variance explained)', sep = ''))
points(rda, choices = choices, display = 'sites', col = clim$cols,  pch = shapes, cex = ifelse(clim$site_type == 'garden', 1, 1), lwd = 2)
#text(rda, choices = choices, display = 'sites', col = col, cex = 0.5)
text(rda, choices = choices, display = 'species', col = 'black', cex = 1.5)
```

```
#dev.off()
```

## 4.3 Plot with ggplot

```
#####################
# ggplot

# merge PCA data for each individual with information dataframe
to_plot <- cbind.data.frame(clim, rda$CA$u)

clims <- rda$CA$v

no_fut <- which(to_plot$period != '2041_2070_13GCMs')

# only minis
no_fut_minis <- which(to_plot$period != '2041_2070_13GCMs' & (to_plot$in_minis == TRUE | to_plot$site_type == 'garden'))

ggplot(dat = to_plot, aes(x = PC1, y = PC2)) +
  geom_line(dat = subset(to_plot, site_type == 'provenance' & in_minis == TRUE), aes(x = PC1, y = PC2, group = site_name), arrow = arrow(ends = 'first', angle = 20, length = unit(0.1, "inches")), col = rgb(0,0,0, alpha = 0.3)) + # arrows
  geom_point(dat = to_plot[no_fut_minis,], aes(x = PC1, y = PC2), bg = clim$cols[no_fut_minis], pch = shapes[no_fut_minis], cex = 2.5, show.legend = T) + # sites
  geom_text(data = clims, label = rownames(clims), aes(x = PC1/3, y = PC2/3), size = 8) + # climate loadings
  scale_color_manual(name = 'sites', breaks = names(col_pal), values = col_pal)
```

```
## Warning: No shared levels found between `names(values)` of the manual scale and the
## data's colour values.
```

```
ggplot(dat = to_plot, aes(x = PC1, y = PC2)) +
  geom_line(dat = subset(to_plot, site_type == 'provenance' & in_minis == TRUE), 
            aes(x = PC1, y = PC2, 
                group = site_name), 
            arrow = arrow(ends = 'first', angle = 20, length = unit(0.1, "inches")), col = rgb(0,0,0, alpha = 0.3)) + # arrows
  geom_point(dat = to_plot[no_fut_minis,], 
             aes(x = PC1, y = PC2), 
             bg = clim$cols[no_fut_minis], 
             pch = shapes[no_fut_minis], 
             cex = 2.5, 
             show.legend = T) + # sites
  geom_text(data = clims, 
            label = rownames(clims), 
            aes(x = PC1/3, y = PC2/3), 
            size = 8) + # climate loadings
  scale_color_manual(name = 'sites', breaks = names(col_pal), values = col_pal)
```

```
## Warning: No shared levels found between `names(values)` of the manual scale and the
## data's colour values.
```

```
# all years
ggplot(dat = to_plot, aes(x = PC1, y = PC2)) +
  geom_line(dat = subset(to_plot, site_type == 'provenance' & in_minis == TRUE), 
            aes(x = PC1, y = PC2, 
                group = site_name), 
            arrow = arrow(ends = 'first', angle = 20, length = unit(0.1, "inches")), col = rgb(0,0,0, alpha = 0.3)) + # arrows
  geom_point(dat = subset(to_plot[no_fut_minis,], site_type == 'garden'),
             aes(x = PC1, y = PC2, color = site_name, shape = period, size = period)) +
  scale_size_manual(values = c("1961_1990" = 3, "2041_2070_13GCMs" = 1, "2021" = 4, "2023" = 1, "2020" = 1, "2022" = 1)) +
  scale_color_manual(name = 'Garden', values = col_pal) + # plot gardens
  new_scale_color() +
  geom_point(dat = subset(to_plot[no_fut_minis,], site_type == 'provenance'), 
             aes(x = PC1, y = PC2, fill = Pt), pch = 21, size = 4) + 
  scale_fill_gradient2(name = '% P. trichocarpa ancestry',
                       low = 'dodgerblue2', 
                       mid = 'grey20', 
                       high = 'darkolivegreen2', 
                       midpoint = 0.5)
```

```
# only 2021 for minis
no_fut_minis <- which(to_plot$period != '2041_2070_13GCMs' & (to_plot$in_minis == TRUE | to_plot$site_type == 'garden'))
  
#no_fut_minis <- which(to_plot$period == '2021' & (to_plot$in_minis == TRUE | to_plot$site_type == 'garden'))
  
# PC1 and PC2

ggplot(dat = to_plot, aes(x = PC1, y = PC2)) +
  xlab(paste('PC1 (', round(info$cont$importance['Proportion Explained','PC1'],3)*100, '% variance explained)', sep = '')) +
  ylab(paste('PC2 (', round(info$cont$importance['Proportion Explained','PC2'],3)*100, '% variance explained)', sep = '')) +
  geom_vline(xintercept = 0, col = 'grey60') + 
  geom_hline(yintercept = 0, col = 'grey60') + 
  geom_line(dat = subset(to_plot, site_type == 'provenance' & in_minis == TRUE), 
            aes(x = PC1, y = PC2, 
                group = site_name), 
            arrow = arrow(ends = 'first', angle = 20, length = unit(0.1, "inches")), col = rgb(0,0,0, alpha = 0.3)) + # arrows
  geom_point(dat = subset(to_plot[no_fut_minis,], site_type == 'garden' & period == '2021'),
             aes(x = PC1, y = PC2, fill = site_name), pch = 24, size = 4) +
  scale_fill_manual(name = 'Garden', values = col_pal) + # plot gardens
  new_scale_fill() +
  geom_point(dat = subset(to_plot[no_fut_minis,], site_type == 'provenance'), 
             aes(x = PC1, y = PC2, fill = Pt), pch = 21, size = 4) + 
  scale_fill_gradient2(name = '% P. trichocarpa ancestry',
                       low = 'dodgerblue2', 
                       mid = 'grey20', 
                       high = 'darkolivegreen2', 
                       midpoint = 0.5) + # provenance points (historic)
  geom_text(data = clims, 
            label = rownames(clims), 
            aes(x = PC1/4, y = PC2/4), 
            size = 6) # climate loadings
```

```
#ggsave('results/climate/climatePCA_1-2_gardens2021_provenanceMinisPastFutureArrows.png', height = 8, width = 12)


# PC1 and PC3

ggplot(dat = to_plot, aes(x = PC1, y = PC3)) +
  xlab(paste('PC1 (', round(info$cont$importance['Proportion Explained','PC1'],3)*100, '% variance explained)', sep = '')) +
  ylab(paste('PC3 (', round(info$cont$importance['Proportion Explained','PC3'],3)*100, '% variance explained)', sep = '')) +
  geom_vline(xintercept = 0, col = 'grey60') + 
  geom_hline(yintercept = 0, col = 'grey60') + 
  geom_line(dat = subset(to_plot, site_type == 'provenance' & in_minis == TRUE), 
            aes(x = PC1, y = PC3, 
                group = site_name), 
            arrow = arrow(ends = 'first', angle = 20, length = unit(0.1, "inches")), col = rgb(0,0,0, alpha = 0.3)) + # arrows
  geom_point(dat = subset(to_plot[no_fut_minis,], site_type == 'garden' & period == '2021'),
             aes(x = PC1, y = PC3, fill = site_name), pch = 24, size = 4) +
  scale_fill_manual(name = 'Garden', values = col_pal) + # plot gardens
  new_scale_fill() +
  geom_point(dat = subset(to_plot[no_fut_minis,], site_type == 'provenance'), 
             aes(x = PC1, y = PC3, fill = Pt), pch = 21, size = 4) + 
  scale_fill_gradient2(name = '% P. trichocarpa ancestry',
                       low = 'dodgerblue2', 
                       mid = 'grey20', 
                       high = 'darkolivegreen2', 
                       midpoint = 0.5) + # provenance points (historic)
  geom_text(data = clims, 
            label = rownames(clims), 
            aes(x = PC1/4, y = PC3/4), 
            size = 6) # climate loadings
```

```
#ggsave('results/climate/climatePCA_1-3_gardens2021_provenanceMinisPastFutureArrows.png', height = 8, width = 12)


# PC2 and PC3

ggplot(dat = to_plot, aes(x = PC2, y = PC3)) +
  xlab(paste('PC2 (', round(info$cont$importance['Proportion Explained','PC2'],3)*100, '% variance explained)', sep = '')) +
  ylab(paste('PC3 (', round(info$cont$importance['Proportion Explained','PC3'],3)*100, '% variance explained)', sep = '')) +
  geom_vline(xintercept = 0, col = 'grey60') + 
  geom_hline(yintercept = 0, col = 'grey60') + 
  geom_line(dat = subset(to_plot, site_type == 'provenance' & in_minis == TRUE), 
            aes(x = PC2, y = PC3, 
                group = site_name), 
            arrow = arrow(ends = 'first', angle = 20, length = unit(0.1, "inches")), col = rgb(0,0,0, alpha = 0.3)) + # arrows
  geom_point(dat = subset(to_plot[no_fut_minis,], site_type == 'garden' & period == '2021'),
             aes(x = PC2, y = PC3, fill = site_name), pch = 24, size = 4) +
  scale_fill_manual(name = 'Garden', values = col_pal) + # plot gardens
  new_scale_fill() +
  geom_point(dat = subset(to_plot[no_fut_minis,], site_type == 'provenance'), 
             aes(x = PC2, y = PC3, fill = Pt), pch = 21, size = 4) + 
  scale_fill_gradient2(name = '% P. trichocarpa ancestry',
                       low = 'dodgerblue2', 
                       mid = 'grey20', 
                       high = 'darkolivegreen2', 
                       midpoint = 0.5) + # provenance points (historic)
  geom_text(data = clims, 
            label = rownames(clims), 
            aes(x = PC2/4, y = PC3/4), 
            size = 6) # climate loadings
```

```
#ggsave('results/climate/climatePCA_2-3_gardens2021_provenanceMinisPastFutureArrows.png', height = 8, width = 12)


##############################

# 2021 and 2022 for minis

ggplot(dat = to_plot, aes(x = PC1, y = PC2)) +
  xlab(paste('PC1 (', round(info$cont$importance['Proportion Explained','PC1'],3)*100, '% variance explained)', sep = '')) +
  ylab(paste('PC2 (', round(info$cont$importance['Proportion Explained','PC2'],3)*100, '% variance explained)', sep = '')) +
  geom_vline(xintercept = 0, col = 'grey60') + 
  geom_hline(yintercept = 0, col = 'grey60') + 
  geom_line(dat = subset(to_plot, site_type == 'provenance' & in_minis == TRUE), 
            aes(x = PC1, y = PC2, 
                group = site_name), 
            arrow = arrow(ends = 'first', angle = 20, length = unit(0.1, "inches")), col = rgb(0,0,0, alpha = 0.3)) + # arrows
  geom_point(dat = subset(to_plot[no_fut_minis,], site_type == 'garden' & period %in% c('2021', '2022'), ),
             aes(x = PC1, y = PC2, fill = site_name, shape = period),  size = 4) +
  scale_shape_manual(name = 'Year', values = c(24,22)) +
  scale_fill_manual(name = 'Garden', values = col_pal) + # plot gardens
    guides(shape = guide_legend(order = 1), 
         fill = guide_legend(order = 2, override.aes = list(shape = 22), ncol = 2)) + # order legends and get garden legend to show correct colors 
  new_scale_fill() +
  geom_point(dat = subset(to_plot[no_fut_minis,], site_type == 'provenance'), 
             aes(x = PC1, y = PC2, fill = Pt), pch = 21, size = 4) + 
  scale_fill_gradient2(name = "% *P. trichocarpa*<br>ancestry",
                       low = 'dodgerblue2', 
                       mid = 'grey20', 
                       high = 'darkolivegreen2', 
                       midpoint = 0.5) + # provenance points (historic)
  theme(legend.title = element_markdown()) +
  geom_text(data = clims, 
            label = rownames(clims), 
            aes(x = PC1/4, y = PC2/4), 
            size = 6) # climate loadings
```

```
#ggsave('results/climate/climatePCA_1-2_gardens2021-2022_provenanceMinisPastFutureArrows.png', height = 8, width = 12)


# PC1 and PC3

ggplot(dat = to_plot, aes(x = PC1, y = PC3)) +
  xlab(paste('PC1 (', round(info$cont$importance['Proportion Explained','PC1'],3)*100, '% variance explained)', sep = '')) +
  ylab(paste('PC3 (', round(info$cont$importance['Proportion Explained','PC3'],3)*100, '% variance explained)', sep = '')) +
  geom_vline(xintercept = 0, col = 'grey60') + 
  geom_hline(yintercept = 0, col = 'grey60') + 
  geom_line(dat = subset(to_plot, site_type == 'provenance' & in_minis == TRUE), 
            aes(x = PC1, y = PC3, 
                group = site_name), 
            arrow = arrow(ends = 'first', angle = 20, length = unit(0.1, "inches")), col = rgb(0,0,0, alpha = 0.3)) + # arrows
  geom_point(dat = subset(to_plot[no_fut_minis,], site_type == 'garden' & period %in% c('2021', '2022'), ),
             aes(x = PC1, y = PC3, fill = site_name, shape = period),  size = 4) +
  scale_shape_manual(name = 'Year', values = c(24,22)) +
  scale_fill_manual(name = 'Garden', values = col_pal) + # plot gardens
    guides(shape = guide_legend(order = 1), 
         fill = guide_legend(order = 2, override.aes = list(shape = 22), ncol = 2)) + # order legends and get garden legend to show correct colors 
  new_scale_fill() +
  geom_point(dat = subset(to_plot[no_fut_minis,], site_type == 'provenance'), 
             aes(x = PC1, y = PC3, fill = Pt), pch = 21, size = 4) + 
  scale_fill_gradient2(name = "% *P. trichocarpa*<br>ancestry",
                       low = 'dodgerblue2', 
                       mid = 'grey20', 
                       high = 'darkolivegreen2', 
                       midpoint = 0.5) + # provenance points (historic)
  theme(legend.title = element_markdown()) +
  geom_text(data = clims, 
            label = rownames(clims), 
            aes(x = PC1/4, y = PC3/4), 
            size = 6) # climate loadings
```

```
#ggsave('results/climate/climatePCA_1-3_gardens2021-2022_provenanceMinisPastFutureArrows.png', height = 8, width = 12)

################################
# all 544 genotypes, not just those in minis

# PC1 and PC2

ggplot(dat = to_plot, aes(x = PC1, y = PC2)) +
  xlab(paste('PC1 (', round(info$cont$importance['Proportion Explained','PC1'],3)*100, '% variance explained)', sep = '')) +
  ylab(paste('PC2 (', round(info$cont$importance['Proportion Explained','PC2'],3)*100, '% variance explained)', sep = '')) +
  geom_vline(xintercept = 0, col = 'grey60') + 
  geom_hline(yintercept = 0, col = 'grey60') + 
  geom_line(dat = subset(to_plot, site_type == 'provenance'), 
            aes(x = PC1, y = PC2, 
                group = site_name), 
            arrow = arrow(ends = 'first', angle = 20, length = unit(0.1, "inches")), col = rgb(0,0,0, alpha = 0.3)) + # arrows
  geom_point(dat = subset(to_plot, site_type == 'garden' & period == '2021'),
             aes(x = PC1, y = PC2, fill = site_name), pch = 24, size = 4) +
  scale_fill_manual(name = 'Garden', values = col_pal) + # plot gardens
  new_scale_fill() +
  geom_point(dat = subset(to_plot[no_fut,], site_type == 'provenance'), 
             aes(x = PC1, y = PC2, fill = Pt), pch = 21, size = 4) + 
  scale_fill_gradient2(name = '% P. trichocarpa ancestry',
                       low = 'dodgerblue2', 
                       mid = 'grey20', 
                       high = 'darkolivegreen2', 
                       midpoint = 0.5) + # provenance points (historic)
  geom_text(data = clims, 
            label = rownames(clims), 
            aes(x = PC1/4, y = PC2/4), 
            size = 6) # climate loadings
```

```
#ggsave('results/climate/climatePCA_1-2_gardens2021_provenanceAllPastFutureArrows.png', height = 8, width = 12)


# PC1 and PC3


ggplot(dat = to_plot, aes(x = PC1, y = PC3)) +
  xlab(paste('PC1 (', round(info$cont$importance['Proportion Explained','PC1'],3)*100, '% variance explained)', sep = '')) +
  ylab(paste('PC3 (', round(info$cont$importance['Proportion Explained','PC3'],3)*100, '% variance explained)', sep = '')) +
  geom_vline(xintercept = 0, col = 'grey60') + 
  geom_hline(yintercept = 0, col = 'grey60') + 
  geom_line(dat = subset(to_plot, site_type == 'provenance'), 
            aes(x = PC1, y = PC3, 
                group = site_name), 
            arrow = arrow(ends = 'first', angle = 20, length = unit(0.1, "inches")), col = rgb(0,0,0, alpha = 0.3)) + # arrows
  geom_point(dat = subset(to_plot, site_type == 'garden' & period == '2021'),
             aes(x = PC1, y = PC3, fill = site_name), pch = 24, size = 4) +
  scale_fill_manual(name = 'Garden', values = col_pal) + # plot gardens
  new_scale_fill() +
  geom_point(dat = subset(to_plot[no_fut,], site_type == 'provenance'), 
             aes(x = PC1, y = PC3, fill = Pt), pch = 21, size = 4) + 
  scale_fill_gradient2(name = '% P. trichocarpa ancestry',
                       low = 'dodgerblue2', 
                       mid = 'grey20', 
                       high = 'darkolivegreen2', 
                       midpoint = 0.5) + # provenance points (historic)
  geom_text(data = clims, 
            label = rownames(clims), 
            aes(x = PC1/4, y = PC3/4), 
            size = 6) # climate loadings
```

```
#ggsave('results/climate/climatePCA_1-3_gardens2021_provenanceAllPastFutureArrows.png', height = 8, width = 12)
```
