# Supplementary material for: Variation in responses to temperature across admixed genotypes of Populus trichocarpa × P. balsamifera predict geographic shifts in regions where hybrids are favored
Source: bioRxiv. 2025 May 22:2025.05.16.654548. Preprint. [Version 1] doi: 10.1101/2025.05.16.654548 (PMC12139819; doi:10.1101/2025.05.16.654548)
Supplement: Supplement 3 [file media-3.gz › map_best_genotype_by_climate.html]

 

 

 

 
 
 


 

 

 Map optimal genotypes by climate 

 
 
 
 
 
 
 
 
 

 


 
 
 


 


 

 

 


 

 


 


 


 Map optimal genotypes by climate 
 Alayna Mead 
 2025-05-13 

 

 
 
   1  Setup 
 
   1.1  Load packages and data  
   1.2  Setup genotype info  
   1.3  Merge genotype responses  
   1.4  Load climate rasters  
  
   2  Mapping (color by species
ancestry) 
 
   2.1  Functions  
   2.2  Plot  
  
   3  Mapping (color by K=3) 
 
   3.1  Functions  
   3.2  Plot  
  
 
 

 Using reaction norms modeled in the
‘transfer_function_multiyear_linear_mixed_effects_model.Rmd’ script,
identify the genotype having highest growth (taking into account
mortality probability) across the sampled hybrid zone under historic and
future values of MCMT. Maps show where the ‘optimal’ genotype/species
ancestry could shift spatially under future temperatures, resulting in
spatial shifts in the location of the hybrid zone. 
 
  1  Setup 
 
  1.1  Load packages and
data 
       library (sf)  # mapping     
  ## Linking to GEOS 3.13.1, GDAL 3.10.2, PROJ 9.6.0; sf_use_s2() is TRUE  
       library (terra)  # mapping     
  ## terra 1.8.42  
       library (RColorBrewer)  # colors  
    library (circlize)  # colorRamp2     
  ## ========================================
## circlize version 0.4.16
## CRAN page: https://cran.r-project.org/package=circlize
## Github page: https://github.com/jokergoo/circlize
## Documentation: https://jokergoo.github.io/circlize_book/book/
## 
## If you use it in published research, please cite:
## Gu, Z. circlize implements and enhances circular visualization
##   in R. Bioinformatics 2014.
## 
## This message can be suppressed by:
##   suppressPackageStartupMessages(library(circlize))
## ========================================  
       # predictions of growth and mortality across climate for each genotype  
    load ( 'results/model_prediction/predictedGrowth_acrossClimates_byGenotype_garden_MCMT_2021-2022.Rdata' ) 
    # rename  
   preds  <-  predictions 
    rm (predictions) 
    
    # make genotype info its own df  
   geno.info  <-  preds $ genotypes 
    #cleanup  
   geno.info $ genotype  <-   rownames (geno.info) 
    rownames (geno.info)  <-   paste ( 'genotype_' ,  rownames (geno.info),  sep =   '' ) 
    
    
    # garden data  
    load ( 'data/clean/garden_climates_average1961-1990_yearly2020-2023.Rdata' ) 
   gards  <-  clim 
    
    # provenance data  
    # extract from garden climate and phenotype origin data - named 'dat'  
    load ( 'data/clean/mini_garden_phenotypic_and_climate_data_2021-2023.Rdata' ) 
    # get first instance of genotype, extract genotype info  
   provs  <-  dat[ !   duplicated (dat $ Genotype),  c ( "Genotype" ,  "Pb" ,  "Pt" ,  
    "transect" ,  "provenance_latitude" ,  "provenance_longitude" ,  "provenance_elevation_m" )] 
    # remove NA genotype  
   provs  <-  provs[ !   is.na (provs $ Genotype),] 
    # remove genotypes without genetic data  
   provs  <-  provs[ !   is.na (provs $ Pt),] 
    
    # change rownames to genotypes  
    rownames (provs)  <-  provs $ Genotype 
    
    sessionInfo ()    
  ## R version 4.5.0 (2025-04-11)
## Platform: x86_64-pc-linux-gnu
## Running under: Arch Linux
## 
## Matrix products: default
## BLAS:   /usr/lib/libblas.so.3.12.0 
## LAPACK: /usr/lib/liblapack.so.3.12.0  LAPACK version 3.12.0
## 
## locale:
##  [1] LC_CTYPE=en_US.UTF-8       LC_NUMERIC=C              
##  [3] LC_TIME=en_US.UTF-8        LC_COLLATE=en_US.UTF-8    
##  [5] LC_MONETARY=en_US.UTF-8    LC_MESSAGES=en_US.UTF-8   
##  [7] LC_PAPER=en_US.UTF-8       LC_NAME=C                 
##  [9] LC_ADDRESS=C               LC_TELEPHONE=C            
## [11] LC_MEASUREMENT=en_US.UTF-8 LC_IDENTIFICATION=C       
## 
## time zone: US/Eastern
## tzcode source: system (glibc)
## 
## attached base packages:
## [1] stats     graphics  grDevices datasets  utils     methods   base     
## 
## other attached packages:
## [1] circlize_0.4.16    RColorBrewer_1.1-3 terra_1.8-42       sf_1.0-20         
## 
## loaded via a namespace (and not attached):
##  [1] cli_3.6.4           knitr_1.50          rlang_1.1.6        
##  [4] xfun_0.52           DBI_1.2.3           KernSmooth_2.23-26 
##  [7] renv_0.17.3         jsonlite_2.0.0      colorspace_2.1-1   
## [10] htmltools_0.5.8.1   e1071_1.7-16        GlobalOptions_0.1.2
## [13] sass_0.4.10         rmarkdown_2.29      grid_4.5.0         
## [16] evaluate_1.0.3      jquerylib_0.1.4     classInt_0.4-11    
## [19] fastmap_1.2.0       yaml_2.3.10         lifecycle_1.0.4    
## [22] compiler_4.5.0      codetools_0.2-20    Rcpp_1.0.14        
## [25] digest_0.6.37       R6_2.6.1            class_7.3-23       
## [28] shape_1.4.6.1       magrittr_2.0.3      bslib_0.9.0        
## [31] tools_4.5.0         proxy_0.4-27        units_0.8-7        
## [34] cachem_1.1.0  
      knitr :: opts_chunk $  set ( fig.width =   12 ,  
                          fig.height =   10 )    
 
 
  1.2  Setup genotype
info 
       # get additional genotype info  
    
    # get first instances of each genotype  
   genos  <-  dat[ match (geno.info $ genotype, dat $ Genotype),] 
    
    # check for match  
    cbind ( as.character (genos $ Genotype), geno.info $ genotype)    
  ##       [,1]  [,2] 
##  [1,] "206" "206"
##  [2,] "210" "210"
##  [3,] "218" "218"
##  [4,] "233" "233"
##  [5,] "255" "255"
##  [6,] "258" "258"
##  [7,] "307" "307"
##  [8,] "311" "311"
##  [9,] "317" "317"
## [10,] "333" "333"
## [11,] "334" "334"
## [12,] "342" "342"
## [13,] "353" "353"
## [14,] "364" "364"
## [15,] "374" "374"
## [16,] "380" "380"
## [17,] "381" "381"
## [18,] "405" "405"
## [19,] "411" "411"
## [20,] "416" "416"
## [21,] "419" "419"
## [22,] "423" "423"
## [23,] "427" "427"
## [24,] "432" "432"
## [25,] "437" "437"
## [26,] "443" "443"
## [27,] "453" "453"
## [28,] "463" "463"
## [29,] "469" "469"
## [30,] "522" "522"
## [31,] "533" "533"
## [32,] "543" "543"
## [33,] "545" "545"
## [34,] "564" "564"
## [35,] "567" "567"
## [36,] "572" "572"
## [37,] "588" "588"
## [38,] "601" "601"
## [39,] "808" "808"
## [40,] "821" "821"
## [41,] "827" "827"
## [42,] "865" "865"
## [43,] "972" "972"
## [44,] "973" "973"  
       # add to dataframe  
   geno.info $ transect  <-  genos $ transect 
   geno.info $ interspecific_heterozygosity  <-  genos $ interspecific_heterozygosity 
   geno.info $ heterozygosity  <-  genos $ heterozygosity 
   geno.info $ hybrid_index  <-  genos $ hybrid_index 
   geno.info $ plastid_ID  <-  genos $ plastid_ID 
   geno.info $ Pt  <-  genos $ Pt 
   geno.info $ color_Pt  <-  genos $ color_Pt 
   geno.info $ color_k3  <-  genos $ color_k3 
    
    str (geno.info)    
  ## 'data.frame':    44 obs. of  14 variables:
##  $ pc1                         : num  0.00423 -0.03207 -0.02393 -0.03496 -0.00995 ...
##  $ pc2                         : num  0.02407 -0.00722 0.01811 0.039 0.03374 ...
##  $ pc3                         : num  -0.0243 -0.0365 -0.0505 -0.0418 -0.0256 ...
##  $ home_clim                   : num  -9.6 -9.9 -8.9 -9.3 -12.2 -12.4 -6.9 -5.8 -5.8 -4.8 ...
##  $ Pt                          : num  0.571 0.908 0.864 0.966 0.715 ...
##  $ optimal_clim                : num  -4.04 -4.04 -3.66 -3.66 -4.79 ...
##  $ genotype                    : chr  "206" "210" "218" "233" ...
##  $ transect                    : Factor w/ 5 levels "Alaska","Cassiar",..: 3 3 3 3 3 3 5 5 5 5 ...
##  $ interspecific_heterozygosity: num  0.4066 NA 0.0956 NA 0.1766 ...
##  $ heterozygosity              : num  0.0996 0.0992 0.0925 0.0989 0.0942 ...
##  $ hybrid_index                : num  0.319 NA 0.0376 NA 0.1769 ...
##  $ plastid_ID                  : chr  "balsamifera" "trichocarpa" "balsamifera" "balsamifera" ...
##  $ color_Pt                    : chr  "#464A3BFF" "#A2C95FFF" "#95B75BFF" "#B2E065FF" ...
##  $ color_k3                    : chr  "#1B7F65" "#846912" "#4D961C" "#3DC200" ...  
 
 
  1.3  Merge genotype
responses 
 Merge each genotype’s response to MCMT into one dataframe for
comparison across genotypes 
       # get the overall predictions by genotype and join into one dataframe to identify optimal genotype for each climate  
    
    # get first genotype  
   pred.overall  <-  preds $ overall[[ 1 ]] 
    
    # rename column  
    colnames (pred.overall)[ 2 ]  <-   names (preds $ overall)[ 1 ] 
    
    
    # loop through other genotypes and add them  
    for (n  in   2  :  length (preds $ overall)){ 
      
     df  <-  preds $ overall[[n]] 
      
     pred.overall[, names (preds $ overall)[n]]  <-  df[, 2 ] 
      
   } 
    
    head (pred.overall)    
  ##   garden_clim genotype_206 pred_se genotype_210  genotype_218  genotype_233
## 1   -23.90000 0.0001453033      NA  0.001661313 -0.0005488445 -0.0007477098
## 2   -23.52525 0.0008145990      NA  0.002649368  0.0002324151 -0.0005727152
## 3   -23.15051 0.0019332056      NA  0.004098252  0.0016045524 -0.0001682450
## 4   -22.77576 0.0037126400      NA  0.006188216  0.0038470405  0.0005955691
## 5   -22.40101 0.0064433609      NA  0.009159220  0.0073407967  0.0019025695
## 6   -22.02626 0.0105181509      NA  0.013327362  0.0125968296  0.0040081112
##   genotype_255 genotype_258  genotype_307  genotype_311 genotype_317
## 1  0.003805555   0.01860611 -0.0007562458 -0.0004039832 6.948694e-05
## 2  0.006222452   0.02524778 -0.0009025296 -0.0003998759 2.372378e-04
## 3  0.009765093   0.03396243 -0.0010361616 -0.0003212149 5.263518e-04
## 4  0.014860769   0.04530761 -0.0011261331 -0.0001126377 1.001396e-03
## 5  0.022070746   0.05996561 -0.0011221848  0.0003094876 1.755144e-03
## 6  0.032124017   0.07876507 -0.0009464785  0.0010684587 2.918889e-03
##    genotype_333  genotype_334  genotype_342 genotype_353 genotype_364
## 1 -1.095539e-04 -1.139752e-04 -7.107731e-05 0.0002346455  0.000644507
## 2 -8.759561e-05 -1.141680e-04 -6.451135e-05 0.0004022242  0.001024722
## 3 -2.756082e-05 -9.365559e-05 -3.840461e-05 0.0006624340  0.001599075
## 4  9.623987e-05 -3.648954e-05  2.170487e-05 0.0010584855  0.002454974
## 5  3.223790e-04  8.170086e-05  1.380191e-04 0.0016509541  0.003714517
## 6  7.075636e-04  2.972501e-04  3.438409e-04 0.0025237490  0.005546452
##   genotype_374 genotype_380 genotype_381 genotype_405 genotype_411 genotype_416
## 1  0.001303226  0.001900577  0.002171135    0.1026272   0.06154594   0.01065818
## 2  0.001971294  0.002792657  0.003239044    0.1254766   0.07962004   0.01462319
## 3  0.002941899  0.004062166  0.004773389    0.1529474   0.10256484   0.01993558
## 4  0.004336623  0.005852323  0.006955304    0.1858737   0.13157179   0.02700721
## 5  0.006320055  0.008354450  0.010027854    0.2252171   0.16809249   0.03636068
## 6  0.009112912  0.011821945  0.014314319    0.2720793   0.21388572   0.04865472
##   genotype_419 genotype_423 genotype_427 genotype_432 genotype_437
## 1   0.01979687  0.004140289  0.002022510  0.001742792 2.083826e-05
## 2   0.02695186  0.005848717  0.002905605  0.002599711 4.632462e-05
## 3   0.03646607  0.008203736  0.004140725  0.003835822 9.176714e-05
## 4   0.04903868  0.011426821  0.005854513  0.005601381 1.698369e-04
## 5   0.06555132  0.015806728  0.008213951  0.008099184 3.001007e-04
## 6   0.08710875  0.021717066  0.011437362  0.011600313 5.122747e-04
##    genotype_443  genotype_453  genotype_463  genotype_469  genotype_522
## 1 -3.405892e-05 -3.604828e-05 -0.0001412089 -0.0003234593 -6.376468e-05
## 2 -3.399126e-05 -4.152790e-05 -0.0001846170 -0.0004112153  1.887725e-04
## 3 -2.346953e-05 -4.152389e-05 -0.0002335555 -0.0005033438  6.356856e-04
## 4  7.975878e-06 -2.885478e-05 -0.0002828596 -0.0005846372  1.376603e-03
## 5  7.810963e-05  9.168883e-06 -0.0003216962 -0.0006264336  2.551654e-03
## 6  2.161200e-04  9.406120e-05 -0.0003299451 -0.0005788848  4.354868e-03
##   genotype_533 genotype_543 genotype_545 genotype_564 genotype_567 genotype_572
## 1 9.796994e-05   0.00997307  0.005722742   0.01015404   0.01669202   0.01806589
## 2 6.508648e-04   0.01433008  0.008893600   0.01465558   0.02289059   0.02396830
## 3 1.559214e-03   0.02030636  0.013404834   0.02084088   0.03108871   0.03156783
## 4 2.981932e-03   0.02841833  0.019722373   0.02924724   0.04184065   0.04128529
## 5 5.134050e-03   0.03932193  0.028446212   0.04055611   0.05582855   0.05362795
## 6 8.302429e-03   0.05384373  0.040341602   0.05562446   0.07388533   0.06920291
##   genotype_588 genotype_601  genotype_808  genotype_821 genotype_827
## 1   0.05864509  0.002224909 -4.528101e-05 -4.234797e-06 0.0005726378
## 2   0.07700620  0.003738267 -5.948961e-05  1.154623e-05 0.0009001163
## 3   0.10045541  0.005996458 -7.456155e-05  4.366055e-05 0.0013919919
## 4   0.13021542  0.009298395 -8.721068e-05  1.033954e-04 0.0021213891
## 5   0.16775328  0.014042733 -9.070855e-05  2.083540e-04 0.0031900826
## 6   0.21481549  0.020754936 -7.250923e-05  3.854411e-04 0.0047381810
##   genotype_865 genotype_972 genotype_973
## 1   0.08367808  0.001978613  0.006505981
## 2   0.10179996  0.002889569  0.009246759
## 3   0.12342780  0.004179856  0.012990662
## 4   0.14914923  0.005991342  0.018055764
## 5   0.17963117  0.008512826  0.024845478
## 6   0.21562559  0.011993422  0.033866880  
       # which genotype has highest predicted success for each value of MCMT (row)?  
    
    # just get genotypes  
   tmp  <-  pred.overall[, -  1 ] 
    
    
   pred.overall $ best_genotype  <-   sapply ( 1  :  nrow (tmp),  function (x)  names ( which.max (tmp[x,]))) 
    
    # get genotype colors  
   pred.overall $ best_genotype_col_Pt  <-  geno.info[pred.overall $ best_genotype,  'color_Pt' ] 
   pred.overall $ best_genotype_col_k3  <-  geno.info[pred.overall $ best_genotype,  'color_k3' ] 
    
   pred.overall $ best_genotype  <-   gsub ( 'genotype_' ,  '' , pred.overall $ best_genotype) 
    
    # quick plot to check  
    plot (pred.overall $ garden_clim,  col =  pred.overall $ best_genotype_col_Pt,  pch =   16 )    
   
       plot (pred.overall $ garden_clim,  col =  pred.overall $ best_genotype_col_k3,  pch =   16 )    
   
       # list 'best' genotype  
    cbind (pred.overall $ garden_clim, pred.overall $ best_genotype)    
  ##        [,1]                 [,2] 
##   [1,] "-23.9"              "405"
##   [2,] "-23.5252525252525"  "405"
##   [3,] "-23.150505050505"   "405"
##   [4,] "-22.7757575757576"  "405"
##   [5,] "-22.4010101010101"  "405"
##   [6,] "-22.0262626262626"  "405"
##   [7,] "-21.6515151515151"  "405"
##   [8,] "-21.2767676767677"  "405"
##   [9,] "-20.9020202020202"  "405"
##  [10,] "-20.5272727272727"  "405"
##  [11,] "-20.1525252525253"  "588"
##  [12,] "-19.7777777777778"  "588"
##  [13,] "-19.4030303030303"  "588"
##  [14,] "-19.0282828282828"  "411"
##  [15,] "-18.6535353535354"  "411"
##  [16,] "-18.2787878787879"  "411"
##  [17,] "-17.9040404040404"  "411"
##  [18,] "-17.5292929292929"  "411"
##  [19,] "-17.1545454545455"  "411"
##  [20,] "-16.779797979798"   "411"
##  [21,] "-16.4050505050505"  "411"
##  [22,] "-16.030303030303"   "411"
##  [23,] "-15.6555555555556"  "411"
##  [24,] "-15.2808080808081"  "588"
##  [25,] "-14.9060606060606"  "588"
##  [26,] "-14.5313131313131"  "588"
##  [27,] "-14.1565656565657"  "588"
##  [28,] "-13.7818181818182"  "588"
##  [29,] "-13.4070707070707"  "588"
##  [30,] "-13.0323232323232"  "545"
##  [31,] "-12.6575757575758"  "545"
##  [32,] "-12.2828282828283"  "545"
##  [33,] "-11.9080808080808"  "545"
##  [34,] "-11.5333333333333"  "545"
##  [35,] "-11.1585858585859"  "545"
##  [36,] "-10.7838383838384"  "545"
##  [37,] "-10.4090909090909"  "545"
##  [38,] "-10.0343434343434"  "218"
##  [39,] "-9.65959595959596"  "218"
##  [40,] "-9.28484848484849"  "218"
##  [41,] "-8.91010101010101"  "218"
##  [42,] "-8.53535353535354"  "218"
##  [43,] "-8.16060606060606"  "218"
##  [44,] "-7.78585858585859"  "311"
##  [45,] "-7.41111111111111"  "311"
##  [46,] "-7.03636363636364"  "311"
##  [47,] "-6.66161616161616"  "311"
##  [48,] "-6.28686868686869"  "311"
##  [49,] "-5.91212121212121"  "311"
##  [50,] "-5.53737373737374"  "311"
##  [51,] "-5.16262626262627"  "307"
##  [52,] "-4.78787878787879"  "307"
##  [53,] "-4.41313131313132"  "307"
##  [54,] "-4.03838383838384"  "307"
##  [55,] "-3.66363636363637"  "307"
##  [56,] "-3.28888888888889"  "307"
##  [57,] "-2.91414141414142"  "307"
##  [58,] "-2.53939393939394"  "307"
##  [59,] "-2.16464646464647"  "307"
##  [60,] "-1.78989898989899"  "307"
##  [61,] "-1.41515151515152"  "307"
##  [62,] "-1.04040404040405"  "307"
##  [63,] "-0.665656565656569" "307"
##  [64,] "-0.290909090909096" "307"
##  [65,] "0.0838383838383798" "307"
##  [66,] "0.458585858585856"  "307"
##  [67,] "0.833333333333329"  "307"
##  [68,] "1.2080808080808"    "307"
##  [69,] "1.58282828282828"   "311"
##  [70,] "1.95757575757575"   "311"
##  [71,] "2.33232323232323"   "311"
##  [72,] "2.7070707070707"    "311"
##  [73,] "3.08181818181818"   "311"
##  [74,] "3.45656565656565"   "311"
##  [75,] "3.83131313131313"   "311"
##  [76,] "4.2060606060606"    "311"
##  [77,] "4.58080808080808"   "311"
##  [78,] "4.95555555555555"   "311"
##  [79,] "5.33030303030302"   "311"
##  [80,] "5.7050505050505"    "311"
##  [81,] "6.07979797979797"   "311"
##  [82,] "6.45454545454545"   "311"
##  [83,] "6.82929292929292"   "311"
##  [84,] "7.2040404040404"    "311"
##  [85,] "7.57878787878787"   "311"
##  [86,] "7.95353535353535"   "311"
##  [87,] "8.32828282828282"   "311"
##  [88,] "8.7030303030303"    "311"
##  [89,] "9.07777777777777"   "311"
##  [90,] "9.45252525252525"   "311"
##  [91,] "9.82727272727272"   "311"
##  [92,] "10.2020202020202"   "311"
##  [93,] "10.5767676767677"   "311"
##  [94,] "10.9515151515151"   "311"
##  [95,] "11.3262626262626"   "311"
##  [96,] "11.7010101010101"   "405"
##  [97,] "12.0757575757576"   "405"
##  [98,] "12.450505050505"    "405"
##  [99,] "12.8252525252525"   "405"
## [100,] "13.2"               "405"  
       # save  
    # save(pred.overall, file = 'results/model_prediction/genotypePredictedHeights_byGardenMCMT.Rdata')  
    # write.csv(pred.overall, file = 'results/model_prediction/genotypePredictedHeights_byGardenMCMT.csv', row.names = F)     
 
 
  1.4  Load climate
rasters 
 Load future and historic MCMT raster files 
 ClimateNA raster files used here are available from DataBasin: 
  https://adaptwest.databasin.org/pages/adaptwest-climatena/  
 Species range shapefiles are from Little 1971 and are available from
DataBasin: 
 P. balsamifera:  https://databasin.org/datasets/91380e091ca048359a66fc65962ed210/  
 P. trichocarpa:  https://databasin.org/datasets/84e47784fe2a463c8b292007fb43f2cd/  
       # which climate variable is being used?   
   clim_var  <-   'MCMT'  
    # calculate 2-year average, used in model  
   dat $ garden_MCMT_2020_2021_avg  <-  (dat $ garden_MCMT_2020  +  dat $ garden_MCMT_2021) /  2  
   gard_clim_colname  <-   'garden_MCMT_2020_2021_avg'  
    
    # get historic and future climate  
    
   hist  <-   rast ( paste ( 'data/climate/climateNA/Normal_1961_1990/Normal_1961_1990_bioclim/Normal_1961_1990_' , clim_var,  '.tif' ,  sep =   '' )) 
    crs (hist,  proj =  T)    
  ## [1] "+proj=laea +lat_0=45 +lon_0=-100 +x_0=0 +y_0=0 +datum=WGS84 +units=m +no_defs"  
      hist91  <-   rast ( paste ( 'data/climate/climateNA/Normal_1991_2020/Normal_1991_2020_' , clim_var,  '.tif' ,  sep =   '' )) 
    crs (hist,  proj =  T)    
  ## [1] "+proj=laea +lat_0=45 +lon_0=-100 +x_0=0 +y_0=0 +datum=WGS84 +units=m +no_defs"  
      fut8  <-   rast ( paste ( 'data/climate/climateNA/future/ensemble_8GCMs_ssp245_2041_2070_bioclim/ensemble_8GCMs_ssp245_2041_2070_' , clim_var,  '.tif' ,  sep =   '' )) 
    
   fut13  <-   rast ( paste ( 'data/climate/climateNA/future/ensemble_13GCMs_ssp245_2041_2070_bioclim/ensemble_13GCMs_ssp245_2041_2070_' , clim_var,  '.tif' ,  sep =   '' )) 
    
    ################ convert coords  
    
    # convert coords to crs of climateNA  
   crs.cna  <-   crs (hist,  proj =  T) 
    
    # provenance coordinates  
   coords.prov  <-   st_as_sf (provs[, c ( "provenance_longitude" ,  "provenance_latitude" )],  coords =   c ( 1 , 2 ),  crs =   st_crs ( 4326 )) 
   coords.prov.cna  <-   st_transform (coords.prov $ geometry,  crs =  crs.cna) 
    
    # garden coordinates  
   coords.gards  <-   st_as_sf (gards[, c ( "Longitude" ,  "Latitude" )],  coords =   c ( 1 , 2 ),  crs =   st_crs ( 4326 )) 
   coords.gards.cna  <-   st_transform (coords.gards $ geometry,  crs =  crs.cna) 
    
    # load shapefile of state/province borders  
   borders  <-   read_sf ( 'data/shapefiles/NorthAmerica_PoliticalBoundaries_Shapefile/NA_PoliticalDivisions/data/bound_p/boundaries_p_2021_v3.shp' ) 
   borders  <-   st_transform (borders,  crs =  crs.cna) 
    # simplify to 1 km  
   borders  <-   st_simplify (borders,  dTolerance =   1000 ) 
    
    # species rangemaps  
    # shapefile  
   balsam  <-   st_read ( 'data/shapefiles/Pbal_shapefile/data/commondata/data0/popubals.shp' )    
  ## Reading layer `popubals' from data source 
##   `/home/alayna/Documents/research/projects/2023_populus_common_gardens/data/shapefiles/Pbal_shapefile/data/commondata/data0/popubals.shp' 
##   using driver `ESRI Shapefile'
## Simple feature collection with 415 features and 5 fields
## Geometry type: POLYGON
## Dimension:     XY
## Bounding box:  xmin: -18247640 ymin: 4662920 xmax: -5857014 ymax: 10826350
## Projected CRS: WGS 84 / Pseudo-Mercator  
      tricho  <-   st_read ( 'data/shapefiles/Ptri_shapefile/data/commondata/data0/poputric.shp' )    
  ## Reading layer `poputric' from data source 
##   `/home/alayna/Documents/research/projects/2023_populus_common_gardens/data/shapefiles/Ptri_shapefile/data/commondata/data0/poputric.shp' 
##   using driver `ESRI Shapefile'
## Simple feature collection with 450 features and 5 fields
## Geometry type: POLYGON
## Dimension:     XY
## Bounding box:  xmin: -17154180 ymin: 3609620 xmax: -11488690 ymax: 8896231
## Projected CRS: WGS 84 / Pseudo-Mercator  
       # convert CRS  
   balsam  <-   st_transform (balsam, crs.cna) 
   tricho  <-   st_transform (tricho, crs.cna) 
    
    # test plot  
    plot (hist) 
    
    plot (balsam $ geometry,  add =  T,  border =   'navy' ) 
    plot (tricho $ geometry,  add =  T,  border =   'grey50' ) 
    plot (borders $ geometry,  add =  T)    
   
 
 
 
  2  Mapping (color by
species ancestry) 
 
  2.1  Functions 
       # function for plotting  
   map_best_geno  <-   function (raster, raster_name,  save =  F, ...){ 
      
      if (save  ==  T){ 
        png ( file =   paste ( 'results/model_prediction/best_genotype_2yearModel_mapped_by_' , clim_var,  '_' , raster_name,  '.png' ,  sep =   '' ), 
       height =   8 ,  width =   8 ,  res =   300 ,  units =   'in' ) 
     } 
      
      
      plot (raster, 
           pax =   list ( side=  NA ), 
           colNA =   'white' , 
          ...) 
      
      plot (borders $ geometry,  add =  T,  lwd =   0.5 ) 
      plot (balsam $ geometry,  add =  T,  border =   'navy' ) 
      plot (tricho $ geometry,  add =  T,  border =   'darkgreen' ) 
      plot (coords.prov.cna,  add =  T,  lwd =   2 ,  bg =  geno.info $ color_Pt,  pch =   21 ,  cex =   1.5 ) 
      plot (coords.gards.cna,  add =  T,  pch =   17 ,  cex =   2 ) 
      
      # legend(-3e6, -1.8e6,   
      #        pch = c(22,22,21,24),  
      #        pt.cex = 2,  
      #        xjust = 0.5,  
      #        pt.bg =  c('darkgreen', 'navy', 'white','white'),   
      #        legend = c(substitute(paste(italic('P. trichocarpa'), ' range')),   
      #                   substitute(paste(italic('P. balsamifera'), ' range')),  
      #                   'Collection Site',  
      #                   'Common Garden Site'))  
      
        legend ( -  3e6 ,  -  1.8e6 ,  
             pch =   c ( 22 , 22 , 21 , 24 ), 
             pt.cex =   2 , 
             xjust =   0.5 , 
             col =    c ( 'darkgreen' ,  'navy' ,  'black' , 'black' ),  
             pt.lwd =   2 , 
             pt.bg =   c ( 'white' ,  'white' ,  'white' ,  'white' ), 
             legend =   c ( substitute ( paste ( italic ( 'P. trichocarpa' ),  ' range' )),  
                        substitute ( paste ( italic ( 'P. balsamifera' ),  ' range' )), 
                        'Collection Site' , 
                        'Common Garden Site' )) 
      
      
      
      # add inset legend for species ancestry gradient  
      # https://stackoverflow.com/questions/13355176/gradient-legend-in-base  
      
      # get breaks for legend labels from color palette  
     pal_range  <-   range ( attr (colf.pt,  'breaks' )) 
     pal_min  <-  pal_range[ 1 ] 
     pal_max  <-  pal_range[ 2 ] 
      
     legend_image  <-   as.raster ( matrix ( colf.pt ( seq (pal_max, pal_min,  length =   100 )),  ncol=  1 )) 
      
      # look at NDC coords  
      grconvertX ( seq ( -  4e6 ,  3e6 ,  1000000 ),  from =   'user' ,  to =   'ndc' ) 
      grconvertY ( seq ( -  3e6 ,  4e6 ,  1000000 ),  from =   'user' ,  to =   'ndc' ) 
      
     figSet  <-   c ( 0.1 ,  0.4 ,  0.15 ,  0.5 ) 
     op  <-   par (   ## set and store par  
        fig= figSet,     ## set figure region,   
        mar=  c ( 1 ,  1 ,  1 ,  9.5 ),                                   ## set margins  
        new=  TRUE )                                 ## set new for overplot w/ next plot  
      
      plot ( 0 , 0 ,  type=  'n' ,  axes= F,  xlab=  '' ,  ylab=  '' )   ## ini plot2  
      rasterImage (legend_image,  0 ,  0 ,  1 ,  1 )                        ## the gradient  
     lbsq  <-   seq.int ( 0 ,  1 ,  l=  5 )                                   ## seq. for labels  
      axis ( 4 ,  at= lbsq,  pos=  1 ,  labels= F,  col=  0 ,  col.ticks=  1 ,  tck=  - . 1 )   ## axis ticks  
      mtext (lbsq,  4 ,  0.3 ,  at= lbsq,  las=  2 ,  cex= . 8 )                     ## tick labels  
      
      mtext ( expression ( atop ( 'Best-performing  \n  genotype' ,  italic ( '(% P. trichocarpa)' ))),  side=  3 ,  line=  0.2 ,  cex=  1 ,  adj= . 1 )           ## legend title  
      
      par (op)   ## reset par  
      
      
      if (save  ==  T){ 
        dev.off () 
     } 
   } 
    
    ######################################  
    # inset zoomed to collection sites  
    
    
    # function  
   map_best_geno_crop  <-   function (raster, raster_name,  save =  F, ...){ 
      
      if (save  ==  T){ 
        png ( file =   paste ( 'results/model_prediction/best_genotype_2yearModel_mapped_by_' , clim_var,  '_inset_' , raster_name,  '.png' ,  sep =   '' ), 
       height =   8 ,  width =   6 ,  res =   300 ,  units =   'in' ) 
     } 
      
      
      plot (raster, 
           pax =   list ( side=  NA ), 
           colNA =   'white' , 
          ...) 
      
      plot (borders $ geometry,  add =  T,  lwd =   0.5 ) 
      plot (balsam $ geometry,  add =  T,  border =   'navy' ) 
      plot (tricho $ geometry,  add =  T,  border =   'darkgreen' ) 
      plot (coords.prov.cna,  add =  T,  lwd =   2 ,  bg =  geno.info $ color_Pt,  pch =   21 ,  cex =   2 ) 
      
      # legend(-3e6, -1.8e6,   
      #        pch = c(22,22,21,24),  
      #        pt.cex = 2,  
      #        xjust = 0.5,  
      #        pt.bg =  c('darkgreen', 'navy', 'white','white'),   
      #        legend = c(substitute(paste(italic('P. trichocarpa'), ' range')),   
      #                   substitute(paste(italic('P. balsamifera'), ' range')),  
      #                   'Collection Site',  
      #                   'Common Garden Site'))  
      
      if (save  ==  T){ 
        dev.off () 
     } 
      
   }    
 
 
  2.2  Plot 
       # color scale - used in legend  
   colf.pt  <-   colorRamp2 ( c ( 0 , 0.5 , 1 ),  colors =   c ( 'dodgerblue2' ,  'grey20' ,  'darkolivegreen2' )) 
    
    # assign color of 'optimal' genotype to each temperature bin using coltab   
   cols  <-   cbind (pred.overall $ garden_clim, pred.overall $ best_genotype_col_Pt) 
    head (cols)    
  ##      [,1]                [,2]       
## [1,] "-23.9"             "#3E4D6CFF"
## [2,] "-23.5252525252525" "#3E4D6CFF"
## [3,] "-23.150505050505"  "#3E4D6CFF"
## [4,] "-22.7757575757576" "#3E4D6CFF"
## [5,] "-22.4010101010101" "#3E4D6CFF"
## [6,] "-22.0262626262626" "#3E4D6CFF"  
       # set raster colors  
    coltab (hist)  <-  cols 
    coltab (hist91)  <-  cols 
    coltab (fut8)  <-  cols 
    coltab (fut13)  <-  cols 
    
    # make values outside prediction range (common gardens) NA  
    # get the range of actual garden climates that were tested  
   pred_clim  <-   c (dat $ garden_MCMT_2021, dat $ garden_MCMT_2022) 
    
   msk  <-   ifel (hist  >   max (pred_clim)  |  hist  <   min (pred_clim),  NA ,  1 ) 
   hist.mask  <-   mask (hist, msk) 
    plot (hist.mask,  colNA =   'grey' )    
   
      hist91.mask  <-   mask (hist91, msk) 
    plot (hist91.mask,  colNA =   'grey' )    
   
       # future climate  
    
    # make values outside predict range NA  
   msk  <-   ifel (fut13  >   max (pred_clim)  |  fut13  <   min (pred_clim),  NA ,  1 ) 
   fut13.mask  <-   mask (fut13, msk) 
    plot (fut13.mask,  colNA =   'grey' )    
   
      msk  <-   ifel (fut8  >   max (pred_clim)  |  fut8  <   min (pred_clim),  NA ,  1 ) 
   fut8.mask  <-   mask (fut8, msk) 
    plot (fut8.mask,  colNA =   'grey' )    
   
       # are we saving plots?  
   save  =   FALSE  
    
    # Maps!  
    
    # plot full range  
    map_best_geno (hist.mask,  raster_name =   'historic' ,  main =   '1961-1990' ,  save =  save)    
   
       map_best_geno (hist91.mask,  raster_name =   '1991-2020' ,  main =   '1991-2020' ,  save =  save)    
   
       map_best_geno (fut13.mask,  raster_name =   '2041-2070_13GCMs' ,  main =   '2041-2070 (13 GCM ensemble)' ,  save =  save)    
   
       map_best_geno (fut8.mask,  raster_name =   '2041-2070_8GCMs' ,  main =   '2041-2070 (8 GCM ensemble)' ,  save =  save)    
   
       # crop rasters, then plot  
   extent  <-   ext ( -  26e5 ,  -  5e5 ,  45e4 ,  3e6 ) 
   hist.crop  <-   crop (hist.mask, extent) 
   hist91.crop  <-   crop (hist91.mask, extent) 
   fut8.crop  <-   crop (fut8.mask, extent) 
   fut13.crop  <-   crop (fut13.mask, extent) 
    
    map_best_geno_crop (hist.crop,  main =   '1961-1990' ,  save =  save,  raster_name =   'historic' )    
   
       map_best_geno_crop (hist91.crop,  raster_name =   '1991-2020' ,  main =   '1991-2020' ,  save =  save)    
   
       map_best_geno_crop (fut8.crop,  main =   '2041-2070 (8 GCM ensemble)' ,  save =  save,  raster_name =   '2041-2070_8GCMs' )    
   
       map_best_geno_crop (fut13.crop,  main =   '2041-2070 (13 GCM ensemble)' ,  save =  save,  raster_name =   '2041-2070_13GCMs' )    
   
       # crop to western interior region  
    
   extent  <-   ext ( -  15e5 ,  1e5 ,  -  10e5 ,  1e6 ) 
   hist.crop  <-   crop (hist.mask, extent) 
   hist91.crop  <-   crop (hist91.mask, extent) 
   fut8.crop  <-   crop (fut8.mask, extent) 
   fut13.crop  <-   crop (fut13.mask, extent) 
    
    map_best_geno_crop (hist.crop,  main =   '1961-1990' ,  save =  save,  raster_name =   'west_historic' )    
   
       map_best_geno_crop (hist91.crop,  raster_name =   'west_1991-2020' ,  main =   '1991-2020' ,  save =  save)    
   
       map_best_geno_crop (fut8.crop,  main =   '2041-2070 (8 GCM ensemble)' ,  save =  save,  raster_name =   'west_2041-2070_8GCMs' )    
   
       map_best_geno_crop (fut13.crop,  main =   '2041-2070 (13 GCM ensemble)' ,  save =  save,  raster_name =   'west_2041-2070_13GCMs' )    
   
 
 
 
  3  Mapping (color by
K=3) 
 Here, color by ancestry based on admixture with K=3 (separating
tricho into a coastal and interior lineage). Colors for each genotype
are calculated as RGB values with blue = balsam, red = interior tricho,
and green = coastal tricho. 
 Did not use these in the manuscript because the tricho/balsam pattern
seems to be stronger, and genetic PC2, which separates coastal and
interior tricho, was not significant in the model of growth in the
common gardens. 
 
  3.1  Functions 
       # function for plotting  
    # comapred to function above, this just removes the ancestry scale  
    
   map_best_geno_k3  <-   function (raster, raster_name,  save =  F, ...){ 
      
      if (save  ==  T){ 
        png ( file =   paste ( 'results/model_prediction/best_genotype_2yearModel_colorK3_mapped_by_' , clim_var,  '_' , raster_name,  '.png' ,  sep =   '' ), 
       height =   8 ,  width =   8 ,  res =   300 ,  units =   'in' ) 
     } 
      
      
      plot (raster, 
           pax =   list ( side=  NA ), 
           colNA =   'white' , 
          ...) 
      
      plot (borders $ geometry,  add =  T,  lwd =   0.5 ) 
      plot (balsam $ geometry,  add =  T,  border =   'navy' ) 
      plot (tricho $ geometry,  add =  T,  border =   'darkgreen' ) 
      plot (coords.prov.cna,  add =  T,  lwd =   2 ,  bg =  geno.info $ color_k3,  pch =   21 ,  cex =   1.5 ) 
      plot (coords.gards.cna,  add =  T,  pch =   17 ,  cex =   2 ) 
      
      legend ( -  3e6 ,  -  1.8e6 ,  
             pch =   c ( 22 , 22 , 21 , 24 ), 
             pt.cex =   2 , 
             xjust =   0.5 , 
             pt.bg =    c ( 'darkgreen' ,  'navy' ,  'white' , 'white' ),  
             legend =   c ( substitute ( paste ( italic ( 'P. trichocarpa' ),  ' range' )),  
                        substitute ( paste ( italic ( 'P. balsamifera' ),  ' range' )), 
                        'Collection Site' , 
                        'Common Garden Site' )) 
      
      
      if (save  ==  T){ 
        dev.off () 
     } 
   } 
    
    
    # inset / zoomed  
    
   map_best_geno_crop_k3  <-   function (raster, raster_name,  save =  F, ...){ 
      
      if (save  ==  T){ 
        png ( file =   paste ( 'results/model_prediction/best_genotype_2yrModel_colorK3_mapped_by_' , clim_var,  '_inset_' , raster_name,  '.png' ,  sep =   '' ), 
       height =   8 ,  width =   6 ,  res =   300 ,  units =   'in' ) 
     } 
      
      
      plot (raster, 
           pax =   list ( side=  NA ), 
           colNA =   'white' , 
          ...) 
      
      plot (borders $ geometry,  add =  T,  lwd =   0.5 ) 
      plot (balsam $ geometry,  add =  T,  border =   'navy' ) 
      plot (tricho $ geometry,  add =  T,  border =   'darkgreen' ) 
      plot (coords.prov.cna,  add =  T,  lwd =   2 ,  bg =  geno.info $ color_k3,  pch =   21 ,  cex =   2 ) 
      
      
      if (save  ==  T){ 
        dev.off () 
     } 
   }    
 
 
  3.2  Plot 
       # color by K=3  
   cols  <-   cbind (pred.overall $ garden_clim, pred.overall $ best_genotype_col_k3) 
    
    # set raster colors  
    coltab (hist.mask)  <-  cols 
    coltab (hist91.mask)  <-  cols 
    coltab (fut8.mask)  <-  cols 
    coltab (fut13.mask)  <-  cols 
    
    # are we saving plots?  
   save  =   FALSE  
    
    # map  
    
    map_best_geno_k3 (hist.mask,  raster_name =   'historic' ,  main =   'Historic (1961-1990)' ,  save =  save)    
   
       map_best_geno_k3 (hist91.mask,  raster_name =   '1991-2020' ,  main =   '1991-2020' ,  save =  save)    
   
       map_best_geno_k3 (fut8.mask,  raster_name =   '2041-2070_8GCMs' ,  main =   '2041-2070 (8 GCM ensemble)' ,  save =  save)    
   
       map_best_geno_k3 (fut13.mask,  raster_name =   '2041-2070_13GCMs' ,  main =   '2041-2070 (13 GCM ensemble)' ,  save =  save)    
   
       # crop rasters, then plot  
   extent  <-   ext ( -  26e5 ,  -  5e5 ,  45e4 ,  3e6 ) 
   hist.crop  <-   crop (hist.mask, extent) 
   hist91.crop  <-   crop (hist91.mask, extent) 
   fut8.crop  <-   crop (fut8.mask, extent) 
   fut13.crop  <-   crop (fut13.mask, extent) 
    
    map_best_geno_crop_k3 (hist.crop,  main =   '1961-1990' ,  save =  save,  raster_name =   'historic' )    
   
       map_best_geno_crop_k3 (hist91.crop,  raster_name =   '1991-2020' ,  main =   '1991-2020' ,  save =  save)    
   
       map_best_geno_crop_k3 (fut8.crop,  main =   '2041-2070 (8 GCM ensemble)' ,  save =  save,  raster_name =   '2041-2070_8GCMs' )    
   
       map_best_geno_crop_k3 (fut13.crop,  main =   '2041-2070 (13 GCM ensemble)' ,  save =  save,  raster_name =   '2041-2070_13GCMs' )    
   
       # crop to western interior region  
    
   extent  <-   ext ( -  15e5 ,  1e5 ,  -  10e5 ,  1e6 ) 
   hist.crop  <-   crop (hist.mask, extent) 
   hist91.crop  <-   crop (hist91.mask, extent) 
   fut8.crop  <-   crop (fut8.mask, extent) 
   fut13.crop  <-   crop (fut13.mask, extent) 
    
    map_best_geno_crop_k3 (hist.crop,  main =   '1961-1990' ,  save =  save,  raster_name =   'west_historic' )    
   
       map_best_geno_crop_k3 (hist91.crop,  raster_name =   'west_1991-2020' ,  main =   '1991-2020' ,  save =  save)    
   
       map_best_geno_crop_k3 (fut8.crop,  main =   '2041-2070 (8 GCM ensemble)' ,  save =  save,  raster_name =   'west_2041-2070_8GCMs' )    
   
       map_best_geno_crop_k3 (fut13.crop,  main =   '2041-2070 (13 GCM ensemble)' ,  save =  save,  raster_name =   'west_2041-2070_13GCMs' )    
   
 
 


 

 

 

 

 


 
 

 
 
