# Supplementary material for: Variation in responses to temperature across admixed genotypes of Populus trichocarpa × P. balsamifera predict geographic shifts in regions where hybrids are favored
Source: bioRxiv. 2025 May 22:2025.05.16.654548. Preprint. [Version 1] doi: 10.1101/2025.05.16.654548 (PMC12139819; doi:10.1101/2025.05.16.654548)
Supplement: Supplement 3 [file media-3.gz › predict_growth_future_climates.html]

 

 

 

 
 
 


 

 

 Predict growth changes under future climate 

 
 
 
 
 
 
 
 
 

 


 
 
 


 


 

 

 


 

 


 


 


 Predict growth changes under future
climate 
 Alayna Mead 
 2025-05-12 

 

 
 
   1  Setup 
 
   1.1  Load libraries and data  
   1.2  Back-transform input data  
   1.3  Extract genotype info  
  
   2  Get future climates for each
genotype 
 
   2.1  Load climate data  
   2.2  Extract future climates for each
genotype’s home site  
  
   3  Predict height at future
climates 
 
   3.1  Predict functions  
   3.2  Predict response to historic and
future climate for each genotype  
   3.3  Calculate change in fitness under
future climates  
  
   4  Map predictions for each collected
genotype/site  
   5  Map rangewide predictions for each
genotype 
 
   5.1  Map past and future fitness
predictions and change for each genotype  
  
 
 

 Using the genotype-specific modeled reaction norms produced by
‘transfer_function_multiyear_linear_mixed_effects_model.Rmd’ script,
predict how each genotype’s growth and survival could change under
future temperatures (represented by MCMT). 
 
  1  Setup 
 
  1.1  Load libraries and
data 
       library (ggplot2) 
    library (glmmTMB)  # model  
    library (sf)  # mapping     
  ## Linking to GEOS 3.13.1, GDAL 3.10.2, PROJ 9.6.0; sf_use_s2() is TRUE  
       library (terra)  # climate rasters     
  ## terra 1.8.42  
       library (RColorBrewer)  # colors  
    library (circlize)  # colorRamp2     
  ## ========================================
## circlize version 0.4.16
## CRAN page: https://cran.r-project.org/package=circlize
## Github page: https://github.com/jokergoo/circlize
## Documentation: https://jokergoo.github.io/circlize_book/book/
## 
## If you use it in published research, please cite:
## Gu, Z. circlize implements and enhances circular visualization
##   in R. Bioinformatics 2014.
## 
## This message can be suppressed by:
##   suppressPackageStartupMessages(library(circlize))
## ========================================  
       # load garden data - named 'dat'  
    # remove the 2024 data  
    load ( 'data/clean/mini_garden_phenotypic_and_climate_data_2021-2024.Rdata' ) 
   dat  <-  dat[,  !  endsWith ( colnames (dat),  '2024' )] 
    
    # load model info  
    # mod: model  
    # df.scaled: scaled mini garden data that went into the model  
    # scaling_factor: used to scale variables before fitting model  
    load ( 'results/model_prediction/glmTMB_multiyear_model_outputs_garden_MCMT_2021-2022_vs_GrowthIncrement_2021-2022.Rda' ) 
    
    # load predicted heights for each genotype - named 'predictions'  
    load ( 'results/model_prediction/predictedGrowth_acrossClimates_byGenotype_garden_MCMT_2021-2022.Rdata' ) 
   preds  <-  predictions 
    rm (predictions) 
    
    sessionInfo ()    
  ## R version 4.5.0 (2025-04-11)
## Platform: x86_64-pc-linux-gnu
## Running under: Arch Linux
## 
## Matrix products: default
## BLAS:   /usr/lib/libblas.so.3.12.0 
## LAPACK: /usr/lib/liblapack.so.3.12.0  LAPACK version 3.12.0
## 
## locale:
##  [1] LC_CTYPE=en_US.UTF-8       LC_NUMERIC=C              
##  [3] LC_TIME=en_US.UTF-8        LC_COLLATE=en_US.UTF-8    
##  [5] LC_MONETARY=en_US.UTF-8    LC_MESSAGES=en_US.UTF-8   
##  [7] LC_PAPER=en_US.UTF-8       LC_NAME=C                 
##  [9] LC_ADDRESS=C               LC_TELEPHONE=C            
## [11] LC_MEASUREMENT=en_US.UTF-8 LC_IDENTIFICATION=C       
## 
## time zone: US/Eastern
## tzcode source: system (glibc)
## 
## attached base packages:
## [1] stats     graphics  grDevices datasets  utils     methods   base     
## 
## other attached packages:
## [1] circlize_0.4.16    RColorBrewer_1.1-3 terra_1.8-42       sf_1.0-20         
## [5] glmmTMB_1.1.11     ggplot2_3.5.2     
## 
## loaded via a namespace (and not attached):
##  [1] sass_0.4.10         generics_0.1.3      renv_0.17.3        
##  [4] class_7.3-23        shape_1.4.6.1       KernSmooth_2.23-26 
##  [7] lattice_0.22-6      lme4_1.1-37         digest_0.6.37      
## [10] magrittr_2.0.3      evaluate_1.0.3      grid_4.5.0         
## [13] fastmap_1.2.0       jsonlite_2.0.0      Matrix_1.7-3       
## [16] e1071_1.7-16        DBI_1.2.3           GlobalOptions_0.1.2
## [19] mgcv_1.9-1          scales_1.3.0        codetools_0.2-20   
## [22] numDeriv_2016.8-1.1 jquerylib_0.1.4     reformulas_0.4.0   
## [25] Rdpack_2.6.4        cli_3.6.4           rlang_1.1.6        
## [28] units_0.8-7         rbibutils_2.3       munsell_0.5.1      
## [31] splines_4.5.0       withr_3.0.2         cachem_1.1.0       
## [34] yaml_2.3.10         tools_4.5.0         nloptr_2.2.1       
## [37] minqa_1.2.8         dplyr_1.1.4         colorspace_2.1-1   
## [40] boot_1.3-31         vctrs_0.6.5         R6_2.6.1           
## [43] proxy_0.4-27        lifecycle_1.0.4     classInt_0.4-11    
## [46] MASS_7.3-65         pkgconfig_2.0.3     pillar_1.10.2      
## [49] bslib_0.9.0         gtable_0.3.6        glue_1.8.0         
## [52] Rcpp_1.0.14         xfun_0.52           tibble_3.2.1       
## [55] tidyselect_1.2.1    knitr_1.50          htmltools_0.5.8.1  
## [58] nlme_3.1-168        rmarkdown_2.29      TMB_1.9.17         
## [61] compiler_4.5.0  
      knitr :: opts_chunk $  set ( fig.width =   8 ,  fig.height =   10 )    
 
 
  1.2  Back-transform input
data 
 Data used for input to model was scaled - back-transform it to the
actual values. 
      df  <-  df.scaled 
    
    for (n  in   1  :  length (scaling_factor)){ 
      
     column  <-   names (scaling_factor)[n] 
      
     df[,column]  <-  df.scaled[,column] / scaling_factor[[n]] 
      
   } 
    
    head (df)    
  ##                      pheno genotype garden  block           indiv year
## NDSU.405.1.1.24_2022     0      405   NDSU NDSU.1 NDSU.405.1.1.24 2022
## NDSU.411.2.4.3_2022      0      411   NDSU NDSU.2  NDSU.411.2.4.3 2022
## NDSU.419.1.1.20_2022     0      419   NDSU NDSU.1 NDSU.419.1.1.20 2022
## NDSU.419.2.3.8_2022      0      419   NDSU NDSU.2  NDSU.419.2.3.8 2022
## NDSU.416.1.2.3_2022      0      416   NDSU NDSU.1  NDSU.416.1.2.3 2022
## NDSU.590.1.2.11_2022     0      590   NDSU NDSU.1 NDSU.590.1.2.11 2022
##                      garden_clim garden_clim_2 home_clim home_clim_2       Pt
## NDSU.405.1.1.24_2022       -16.5        272.25     -23.9      571.21 0.334682
## NDSU.411.2.4.3_2022        -16.5        272.25     -20.0      400.00 0.293048
## NDSU.419.1.1.20_2022       -16.5        272.25     -16.7      278.89 0.324367
## NDSU.419.2.3.8_2022        -16.5        272.25     -16.7      278.89 0.324367
## NDSU.416.1.2.3_2022        -16.5        272.25     -16.4      268.96 0.315421
## NDSU.590.1.2.11_2022       -16.5        272.25     -14.6      213.16       NA
##                             pc1        pc2        pc3         pc4        pc5
## NDSU.405.1.1.24_2022 0.02748668 0.02411587 0.03816905 0.000560667 0.03818182
## NDSU.411.2.4.3_2022  0.03132797 0.02236233 0.03757786 0.012868191 0.04275311
## NDSU.419.1.1.20_2022 0.02841758 0.02582109 0.02883672 0.008416795 0.02996169
## NDSU.419.2.3.8_2022  0.02841758 0.02582109 0.02883672 0.008416795 0.02996169
## NDSU.416.1.2.3_2022  0.03009858 0.02374161 0.03372735 0.012894037 0.04524013
## NDSU.590.1.2.11_2022         NA         NA         NA          NA         NA  
 
 
  1.3  Extract genotype
info 
 Get genetic and climate data for each genotype 
       # get info for each genotype to use for extracting climate data and predicting height under future climates  
    
    # columns to get - these will be the same for each replicate of a genotype  
    dput ( colnames (dat))    
  ## c("Unique_ID", "MiniCG_Site", "Genotype", "Plant_ID", "Pb", "Pt", 
## "transect", "provenance_latitude", "provenance_longitude", "provenance_elevation_m", 
## "block", "row", "column", "Survival_09_2021", "PreFlush_Height_cm_2021", 
## "PostSet_Height_cm_2021", "GrowthIncrement_2021", "PetioleColor_2021", 
## "RustDisease_2021", "DOY_Stage2_2021", "DOY_Stage3_2021", "DOY_Stage6_2021", 
## "Stage2_cGDD_2021", "Stage3_cGDD_2021", "MAT_TransferDist", "MAP_TransferDist", 
## "MiniCG_Site_2022", "Unique_ID_2022", "Genotype_2022", "PLANT_ID_2022", 
## "Survival_09_2022", "PreFlush_Height_cm_2022", "PostSet_Height_cm_2022", 
## "GrowthIncrement_2022", "RustDisease_2022", "DOY_Stage2_2022", 
## "DOY_Stage3_2022", "DOY_Stage6_2022", "Stage2_cGDD_2022", "Stage3_cGDD_2022", 
## "Unique_ID_2023", "MiniCG_Site_2023", "Genotype_2023", "block_2023", 
## "row_2023", "column_2023", "DOY_Stage2.DD.MM.YY_2023", "DOY_Stage3.DD.MM.YY_2023", 
## "DOY_Stage6.DD.MM.YY_2023", "DOY_Stage7.DD.MM.YY_2023", "DOY_Stage8.DD.MM.YY_2023", 
## "Survival_09_2023", "PreFlush_Height_cm_2023", "PostSet_Height_cm_2023", 
## "GrowthIncrement_2023", "RustDisease.1.or.0_2023", "DOY_Stage2_2023", 
## "DOY_Stage3_2023", "DOY_Stage6_2023", "DOY_Stage7_2023", "DOY_Stage8_2023", 
## "Notes_2023", "DOY_leaf_measurements.DD.MM.YY_2023", "DOY_leaf_measurements_2023", 
## "leaf_measurement_growth_stage_2023", "leaf_thickness_1_mm_2023", 
## "leaf_thickness_2_mm_2023", "leaf_thickness_3_mm_2023", "leaf_thickness_4_mm_2023", 
## "leaf_thickness_5_mm_2023", "leaf_thickness_avg_mm_2023", "leaf_thickness_sd_mm_2023", 
## "DOY_LICOR_measurement.DD.MM.YY_2023", "DOY_LICOR_measurement_2023", 
## "weather_LICOR_measurement_2023", "LICOR_codes_2023", "LICOR_notes_2023", 
## "Survival_09_2021_2023", "notes_2023", "leaf_mass_g_2023", "leaf_area_cm2_2023", 
## "LMA_g_m2_2023", "DOY_Stage2.DD.MM.YY_2021", "DOY_Stage3.DD.MM.YY_2021", 
## "DOY_Stage6.DD.MM.YY_2021", "DOY_Stage2.DD.MM.YY_2022", "DOY_Stage3.DD.MM.YY_2022", 
## "DOY_Stage6.DD.MM.YY_2022", "total_growth_increment_2021_2022", 
## "total_growth_increment_2021_2023", "rgr_2021", "rgr_2022", "rgr_2023", 
## "DOY_last_budset_2023", "stage7_presence_2023", "growing_season_days_2021", 
## "growing_season_days_2022", "growing_season_days_2023", "garden_Arboreta.University.Partner", 
## "garden_City", "garden_State", "garden_Latitude", "garden_Longitude", 
## "garden_Elevation_m", "garden_MAT_1961_1990", "garden_MWMT_1961_1990", 
## "garden_MCMT_1961_1990", "garden_TD_1961_1990", "garden_MAP_1961_1990", 
## "garden_MSP_1961_1990", "garden_AHM_1961_1990", "garden_SHM_1961_1990", 
## "garden_DD_0_1961_1990", "garden_DD5_1961_1990", "garden_DD_18_1961_1990", 
## "garden_DD18_1961_1990", "garden_NFFD_1961_1990", "garden_bFFP_1961_1990", 
## "garden_eFFP_1961_1990", "garden_FFP_1961_1990", "garden_PAS_1961_1990", 
## "garden_EMT_1961_1990", "garden_EXT_1961_1990", "garden_Eref_1961_1990", 
## "garden_CMD_1961_1990", "garden_MAR_1961_1990", "garden_RH_1961_1990", 
## "garden_CMI_1961_1990", "garden_DD1040_1961_1990", "garden_MAT_2020", 
## "garden_MWMT_2020", "garden_MCMT_2020", "garden_TD_2020", "garden_MAP_2020", 
## "garden_MSP_2020", "garden_AHM_2020", "garden_SHM_2020", "garden_DD_0_2020", 
## "garden_DD5_2020", "garden_DD_18_2020", "garden_DD18_2020", "garden_NFFD_2020", 
## "garden_bFFP_2020", "garden_eFFP_2020", "garden_FFP_2020", "garden_PAS_2020", 
## "garden_EMT_2020", "garden_EXT_2020", "garden_Eref_2020", "garden_CMD_2020", 
## "garden_MAR_2020", "garden_RH_2020", "garden_CMI_2020", "garden_DD1040_2020", 
## "garden_MAT_2021", "garden_MWMT_2021", "garden_MCMT_2021", "garden_TD_2021", 
## "garden_MAP_2021", "garden_MSP_2021", "garden_AHM_2021", "garden_SHM_2021", 
## "garden_DD_0_2021", "garden_DD5_2021", "garden_DD_18_2021", "garden_DD18_2021", 
## "garden_NFFD_2021", "garden_bFFP_2021", "garden_eFFP_2021", "garden_FFP_2021", 
## "garden_PAS_2021", "garden_EMT_2021", "garden_EXT_2021", "garden_Eref_2021", 
## "garden_CMD_2021", "garden_MAR_2021", "garden_RH_2021", "garden_CMI_2021", 
## "garden_DD1040_2021", "garden_MAT_2022", "garden_MWMT_2022", 
## "garden_MCMT_2022", "garden_TD_2022", "garden_MAP_2022", "garden_MSP_2022", 
## "garden_AHM_2022", "garden_SHM_2022", "garden_DD_0_2022", "garden_DD5_2022", 
## "garden_DD_18_2022", "garden_DD18_2022", "garden_NFFD_2022", 
## "garden_bFFP_2022", "garden_eFFP_2022", "garden_FFP_2022", "garden_PAS_2022", 
## "garden_EMT_2022", "garden_EXT_2022", "garden_Eref_2022", "garden_CMD_2022", 
## "garden_MAR_2022", "garden_RH_2022", "garden_CMI_2022", "garden_DD1040_2022", 
## "garden_MAT_2023", "garden_MWMT_2023", "garden_MCMT_2023", "garden_TD_2023", 
## "garden_MAP_2023", "garden_MSP_2023", "garden_AHM_2023", "garden_SHM_2023", 
## "garden_DD_0_2023", "garden_DD5_2023", "garden_DD_18_2023", "garden_DD18_2023", 
## "garden_NFFD_2023", "garden_bFFP_2023", "garden_eFFP_2023", "garden_FFP_2023", 
## "garden_PAS_2023", "garden_EMT_2023", "garden_EXT_2023", "garden_Eref_2023", 
## "garden_CMD_2023", "garden_MAR_2023", "garden_RH_2023", "garden_CMI_2023", 
## "garden_DD1040_2023", "licor_Unique_ID", "licor_obs", "licor_gsw", 
## "licor_gbw", "licor_gtw", "licor_E_apparent", "licor_VPcham", 
## "licor_VPref", "licor_VPleaf", "licor_VPDleaf", "licor_H2O_r", 
## "licor_H2O_s", "licor_H2O_leaf", "licor_Fs", "licor_Fm.", "licor_PhiPS2", 
## "licor_PS2.1", "licor_abs", "licor_ETR", "licor_rh_s", "licor_rh_r", 
## "licor_Tref", "licor_Tleaf", "licor_P_atm", "licor_Qamb", "licor_doy", 
## "licor_pitch", "licor_roll", "licor_heading", "licor_angle_inc_leaf", 
## "licor_direct_pct", "licor_slope_leaf", "licor_az_leaf", "licor_dec_solar", 
## "licor_az_solar", "licor_zenith_solar", "licor_latitude", "licor_longitude", 
## "licor_altitude", "licor_gps_sats", "licor_MiniCG_Site_first_measure", 
## "licor_Time_first_measure", "licor_Date_first_measure", "licor_lciSerNum_first_measure", 
## "licor_remark_first_measure", "licor_gps_time_first_measure", 
## "licor_gps_date_first_measure", "licor_Notes_first_measure", 
## "licor_Bud.stage_first_measure", "licor_Shade._first_measure", 
## "licor_Weather.notes_first_measure", "lower_stomata_presence", 
## "lower_fiveOrMoreStomata", "lower_stomata_count", "lower_stomata_pore_length_mean_um", 
## "lower_stomata_pore_length_sd_um", "upper_stomata_presence", 
## "upper_fiveOrMoreStomata", "upper_stomata_count", "upper_stomata_pore_length_mean_um", 
## "upper_stomata_pore_length_sd_um", "upper_stomata_density_mm2", 
## "lower_stomata_density_mm2", "stomata_ratio", "provenance_MAT", 
## "provenance_MWMT", "provenance_MCMT", "provenance_TD", "provenance_MAP", 
## "provenance_MSP", "provenance_AHM", "provenance_SHM", "provenance_DD_0", 
## "provenance_DD5", "provenance_DD_18", "provenance_DD18", "provenance_NFFD", 
## "provenance_bFFP", "provenance_eFFP", "provenance_FFP", "provenance_PAS", 
## "provenance_EMT", "provenance_EXT", "provenance_MAR", "provenance_Eref", 
## "provenance_CMD", "provenance_RH", "ID", "k2_balsam", "k2_tricho", 
## "k3_balsam", "k3_coastal_tricho", "k3_interior_tricho", "genetic_PC1", 
## "genetic_PC2", "genetic_PC3", "genetic_PC4", "genetic_PC5", "genetic_PC6", 
## "interspecific_heterozygosity", "hybrid_index_Pb", "heterozygosity", 
## "plastid_ID", "garden_climate_1961_1990_PC1", "garden_climate_1961_1990_PC2", 
## "garden_climate_1961_1990_PC3", "garden_climate_1961_1990_PC4", 
## "garden_climate_1961_1990_PC5", "garden_climate_1961_1990_PC6", 
## "garden_climate_1961_1990_PC7", "garden_climate_1961_1990_PC8", 
## "garden_climate_1961_1990_PC9", "garden_climate_2020_PC1", "garden_climate_2020_PC2", 
## "garden_climate_2020_PC3", "garden_climate_2020_PC4", "garden_climate_2020_PC5", 
## "garden_climate_2020_PC6", "garden_climate_2020_PC7", "garden_climate_2020_PC8", 
## "garden_climate_2020_PC9", "garden_climate_2021_PC1", "garden_climate_2021_PC2", 
## "garden_climate_2021_PC3", "garden_climate_2021_PC4", "garden_climate_2021_PC5", 
## "garden_climate_2021_PC6", "garden_climate_2021_PC7", "garden_climate_2021_PC8", 
## "garden_climate_2021_PC9", "garden_climate_2022_PC1", "garden_climate_2022_PC2", 
## "garden_climate_2022_PC3", "garden_climate_2022_PC4", "garden_climate_2022_PC5", 
## "garden_climate_2022_PC6", "garden_climate_2022_PC7", "garden_climate_2022_PC8", 
## "garden_climate_2022_PC9", "garden_climate_2023_PC1", "garden_climate_2023_PC2", 
## "garden_climate_2023_PC3", "garden_climate_2023_PC4", "garden_climate_2023_PC5", 
## "garden_climate_2023_PC6", "garden_climate_2023_PC7", "garden_climate_2023_PC8", 
## "garden_climate_2023_PC9", "provenance_climate_PC1", "provenance_climate_PC2", 
## "provenance_climate_PC3", "provenance_climate_PC4", "provenance_climate_PC5", 
## "provenance_climate_PC6", "provenance_climate_PC7", "provenance_climate_PC8", 
## "provenance_climate_PC9", "color_Pt", "color_k3")  
      columns  <-   c ( "Genotype" ,  "Plant_ID" ,  "Pb" ,  "Pt" ,  "transect" ,  "provenance_latitude" ,  "provenance_longitude" ,  "provenance_elevation_m" ,  "provenance_TD" ,  "provenance_MCMT" ,  "genetic_PC1" ,  "genetic_PC2" ,  "genetic_PC3" ,  "genetic_PC4" ,  "genetic_PC5" ,  "plastid_ID" ,  "color_Pt" ,  "color_k3" ) 
    
    # get genotypes  
   genos  <-   unique (dat $ Genotype) 
   genos  <-  genos[ !   is.na (genos)] 
    
    # get the rows that have the first match of each genotype  
   rows  <-   match (genos, dat $ Genotype) 
    
    # get subset of dataframe with these rows and the info we want for each genotype  
   geno.info  <-  dat[rows, columns] 
    rownames (geno.info)  <-  geno.info $ Genotype 
    
    # remove the ones with missing genetic info  
   geno.info  <-  geno.info[ !   is.na (geno.info $ genetic_PC1),] 
    dim (geno.info)    
  ## [1] 44 18  
       str (geno.info)    
  ## 'data.frame':    44 obs. of  18 variables:
##  $ Genotype              : Factor w/ 47 levels "206","210","218",..: 1 2 3 4 5 6 7 8 9 10 ...
##  $ Plant_ID              : chr  "206" "210" "218" "233" ...
##  $ Pb                    : num  0.4289 0.0916 0.1356 0.0342 0.2851 ...
##  $ Pt                    : num  0.571 0.908 0.864 0.966 0.715 ...
##  $ transect              : Factor w/ 5 levels "Alaska","Cassiar",..: 3 3 3 3 3 3 5 5 5 5 ...
##  $ provenance_latitude   : num  51.9 52.1 52 53.3 55.5 ...
##  $ provenance_longitude  : num  -124 -123 -122 -122 -123 ...
##  $ provenance_elevation_m: num  1064 744 381 677 816 ...
##  $ provenance_TD         : num  22.6 25.5 26.4 24.7 25.7 27.6 19.9 23.4 24.6 23.3 ...
##  $ provenance_MCMT       : num  -9.6 -9.9 -8.9 -9.3 -12.2 -12.4 -6.9 -5.8 -5.8 -4.8 ...
##  $ genetic_PC1           : num  0.00423 -0.03207 -0.02393 -0.03496 -0.00995 ...
##  $ genetic_PC2           : num  0.02407 -0.00722 0.01811 0.039 0.03374 ...
##  $ genetic_PC3           : num  -0.0243 -0.0365 -0.0505 -0.0418 -0.0256 ...
##  $ genetic_PC4           : num  0.0378 0.0122 0.046 0.0279 0.0399 ...
##  $ genetic_PC5           : num  0.00732 0.02208 0.01169 -0.02046 -0.00303 ...
##  $ plastid_ID            : chr  "balsamifera" "trichocarpa" "balsamifera" "balsamifera" ...
##  $ color_Pt              : chr  "#464B3CFF" "#A0C65FFF" "#94B55AFF" "#AFDB64FF" ...
##  $ color_k3              : chr  "#1B8163" "#836913" "#4E941D" "#39C600" ...  
 
 
 
  2  Get future climates for
each genotype 
 
  2.1  Load climate
data 
 Load future and historic MCMT raster files 
 ClimateNA raster files used here are available from DataBasin: 
  https://adaptwest.databasin.org/pages/adaptwest-climatena/  
 Species range shapefiles are from Little 1971 and are available from
DataBasin: 
 P. balsamifera:  https://databasin.org/datasets/91380e091ca048359a66fc65962ed210/  
 P. trichocarpa:  https://databasin.org/datasets/84e47784fe2a463c8b292007fb43f2cd/  
       # which climate variable is being used?   
    # can change this to use a different variable  
   clim_var  <-   'MCMT'  
    
    # get historic and future climate  
    
   hist  <-   rast ( paste ( 'data/climate/climateNA/Normal_1961_1990/Normal_1961_1990_bioclim/Normal_1961_1990_' , clim_var,  '.tif' ,  sep =   '' )) 
    crs (hist,  proj =  T)    
  ## [1] "+proj=laea +lat_0=45 +lon_0=-100 +x_0=0 +y_0=0 +datum=WGS84 +units=m +no_defs"  
      hist91  <-   rast ( paste ( 'data/climate/climateNA/Normal_1991_2020/Normal_1991_2020_' , clim_var,  '.tif' ,  sep =   '' )) 
    crs (hist,  proj =  T)    
  ## [1] "+proj=laea +lat_0=45 +lon_0=-100 +x_0=0 +y_0=0 +datum=WGS84 +units=m +no_defs"  
       # climateNA provides future ensemble models built from 8 GCMs and 13 GCMs - test both  
    
   fut8  <-   rast ( paste ( 'data/climate/climateNA/future/ensemble_8GCMs_ssp245_2041_2070_bioclim/ensemble_8GCMs_ssp245_2041_2070_' , clim_var,  '.tif' ,  sep =   '' )) 
    
   fut13  <-   rast ( paste ( 'data/climate/climateNA/future/ensemble_13GCMs_ssp245_2041_2070_bioclim/ensemble_13GCMs_ssp245_2041_2070_' , clim_var,  '.tif' ,  sep =   '' )) 
    
    ################ convert coords  
    
    # convert coordinates of collection sites to the CRS used by climateNA  
   crs.cna  <-   crs (hist,  proj =  T) 
    
    # provenance coordinates  
   coords.prov  <-   st_as_sf (geno.info[, c ( "provenance_longitude" ,  "provenance_latitude" )],  coords =   c ( 1 , 2 ),  crs =   st_crs ( 4326 )) 
   coords.prov.cna  <-   st_transform (coords.prov $ geometry,  crs =  crs.cna) 
    
    # load shapefile of state/province borders  
   borders  <-   read_sf ( 'data/shapefiles/NorthAmerica_PoliticalBoundaries_Shapefile/NA_PoliticalDivisions/data/bound_p/boundaries_p_2021_v3.shp' ) 
   borders  <-   st_transform (borders,  crs =  crs.cna) 
    # simplify to 1 km  
   borders  <-   st_simplify (borders,  dTolerance =   1000 ) 
    
    # species rangemaps  
    # shapefile  
   balsam  <-   st_read ( 'data/shapefiles/Pbal_shapefile/data/commondata/data0/popubals.shp' )    
  ## Reading layer `popubals' from data source 
##   `/home/alayna/Documents/research/projects/2023_populus_common_gardens/data/shapefiles/Pbal_shapefile/data/commondata/data0/popubals.shp' 
##   using driver `ESRI Shapefile'
## Simple feature collection with 415 features and 5 fields
## Geometry type: POLYGON
## Dimension:     XY
## Bounding box:  xmin: -18247640 ymin: 4662920 xmax: -5857014 ymax: 10826350
## Projected CRS: WGS 84 / Pseudo-Mercator  
      tricho  <-   st_read ( 'data/shapefiles/Ptri_shapefile/data/commondata/data0/poputric.shp' )    
  ## Reading layer `poputric' from data source 
##   `/home/alayna/Documents/research/projects/2023_populus_common_gardens/data/shapefiles/Ptri_shapefile/data/commondata/data0/poputric.shp' 
##   using driver `ESRI Shapefile'
## Simple feature collection with 450 features and 5 fields
## Geometry type: POLYGON
## Dimension:     XY
## Bounding box:  xmin: -17154180 ymin: 3609620 xmax: -11488690 ymax: 8896231
## Projected CRS: WGS 84 / Pseudo-Mercator  
       # convert CRS  
   balsam  <-   st_transform (balsam, crs.cna) 
   tricho  <-   st_transform (tricho, crs.cna) 
    
    # test plot  
    plot (hist) 
    
    plot (balsam $ geometry,  add =  T,  border =   'navy' ) 
    plot (tricho $ geometry,  add =  T,  border =   'grey50' ) 
    plot (borders $ geometry,  add =  T)    
   
 
 
  2.2  Extract future
climates for each genotype’s home site 
       # extract from raster and add to geno.info  
    
    # 8 GCM ensemble  
   tmp  <-   extract (fut8,  y =   st_as_sf (coords.prov))    
  ## Warning: [extract] transforming vector data to the CRS of the raster  
      geno.info $ future8_MCMT  <-  tmp[, 2 ] 
    
    # 13 GCM ensemble  
   tmp  <-   extract (fut13,  y =   st_as_sf (coords.prov))    
  ## Warning: [extract] transforming vector data to the CRS of the raster  
      geno.info $ future13_MCMT  <-  tmp[, 2 ] 
    
    # predictions from the two ensembles are very similar  
    plot (geno.info $ future8_MCMT, geno.info $ future13_MCMT) 
    abline ( 0 ,  1 ,  col =   'blue' )    
   
 
 
 
  3  Predict height at
future climates 
 
  3.1  Predict
functions 
       # originally from transfer function script  
    
    # predict each genotype's response to a vector of climate (MCMT) values  
    # garden_clims is a vector  
    
   predict_genotype  <-   function (model,  
                                 type =   'response' ,  # can also be conditional or zprob  
                                effects, 
                                home_clim, 
                                garden_clims){ 
      
      # vector to save predictions  
     pred_height  <-   vector () 
      
      # loop through garden climates  
      for (g  in   1  :  length (garden_clims)){ 
        
       home  <-  home_clim 
       garden  <-  garden_clims[g] 
        
        # make newdata to give to predict()  
       new_data  <-   as.data.frame ( cbind (home, garden, home ^  2 , garden ^  2 )) 
        colnames (new_data)  <-   c ( 'home_clim' ,  'garden_clim' ,  'home_clim_2' ,  'garden_clim_2' ) 
        # add other random or fixed effects  
          for (n  in   1  :  length (effects)){ 
           new_data[ names (effects[n])]  <-  effects[n] 
         } 
        
        # first have to scale new_data to match the scaled data used in model  
        for (v  in   1  :  length (new_data)){ 
         var  <-   names (new_data)[v] 
          
          # only scale if var exists in scaling_factor - random effects like genotype are not in scaling factor  
          # IF VARIABLES ARE NAMED DIFFERENTLY HERE IN effects AND scaling_factor IT WILL SKIP THEM  
          if (var  %in%   names (scaling_factor)){ 
           new_data[var]  <-  new_data[var] * scaling_factor[[var]] 
         }  else  { 
            warning (var,  ' does not have a scaling factor, skipping' ) 
         } 
          
          
       } 
        
       pred  <-   predict (mod,  type =  type,  newdata =  new_data,  re.form =   NA ,  allow.new.levels=  TRUE ) 
        # back transform from log if we're predicting height  
        # zprob is probability of a zero, so don't transform  
        if (type  %in%   c ( 'response' ,  'conditional' )){ 
         pred  <-   exp (pred) -  1  
       } 
       pred_height[g]  <-  pred 
        
     } 
      
      # save  
     pred_height_df  <-   data.frame ( garden_clim =  garden_clims,  predicted_height =  pred_height)  
      
    return (pred_height_df) 
   }    
 
 
  3.2  Predict response to
historic and future climate for each genotype 
       # model predictions for historic data  
    # we already have historic provenance data for the model, so don't need to extract it from the raster  
    
    # what climate variable are we using?  
   clim_var  <-   'MCMT'  
   prov_clim_colname  <-   paste ( 'provenance' , clim_var,  sep =   '_' ) 
   gard_clim_colname  <-   paste ( 'garden' , clim_var,  '1961_1990' ,  sep =   '_' ) 
   fut8_colname  <-   paste ( 'future8' , clim_var,  sep =   '_' ) 
   fut13_colname  <-   paste ( 'future13' , clim_var,  sep =   '_' ) 
    
    
    # add predictions to geno.info dataframe  
    # historic  
   geno.info $ hist_response  <-   NA  
   geno.info $ hist_height  <-   NA  
   geno.info $ hist_mortality  <-   NA  
    
    # future 8  
   geno.info $ fut8_response  <-   NA  
   geno.info $ fut8_height  <-   NA  
   geno.info $ fut8_mortality  <-   NA  
    
    # future 13  
   geno.info $ fut13_response  <-   NA  
   geno.info $ fut13_height  <-   NA  
   geno.info $ fut13_mortality  <-   NA  
    
    
    for (n  in   1  :  nrow (geno.info)){ 
      
     geno  <-   rownames (geno.info)[n] 
      
      # setup effects  
     g.pc1  <-  geno.info $ genetic_PC1[n] 
     g.pc2  <-  geno.info $ genetic_PC2[n] 
     g.pc3  <-  geno.info $ genetic_PC3[n] 
     g.home_clim  <-  geno.info[,prov_clim_colname][n] 
      
      # garden clims are the historic and future climates  
      # in order: home climate (historic), future climate 8 GCMs, future climate 13 GCMs  
     g.clims  <-   c (g.home_clim, geno.info[,fut8_colname][n], geno.info[,fut13_colname][n]) 
      
      # predict overall response  
     resp  <-   predict_genotype (mod, 
                       type =   'response' , 
                       effects =   list ( pc1 =  g.pc1,  
                                      pc2 =  g.pc2, 
                                      pc3 =  g.pc3, 
                                      genotype =  geno),  
                       home_clim =  g.home_clim,  
                       garden_clims =  g.clims) 
      
      # save to df  
     geno.info $ hist_response[n]  <-  resp $ predicted_height[ 1 ] 
     geno.info $ fut8_response[n]  <-  resp $ predicted_height[ 2 ] 
     geno.info $ fut13_response[n]  <-  resp $ predicted_height[ 3 ] 
    
      
      
      # predict height (conditional model)  
     cond  <-   predict_genotype (mod, 
                               type =   'conditional' , 
                               effects =   list ( pc1 =  g.pc1,  
                                              pc2 =  g.pc2, 
                                              pc3 =  g.pc3, 
                                              genotype =  geno),  
                               home_clim =  g.home_clim,  
                               garden_clims =  g.clims) 
      
      # save to df  
     geno.info $ hist_height[n]  <-  cond $ predicted_height[ 1 ] 
     geno.info $ fut8_height[n]  <-  cond $ predicted_height[ 2 ] 
     geno.info $ fut13_height[n]  <-  cond $ predicted_height[ 3 ] 
    
      # predict probability of mortality (zero-inflated model)  
     zi  <-   predict_genotype (mod, 
                             type =   'zprob' , 
                             effects =   list ( pc1 =  g.pc1,  
                                            pc2 =  g.pc2, 
                                            pc3 =  g.pc3, 
                                            genotype =  geno),  
                             home_clim =  g.home_clim,  
                             garden_clims =  g.clims) 
      # save to df  
     geno.info $ hist_mortality[n]  <-  zi $ predicted_height[ 1 ] 
     geno.info $ fut8_mortality[n]  <-  zi $ predicted_height[ 2 ] 
     geno.info $ fut13_mortality[n]  <-  zi $ predicted_height[ 3 ] 
    
      
     #cat(paste('done with', n, '\n'))  
      
   } 
    
    # predict_genotype() gives warning:  
    # '## Warning in predict_genotype(mod, type = "response", effects = list(pc1 = g.pc1,  
    ## : genotype does not have a scaling factor, skipping'  
    
    # this is fine, genotype is a string not numeric and shouldn't be scaled     
 
 
  3.3  Calculate change in
fitness under future climates 
       # calculate changes in fitness under future climates  
    
    ##########  
    # future (8 GCMs)  
    
    # net change in response  
   geno.info $ fut8_response_change  <-  geno.info $ fut8_response  -  geno.info $ hist_response 
    # percent change  
   geno.info $ fut8_response_change_percent  <-  (geno.info $ fut8_response  -  geno.info $ hist_response) / geno.info $ hist_response 
    
    # net change in height  
   geno.info $ fut8_height_change  <-  geno.info $ fut8_height  -  geno.info $ hist_height 
    # percent change  
   geno.info $ fut8_height_change_percent  <-  (geno.info $ fut8_height  -  geno.info $ hist_height) / geno.info $ hist_height 
    
    # net change in mortality probability  
   geno.info $ fut8_mortality_change  <-  geno.info $ fut8_mortality  -  geno.info $ hist_mortality 
    # percent change  
   geno.info $ fut8_mortality_change_percent  <-  (geno.info $ fut8_mortality  -  geno.info $ hist_mortality) / geno.info $ hist_mortality 
    
    ##########  
    # future (13 GCMs)  
    
    # net change in response  
   geno.info $ fut13_response_change  <-  geno.info $ fut13_response  -  geno.info $ hist_response 
    # percent change  
   geno.info $ fut13_response_change_percent  <-  (geno.info $ fut13_response  -  geno.info $ hist_response) / geno.info $ hist_response 
    
    # net change in height  
   geno.info $ fut13_height_change  <-  geno.info $ fut13_height  -  geno.info $ hist_height 
    # percent change  
   geno.info $ fut13_height_change_percent  <-  (geno.info $ fut13_height  -  geno.info $ hist_height) / geno.info $ hist_height 
    
    # net change in mortality probability  
   geno.info $ fut13_mortality_change  <-  geno.info $ fut13_mortality  -  geno.info $ hist_mortality 
    # percent change  
   geno.info $ fut13_mortality_change_percent  <-  (geno.info $ fut13_mortality  -  geno.info $ hist_mortality) / geno.info $ hist_mortality 
    
    # preliminary plots  
    par ( mfrow =   c ( 2 , 3 )) 
    hist (geno.info $ fut8_response_change_percent) 
    hist (geno.info $ fut8_height_change_percent) 
    hist (geno.info $ fut8_mortality_change_percent) 
    
    hist (geno.info $ fut13_response_change_percent) 
    hist (geno.info $ fut13_height_change_percent) 
    hist (geno.info $ fut13_mortality_change_percent)    
   
 
 
 
  4  Map predictions for
each collected genotype/site 
       # baseline plot of climate difference  
    
   diff13  <-  fut13  -  hist 
   diff8  <-  fut8  -  hist 
    
    par ( mfrow =   c ( 1 , 2 )) 
    plot (diff13) 
    plot (diff8)    
   
       # color palette for MCMT base layer  
    
   colf  <-   colorRampPalette ( brewer.pal ( 9 ,  'YlOrRd' )) 
   cols  <-   colf ( 100 ) 
   cols2  <-   adjustcolor (cols,  alpha =   0.5 ) 
    
    # function to map the response   
    
    # future = the raster object for change in climate to use as background map  
    # response = the column name from geno.info of the response to future climate to plot  
    # main = plot title  
    # rev = reverse response color palette? Can use for mortality so a negative/positive response has the same color  
   map_change  <-   function (future, response,  main =   '' ,  rev =   FALSE ,  legend =   FALSE ,  raster =   FALSE , ...){ 
      
      # which model response are we plotting?  
     resp_name  <-  response 
      
     colf  <-   colorRampPalette ( brewer.pal ( 9 ,  'YlOrRd' ),  alpha =   0.5 ) 
     cols  <-   colf ( 100 ) 
     cols  <-   adjustcolor (cols,  alpha =   0.6 ) 
      
      #par(mfrow = c(1,1))  
      plot (future,  
           col =   ifelse (raster  ==   TRUE , cols,  'white' ), 
           main =  main, 
           xaxt =   'n' ,  yaxt =   'n' ,  
           pax =   list ( side=  NA ), 
           xlim =   c ( -  3000000 , -  500000 ),  ylim =   c ( 400000 , 3200000 ),  
           legend =  legend, 
           plg =   list ( title =   'Future -  \n  Historic  \n   MCMT (°C)' ), 
          ...) 
      
      # add borders and species ranges  
      plot (borders $ geometry,  add =  T,  lwd =   0.5 ) 
      plot (balsam $ geometry,  add =  T,  border =   'navy' ) 
      plot (tricho $ geometry,  add =  T,  border =   'darkgreen' ) 
      
    
      # color palette for change in response  
     min_change  <-   min (geno.info[,resp_name]) 
     max_change  <-   max (geno.info[,resp_name]) 
      
     ceil2  <-   max ( abs (min_change),  abs (max_change)) 
      
      if (rev  ==   FALSE ){ 
       colf  <-   colorRamp2 (  breaks =   c ( - ceil2,  0 , ceil2),  colors =   c ( 'darkorchid4' ,  'white' ,  'palegreen4' )) 
     }  else   if (rev  ==   TRUE ){ 
       colf  <-   colorRamp2 (  breaks =   c ( - ceil2,  0 , ceil2),  colors =   c ( 'palegreen4' ,  'white' ,  'darkorchid4' )) 
     } 
      
      
     cols2  <-   colf (geno.info[,resp_name]) 
      
      # plot points for each genotype  
      points (coords.prov.cna,  bg =  cols2,   pch =   21 ,  cex =   2 ,  lwd =   1.5 ) 
      
      # add colors of upper and lower bounds to legend  
      if (rev  ==   FALSE ){ 
        pt.cols  <-   c ( 'darkorchid4' ,  'white' ,  'palegreen4' ) 
     }  else   if (rev  ==   TRUE ) { 
       pt.cols  =   c ( 'palegreen4' ,  'white' ,  'darkorchid4' ) 
     } 
     
      
      # legend(-2.8e6,1.5e6, title = '% Change',  
      #        legend = c(round(-ceil2,2), 0, round(ceil2,2)),  
      #        pch = 21,  
      #        pt.bg = pt.cols,  
      #        pt.cex = 2)  
      
     vals  <-   round ( c (min_change,  0 , max_change), 2 ) 
      # if range doesn't overlap 0, remove it  
      if (min_change  *  max_change  >   0 ){ 
       vals  <-  vals[vals !=  0 ] 
     } 
      
      legend ( -  2.8e6 , 1.5e6 ,  title =   '% Change' , 
             legend =  vals, 
             pch =   21 , 
             pt.bg =   colf (vals), 
             pt.cex =   2 ) 
      
      
   } 
    
    
    par ( mfrow =   c ( 2 , 3 )) 
    map_change (diff13,  'fut13_response_change_percent' ) 
    map_change (diff13,  'fut13_height_change_percent' ) 
    map_change (diff13,  'fut13_mortality_change_percent' ,  rev =  T) 
    
    map_change (diff8,  'fut8_response_change_percent' ) 
    map_change (diff8,  'fut8_height_change_percent' ) 
    map_change (diff8,  'fut8_mortality_change_percent' ,  rev =  T)    
   
       # raw values (cm or % mortality)  
    # legend title not correct here  
    par ( mfrow =   c ( 2 , 3 )) 
    map_change (diff13,  'fut13_response_change' ) 
    map_change (diff13,  'fut13_height_change' ) 
    map_change (diff13,  'fut13_mortality_change' ,  rev =  T) 
    
    map_change (diff8,  'fut8_response_change' ) 
    map_change (diff8,  'fut8_height_change' ) 
    map_change (diff8,  'fut8_mortality_change' ,  rev =  T)    
   
       #png(file = paste('results/model_prediction/fitness_changes_map_2yearModel', clim_var, '_13GCMs.png', sep = ''), height = 8, width = 8, res = 300, units = 'in')  
    
    
    # lighter colors for temperature raster  
   colf  <-   colorRampPalette ( brewer.pal ( 9 ,  'YlOrRd' )) 
   cols  <-   colf ( 100 ) 
   cols2  <-   adjustcolor (cols,  alpha =   0.5 ) 
    
    # plot genotypes and the 13-gcm ensemble  
    par ( mfrow =   c ( 2 , 2 )) 
    # set margins  
   marg  <-   c ( 1 , 1 , 4 , 3 ) 
    
    plot (diff13,  col =  cols2,  
         main =   paste (clim_var,  ' 2041-2070, 13 GCMs' ,  sep =   '' ), 
         xaxt =   'n' ,  yaxt =   'n' ,  
         pax =   list ( side=  NA ), 
         mar =  marg, 
         xlim =   c ( -  3000000 , -  500000 ),  ylim =   c ( 400000 , 3200000 ),  
         plg =   list ( title =   'Future -  \n  Historic  \n   MCMT (°C)' )) 
    # add borders and species ranges  
    plot (borders $ geometry,  add =  T,  lwd =   0.5 ) 
    plot (balsam $ geometry,  add =  T,  border =   'navy' ) 
    plot (tricho $ geometry,  add =  T,  border =   'darkgreen' ) 
    
    points (coords.prov.cna,  bg =  geno.info $ color_Pt,  pch =   21 ,  cex =   2 ) 
    
    map_change (diff13,  'fut13_response_change_percent' ,  main =   'Overall response' ,  mar =  marg) 
    map_change (diff13,  'fut13_height_change_percent' ,  main =   'Growth' ,  mar =  marg) 
    map_change (diff13,  'fut13_mortality_change_percent' ,  rev =  T,  main =   'Probability of Mortality' ,  mar =  marg)    
   
       #dev.off()     
 
 
  5  Map rangewide
predictions for each genotype 
 
  5.1  Map past and future
fitness predictions and change for each genotype 
       # custom predict function - predict height from discrete set of climate values that have already been calculated  
    
   predict_disc  <-   function (value, pred_clim, pred_value){ 
      if ( !   is.na (value)){ 
        # which value in pred_clim (already predicted climates) is closest to the value we want to predict?  
        # returns the index  
       ind  <-   which.min ( abs (pred_clim  -  value)) 
        
        # return the predicted height for that index  
        return (pred_value[ind]) 
        
     }  else   if ( is.na (value)){ 
        return ( NA ) 
     } 
   } 
    
    # which climate variable to use?  
   clim_var  <-   'MCMT'  
   hist_rast  <-  hist 
   fut_rast  <-  fut13 
    
    # set up input raster - historic climate  
   hist.crop  <-   crop (hist_rast,  ext ( -  4e6 ,  0 ,  0 ,  3e6 )) 
    plot (hist.crop)    
   
       # make values outside predict range NA  
    # need pred_clim - this is the same for all genotypes, so can just use first one  
    #pred_clim <- preds$overall$genotype_210$garden_clim  
    #pred_clim <- dat[,gard_clim_colname]  
   pred_clim  <-   c (dat $ garden_MCMT_2021, dat $ garden_MCMT_2022) 
    
   msk  <-   ifel (hist.crop  >   max (pred_clim)  |  hist.crop  <   min (pred_clim),  NA ,  1 ) 
   hist.crop  <-   mask (hist.crop, msk) 
    plot (hist.crop,  colNA =   'grey' )    
   
       # future climate  
   fut13.crop  <-   crop (fut_rast,  ext ( -  4e6 ,  0 ,  0 ,  3e6 )) 
    plot (fut13.crop)    
   
       # make values outside predict range NA  
    # need pred_clim - this is the same for all genotypes, so can just use first one  
    # pred_clim <- preds$overall$genotype_210$garden_clim  
   msk  <-   ifel (fut13.crop  >   max (pred_clim)  |  fut13.crop  <   min (pred_clim),  NA ,  1 ) 
   fut13.crop  <-   mask (fut13.crop, msk) 
    plot (fut13.crop,  colNA =   'grey' )    
   
       # loop through genotypes, predict height across range  
    
    par ( mfrow =   c ( 1 , 3 ),  mar =   c ( 5 , 4 , 4 , 5 )) 
    for (n  in   1  :  length (preds $ overall)){ 
      
      # predicted climate and predicted height for this genotype  
     pred_clim  <-  preds $ overall[[n]] $ garden_clim 
     pred_value  <-  preds $ overall[[n]] $ predicted_height 
      
      #### historic  
      # set heights for each raster cell in historic climate (returns a list)  
     heights_cells_hist  <-   lapply ( values (hist.crop),  FUN =   function (x)  predict_disc (x, pred_clim, pred_value)) 
      # assign heights to raster  
     height_hist  <-   setValues (hist.crop,  values =   unlist (heights_cells_hist)) 
      
      # plot  
      colf  <-   colorRampPalette ( rev ( brewer.pal ( 9 ,  'YlGn' ))) 
      cols  <-   rev ( colf ( 100 )) 
      
      plot (height_hist,  col =  cols,  main =   paste ( names (preds $ overall)[n],  '  \n  Fitness in Historic Climate' ,  sep =   '' ),  colNA =   'grey90' ) 
      
      plot (borders $ geometry,  add =  T) 
      plot (balsam $ geometry,  add =  T,  border =   'navy' ) 
      plot (tricho $ geometry,  add =  T,  border =   'grey50' ) 
      
      points (coords.prov.cna[n],  col =   'mediumpurple' ,  lwd =   2 ,  cex =   1.5 ) 
      #points(coords.prov.cna)  
      
        #### future  
      # set heights for each raster cell in historic climate (returns a list)  
     heights_cells_fut  <-   lapply ( values (fut13.crop),  FUN =   function (x)  predict_disc (x, pred_clim, pred_value)) 
      # assign heights to raster  
     height_fut  <-   setValues (fut13.crop,  values =   unlist (heights_cells_fut)) 
      
      # plot  
    
      plot (height_fut,  col =  cols,  main =   'Fitness in Future Climate' ,  colNA =   'grey90' ) 
      
      plot (borders $ geometry,  add =  T) 
      plot (balsam $ geometry,  add =  T,  border =   'navy' ) 
      plot (tricho $ geometry,  add =  T,  border =   'grey50' ) 
      
      points (coords.prov.cna[n],  col =   'mediumpurple' ,  lwd =   2 ,  cex =   1.5 ) 
      #points(coords.prov.cna)  
      
      ### plot difference  
     height_diff  <-  height_fut  -  height_hist 
      
      # min and max values for diverging color palette  
     min_var  <-   minmax (height_diff)[ 1 ,] 
     max_var  <-   minmax (height_diff)[ 2 ,] 
     ceil  <-   max ( abs (min_var),  abs (max_var)) 
      
     colf_diff  <-   colorRampPalette ( brewer.pal ( 11 ,  'RdBu' )) 
     cols_diff  <-   colf_diff ( 100 ) 
      
      plot (height_diff,  col =  cols_diff,  range =   c ( - ceil, ceil),  main =   'Fitness change' ,  colNA =   'grey90' ) 
      
      plot (borders $ geometry,  add =  T) 
      plot (balsam $ geometry,  add =  T,  border =   'navy' ) 
      plot (tricho $ geometry,  add =  T,  border =   'grey50' ) 
      
      points (coords.prov.cna[n],  col =   'black' ,  lwd =   2 ,  cex =   1.5 ) 
      
      print ( paste ( 'done with' , n)) 
      
   }    
   
  ## [1] "done with 1"  
   
  ## [1] "done with 2"  
   
  ## [1] "done with 3"  
   
  ## [1] "done with 4"  
   
  ## [1] "done with 5"  
   
  ## [1] "done with 6"  
   
  ## [1] "done with 7"  
   
  ## [1] "done with 8"  
   
  ## [1] "done with 9"  
   
  ## [1] "done with 10"  
   
  ## [1] "done with 11"  
   
  ## [1] "done with 12"  
   
  ## [1] "done with 13"  
   
  ## [1] "done with 14"  
   
  ## [1] "done with 15"  
   
  ## [1] "done with 16"  
   
  ## [1] "done with 17"  
   
  ## [1] "done with 18"  
   
  ## [1] "done with 19"  
   
  ## [1] "done with 20"  
   
  ## [1] "done with 21"  
   
  ## [1] "done with 22"  
   
  ## [1] "done with 23"  
   
  ## [1] "done with 24"  
   
  ## [1] "done with 25"  
   
  ## [1] "done with 26"  
   
  ## [1] "done with 27"  
   
  ## [1] "done with 28"  
   
  ## [1] "done with 29"  
   
  ## [1] "done with 30"  
   
  ## [1] "done with 31"  
   
  ## [1] "done with 32"  
   
  ## [1] "done with 33"  
   
  ## [1] "done with 34"  
   
  ## [1] "done with 35"  
   
  ## [1] "done with 36"  
   
  ## [1] "done with 37"  
   
  ## [1] "done with 38"  
   
  ## [1] "done with 39"  
   
  ## [1] "done with 40"  
   
  ## [1] "done with 41"  
   
  ## [1] "done with 42"  
   
  ## [1] "done with 43"  
   
  ## [1] "done with 44"  
 
 


 

 

 

 

 


 
 

 
 
