# Supplementary material for: Variation in responses to temperature across admixed genotypes of Populus trichocarpa × P. balsamifera predict geographic shifts in regions where hybrids are favored
Source: bioRxiv. 2025 May 22:2025.05.16.654548. Preprint. [Version 1] doi: 10.1101/2025.05.16.654548 (PMC12139819; doi:10.1101/2025.05.16.654548)
Supplement: Supplement 3 [file media-3.gz › transfer_function_multiyear_linear_mixed_effects_model.html]

 

 

 

 
 
 


 

 

 Predict genotype-specific reaction norms 

 
 
 
 
 
 
 
 
 

 


 
 
 


 


 

 

 


 

 


 


 


 Predict genotype-specific reaction
norms 
 Alayna Mead 
 2025-05-12 

 

 
 
   1  Setup 
 
   1.1  data and packages  
   1.2  Clean up input data  
   1.3  Save input data  
  
   2  Functions  
   3  Multi-year Model Setup 
 
   3.1  Plot MCMT over three
years  
   3.2  Select variables and merge years
into one dataframe  
   3.3  Scale variables  
  
   4  Run multiyear models 
 
   4.1  Compare setting year as a fixed vs
random effect  
  
   5  Plots for selected model  
   6  Model validation using subsets 
 
   6.1  By garden  
   6.2  By garden + year  
  
   7  Compare model predictive ability
when genetic and home climate information is removed  
   8  Predicting phenotypes based on
model 
 
   8.1  Function for predicting the
response to climate  
   8.2  Setup  
   8.3  Predict response to climate by
genotype  
  
 
 

 Test the effects of home climate, garden climate, and genetics on the
growth and mortality responses of hybrid poplars across different common
gardens, then predict genotype-specific reaction norms to MCMT. Uses
glmmTMB to fit a zero-inflated mixed-effects model. 
 
  1  Setup 
 
  1.1  data and
packages 
       library (ggplot2) 
    library (colorRamp2) 
    library (RColorBrewer) 
    library (lme4)    
  ## Loading required package: Matrix  
       library (sjPlot)  # nice plots of models  
    library (glmmTMB)  #zero-inflated  model and also used with sjPlot  
    library (bbmle)  # for AICtab     
  ## Loading required package: stats4  
       library (patchwork)  # plot ggplots together  
    library (ggpubr)  # easily add pvals to ggplots  
    library (performance)  # for rmse (root mean squared error function)  
    library (psych)  # for pairs.panels()     
  ## 
## Attaching package: 'psych'  
  ## The following objects are masked from 'package:ggplot2':
## 
##     %+%, alpha  
       # using the merged 2024 dataset because Idaho wasn't included in the old 2023 merged file  
    # remove the 2024 data  
    load ( 'data/clean/mini_garden_phenotypic_and_climate_data_2021-2024.Rdata' ) 
   dat  <-  dat[,  !  endsWith ( colnames (dat),  '2024' )] 
    
    str (dat)    
  ## 'data.frame':    1656 obs. of  388 variables:
##  $ Unique_ID                          : chr  "EVERGREEN.206.1.7.4" "EVERGREEN.206.2.4.1" "EVERGREEN.210.1.7.3" "EVERGREEN.210.2.4.2" ...
##  $ MiniCG_Site                        : Factor w/ 17 levels "OLLU","LOCK",..: 16 16 16 16 16 16 16 16 16 16 ...
##  $ Genotype                           : Factor w/ 47 levels "206","210","218",..: 1 1 2 2 3 3 4 4 5 5 ...
##  $ Plant_ID                           : chr  "206" "206" "210" "210" ...
##  $ Pb                                 : num  0.4289 0.4289 0.0916 0.0916 0.1356 ...
##  $ Pt                                 : num  0.571 0.571 0.908 0.908 0.864 ...
##  $ transect                           : Factor w/ 5 levels "Alaska","Cassiar",..: 3 3 3 3 3 3 3 3 3 3 ...
##  $ provenance_latitude                : num  51.9 51.9 52.1 52.1 52 ...
##  $ provenance_longitude               : num  -124 -124 -123 -123 -122 ...
##  $ provenance_elevation_m             : num  1064 1064 744 744 381 ...
##  $ block                              : int  1 2 1 2 1 2 1 2 1 2 ...
##  $ row                                : int  7 4 7 4 8 2 2 5 3 3 ...
##  $ column                             : chr  "4" "1" "3" "2" ...
##  $ Survival_09_2021                   : int  1 1 1 1 1 1 1 1 1 1 ...
##  $ PreFlush_Height_cm_2021            : num  12.5 8 12 11 11 17 12.5 17 16.5 12 ...
##  $ PostSet_Height_cm_2021             : num  43.3 45.7 42.7 45.7 36.6 36.6 36.6 41.1 36.6 41.1 ...
##  $ GrowthIncrement_2021               : num  30.8 37.7 30.7 34.7 25.6 19.6 24.1 24.1 20.1 29.1 ...
##  $ PetioleColor_2021                  : chr  NA NA NA NA ...
##  $ RustDisease_2021                   : int  0 1 1 1 0 1 0 1 0 1 ...
##  $ DOY_Stage2_2021                    : num  92 88 88 88 85 85 95 92 88 88 ...
##  $ DOY_Stage3_2021                    : num  103 99 103 103 103 103 107 107 95 103 ...
##  $ DOY_Stage6_2021                    : num  200 264 200 264 283 266 190 283 190 200 ...
##  $ Stage2_cGDD_2021                   : num  488 462 462 462 441 ...
##  $ Stage3_cGDD_2021                   : num  554 534 554 554 554 ...
##  $ MAT_TransferDist                   : num  8.1 8.1 6.6 6.6 4.8 4.8 6.5 6.5 9.2 9.2 ...
##  $ MAP_TransferDist                   : int  357 357 461 461 462 462 176 176 -373 -373 ...
##  $ MiniCG_Site_2022                   : Factor w/ 15 levels "VA","UCM","SU",..: 14 14 14 14 14 14 14 14 14 14 ...
##  $ Unique_ID_2022                     : chr  "EVERGREEN.206.1.7.4" "EVERGREEN.206.2.4.1" "EVERGREEN.210.1.7.3" "EVERGREEN.210.2.4.2" ...
##  $ Genotype_2022                      : Factor w/ 47 levels "206","210","218",..: 1 1 2 2 3 3 4 4 5 5 ...
##  $ PLANT_ID_2022                      : chr  "206" "206" "210" "210" ...
##  $ Survival_09_2022                   : num  1 1 1 1 1 1 1 1 1 1 ...
##  $ PreFlush_Height_cm_2022            : num  44.2 47.2 43.6 45.7 35.1 ...
##  $ PostSet_Height_cm_2022             : num  39.6 57.9 51.8 85.3 112.8 ...
##  $ GrowthIncrement_2022               : num  NA 10.67 8.23 39.62 77.72 ...
##  $ RustDisease_2022                   : chr  "1" "1" "1" "1" ...
##  $ DOY_Stage2_2022                    : num  87 87 87 87 87 87 95 95 95 90 ...
##  $ DOY_Stage3_2022                    : num  95 95 95 98 95 95 98 98 98 98 ...
##  $ DOY_Stage6_2022                    : num  188 188 207 207 225 207 207 207 188 188 ...
##  $ Stage2_cGDD_2022                   : int  494 494 494 494 494 494 559 559 559 522 ...
##  $ Stage3_cGDD_2022                   : int  559 559 559 584 559 559 584 584 584 584 ...
##  $ Unique_ID_2023                     : chr  "EVERGREEN.206.1.7.4" "EVERGREEN.206.2.4.1" "EVERGREEN.210.1.7.3" "EVERGREEN.210.2.4.2" ...
##  $ MiniCG_Site_2023                   : Factor w/ 17 levels "OLLU","LOCK",..: 16 16 16 16 16 16 16 16 16 16 ...
##  $ Genotype_2023                      : chr  "206" "206" "210" "210" ...
##  $ block_2023                         : num  1 2 1 2 1 2 1 2 1 2 ...
##  $ row_2023                           : num  7 4 7 4 8 2 2 5 3 3 ...
##  $ column_2023                        : chr  "4" "1" "3" "2" ...
##  $ DOY_Stage2.DD.MM.YY_2023           : chr  "07.04.23" "11.04.23" "03.04.23" "11.04.23" ...
##  $ DOY_Stage3.DD.MM.YY_2023           : chr  "19.04.23" "21.04.23" "19.04.23" "19.04.23" ...
##  $ DOY_Stage6.DD.MM.YY_2023           : chr  "30.06.23" "12.06.23" "30.06.23" "12.06.23" ...
##  $ DOY_Stage7.DD.MM.YY_2023           : chr  "" "20.07.23" "06.07.23" "" ...
##  $ DOY_Stage8.DD.MM.YY_2023           : chr  "" "03.08.23" "20.07.23" "" ...
##  $ Survival_09_2023                   : num  1 1 1 1 1 1 1 1 1 1 ...
##  $ PreFlush_Height_cm_2023            : num  45.7 61 47.5 75 102.4 ...
##  $ PostSet_Height_cm_2023             : num  45.7 106.7 70.1 140.2 128 ...
##  $ GrowthIncrement_2023               : num  0 45.7 22.6 65.2 25.6 ...
##  $ RustDisease.1.or.0_2023            : chr  NA NA NA NA ...
##  $ DOY_Stage2_2023                    : num  97 101 93 101 93 93 101 101 93 97 ...
##  $ DOY_Stage3_2023                    : num  109 111 109 109 109 109 117 114 101 109 ...
##  $ DOY_Stage6_2023                    : num  181 163 181 163 162 NA 187 163 162 163 ...
##  $ DOY_Stage7_2023                    : num  NA 201 187 NA 194 194 NA NA NA NA ...
##  $ DOY_Stage8_2023                    : num  NA 215 201 NA 240 NA 225 NA NA NA ...
##  $ Notes_2023                         : chr  "" "" "" "" ...
##  $ DOY_leaf_measurements.DD.MM.YY_2023: chr  "11.06.23" "12.06.23" "11.06.23" "12.06.23" ...
##  $ DOY_leaf_measurements_2023         : num  162 163 162 163 162 163 162 163 162 163 ...
##  $ leaf_measurement_growth_stage_2023 : chr  "4" "6" "4" "6" ...
##  $ leaf_thickness_1_mm_2023           : num  0.21 0.29 0.2 0.28 0.22 0.32 0.39 0.27 0.22 0.23 ...
##  $ leaf_thickness_2_mm_2023           : num  0.19 0.18 0.26 0.26 0.26 0.3 0.24 0.26 0.21 0.23 ...
##  $ leaf_thickness_3_mm_2023           : num  0.23 0.21 0.2 0.25 0.22 0.28 0.32 0.17 0.17 0.19 ...
##  $ leaf_thickness_4_mm_2023           : num  0.23 0.35 0.19 0.19 0.22 0.38 0.33 0.23 0.21 0.18 ...
##  $ leaf_thickness_5_mm_2023           : num  0.2 0.22 0.27 0.19 0.24 0.29 0.23 0.2 0.18 0.23 ...
##  $ leaf_thickness_avg_mm_2023         : num  0.212 0.25 0.224 0.234 0.232 0.314 0.302 0.226 0.198 0.212 ...
##  $ leaf_thickness_sd_mm_2023          : num  0.0179 0.0689 0.0378 0.0416 0.0179 ...
##  $ DOY_LICOR_measurement.DD.MM.YY_2023: chr  "11.06.23" "12.06.23" "11.06.23" "12.06.23" ...
##  $ DOY_LICOR_measurement_2023         : num  162 163 162 163 162 163 162 163 162 163 ...
##  $ weather_LICOR_measurement_2023     : chr  "Partly Cloudy, 64°F" "Sunny, 65°F" "Partly Cloudy, 64°F" "Sunny, 65°F" ...
##  $ LICOR_codes_2023                   : chr  "117-118-119" "79-80-81" "114-115-116" "82-83-84" ...
##  $ LICOR_notes_2023                   : chr  NA NA NA NA ...
##  $ Survival_09_2021_2023              : num  NA NA NA NA NA NA NA NA NA NA ...
##  $ notes_2023                         : chr  NA NA NA NA ...
##  $ leaf_mass_g_2023                   : num  NA NA NA NA NA NA NA NA NA NA ...
##  $ leaf_area_cm2_2023                 : num  NA NA NA NA NA NA NA NA NA NA ...
##  $ LMA_g_m2_2023                      : num  NA NA NA NA NA NA NA NA NA NA ...
##  $ DOY_Stage2.DD.MM.YY_2021           : chr  "4/2/21" "3/29/21" "3/29/21" "3/29/21" ...
##  $ DOY_Stage3.DD.MM.YY_2021           : chr  "4/13/21" "4/9/21" "4/13/21" "4/13/21" ...
##  $ DOY_Stage6.DD.MM.YY_2021           : chr  "7/19/21" "9/21/21" "7/19/21" "9/21/21" ...
##  $ DOY_Stage2.DD.MM.YY_2022           : chr  "3/28/22" "3/28/22" "3/28/22" "3/28/22" ...
##  $ DOY_Stage3.DD.MM.YY_2022           : chr  "4/5/22" "4/5/22" "4/5/22" "4/8/22" ...
##  $ DOY_Stage6.DD.MM.YY_2022           : chr  "7/7/22" "7/7/22" "7/26/22" "7/26/22" ...
##  $ total_growth_increment_2021_2022   : num  NA 48.4 38.9 74.3 103.3 ...
##  $ total_growth_increment_2021_2023   : num  NA 94.1 61.5 139.6 128.9 ...
##  $ rgr_2021                           : num  1.24 1.74 1.27 1.42 1.2 ...
##  $ rgr_2022                           : num  -0.109 0.204 0.173 0.624 1.169 ...
##  $ rgr_2023                           : num  0 0.56 0.388 0.626 0.223 ...
##  $ DOY_last_budset_2023               : num  181 215 201 163 240 NA 225 163 162 163 ...
##  $ stage7_presence_2023               : num  0 1 1 0 1 1 0 0 0 0 ...
##  $ growing_season_days_2021           : num  108 176 112 176 198 181 95 191 102 112 ...
##  $ growing_season_days_2022           : num  101 101 120 120 138 120 112 112 93 98 ...
##  $ growing_season_days_2023           : num  84 114 108 62 147 NA 124 62 69 66 ...
##  $ garden_Arboreta.University.Partner : chr  "Evergreen State" "Evergreen State" "Evergreen State" "Evergreen State" ...
##   [list output truncated]  
       # print session info  
    sessionInfo ()    
  ## R version 4.5.0 (2025-04-11)
## Platform: x86_64-pc-linux-gnu
## Running under: Arch Linux
## 
## Matrix products: default
## BLAS:   /usr/lib/libblas.so.3.12.0 
## LAPACK: /usr/lib/liblapack.so.3.12.0  LAPACK version 3.12.0
## 
## locale:
##  [1] LC_CTYPE=en_US.UTF-8       LC_NUMERIC=C              
##  [3] LC_TIME=en_US.UTF-8        LC_COLLATE=en_US.UTF-8    
##  [5] LC_MONETARY=en_US.UTF-8    LC_MESSAGES=en_US.UTF-8   
##  [7] LC_PAPER=en_US.UTF-8       LC_NAME=C                 
##  [9] LC_ADDRESS=C               LC_TELEPHONE=C            
## [11] LC_MEASUREMENT=en_US.UTF-8 LC_IDENTIFICATION=C       
## 
## time zone: US/Eastern
## tzcode source: system (glibc)
## 
## attached base packages:
## [1] stats4    stats     graphics  grDevices datasets  utils     methods  
## [8] base     
## 
## other attached packages:
##  [1] psych_2.5.3        performance_0.13.0 ggpubr_0.6.0       patchwork_1.3.0   
##  [5] bbmle_1.0.25.1     glmmTMB_1.1.11     sjPlot_2.8.17      lme4_1.1-37       
##  [9] Matrix_1.7-3       RColorBrewer_1.1-3 colorRamp2_0.1.0   ggplot2_3.5.2     
## 
## loaded via a namespace (and not attached):
##  [1] gtable_0.3.6        TMB_1.9.17          xfun_0.52          
##  [4] bslib_0.9.0         rstatix_0.7.2       insight_1.2.0      
##  [7] lattice_0.22-6      numDeriv_2016.8-1.1 vctrs_0.6.5        
## [10] tools_4.5.0         sjstats_0.19.0      Rdpack_2.6.4       
## [13] generics_0.1.3      parallel_4.5.0      datawizard_1.0.2   
## [16] tibble_3.2.1        pkgconfig_2.0.3     ggeffects_2.2.1    
## [19] lifecycle_1.0.4     compiler_4.5.0      farver_2.1.2       
## [22] sjmisc_2.8.10       mnormt_2.1.1        munsell_0.5.1      
## [25] carData_3.0-5       htmltools_0.5.8.1   sass_0.4.10        
## [28] yaml_2.3.10         Formula_1.2-5       car_3.1-3          
## [31] pillar_1.10.2       nloptr_2.2.1        jquerylib_0.1.4    
## [34] tidyr_1.3.1         MASS_7.3-65         cachem_1.1.0       
## [37] reformulas_0.4.0    abind_1.4-8         boot_1.3-31        
## [40] nlme_3.1-168        tidyselect_1.2.1    sjlabelled_1.2.0   
## [43] bdsmatrix_1.3-7     digest_0.6.37       mvtnorm_1.3-3      
## [46] dplyr_1.1.4         purrr_1.0.4         splines_4.5.0      
## [49] fastmap_1.2.0       grid_4.5.0          colorspace_2.1-1   
## [52] cli_3.6.4           magrittr_2.0.3      broom_1.0.8        
## [55] withr_3.0.2         backports_1.5.0     scales_1.3.0       
## [58] rmarkdown_2.29      ggsignif_0.6.4      evaluate_1.0.3     
## [61] knitr_1.50          rbibutils_2.3       mgcv_1.9-1         
## [64] rlang_1.1.6         Rcpp_1.0.14         glue_1.8.0         
## [67] renv_0.17.3         minqa_1.2.8         jsonlite_2.0.0     
## [70] R6_2.6.1  
       # ggplot settings  
    theme_set ( theme_bw ()  +   theme ( text=  element_text ( size=  16 )))    
      knitr :: opts_chunk $  set ( fig.width =   12 ,  
                          fig.height =   10 )    
 
 
  1.2  Clean up input
data 
 Set the growth increment to 0 for all trees that died and didn’t have
a growth increment value. This is needed to include both growth and
mortality measures within the same model; it is zero-inflated to
evaluate the zeros arising from mortality separately. 
       # dead trees should have a growth of zero if they were dead the whole year  
    # some trees died during the season and their height was still recorded - keep these  values since it represents real growth and should be related to fitness  
    
    
    # how many of these trees are there?  
    length ( which (dat $ Survival_09_2021  ==   0   &   is.na (dat $ GrowthIncrement_2021)))  # 336     
  ## [1] 336  
       length ( which (dat $ Survival_09_2022  ==   0   &   is.na (dat $ GrowthIncrement_2022)))  # 324     
  ## [1] 324  
       length ( which (dat $ Survival_09_2023  ==   0   &   is.na (dat $ GrowthIncrement_2023)))  # 337     
  ## [1] 337  
       # change these growth values from NA to 0  
   dat[ which (dat $ Survival_09_2021  ==   0   &   is.na (dat $ GrowthIncrement_2021)),  'GrowthIncrement_2021' ]  <-   0  
   dat[ which (dat $ Survival_09_2022  ==   0   &   is.na (dat $ GrowthIncrement_2022)),  'GrowthIncrement_2022' ]  <-   0  
   dat[ which (dat $ Survival_09_2023  ==   0   &   is.na (dat $ GrowthIncrement_2023)),  'GrowthIncrement_2023' ]  <-   0  
    
    
    # save a list of which trees are alive at the end of each year  
    # used later to exclude dead trees when evaluating conditional model  
   alive21  <-   rownames (dat)[dat $ Survival_09_2021  ==   1   &   !   is.na (dat $ Survival_09_2021)] 
   alive22  <-   rownames (dat)[dat $ Survival_09_2022  ==   1   &   !   is.na (dat $ Survival_09_2022)] 
   alive23  <-   rownames (dat)[dat $ Survival_09_2023  ==   1   &   !   is.na (dat $ Survival_09_2023)] 
    
    nrow (dat)    
  ## [1] 1656  
       length (alive21)    
  ## [1] 1259  
       length (alive22)    
  ## [1] 1049  
       length (alive23)    
  ## [1] 908  
       # how many trees have values of zero growth?  
    sum (dat $ GrowthIncrement_2021  ==   0 ,  na.rm =  T)  # 381     
  ## [1] 381  
       sum (dat $ GrowthIncrement_2022  ==   0 ,  na.rm =  T)  # 343     
  ## [1] 343  
       sum (dat $ GrowthIncrement_2023  ==   0 ,  na.rm =  T)  # 370     
  ## [1] 370  
       # total number of trees that are not NA  
    sum ( !   is.na (dat $ GrowthIncrement_2021))  # 1476     
  ## [1] 1476  
       sum ( !   is.na (dat $ GrowthIncrement_2022))  # 1234     
  ## [1] 1234  
       sum ( !   is.na (dat $ GrowthIncrement_2023))  # 1133     
  ## [1] 1133  
       # note: 'total_growth_increment_2021_2022' has already been calculated in cleanup script. The NAs for dead trees mean that the total growth increment will also (correctly) be an NA if the tree died during one of the years. Don't recalculate total growth increment here - if growth for one year was a 0 because it was dead, multi-year growth would sum to an actual number and would not be left out of the analysis, as it is when the value is NA.  
    # total_growth_increment summed across years is no longer used in this script, but I'll leave this note here in case that changes     
       # remove NA genotypes  
    
   dat  <-  dat[ !   is.na (dat $ Genotype),] 
    
    table (dat $ Genotype)     
  ## 
## 206 210 218 233 255 258 307 311 317 333 334 342 353 364 374 380 381 393 405 411 
##  34  32  33  36  32  37  38  34  33  37  34  36  35  34  33  31  35  34  36  36 
## 416 419 423 427 432 437 443 453 463 469 522 533 543 545 564 567 572 588 590 601 
##  36  36  33  32  37  39  36  36  33  33  34  33  32  32  34  32  30  31  32  36 
## 808 821 827 865 947 972 973 
##  32  31  36  31  34  36  43  
       length ( table (dat $ Genotype))  # 47 genotypes     
  ## [1] 47  
 
 
  1.3  Save input data 
       # save(dat, file = 'data/clean/mini_garden_phenotypic_and_climate_data_2021-2023_deadHeight0_removeNegativeGrowth.Rdata')  
    # write.csv(dat, file = 'data/clean/mini_garden_phenotypic_and_climate_data_2021-2023_deadHeight0_removeNegativeGrowth.csv')     
 
 
 
  2  Functions 
       # function to calculate correlation between predicted and observed values for a model (essentially the R value)  
    
    # mod is the model  
    # it pulls the dataset from the model object, which allows us to remove rows with NAs for just the variables used in this model  
    # re.form argument goes to predict(); to include random effects set to NULL, to set random effects to zero set to NA  
    # 'alive' is the list of individual trees that are alive and needs to be defined in the environment - it's set up in 'setup_choose_model_variables' chunk  
    
   model_R  <-   function (mod,  type =   'response' ,  re.form =   NULL ,  se.fit =   FALSE ){ 
      
      # get dataframe with all variables used in model  
     df  <-  mod $ frame 
      
      # first column is phenotype  
      colnames (df)[ 1 ]  <-   'pheno'  
      
      # make new dataframe with no NA values  
      # not necessary when using dataframe from model output  
      #noNA <- df[complete.cases(df),]  
    
      
      # predict phenotype (generally height)  
     df $ pred  <-   predict (mod,  type =  type,  re.form =  re.form,  se.fit =  se.fit) 
      
      # un-log-transform height  
     df $ pred  <-   exp (df $ pred)  -   1  
     df $ pheno  <-   exp (df $ pheno)  -   1  
      
      # if type = conditional, remove dead trees (which are modeled separately in zero-inflated model)  
      if (type  ==   'conditional' ){ 
       df  <-  df[alive,] 
     } 
      
      # calculate correlation between actual and predicted values  
     cor  <-   cor.test (df $ pheno, df $ pred) 
      
      return ( list ( actual =  df $ pheno,  predicted =  df $ pred,  cor =  cor,  data_noNAs =  df)) 
      
   } 
    
    # plot actual vs predicted values and prints the correlation and p-value using the output of model_R as 'input' argument  
    
   plot_predicted_vs_actual  <-   function (input,  title =   NULL ,  col =   rgb ( 0 , 0 , 0 , 0.1 ),  col_1to1 =   'red' ,  col_fit =   'blue' ,  legend =   TRUE ){ 
      
     pval  <-   round (input $ cor $ p.value,  4 ) 
     cor_est  <-   as.numeric ( round (input $ cor $ estimate,  3 )) 
     main  <-   paste (title,  '  \n  ' ,  'R = ' , cor_est,  ' | p = ' , pval,  sep =   '' ) 
      
      plot (input $ actual, input $ predicted,  
           pch =   16 ,  
           col =  col,  
           xlab =   "Height (actual)" ,  
           ylab =   "Height (predicted)" , 
           title (main,  adj =   0 )) 
      abline ( 0 ,  1 ,  col =  col_1to1,  lty =   2 )  # 1to1 line  
      abline ( lm (input $ predicted  ~  input $ actual),  col =  col_fit)  # fit line  
      
      # add text  
      if (legend  ==   TRUE ){ 
        legend ( 'bottomright' ,  fill =   c (col_1to1, col_fit),  legend =   c ( '1:1 line' ,  'Best fit' )) 
     } 
   } 
    
    
    #######  
    # for plotting actual mortality vs mortality probability - need logistic model  
    # this reports the correct statistics but the plot function just uses a linear line - should be changed to a binomial model  
    # but this is okay for a quick look  
    
    
   model_R_mortality  <-   function (mod,  re.form =   NULL ,  se.fit =   TRUE ){ 
      
      # setup  
      # include random effects in predictions or set random effects to zero?  
      # this is the re.form argument in predict()  
      #re.form <- ifelse(include_REs == TRUE, eval(NULL), NA)  
      
     df  <-  mod $ frame 
      
      # first column is phenotype  
      colnames (df)[ 1 ]  <-   'pheno'  
      
      # make new dataframe with no NA values  
      # not necessary when using dataframe from model output  
     noNA  <-  df[ complete.cases (df),] 
    
      
      # predict phenotype - here, probability of mortality  
     df $ pred  <-   predict (mod,  type =   'zprob' ,  re.form =  re.form,  se.fit =  se.fit) 
      
      # un-log-transform actual height  
     df $ pheno  <-   exp (df $ pheno)  -   1  
      
      # convert growth increment to binary 0/1  
      # if value is not 0, convert to 1  
     df $ alive  <-   ifelse (df $ pheno  ==   0 ,  0 ,  1 ) 
      
      # logistic model  
     mort  <-   glm (alive  ~  pred,  family =   'binomial' ,  data =  df) 
      
      # this really should be a logistic fit not linear, but using this for now  
     cor  <-   cor.test (df $ alive, df $ pred) 
      
      return ( list ( actual =  df $ alive,  predicted =  df $ pred,  cor =  cor,  data_noNAs =  noNA)) 
      
   } 
    
   plot_predicted_vs_actual_mortality  <-   function (input,  title =   NULL ,  col =   rgb ( 0 , 0 , 0 , 0.1 ),  col_1to1 =   'red' ,  col_fit =   'blue' ){ 
      
     pval  <-   round (input $ cor $ p.value,  4 ) 
     cor_est  <-   as.numeric ( round (input $ cor $ estimate,  3 )) 
     main  <-   paste (title,  '  \n  ' ,  'R = ' , cor_est,  ' | p = ' , pval,  sep =   '' ) 
      
      plot (input $ actual, input $ predicted,  
           pch =   16 ,  
           col =  col,  
           xlab =   "Mortality (actual)" ,  
           ylab =   "Probability of mortality" , 
           title (main,  adj =   0 )) 
      #abline(0, 1, col = col_1to1, lty = 2) # 1to1 line  
      abline ( lm (input $ predicted  ~  input $ actual),  col =  col_fit)  # fit line  
      
      # add text  
      
     # legend('bottomright', fill = c(col_1to1, col_fit), legend = c('1:1 line', 'Best fit'))  
      
   } 
    
    # compare the distribution of residuals among multiple models  
   plot_resid  <-   function (mods, ...){ 
      
      
      # mods should be a named list of model outputs  
      
      # set colors - cols25() with some similar colors removed  
     cols  <-   c ( "#1F78C8" ,  "#ff0000" ,  "#33a02c" ,  "#6A33C2" ,  "#ff7f00" ,  "#FFD700" ,  "#a6cee3" ,  "#FB6496" ,  "#b2df8a" ,  "#CAB2D6" ,  "#FDBF6F" ,  "#999999" ,  "#EEE685" ,  "#C8308C" ,  "#FF83FA" ,  "#C814FA" ,  "#0000FF" ,  "#36648B" ,  "#00E2E5" ,  "#00FF00" ,  "#778B00" ,  "#BEBE00" ,  "#8B3B00" ,  "#A52A3C" ) 
      
      
      par ( mfrow =   c ( 1 , 3 )) 
      
      # calculate residuals for all models  
     res  <-   list () 
      for (n  in   1  :  length (mods)){ 
       res[[n]]  <-   residuals (mods[[n]]) 
     } 
      
      ##############################  
      # version with transformed residuals (from log scale to cm)  
     res.cm  <-   list () 
      for (n  in   1  :  length (res)){ 
       res.cm[[n]]  <-   exp (res[[n]]) -  1  
     } 
      # plot first one  
      # ugly code to get the max value for y axis  
     ymax  <-   max ( sapply ( 1  :  length (res.cm),  FUN =    function (x)  max ( density (res.cm[[x]]) $ y))) 
      plot ( density (res.cm[[ 1 ]]),  col =  cols[ 1 ],  ylim =   c ( 0 , ymax),  main =   'residuals in cm' ) 
      abline ( v =   mean (res.cm[[ 1 ]]),  col =  cols[ 1 ]) 
      # add the rest  
      for (n  in   2  :  length (mods)){ 
        # get residuals and un-log-transfrom  
        lines ( density (res.cm[[n]]),  col =  cols[n]) 
        abline ( v =   mean (res.cm[[n]]),  col =  cols[n]) 
     } 
      
      abline ( v =   0 ,  lty =   2 ) 
      legend ( 'topright' ,  
             legend =   names (mods), 
             fill =  cols[ 1  :  length (mods)]) 
      
      #####################################  
      # untransformed (log scale) version  
      
      # ugly code to get the max value for y axis  
     ymax  <-   max ( sapply ( 1  :  length (res),  FUN =    function (x)  max ( density (res[[x]]) $ y))) 
      
      # plot first one  
    
      plot ( density (res[[ 1 ]]),  col =  cols[ 1 ],  main =   'log-transformed residuals' ,  ylim =   c ( 0 ,ymax)) 
      abline ( v =   mean (res.cm[[ 1 ]]),  col =  cols[ 1 ]) 
      # add the rest  
      for (n  in   2  :  length (mods)){ 
        lines ( density (res[[n]]),  col =  cols[n]) 
        abline ( v =   mean (res.cm[[n]]),  col =  cols[n]) 
     } 
      
      abline ( v =   0 ,  lty =   2 ) 
      legend ( 'topright' ,  
             legend =   names (mods), 
             fill =  cols[ 1  :  length (mods)]) 
      
      ########################################  
      # use absolute value to compare how "wrong" each model is without regard to over/underestimation  
      
      # calc absolute values  
     res.abs  <-   list () 
      for (n  in   1  :  length (res)){ 
       res.abs[[n]] <-   abs (res[[n]]) 
     } 
      
      # ugly code to get the max value for y axis  
     ymax  <-   max ( sapply ( 1  :  length (res.abs),  FUN =    function (x)  max ( density (res.abs[[x]]) $ y))) 
      
      # plot first one  
      plot ( density (res.abs[[ 1 ]]),  col =  cols[ 1 ],  ylim =   c ( 0 , ymax),  main =   'absolute log-transformed residuals' ) 
      abline ( v =   mean (res.cm[[ 1 ]]),  col =  cols[ 1 ]) 
      # add the rest  
      for (n  in   2  :  length (mods)){ 
        lines ( density (res.abs[[n]]),  col =  cols[n]) 
        abline ( v =   mean (res.cm[[n]]),  col =  cols[n]) 
     } 
      
      abline ( v =   0 ,  lty =   2 ) 
      legend ( 'topright' ,  
             legend =   names (mods), 
             fill =  cols[ 1  :  length (mods)]) 
      
      # reset par  
      par ( mfrow =   c ( 1 , 1 )) 
      
   }    
 
 
  3  Multi-year Model
Setup 
 
  3.1  Plot MCMT over three
years 
       # the MCMT for each year is highly correlated but shows yearly variation  
    par ( mfrow =   c ( 1 , 3 )) 
    
    plot (dat $ garden_MCMT_2021, dat $ garden_MCMT_2020,  ylim =   c ( -  16 , 15 )) 
    abline ( 0 , 1 ,  col =   'blue' ) 
    plot (dat $ garden_MCMT_2021, dat $ garden_MCMT_2022,  ylim =   c ( -  16 , 15 )) 
    abline ( 0 , 1 ,  col =   'blue' ) 
    plot (dat $ garden_MCMT_2021, dat $ garden_MCMT_2023,  ylim =   c ( -  16 , 15 )) 
    abline ( 0 , 1 ,  col =   'blue' )    
   
 
 
  3.2  Select variables and
merge years into one dataframe 
       # select the climate variables being used and merge growth values from 2021 and 2022 into the same column, adding a random effect for year  
    # to run the model using a climate variable other than MCMT, change 'colname' variables here  
    # NOT including 2023 data in this script because the loss of extreme warm and cold gardens resulted in weird response curves (eg U-shaped curves with height increasing as temperatures became more extreme)  
    # note: some effects included here were used in testing models in previous versions, but are not used in the final model  
    
    # make a subset dataframe for 2021  
    # we need to include a random effect for the individual (to account for repeated sampling) and for year  
    
    # labels  
   pheno_label  <-   'Growth Increment 2021-2022'  
   clim_label  <-   'MCMT'  
    
    # for 2021  
   garden_clim_colname  <-   'garden_MCMT_2021'  
   home_clim_colname  <-   'provenance_MCMT'  
    
    # phenotype to use  
   pheno_colname  <-   'GrowthIncrement_2021'  
    
    # setup variables  
    
    # set climate and phenotype variables  
   garden_clim  <-  dat[,garden_clim_colname] 
   garden_clim_2  <-  dat[,garden_clim_colname] ^  2  
   home_clim  <-  dat[,home_clim_colname] 
   home_clim_2  <-  dat[,home_clim_colname] ^  2  
   pheno  <-  dat[,pheno_colname] 
    
    # add other random and fixed effects  
   block  <-   as.character ( interaction (dat $ MiniCG_Site, dat $ block,  drop =  T)) 
   Pt  <-  dat $ Pt 
   indiv  <-   as.character (dat $ Unique_ID) 
   genotype  <-   as.character (dat $ Genotype) 
   garden  <-   as.character (dat $ MiniCG_Site) 
   pc1  <-  dat $ genetic_PC1 
   pc2  <-  dat $ genetic_PC2 
   pc3  <-  dat $ genetic_PC3 
   pc4  <-  dat $ genetic_PC4 
   pc5  <-  dat $ genetic_PC5 
    
    # put in df  
   df .21   <-   data.frame (pheno, garden_clim, garden_clim_2, home_clim, home_clim_2, Pt, indiv, genotype, garden, block, pc1, pc2, pc3, pc4, pc5) 
   df .21  $ year  <-   '2021'  
    str (df .21 )    
  ## 'data.frame':    1610 obs. of  16 variables:
##  $ pheno        : num  30.8 37.7 30.7 34.7 25.6 19.6 24.1 24.1 20.1 29.1 ...
##  $ garden_clim  : num  2.7 2.7 2.7 2.7 2.7 2.7 2.7 2.7 2.7 2.7 ...
##  $ garden_clim_2: num  7.29 7.29 7.29 7.29 7.29 7.29 7.29 7.29 7.29 7.29 ...
##  $ home_clim    : num  -9.6 -9.6 -9.9 -9.9 -8.9 -8.9 -9.3 -9.3 -12.2 -12.2 ...
##  $ home_clim_2  : num  92.2 92.2 98 98 79.2 ...
##  $ Pt           : num  0.571 0.571 0.908 0.908 0.864 ...
##  $ indiv        : chr  "EVERGREEN.206.1.7.4" "EVERGREEN.206.2.4.1" "EVERGREEN.210.1.7.3" "EVERGREEN.210.2.4.2" ...
##  $ genotype     : chr  "206" "206" "210" "210" ...
##  $ garden       : chr  "EVERGREEN" "EVERGREEN" "EVERGREEN" "EVERGREEN" ...
##  $ block        : chr  "EVERGREEN.1" "EVERGREEN.2" "EVERGREEN.1" "EVERGREEN.2" ...
##  $ pc1          : num  0.00423 0.00423 -0.03207 -0.03207 -0.02393 ...
##  $ pc2          : num  0.02407 0.02407 -0.00722 -0.00722 0.01811 ...
##  $ pc3          : num  -0.0243 -0.0243 -0.0365 -0.0365 -0.0505 ...
##  $ pc4          : num  0.0378 0.0378 0.0122 0.0122 0.046 ...
##  $ pc5          : num  0.00732 0.00732 0.02208 0.02208 0.01169 ...
##  $ year         : chr  "2021" "2021" "2021" "2021" ...  
       ###################################  
    # now do the same for 2022   
    
   garden_clim_colname  <-   'garden_MCMT_2022'  
   home_clim_colname  <-   'provenance_MCMT'  
    
    # phenotype to use  
   pheno_colname  <-   'GrowthIncrement_2022'  
    
    
    # setup variables  
    
    # set climate and phenotype variables  
   garden_clim  <-  dat[,garden_clim_colname] 
   garden_clim_2  <-  dat[,garden_clim_colname] ^  2  
   home_clim  <-  dat[,home_clim_colname] 
   home_clim_2  <-  dat[,home_clim_colname] ^  2  
   pheno  <-  dat[,pheno_colname] 
    
    # add other random and fixed effects  
   block  <-   as.character ( interaction (dat $ MiniCG_Site, dat $ block,  drop =  T)) 
   Pt  <-  dat $ Pt 
   indiv  <-   as.character (dat $ Unique_ID) 
   genotype  <-   as.character (dat $ Genotype) 
   garden  <-   as.character (dat $ MiniCG_Site) 
   pc1  <-  dat $ genetic_PC1 
   pc2  <-  dat $ genetic_PC2 
   pc3  <-  dat $ genetic_PC3 
   pc4  <-  dat $ genetic_PC4 
   pc5  <-  dat $ genetic_PC5 
    
    # put in df  
   df .22   <-   data.frame (pheno, garden_clim, garden_clim_2, home_clim, home_clim_2, Pt, indiv, genotype, garden, block, pc1, pc2, pc3, pc4, pc5) 
   df .22  $ year  <-   '2022'  
    str (df .22 )    
  ## 'data.frame':    1610 obs. of  16 variables:
##  $ pheno        : num  NA 10.67 8.23 39.62 77.72 ...
##  $ garden_clim  : num  3.2 3.2 3.2 3.2 3.2 3.2 3.2 3.2 3.2 3.2 ...
##  $ garden_clim_2: num  10.2 10.2 10.2 10.2 10.2 ...
##  $ home_clim    : num  -9.6 -9.6 -9.9 -9.9 -8.9 -8.9 -9.3 -9.3 -12.2 -12.2 ...
##  $ home_clim_2  : num  92.2 92.2 98 98 79.2 ...
##  $ Pt           : num  0.571 0.571 0.908 0.908 0.864 ...
##  $ indiv        : chr  "EVERGREEN.206.1.7.4" "EVERGREEN.206.2.4.1" "EVERGREEN.210.1.7.3" "EVERGREEN.210.2.4.2" ...
##  $ genotype     : chr  "206" "206" "210" "210" ...
##  $ garden       : chr  "EVERGREEN" "EVERGREEN" "EVERGREEN" "EVERGREEN" ...
##  $ block        : chr  "EVERGREEN.1" "EVERGREEN.2" "EVERGREEN.1" "EVERGREEN.2" ...
##  $ pc1          : num  0.00423 0.00423 -0.03207 -0.03207 -0.02393 ...
##  $ pc2          : num  0.02407 0.02407 -0.00722 -0.00722 0.01811 ...
##  $ pc3          : num  -0.0243 -0.0243 -0.0365 -0.0365 -0.0505 ...
##  $ pc4          : num  0.0378 0.0378 0.0122 0.0122 0.046 ...
##  $ pc5          : num  0.00732 0.00732 0.02208 0.02208 0.01169 ...
##  $ year         : chr  "2022" "2022" "2022" "2022" ...  
       # cleanup 2022  
    # remove the gardens that weren't measured in 2022  
   df .22   <-  df .22 [df .22  $ garden  %in%   levels (dat $ MiniCG_Site_2022),] 
    # remove NWMO, which is missing preflush heights  
   df .22   <-  df .22 [ !  df .22  $ garden  %in%   'NWMO' ,] 
    # remove trees that were dead in both 2021 and 2022  
   dead  <-   rownames (dat)[dat $ Survival_09_2021  ==   0   &  dat $ Survival_09_2022  ==   0 ] 
   df .22   <-  df .22 [ !  df .22  $ indiv  %in%  dead,] 
    
    
    ######################################  
    # combine 2021 and 2022  
   df  <-   merge (df .21 , df .22 ,  all =  T ) 
    str (df)    
  ## 'data.frame':    2768 obs. of  16 variables:
##  $ pheno        : num  0 0 0 0 0 0 0 0 0 0 ...
##  $ garden_clim  : num  -16.5 -16.5 -16.5 -16.5 -16.5 -16.5 -16.5 -16.5 -16.5 -16.5 ...
##  $ garden_clim_2: num  272 272 272 272 272 ...
##  $ home_clim    : num  -23.9 -20 -16.7 -16.7 -16.4 -14.6 -14.6 -14 -13.4 -12.5 ...
##  $ home_clim_2  : num  571 400 279 279 269 ...
##  $ Pt           : num  0.335 0.293 0.324 0.324 0.315 ...
##  $ indiv        : chr  "NDSU.405.1.1.24" "NDSU.411.2.4.3" "NDSU.419.1.1.20" "NDSU.419.2.3.8" ...
##  $ genotype     : chr  "405" "411" "419" "419" ...
##  $ garden       : chr  "NDSU" "NDSU" "NDSU" "NDSU" ...
##  $ block        : chr  "NDSU.1" "NDSU.2" "NDSU.1" "NDSU.2" ...
##  $ pc1          : num  0.0275 0.0313 0.0284 0.0284 0.0301 ...
##  $ pc2          : num  0.0241 0.0224 0.0258 0.0258 0.0237 ...
##  $ pc3          : num  0.0382 0.0376 0.0288 0.0288 0.0337 ...
##  $ pc4          : num  0.000561 0.012868 0.008417 0.008417 0.012894 ...
##  $ pc5          : num  0.0382 0.0428 0.03 0.03 0.0452 ...
##  $ year         : chr  "2022" "2022" "2022" "2022" ...  
       # add rownames and get list of alive individuals for each year  
    rownames (df)  <-   paste (df $ indiv, df $ year,  sep =   '_' ) 
    
    
   alive  <-   c ( paste (alive21,  '_2021' ,  sep =   '' ), 
               paste (alive22,  '_2022' ,  sep =   '' )) 
    
    dim (df)    
  ## [1] 2768   16  
       dim (df[alive,])    
  ## [1] 2308   16  
       #checks  
    
    # look at growth increment  
    par ( mfrow =   c ( 1 , 2 )) 
    hist (df $ pheno) 
    hist ( log (df $ pheno  +   1 ))    
   
       # variation across gardens/years  
    par ( mfrow =   c ( 1 , 1 ),  mar =   c ( 10 , 4 , 4 , 3 )) 
    boxplot (df $ pheno ~  df $ year  *  df $ garden,  las =   2 ,  xlab =   '' ,  col =   c ( 'lightblue' ,  'dodgerblue3' ))    
   
       #check climate - make sure home/garden climate was assigned correctly  
    boxplot (df $ garden_clim  ~  df $ year  *  df $ garden,  las =   2 ,  xlab =   '' ,  col =   c ( 'lightblue' ,  'dodgerblue3' ),  drop =  T)    
   
       boxplot (df $ home_clim  ~  df $ year  *  df $ garden,  las =   2 ,  xlab =   '' ,  col =   c ( 'lightblue' ,  'dodgerblue3' ),  drop =  T)    
   
       boxplot (df $ home_clim  ~  df $ genotype,  las =   2 ,  xlab =   '' )    
   
       # reset colnames - these get used for labeling outputs later  
   garden_clim_colname  <-   'garden_MCMT_2021-2022'  
   home_clim_colname  <-   'provenance_MCMT'  
   pheno_colname  <-   'GrowthIncrement_2021-2022'  
    
    # check correlations among variables  
    pairs.panels (df,  scale =  T)    
   
 
 
  3.3  Scale variables 
       # scale variables - having them on a similar scale improves model convergence. Previously got warnings about variables being on different scales.  
    
    dput ( colnames (df))    
  ## c("pheno", "garden_clim", "garden_clim_2", "home_clim", "home_clim_2", 
## "Pt", "indiv", "genotype", "garden", "block", "pc1", "pc2", "pc3", 
## "pc4", "pc5", "year")  
       # just scale numeric variables, then re-combine  
    
    
    # don't center - this means scale factor will be the same for each variable (column) so back-transforming is easier  
   orig  <-  df[, c ( "garden_clim" ,  "garden_clim_2" ,  "home_clim" ,  "home_clim_2" ,  "Pt" ,  "pc1" ,  "pc2" ,  "pc3" ,  "pc4" ,  "pc5" )] 
   scaled  <-   scale (orig,  center =  F) 
    
    # merge  
   df.scaled  <-   data.frame (df[,  c ( 'pheno' ,  'genotype' ,  'garden' ,  'block' ,  'indiv' ,  'year' )], scaled) 
    
    # plot  
    pairs (df.scaled[, c ( "pheno" ,  "garden_clim" ,  "garden_clim_2" ,  "home_clim" ,  "home_clim_2" ,  "Pt" ,  "pc1" ,  "pc2" ,  "pc3" ,  "pc4" ,  "pc5" )])    
   
       # get scale factors for back-transforming  
    summary (scaled / orig)    
  ##   garden_clim     garden_clim_2       home_clim        home_clim_2      
##  Min.   :0.1316   Min.   :0.01165   Min.   :0.08452   Min.   :0.005582  
##  1st Qu.:0.1316   1st Qu.:0.01165   1st Qu.:0.08452   1st Qu.:0.005582  
##  Median :0.1316   Median :0.01165   Median :0.08452   Median :0.005582  
##  Mean   :0.1316   Mean   :0.01165   Mean   :0.08452   Mean   :0.005582  
##  3rd Qu.:0.1316   3rd Qu.:0.01165   3rd Qu.:0.08452   3rd Qu.:0.005582  
##  Max.   :0.1316   Max.   :0.01165   Max.   :0.08452   Max.   :0.005582  
##                                                                         
##        Pt            pc1             pc2             pc3             pc4       
##  Min.   :1.36   Min.   :28.21   Min.   :26.29   Min.   :25.62   Min.   :38.18  
##  1st Qu.:1.36   1st Qu.:28.21   1st Qu.:26.29   1st Qu.:25.62   1st Qu.:38.18  
##  Median :1.36   Median :28.21   Median :26.29   Median :25.62   Median :38.18  
##  Mean   :1.36   Mean   :28.21   Mean   :26.29   Mean   :25.62   Mean   :38.18  
##  3rd Qu.:1.36   3rd Qu.:28.21   3rd Qu.:26.29   3rd Qu.:25.62   3rd Qu.:38.18  
##  Max.   :1.36   Max.   :28.21   Max.   :26.29   Max.   :25.62   Max.   :38.18  
##  NA's   :173    NA's   :173     NA's   :173     NA's   :173     NA's   :173    
##       pc5       
##  Min.   :27.48  
##  1st Qu.:27.48  
##  Median :27.48  
##  Mean   :27.48  
##  3rd Qu.:27.48  
##  Max.   :27.48  
##  NA's   :173  
       # get and save the first line (which is the same for all rows in a column)  
    # convert real data to scaled data: orig * scaling_factor  
    # convert scaled data to real data: scaled/scaling_factor  
   scaled_orig_df  <-  scaled / orig 
   scaling_factor  <-   as.vector (scaled_orig_df[ 1 ,])    
 
 
 
  4  Run multiyear
models 
 
  4.1  Compare setting year
as a fixed vs random effect 
 Fit a zero-inflated model predicting growth (conditional) and
mortality (zero-inflated) for each individual based on garden MCMT, home
MCMT, their square terms (accounting for parabolic response to
temperature), genetic structure represented by genetic PCs 1-3, and
interaction effects between genetic PCs and garden environment (GxE) and
home climate and garden environment (GxE when home climate is a proxy
for genetic variation due to adaptation). 
 This section compares setting year as a fixed effect vs random
effect. 
       # info about repeated measures  
    # https://stackoverflow.com/questions/63360751/specifying-random-effects-for-repeated-measures-in-logistic-mixed-model-in-r-lm  
    # https://academic.oup.com/treephys/article/37/1/33/2527789#58152734  
    
    
    # set up list to save different models to for comparison  
   mods  <-   list () 
    
    # year as RE, individual as separate RE  
    # used this one for the final version!  
    
   mods $ year_indiv_RE  <-   glmmTMB ( log (pheno  +   1 )  ~  garden_clim * home_clim  +  garden_clim_2 * home_clim_2   +  garden_clim_2  *  home_clim  +  home_clim_2 *  garden_clim  +   pc1 * garden_clim  +  pc2 * garden_clim  +  pc3 * garden_clim  +  pc1 * garden_clim_2  +  pc2 * garden_clim_2  +  pc3 * garden_clim_2  +  
                         ( 1   |  genotype)  +  ( 1   |  garden / block)  + ( 1   |  year)  +  ( 1   |  indiv), 
                   data =  df.scaled, 
                   family =   gaussian (), 
                   ziformula =   ~ .) 
    
    summary (mods $ year_indiv_RE)    
  ##  Family: gaussian  ( identity )
## Formula:          
## log(pheno + 1) ~ garden_clim * home_clim + garden_clim_2 * home_clim_2 +  
##     garden_clim_2 * home_clim + home_clim_2 * garden_clim + pc1 *  
##     garden_clim + pc2 * garden_clim + pc3 * garden_clim + pc1 *  
##     garden_clim_2 + pc2 * garden_clim_2 + pc3 * garden_clim_2 +  
##     (1 | genotype) + (1 | garden/block) + (1 | year) + (1 | indiv)
## Zero inflation:                  ~.
## Data: df.scaled
## 
##       AIC       BIC    logLik -2*log(L)  df.resid 
##    5870.5    6140.5   -2888.2    5776.5      2261 
## 
## Random effects:
## 
## Conditional model:
##  Groups       Name        Variance  Std.Dev. 
##  genotype     (Intercept) 4.577e-02 2.139e-01
##  block:garden (Intercept) 2.653e-03 5.151e-02
##  garden       (Intercept) 2.442e-01 4.942e-01
##  year         (Intercept) 5.076e-14 2.253e-07
##  indiv        (Intercept) 5.747e-02 2.397e-01
##  Residual                 4.770e-01 6.906e-01
## Number of obs: 2308, groups:  
## genotype, 44; block:garden, 35; garden, 17; year, 2; indiv, 1448
## 
## Zero-inflation model:
##  Groups       Name        Variance  Std.Dev. 
##  genotype     (Intercept) 1.849e-01 0.4300427
##  block:garden (Intercept) 2.485e-01 0.4985025
##  garden       (Intercept) 1.284e+00 1.1331755
##  year         (Intercept) 1.134e-01 0.3368161
##  indiv        (Intercept) 4.862e-08 0.0002205
##  Residual                 4.770e-01 0.6906386
## Number of obs: 2308, groups:  
## genotype, 44; block:garden, 35; garden, 17; year, 2; indiv, 1448
## 
## Dispersion estimate for gaussian family (sigma^2): 0.477 
## 
## Conditional model:
##                            Estimate Std. Error z value Pr(>|z|)    
## (Intercept)                2.850565   0.368491   7.736 1.03e-14 ***
## garden_clim               -0.278048   0.285215  -0.975 0.329625    
## home_clim                 -0.424493   0.584727  -0.726 0.467859    
## garden_clim_2             -0.842726   0.362667  -2.324 0.020142 *  
## home_clim_2               -0.538295   0.304891  -1.766 0.077474 .  
## pc1                       -0.334256   0.063319  -5.279 1.30e-07 ***
## pc2                        0.050730   0.054276   0.935 0.349963    
## pc3                       -0.182640   0.052511  -3.478 0.000505 ***
## garden_clim:home_clim      0.644678   0.467460   1.379 0.167862    
## garden_clim_2:home_clim_2  0.230843   0.290862   0.794 0.427398    
## home_clim:garden_clim_2   -0.102599   0.561409  -0.183 0.854992    
## garden_clim:home_clim_2    0.379505   0.244882   1.550 0.121203    
## garden_clim:pc1           -0.009719   0.055085  -0.176 0.859951    
## garden_clim:pc2            0.010854   0.042040   0.258 0.796258    
## garden_clim:pc3           -0.029393   0.042317  -0.695 0.487312    
## garden_clim_2:pc1          0.040787   0.063614   0.641 0.521415    
## garden_clim_2:pc2         -0.102621   0.052763  -1.945 0.051784 .  
## garden_clim_2:pc3          0.036302   0.052256   0.695 0.487247    
## ---
## Signif. codes:  0 '***' 0.001 '**' 0.01 '*' 0.05 '.' 0.1 ' ' 1
## 
## Zero-inflation model:
##                           Estimate Std. Error z value Pr(>|z|)   
## (Intercept)               -2.98943    1.11820  -2.673  0.00751 **
## garden_clim                0.05334    0.70410   0.076  0.93961   
## home_clim                 -0.06754    1.80730  -0.037  0.97019   
## garden_clim_2              2.10350    1.01858   2.065  0.03891 * 
## home_clim_2                0.14517    0.92973   0.156  0.87593   
## pc1                        0.13523    0.18918   0.715  0.47474   
## pc2                       -0.21000    0.16997  -1.236  0.21664   
## pc3                       -0.11124    0.16744  -0.664  0.50646   
## garden_clim:home_clim     -1.55053    1.11284  -1.393  0.16353   
## garden_clim_2:home_clim_2 -0.27616    0.83657  -0.330  0.74131   
## home_clim:garden_clim_2    0.15528    1.64149   0.095  0.92464   
## garden_clim:home_clim_2   -0.88899    0.59665  -1.490  0.13624   
## garden_clim:pc1            0.03859    0.12426   0.310  0.75615   
## garden_clim:pc2            0.03844    0.10329   0.372  0.70982   
## garden_clim:pc3           -0.02016    0.10204  -0.198  0.84336   
## garden_clim_2:pc1         -0.06357    0.17140  -0.371  0.71072   
## garden_clim_2:pc2          0.06635    0.15628   0.424  0.67117   
## garden_clim_2:pc3          0.20171    0.15473   1.304  0.19235   
## ---
## Signif. codes:  0 '***' 0.001 '**' 0.01 '*' 0.05 '.' 0.1 ' ' 1  
       plot_model (mods $ year_indiv_RE,  type =   'std' ,  vline.color =   "black" ,  show.values =  T)  +  
       theme ( text=  element_text ( size=  16 ))  +   
      ggtitle ( 'multiyear test' )    
   
       plot_model (mods $ year_indiv_RE,  type =   're' ,  
                      sort.est =   'sort.all' ,  
                      grid =  F,  
                      vline.color =   'black' ,  
                      wrap.title =   20 ,  wrap.labels =   20 )    
  ## [[1]]  
   
  ## 
## [[2]]  
   
  ## 
## [[3]]  
   
  ## 
## [[4]]  
   
  ## 
## [[5]]  
   
       # use genotype as  RE  
    # year as FE  
   mods $ yearFE_indivRE  <-   glmmTMB ( log (pheno  +   1 )  ~  garden_clim * home_clim  +  garden_clim_2 * home_clim_2   +  garden_clim_2  *  home_clim  +  home_clim_2 *  garden_clim  
                        +  pc1  +  pc2  +  pc3  +  pc1 * garden_clim  +  pc2 * garden_clim  +  pc3 * garden_clim  +  pc1 * garden_clim_2  +  pc2 * garden_clim_2  +  pc3 * garden_clim_2  +   
                         year  +  
                         ( 1   |  genotype)  +  ( 1   |  garden / block)  +  ( 1   |  indiv), 
                   data =  df.scaled,  
                   family =   gaussian (),  
                   ziformula =   ~ .) 
    
    
    summary (mods $ yearFE_indivRE)    
  ##  Family: gaussian  ( identity )
## Formula:          
## log(pheno + 1) ~ garden_clim * home_clim + garden_clim_2 * home_clim_2 +  
##     garden_clim_2 * home_clim + home_clim_2 * garden_clim + pc1 +  
##     pc2 + pc3 + pc1 * garden_clim + pc2 * garden_clim + pc3 *  
##     garden_clim + pc1 * garden_clim_2 + pc2 * garden_clim_2 +  
##     pc3 * garden_clim_2 + year + (1 | genotype) + (1 | garden/block) +  
##     (1 | indiv)
## Zero inflation:                  ~.
## Data: df.scaled
## 
##       AIC       BIC    logLik -2*log(L)  df.resid 
##    5865.9    6135.9   -2885.9    5771.9      2261 
## 
## Random effects:
## 
## Conditional model:
##  Groups       Name        Variance Std.Dev.
##  genotype     (Intercept) 0.045873 0.21418 
##  block:garden (Intercept) 0.002705 0.05201 
##  garden       (Intercept) 0.264741 0.51453 
##  indiv        (Intercept) 0.058652 0.24218 
##  Residual                 0.475868 0.68983 
## Number of obs: 2308, groups:  
## genotype, 44; block:garden, 35; garden, 17; indiv, 1448
## 
## Zero-inflation model:
##  Groups       Name        Variance  Std.Dev. 
##  genotype     (Intercept) 1.834e-01 0.4282750
##  block:garden (Intercept) 2.474e-01 0.4973539
##  garden       (Intercept) 1.245e+00 1.1156979
##  indiv        (Intercept) 6.139e-08 0.0002478
##  Residual                 4.759e-01 0.6898315
## Number of obs: 2308, groups:  
## genotype, 44; block:garden, 35; garden, 17; indiv, 1448
## 
## Dispersion estimate for gaussian family (sigma^2): 0.476 
## 
## Conditional model:
##                            Estimate Std. Error z value Pr(>|z|)    
## (Intercept)                2.834841   0.374438   7.571 3.71e-14 ***
## garden_clim               -0.307281   0.292874  -1.049  0.29409    
## home_clim                 -0.423082   0.585377  -0.723  0.46983    
## garden_clim_2             -0.822622   0.368850  -2.230  0.02573 *  
## home_clim_2               -0.537761   0.305211  -1.762  0.07808 .  
## pc1                       -0.334407   0.063394  -5.275 1.33e-07 ***
## pc2                        0.050759   0.054336   0.934  0.35021    
## pc3                       -0.182719   0.052572  -3.476  0.00051 ***
## year2022                  -0.020813   0.040712  -0.511  0.60919    
## garden_clim:home_clim      0.638045   0.467958   1.363  0.17274    
## garden_clim_2:home_clim_2  0.227740   0.291279   0.782  0.43430    
## home_clim:garden_clim_2   -0.105320   0.561936  -0.187  0.85133    
## garden_clim:home_clim_2    0.373885   0.245367   1.524  0.12756    
## garden_clim:pc1           -0.009645   0.055100  -0.175  0.86105    
## garden_clim:pc2            0.010119   0.042088   0.240  0.81001    
## garden_clim:pc3           -0.029049   0.042357  -0.686  0.49283    
## garden_clim_2:pc1          0.040908   0.063663   0.643  0.52050    
## garden_clim_2:pc2         -0.103257   0.052811  -1.955  0.05055 .  
## garden_clim_2:pc3          0.036781   0.052312   0.703  0.48199    
## ---
## Signif. codes:  0 '***' 0.001 '**' 0.01 '*' 0.05 '.' 0.1 ' ' 1
## 
## Zero-inflation model:
##                           Estimate Std. Error z value Pr(>|z|)    
## (Intercept)               -2.71075    1.08742  -2.493 0.012673 *  
## garden_clim                0.03231    0.70307   0.046 0.963346    
## home_clim                 -0.06441    1.80667  -0.036 0.971561    
## garden_clim_2              2.11057    1.01715   2.075 0.037989 *  
## home_clim_2                0.14461    0.92938   0.156 0.876350    
## pc1                        0.13419    0.18914   0.709 0.478043    
## pc2                       -0.21064    0.16986  -1.240 0.214945    
## pc3                       -0.11085    0.16744  -0.662 0.507934    
## year2022                  -0.61246    0.16543  -3.702 0.000214 ***
## garden_clim:home_clim     -1.56333    1.11695  -1.400 0.161623    
## garden_clim_2:home_clim_2 -0.28559    0.83816  -0.341 0.733308    
## home_clim:garden_clim_2    0.13801    1.64287   0.084 0.933053    
## garden_clim:home_clim_2   -0.89908    0.60075  -1.497 0.134497    
## garden_clim:pc1            0.03778    0.12441   0.304 0.761403    
## garden_clim:pc2            0.03757    0.10337   0.363 0.716255    
## garden_clim:pc3           -0.02014    0.10218  -0.197 0.843727    
## garden_clim_2:pc1         -0.06315    0.17146  -0.368 0.712646    
## garden_clim_2:pc2          0.06666    0.15629   0.427 0.669720    
## garden_clim_2:pc3          0.20099    0.15486   1.298 0.194331    
## ---
## Signif. codes:  0 '***' 0.001 '**' 0.01 '*' 0.05 '.' 0.1 ' ' 1  
       plot_model (mods $ yearFE_indivRE,  type =   'std' ,  vline.color =   "black" ,  show.values =  T)  +  
       theme ( text=  element_text ( size=  16 ))  +   
      ggtitle ( 'multiyear test' )    
   
       # compare models  
    plot_models (mods,  std.est =  T,  vline.color =   "black" ,  show.values =  F,  p.shape =  T,  m.labels =   names (mods))    
   
      mods.yrs  <-   list () 
    
    
    par ( mar =   c ( 5 , 4 , 4 , 3 ),  mfrow =   c ( 2 , 2 )) 
    for (n  in   1  :  length (mods)){ 
      
     mod  <-  mods[[n]] 
      
      # overall model  
      plot_predicted_vs_actual ( model_R (mod,  re.form =   NULL ),  title =   paste ( names (mods)[n],  'overall, with random effects' ,  sep =   '  \n  ' )) 
      plot_predicted_vs_actual ( model_R (mod,  re.form =   NA ),  title =   'overall, no random effects' ) 
      # conditional model (just growth)  
      # with dead trees removed  
      plot_predicted_vs_actual ( model_R (mod,  re.form =   NULL ,  type =   'conditional' ),  title =   'conditional, with random effects' ) 
      plot_predicted_vs_actual ( model_R (mod,  re.form =   NA ,  type =   'conditional' ),  title =   'conditional, no random effects' ) 
      
   }    
    
       # which model to save?  
   mod  <-  mods $ year_indiv_RE 
    
    save (mod, df.scaled, scaling_factor, 
         file =   paste ( 'results/model_prediction/glmTMB_multiyear_model_outputs_' , garden_clim_colname,  '_vs_' , pheno_colname,  '.Rda' ,  sep =   '' ))    
 
 
 
  5  Plots for selected
model 
       # which model to use?  
   mod  <-  mods $ year_indiv_RE 
    
    # print call  
   mod $ call    
  ## glmmTMB(formula = log(pheno + 1) ~ garden_clim * home_clim + 
##     garden_clim_2 * home_clim_2 + garden_clim_2 * home_clim + 
##     home_clim_2 * garden_clim + pc1 * garden_clim + pc2 * garden_clim + 
##     pc3 * garden_clim + pc1 * garden_clim_2 + pc2 * garden_clim_2 + 
##     pc3 * garden_clim_2 + (1 | genotype) + (1 | garden/block) + 
##     (1 | year) + (1 | indiv), data = df.scaled, family = gaussian(), 
##     ziformula = ~., dispformula = ~1)  
       # nice model plot using sjPlot  
    # see ?plot_model  
    # https://github.com/strengejacke/sjPlot/blob/6f57b80824575cc238834551be7270f8ecb19c8c/R/plot_type_est.R#L6  
    
    # get names of terms, excluding intercept  
   terms  <-   names ( fixef (mod)[[ 1 ]][ -  1 ]) 
   labs  <-   rep ( NA ,  length =   length (terms)) 
    # add labels for the MCMT model  
   labs  <-   c ( "Garden MCMT" ,  "Home MCMT" ,  "Garden MCMT^2" ,  "Home MCMT^2" ,  "Genetic PC1" ,  "Genetic PC2" ,  "Genetic PC3" ,  "Garden MCMT x Home MCMT" ,  "Garden MCMT^2 x Home MCMT^2" ,  "Garden MCMT^2 x Home MCMT" ,  "Garden MCMT x Home MCMT^2" ,  "Garden MCMT x Genetic PC1" ,  "Garden MCMT x Genetic PC2" ,  "Garden MCMT x Genetic PC3" ,  "Garden MCMT^2 x Genetic PC1" ,  "Garden MCMT^2 x Genetic PC2" ,  "Garden MCMT^2 x Genetic PC3" ) 
    names (labs)  <-  terms 
    
    # check that they match  
   labs    
  ##                   garden_clim                     home_clim 
##                 "Garden MCMT"                   "Home MCMT" 
##                 garden_clim_2                   home_clim_2 
##               "Garden MCMT^2"                 "Home MCMT^2" 
##                           pc1                           pc2 
##                 "Genetic PC1"                 "Genetic PC2" 
##                           pc3         garden_clim:home_clim 
##                 "Genetic PC3"     "Garden MCMT x Home MCMT" 
##     garden_clim_2:home_clim_2       home_clim:garden_clim_2 
## "Garden MCMT^2 x Home MCMT^2"   "Garden MCMT^2 x Home MCMT" 
##       garden_clim:home_clim_2               garden_clim:pc1 
##   "Garden MCMT x Home MCMT^2"   "Garden MCMT x Genetic PC1" 
##               garden_clim:pc2               garden_clim:pc3 
##   "Garden MCMT x Genetic PC2"   "Garden MCMT x Genetic PC3" 
##             garden_clim_2:pc1             garden_clim_2:pc2 
## "Garden MCMT^2 x Genetic PC1" "Garden MCMT^2 x Genetic PC2" 
##             garden_clim_2:pc3 
## "Garden MCMT^2 x Genetic PC3"  
       cbind (labs,  names (labs))    
  ##                           labs                         
## garden_clim               "Garden MCMT"                
## home_clim                 "Home MCMT"                  
## garden_clim_2             "Garden MCMT^2"              
## home_clim_2               "Home MCMT^2"                
## pc1                       "Genetic PC1"                
## pc2                       "Genetic PC2"                
## pc3                       "Genetic PC3"                
## garden_clim:home_clim     "Garden MCMT x Home MCMT"    
## garden_clim_2:home_clim_2 "Garden MCMT^2 x Home MCMT^2"
## home_clim:garden_clim_2   "Garden MCMT^2 x Home MCMT"  
## garden_clim:home_clim_2   "Garden MCMT x Home MCMT^2"  
## garden_clim:pc1           "Garden MCMT x Genetic PC1"  
## garden_clim:pc2           "Garden MCMT x Genetic PC2"  
## garden_clim:pc3           "Garden MCMT x Genetic PC3"  
## garden_clim_2:pc1         "Garden MCMT^2 x Genetic PC1"
## garden_clim_2:pc2         "Garden MCMT^2 x Genetic PC2"
## garden_clim_2:pc3         "Garden MCMT^2 x Genetic PC3"
##                                                      
## garden_clim               "garden_clim"              
## home_clim                 "home_clim"                
## garden_clim_2             "garden_clim_2"            
## home_clim_2               "home_clim_2"              
## pc1                       "pc1"                      
## pc2                       "pc2"                      
## pc3                       "pc3"                      
## garden_clim:home_clim     "garden_clim:home_clim"    
## garden_clim_2:home_clim_2 "garden_clim_2:home_clim_2"
## home_clim:garden_clim_2   "home_clim:garden_clim_2"  
## garden_clim:home_clim_2   "garden_clim:home_clim_2"  
## garden_clim:pc1           "garden_clim:pc1"          
## garden_clim:pc2           "garden_clim:pc2"          
## garden_clim:pc3           "garden_clim:pc3"          
## garden_clim_2:pc1         "garden_clim_2:pc1"        
## garden_clim_2:pc2         "garden_clim_2:pc2"        
## garden_clim_2:pc3         "garden_clim_2:pc3"  
       # reorder effect names   
   ord  <-   c ( 1 , 3 , 2 , 4 , 5  :  17 ) 
    
    # png(file = paste('results/model_prediction/glmTMB_model_plot_standardized_', garden_clim_colname, '_vs_', pheno_colname, '.png', sep = ''),  
    # height = 12,  
    # width = 9,  
    # res = 300,  
    # units = 'in')  
    
    plot_model (mod,  type =   'std' ,  vline.color =   "black" ,  show.values =  T,  value.offset =   0.4 ,  wrap.labels =   30 ,  dot.size =   2 )  +  
       theme ( text=  element_text ( size=  16 ))  +  
      ggtitle ( 'Annual Growth Increment (log transformed)' )  +  
      scale_x_discrete ( limits =   rev ( names (labs)[ord]),  labels =  labs[ord])    
  ## Scale for x is already present.
## Adding another scale for x, which will replace the existing scale.  
   
       #dev.off()  
    
    
    
    # plot predicted vs actual height values  
    # info on including random effects: https://stats.stackexchange.com/questions/191648/using-re-form-in-predict-mermod-for-a-lmer-model  
    
    
    # png(file = paste('results/model_prediction/glmTMB_full_model_actual_vs_predicted_', garden_clim_colname, '_vs_', pheno_colname, '.png', sep = ''),  
    #     height = 10,  
    #     width = 10,  
    #     res = 300,  
    #     units = 'in')  
    
    par ( mar =   c ( 5 , 4 , 4 , 3 ),  mfrow =   c ( 2 , 2 )) 
    plot_predicted_vs_actual ( model_R (mod,  re.form =   NULL ),  title =   'overall, with random effects' ) 
    plot_predicted_vs_actual ( model_R (mod,  re.form =   NA ),  title =   'overall, no random effects' ) 
    plot_predicted_vs_actual ( model_R (mod,  re.form =   NULL ,  type =   'conditional' ,),  title =   'conditional, with random effects' ) 
    plot_predicted_vs_actual ( model_R (mod,  re.form =   NA ,  type =   'conditional' ,),  title =   'conditional, no random effects' )    
   
       #dev.off()  
    
    # looking at model  
    # see ?Anova.glmmTMB  
    # anova: https://stats.stackexchange.com/questions/60362/choice-between-type-i-type-ii-or-type-iii-anova  
    summary (mod)    
  ##  Family: gaussian  ( identity )
## Formula:          
## log(pheno + 1) ~ garden_clim * home_clim + garden_clim_2 * home_clim_2 +  
##     garden_clim_2 * home_clim + home_clim_2 * garden_clim + pc1 *  
##     garden_clim + pc2 * garden_clim + pc3 * garden_clim + pc1 *  
##     garden_clim_2 + pc2 * garden_clim_2 + pc3 * garden_clim_2 +  
##     (1 | genotype) + (1 | garden/block) + (1 | year) + (1 | indiv)
## Zero inflation:                  ~.
## Data: df.scaled
## 
##       AIC       BIC    logLik -2*log(L)  df.resid 
##    5870.5    6140.5   -2888.2    5776.5      2261 
## 
## Random effects:
## 
## Conditional model:
##  Groups       Name        Variance  Std.Dev. 
##  genotype     (Intercept) 4.577e-02 2.139e-01
##  block:garden (Intercept) 2.653e-03 5.151e-02
##  garden       (Intercept) 2.442e-01 4.942e-01
##  year         (Intercept) 5.076e-14 2.253e-07
##  indiv        (Intercept) 5.747e-02 2.397e-01
##  Residual                 4.770e-01 6.906e-01
## Number of obs: 2308, groups:  
## genotype, 44; block:garden, 35; garden, 17; year, 2; indiv, 1448
## 
## Zero-inflation model:
##  Groups       Name        Variance  Std.Dev. 
##  genotype     (Intercept) 1.849e-01 0.4300427
##  block:garden (Intercept) 2.485e-01 0.4985025
##  garden       (Intercept) 1.284e+00 1.1331755
##  year         (Intercept) 1.134e-01 0.3368161
##  indiv        (Intercept) 4.862e-08 0.0002205
##  Residual                 4.770e-01 0.6906386
## Number of obs: 2308, groups:  
## genotype, 44; block:garden, 35; garden, 17; year, 2; indiv, 1448
## 
## Dispersion estimate for gaussian family (sigma^2): 0.477 
## 
## Conditional model:
##                            Estimate Std. Error z value Pr(>|z|)    
## (Intercept)                2.850565   0.368491   7.736 1.03e-14 ***
## garden_clim               -0.278048   0.285215  -0.975 0.329625    
## home_clim                 -0.424493   0.584727  -0.726 0.467859    
## garden_clim_2             -0.842726   0.362667  -2.324 0.020142 *  
## home_clim_2               -0.538295   0.304891  -1.766 0.077474 .  
## pc1                       -0.334256   0.063319  -5.279 1.30e-07 ***
## pc2                        0.050730   0.054276   0.935 0.349963    
## pc3                       -0.182640   0.052511  -3.478 0.000505 ***
## garden_clim:home_clim      0.644678   0.467460   1.379 0.167862    
## garden_clim_2:home_clim_2  0.230843   0.290862   0.794 0.427398    
## home_clim:garden_clim_2   -0.102599   0.561409  -0.183 0.854992    
## garden_clim:home_clim_2    0.379505   0.244882   1.550 0.121203    
## garden_clim:pc1           -0.009719   0.055085  -0.176 0.859951    
## garden_clim:pc2            0.010854   0.042040   0.258 0.796258    
## garden_clim:pc3           -0.029393   0.042317  -0.695 0.487312    
## garden_clim_2:pc1          0.040787   0.063614   0.641 0.521415    
## garden_clim_2:pc2         -0.102621   0.052763  -1.945 0.051784 .  
## garden_clim_2:pc3          0.036302   0.052256   0.695 0.487247    
## ---
## Signif. codes:  0 '***' 0.001 '**' 0.01 '*' 0.05 '.' 0.1 ' ' 1
## 
## Zero-inflation model:
##                           Estimate Std. Error z value Pr(>|z|)   
## (Intercept)               -2.98943    1.11820  -2.673  0.00751 **
## garden_clim                0.05334    0.70410   0.076  0.93961   
## home_clim                 -0.06754    1.80730  -0.037  0.97019   
## garden_clim_2              2.10350    1.01858   2.065  0.03891 * 
## home_clim_2                0.14517    0.92973   0.156  0.87593   
## pc1                        0.13523    0.18918   0.715  0.47474   
## pc2                       -0.21000    0.16997  -1.236  0.21664   
## pc3                       -0.11124    0.16744  -0.664  0.50646   
## garden_clim:home_clim     -1.55053    1.11284  -1.393  0.16353   
## garden_clim_2:home_clim_2 -0.27616    0.83657  -0.330  0.74131   
## home_clim:garden_clim_2    0.15528    1.64149   0.095  0.92464   
## garden_clim:home_clim_2   -0.88899    0.59665  -1.490  0.13624   
## garden_clim:pc1            0.03859    0.12426   0.310  0.75615   
## garden_clim:pc2            0.03844    0.10329   0.372  0.70982   
## garden_clim:pc3           -0.02016    0.10204  -0.198  0.84336   
## garden_clim_2:pc1         -0.06357    0.17140  -0.371  0.71072   
## garden_clim_2:pc2          0.06635    0.15628   0.424  0.67117   
## garden_clim_2:pc3          0.20171    0.15473   1.304  0.19235   
## ---
## Signif. codes:  0 '***' 0.001 '**' 0.01 '*' 0.05 '.' 0.1 ' ' 1  
       # create model table (using sjPlot package)  
    # on standardizing (from ?tab.model):  
    # "Default standardization is done by completely refitting the model on the standardized data. Hence, this approach is equal to standardizing the variables before fitting the model, which is particularly recommended for complex models that include interactions or transformations (e.g., polynomial or spline terms)."  
    tab_model (mod,  show.std =  T,  show.aic =  T)    
  ## Warning: Could not compute corrected log-likelihood for models with transformed
##   response. Log-likelihood value is probably inaccurate.  
 
 
 
 
 
 
log(pheno + 1)
 
 
 
 
Predictors
 
 
Estimates
 
 
std. Beta
 
 
CI
 
 
standardized CI
 
 
p
 
 
std. p
 
 
 
 
Count Model
 
 
 
 
(Intercept)
 
 
2.85
 
 
2.73
 
 
2.13 – 3.57
 
 
2.48 – 2.98
 
 
 <0.001 
 
 
 <0.001 
 
 
 
 
garden clim
 
 
-0.28
 
 
-0.53
 
 
-0.84 – 0.28
 
 
-0.74 – -0.31
 
 
0.330
 
 
 <0.001 
 
 
 
 
home clim
 
 
-0.42
 
 
-0.27
 
 
-1.57 – 0.72
 
 
-0.65 – 0.11
 
 
0.468
 
 
0.169
 
 
 
 
garden clim 2
 
 
-0.84
 
 
-0.46
 
 
-1.55 – -0.13
 
 
-0.74 – -0.18
 
 
 0.020 
 
 
 0.001 
 
 
 
 
home clim 2
 
 
-0.54
 
 
-0.33
 
 
-1.14 – 0.06
 
 
-0.68 – 0.01
 
 
0.077
 
 
0.060
 
 
 
 
pc1
 
 
-0.33
 
 
-0.30
 
 
-0.46 – -0.21
 
 
-0.41 – -0.18
 
 
 <0.001 
 
 
 <0.001 
 
 
 
 
pc2
 
 
0.05
 
 
-0.02
 
 
-0.06 – 0.16
 
 
-0.12 – 0.07
 
 
0.350
 
 
0.660
 
 
 
 
pc3
 
 
-0.18
 
 
-0.15
 
 
-0.29 – -0.08
 
 
-0.24 – -0.05
 
 
 0.001 
 
 
 0.003 
 
 
 
 
garden clim × home clim
 
 
0.64
 
 
0.21
 
 
-0.27 – 1.56
 
 
-0.09 – 0.51
 
 
0.168
 
 
0.168
 
 
 
 
garden clim 2 × home clim 2
 
 
0.23
 
 
0.11
 
 
-0.34 – 0.80
 
 
-0.16 – 0.37
 
 
0.427
 
 
0.427
 
 
 
 
home clim × garden clim 2
 
 
-0.10
 
 
-0.03
 
 
-1.20 – 1.00
 
 
-0.32 – 0.27
 
 
0.855
 
 
0.855
 
 
 
 
garden clim × home clim 2
 
 
0.38
 
 
0.21
 
 
-0.10 – 0.86
 
 
-0.06 – 0.49
 
 
0.121
 
 
0.121
 
 
 
 
garden clim × pc1
 
 
-0.01
 
 
-0.01
 
 
-0.12 – 0.10
 
 
-0.11 – 0.09
 
 
0.860
 
 
0.860
 
 
 
 
garden clim × pc2
 
 
0.01
 
 
0.01
 
 
-0.07 – 0.09
 
 
-0.06 – 0.08
 
 
0.796
 
 
0.796
 
 
 
 
garden clim × pc3
 
 
-0.03
 
 
-0.03
 
 
-0.11 – 0.05
 
 
-0.10 – 0.05
 
 
0.487
 
 
0.487
 
 
 
 
garden clim 2 × pc1
 
 
0.04
 
 
0.03
 
 
-0.08 – 0.17
 
 
-0.06 – 0.12
 
 
0.521
 
 
0.521
 
 
 
 
garden clim 2 × pc2
 
 
-0.10
 
 
-0.07
 
 
-0.21 – 0.00
 
 
-0.15 – 0.00
 
 
0.052
 
 
0.052
 
 
 
 
garden clim 2 × pc3
 
 
0.04
 
 
0.03
 
 
-0.07 – 0.14
 
 
-0.05 – 0.10
 
 
0.487
 
 
0.487
 
 
 
 
(Intercept)
 
 
0.69
 
 
0.69
 
 
0.65 – 0.73
 
 
0.65 – 0.73
 
 
 
 
 
 
 
 
Zero-Inflated Model
 
 
 
 
(Intercept)
 
 
-2.99
 
 
-2.03
 
 
-5.18 – -0.80
 
 
-2.81 – -1.26
 
 
 0.008 
 
 
 <0.001 
 
 
 
 
garden clim
 
 
0.05
 
 
0.74
 
 
-1.33 – 1.43
 
 
0.10 – 1.37
 
 
0.940
 
 
 0.023 
 
 
 
 
home clim
 
 
-0.07
 
 
0.24
 
 
-3.61 – 3.47
 
 
-0.74 – 1.21
 
 
0.970
 
 
0.636
 
 
 
 
garden clim 2
 
 
2.10
 
 
1.34
 
 
0.11 – 4.10
 
 
0.72 – 1.95
 
 
 0.039 
 
 
 <0.001 
 
 
 
 
home clim 2
 
 
0.15
 
 
0.20
 
 
-1.68 – 1.97
 
 
-0.69 – 1.08
 
 
0.876
 
 
0.663
 
 
 
 
pc1
 
 
0.14
 
 
0.08
 
 
-0.24 – 0.51
 
 
-0.21 – 0.36
 
 
0.475
 
 
0.604
 
 
 
 
pc2
 
 
-0.21
 
 
-0.17
 
 
-0.54 – 0.12
 
 
-0.42 – 0.07
 
 
0.217
 
 
0.163
 
 
 
 
pc3
 
 
-0.11
 
 
0.03
 
 
-0.44 – 0.22
 
 
-0.22 – 0.28
 
 
0.506
 
 
0.803
 
 
 
 
garden clim × home clim
 
 
-1.55
 
 
-0.51
 
 
-3.73 – 0.63
 
 
-1.22 – 0.21
 
 
0.164
 
 
0.164
 
 
 
 
garden clim 2 × home clim 2
 
 
-0.28
 
 
-0.13
 
 
-1.92 – 1.36
 
 
-0.89 – 0.64
 
 
0.741
 
 
0.741
 
 
 
 
home clim × garden clim 2
 
 
0.16
 
 
0.04
 
 
-3.06 – 3.37
 
 
-0.82 – 0.91
 
 
0.925
 
 
0.925
 
 
 
 
garden clim × home clim 2
 
 
-0.89
 
 
-0.50
 
 
-2.06 – 0.28
 
 
-1.16 – 0.16
 
 
0.136
 
 
0.136
 
 
 
 
garden clim × pc1
 
 
0.04
 
 
0.03
 
 
-0.20 – 0.28
 
 
-0.18 – 0.25
 
 
0.756
 
 
0.756
 
 
 
 
garden clim × pc2
 
 
0.04
 
 
0.03
 
 
-0.16 – 0.24
 
 
-0.14 – 0.21
 
 
0.710
 
 
0.710
 
 
 
 
garden clim × pc3
 
 
-0.02
 
 
-0.02
 
 
-0.22 – 0.18
 
 
-0.20 – 0.16
 
 
0.843
 
 
0.843
 
 
 
 
garden clim 2 × pc1
 
 
-0.06
 
 
-0.05
 
 
-0.40 – 0.27
 
 
-0.30 – 0.20
 
 
0.711
 
 
0.711
 
 
 
 
garden clim 2 × pc2
 
 
0.07
 
 
0.05
 
 
-0.24 – 0.37
 
 
-0.17 – 0.27
 
 
0.671
 
 
0.671
 
 
 
 
garden clim 2 × pc3
 
 
0.20
 
 
0.15
 
 
-0.10 – 0.50
 
 
-0.08 – 0.38
 
 
0.192
 
 
0.192
 
 
 
 
Random Effects
 
 
 
 
σ 2 
 
 
0.69
 
 
 
 
τ 00   genotype 
 
 
0.05
 
 
 
τ 00   block:garden 
 
 
0.00
 
 
 
τ 00   garden 
 
 
0.24
 
 
 
τ 00   year 
 
 
0.00
 
 
 
τ 00   indiv 
 
 
0.06
 
 
 
N  genotype 
 
 
44
 
 
 
N  block 
 
 
35
 
 
 
N  garden 
 
 
17
 
 
 
N  year 
 
 
2
 
 
 
N  indiv 
 
 
1448
 
 
 
Observations
 
 
2308
 
 
 
 
Marginal R 2  / Conditional R 2 
 
 
0.348 / NA
 
 
 
 
AIC
 
 
5870.497
 
 
 
       # save  
    # tab_model(mod, show.std = T, show.aic = T,  
    #           file = paste('results/model_prediction/glmTMB_model_anova_table_', garden_clim_colname, '_vs_', pheno_colname, '.html', sep = ''))  
    
    # these produce the same results  
    # car::Anova(mod, component = "cond", type = 2)  
    # car::Anova(mod, component = "zi", type = 2)  
   glmmTMB :::  Anova.glmmTMB (mod,  component =   'cond' ,  type =   2 )    
  ## Analysis of Deviance Table (Type II Wald chisquare tests)
## 
## Response: log(pheno + 1)
##                             Chisq Df Pr(>Chisq)    
## garden_clim               21.6483  1  3.275e-06 ***
## home_clim                  1.8363  1   0.175387    
## garden_clim_2             10.4214  1   0.001246 ** 
## home_clim_2                3.9232  1   0.047625 *  
## pc1                       27.4360  1  1.624e-07 ***
## pc2                        0.0352  1   0.851119    
## pc3                        9.6518  1   0.001892 ** 
## garden_clim:home_clim      1.9019  1   0.167862    
## garden_clim_2:home_clim_2  0.6299  1   0.427398    
## home_clim:garden_clim_2    0.0334  1   0.854992    
## garden_clim:home_clim_2    2.4017  1   0.121203    
## garden_clim:pc1            0.0311  1   0.859951    
## garden_clim:pc2            0.0667  1   0.796258    
## garden_clim:pc3            0.4825  1   0.487312    
## garden_clim_2:pc1          0.4111  1   0.521415    
## garden_clim_2:pc2          3.7827  1   0.051784 .  
## garden_clim_2:pc3          0.4826  1   0.487247    
## ---
## Signif. codes:  0 '***' 0.001 '**' 0.01 '*' 0.05 '.' 0.1 ' ' 1  
      glmmTMB :::  Anova.glmmTMB (mod,  component =   'zi' ,  type =   2 )    
  ## Analysis of Deviance Table (Type II Wald chisquare tests)
## 
## Response: log(pheno + 1)
##                             Chisq Df Pr(>Chisq)    
## garden_clim                5.0332  1    0.02487 *  
## home_clim                  0.2057  1    0.65012    
## garden_clim_2             16.7801  1  4.197e-05 ***
## home_clim_2                0.1185  1    0.73068    
## pc1                        0.2068  1    0.64931    
## pc2                        1.7650  1    0.18400    
## pc3                        0.2588  1    0.61092    
## garden_clim:home_clim      1.9413  1    0.16353    
## garden_clim_2:home_clim_2  0.1090  1    0.74131    
## home_clim:garden_clim_2    0.0089  1    0.92464    
## garden_clim:home_clim_2    2.2200  1    0.13624    
## garden_clim:pc1            0.0964  1    0.75615    
## garden_clim:pc2            0.1385  1    0.70982    
## garden_clim:pc3            0.0390  1    0.84336    
## garden_clim_2:pc1          0.1376  1    0.71072    
## garden_clim_2:pc2          0.1802  1    0.67117    
## garden_clim_2:pc3          1.6995  1    0.19235    
## ---
## Signif. codes:  0 '***' 0.001 '**' 0.01 '*' 0.05 '.' 0.1 ' ' 1  
       # print fixed and random effects  
    fixef (mod)    
  ## 
## Conditional model:
##               (Intercept)                garden_clim  
##                  2.850565                  -0.278048  
##                 home_clim              garden_clim_2  
##                 -0.424493                  -0.842726  
##               home_clim_2                        pc1  
##                 -0.538295                  -0.334256  
##                       pc2                        pc3  
##                  0.050730                  -0.182640  
##     garden_clim:home_clim  garden_clim_2:home_clim_2  
##                  0.644678                   0.230843  
##   home_clim:garden_clim_2    garden_clim:home_clim_2  
##                 -0.102599                   0.379505  
##           garden_clim:pc1            garden_clim:pc2  
##                 -0.009719                   0.010854  
##           garden_clim:pc3          garden_clim_2:pc1  
##                 -0.029393                   0.040787  
##         garden_clim_2:pc2          garden_clim_2:pc3  
##                 -0.102621                   0.036302  
## 
## Zero-inflation model:
##               (Intercept)                garden_clim  
##                  -2.98943                    0.05334  
##                 home_clim              garden_clim_2  
##                  -0.06754                    2.10350  
##               home_clim_2                        pc1  
##                   0.14517                    0.13523  
##                       pc2                        pc3  
##                  -0.21000                   -0.11124  
##     garden_clim:home_clim  garden_clim_2:home_clim_2  
##                  -1.55053                   -0.27616  
##   home_clim:garden_clim_2    garden_clim:home_clim_2  
##                   0.15528                   -0.88899  
##           garden_clim:pc1            garden_clim:pc2  
##                   0.03859                    0.03844  
##           garden_clim:pc3          garden_clim_2:pc1  
##                  -0.02016                   -0.06357  
##         garden_clim_2:pc2          garden_clim_2:pc3  
##                   0.06635                    0.20171  
       #ranef(mod) # skipping this because the RE for each individual prints a lot of text   
    
    # plot random effects  
    # plotting together doesn't save well, plot them all separately then combine later  
   res  <-   plot_model (mod,  type =   're' ,  
                      sort.est =   'sort.all' ,  
                      grid =  F,  
                      vline.color =   'black' ,  
                      wrap.title =   20 ,  wrap.labels =   20 ) 
    
    plot (res[[ 3 ]])  +  
      labs ( tag =   'A' )    
    
       #ggsave(paste('results/model_prediction/glmTMB_model_randomEffects_garden_', garden_clim_colname, '_vs_', pheno_colname, '.png', sep = ''),  
    #             height = 9, width = 8)  
    
    plot (res[[ 2 ]])  +  
      labs ( tag =   'B' )    
    
       #ggsave(paste('results/model_prediction/glmTMB_model_randomEffects_block_garden_', garden_clim_colname, '_vs_', pheno_colname, '.png', sep = ''),  
    #            height = 9, width = 8)  
    
    plot (res[[ 1 ]])  +  
      labs ( tag =   'C' )    
    
       #ggsave(paste('results/model_prediction/glmTMB_model_randomEffects_genotype_', garden_clim_colname, '_vs_', pheno_colname, '.png', sep = ''),  
    #             height = 9, width = 8)  
    
    plot (res[[ 4 ]])  +  
      labs ( tag =   'D' )    
    
       #ggsave(paste('results/model_prediction/glmTMB_model_randomEffects_year_', garden_clim_colname, '_vs_', pheno_colname, '.png', sep = ''),  
    #            height = 9, width = 8)  
    
    
    # save individual random effects save as text - too big to easily visualize on plot  
   res  <-   ranef (mod) 
    #write.csv(res$cond$indiv, file = paste('results/model_prediction/glmTMB_model_randomEffects_conditional_individual_', garden_clim_colname, '_vs_', pheno_colname, '.csv', sep = ''))  
    #write.csv(res$zi$indiv, file = paste('results/model_prediction/glmTMB_model_randomEffects_zero-inflated_individual_', garden_clim_colname, '_vs_', pheno_colname, '.csv', sep = ''))  
    
    
    ###############  
    # plots of residuals  
    # do residuals vary by home climate?  
    par ( mfrow =   c ( 1 , 1 )) 
    plot (mod $ frame $ home_clim[ 1  :  44 ], res $ cond $ genotype $  `  (Intercept)  ` )    
   
       # plots of residuals  
    # distribution  
    hist ( residuals (mod))    
   
       # residuals for each effect  
    plot_model (mod,  type =   'resid' )    
  ## Warning: Interaction terms are not supported by this plot type. Output for
## interaction terms may be inappropriate.  
  ## `geom_smooth()` using formula = 'y ~ x'
## `geom_smooth()` using formula = 'y ~ x'  
   
       plot_residuals (mod,  show.pred =  F)    
  ## `geom_smooth()` using formula = 'y ~ x'  
   
       # residuals vs height  
    #plot(pheno[! is.na(pheno)], residuals(mod))  
    hist ( residuals (mod))    
   
       # check model assumptions  
    # "Note: For mixed models, the diagnostic plots like linear relationship or check for Homoscedasticity, do not take the uncertainty of random effects into account, but is only based on the fixed effects part of the model."  
    # "For linear (mixed) models, plots for multicollinearity-check (Variance Inflation Factors), QQ-plots, checks for normal distribution of residuals and homoscedasticity (constant variance of residuals) are shown. For generalized linear mixed models, returns the QQ-plot for random effects."  
    plot_model (mod,  type =   'diag' )    
  ## $genotype  
  ## `geom_smooth()` using formula = 'y ~ x'  
   
  ## 
## $`block:garden`  
  ## `geom_smooth()` using formula = 'y ~ x'  
   
  ## 
## $garden  
  ## `geom_smooth()` using formula = 'y ~ x'  
   
  ## 
## $year  
  ## `geom_smooth()` using formula = 'y ~ x'  
  ## Warning in qt((1 - level)/2, df): NaNs produced  
  ## Warning in max(ids, na.rm = TRUE): no non-missing arguments to max; returning
## -Inf  
   
  ## 
## $indiv  
  ## `geom_smooth()` using formula = 'y ~ x'  
   
       # model fit   
    sjp.poly (mod,  poly.term =   'garden_clim' ,  poly.degree =   2 )    
  ## Polynomial degrees: 2
## ---------------------
## p(x^1): 0.000
## p(x^2): 0.000  
  ## `geom_smooth()` using formula = 'y ~ x'  
   
       # visualize effects of factors on growth  
    plot_model (mod,  type =   'pred' ,  terms =   'pc1' )    
  ## You are calculating adjusted predictions on the population-level (i.e.
##   `type = "fixed"`) for a *generalized* linear mixed model.
##   This may produce biased estimates due to Jensen's inequality. Consider
##   setting `bias_correction = TRUE` to correct for this bias.
##   See also the documentation of the `bias_correction` argument.
## Model has log(x+1)-transformed response. Back-transforming predictions
##   to original response scale. Standard errors are still on the transformed
##   scale.  
   
       plot_model (mod,  type =   'pred' ,  terms =   'pc2' )    
  ## Model has log(x+1)-transformed response. Back-transforming predictions
##   to original response scale. Standard errors are still on the transformed
##   scale.  
   
       plot_model (mod,  type =   'pred' ,  terms =   'pc3' )    
  ## Model has log(x+1)-transformed response. Back-transforming predictions
##   to original response scale. Standard errors are still on the transformed
##   scale.  
   
       plot_model (mod,  type =   'pred' ,  terms =   c ( 'pc1' ,  'pc3' ))    
  ## Model has log(x+1)-transformed response. Back-transforming predictions
##   to original response scale. Standard errors are still on the transformed
##   scale.  
   
       plot_model (mod,  type =   'pred' ,  terms =   'home_clim' )    
  ## Model has log(x+1)-transformed response. Back-transforming predictions
##   to original response scale. Standard errors are still on the transformed
##   scale.  
   
       plot_model (mod,  type =   'pred' ,  terms =   'home_clim_2' )    
  ## Model has log(x+1)-transformed response. Back-transforming predictions
##   to original response scale. Standard errors are still on the transformed
##   scale.  
   
       plot_model (mod,  type =   'pred' ,  terms =   c ( 'home_clim' ,  'garden_clim' ))    
  ## Model has log(x+1)-transformed response. Back-transforming predictions
##   to original response scale. Standard errors are still on the transformed
##   scale.  
   
       # correlation of random effects from the conditional and zero-inflated model  
    par ( mfrow =   c ( 1 , 1 )) 
    plot ( ranef (mod) $ cond $ garden[, 1 ],  ranef (mod) $ zi $ garden[, 1 ])    
   
       plot ( ranef (mod) $ cond $ genotype[, 1 ],  ranef (mod) $ zi $ genotype[, 1 ])    
   
       # plot genetic PCs, colored by species ancestry  
    
    par ( mfrow =   c ( 1 , 2 )) 
    # label by transect  
    plot (dat $ genetic_PC1, dat $ genetic_PC3,  type =   'n' ) 
    text (dat $ genetic_PC1, dat $ genetic_PC3, dat $ transect,  col =  dat $ color_Pt) 
    # label by genotyoe  
    plot (dat $ genetic_PC1, dat $ genetic_PC3,  type =   'n' ) 
    text (dat $ genetic_PC1, dat $ genetic_PC3, dat $ Genotype,  col =  dat $ color_Pt)    
   
       ##########################  
    # plot actual vs predicted height for different subsets of data   
    
    # each genotype separately  
    # when random effects are included  
    # use se.fit = T to get standard errors  
   pred  <-   predict (mod,  type =   'response' ,  se.fit =  F) 
    # un-log-transform  
   pred  <-   exp (pred)  -   1  
    
    
   genos  <-   sort ( unique (df.scaled $ genotype)) 
    
    # use only data with no NAs (lm removes this automatically)  
   noNA  <-  df.scaled[, c ( "pheno" ,  "garden_clim" ,  "garden_clim_2" ,  "home_clim" ,  "home_clim_2" ,  "genotype" ,  "garden" ,  "year" ,  "pc1" ,  "pc2" ,  "pc3" )] 
   noNA  <-  noNA[ complete.cases (noNA),] 
    
    par ( mfrow =   c ( 4 , 4 )) 
   genos_r  <-   rep ( NA ,  length (genos)) 
    names (genos_r)  <-  genos 
   genos_p  <-   rep ( NA ,  length (genos)) 
    names (genos_p)  <-  genos 
    
    for (n  in   1  :  length (genos)){ 
      
     geno  <-  genos[n] 
      
     keep  <-   which (noNA $ genotype  ==  geno) 
      
      if ( length (keep)  >   0 ){ 
        
       cortest  <-   cor.test (noNA $ pheno[keep], pred[keep]) 
       pval  <-   round (cortest $ p.value,  4 ) 
       Rval  <-   round (cortest $ estimate,  3 ) 
        
        #par(mfrow = c(1,2))  
        plot (noNA $ pheno[keep], pred[keep],  pch =   16 ,  col =   rgb ( 0 , 0 , 0 , 0.4 ),  
             main =   paste (  'genotype ' , geno,  '  \n  ' ,  'R = ' , Rval,  '  |  pval = ' , pval,  sep =   '' ),  
             xlab =   "Height (actual)" ,  
             ylab =   "Height (predicted)" ) 
        abline ( 0 ,  1 ,  col =   'red' ,  lty =   2 )  # 1to1 line  
        abline ( lm (pred[keep]  ~  noNA $ pheno[keep]),  col =   'blue' )  # fit line  
        
       genos_r[geno]  <-  Rval 
       genos_p[geno]  <-  pval 
        
     }   
    
   }    
    
       par ( mfrow =   c ( 1 , 2 ))    
   
       hist (genos_r) 
    hist (genos_r ^  2 )    
   
       # plot each garden separately  
    
   gards  <-   unique (df.scaled $ garden) 
    
    par ( mfrow =   c ( 4 , 5 )) 
   gards_r  <-   rep ( NA ,  length (gards)) 
    names (gards_r)  <-  gards 
    
    
    for (n  in   1  :  length (gards)){ 
      
     gard  <-  gards[n] 
      
      # use only data with no NAs (lm removes this automatically)  
     noNA  <-  df.scaled[, c ( "pheno" ,  "garden_clim" ,  "garden_clim_2" ,  "home_clim" ,  "home_clim_2" ,  "genotype" ,  "garden" ,  "year" ,  "pc1" ,  "pc2" ,  "pc3" )] 
     noNA  <-  noNA[ complete.cases (noNA),] 
      
     keep  <-   which (noNA $ garden  ==  gard) 
      
      if ( length (keep)  >   0 ){ 
        
       cortest  <-   cor.test (noNA $ pheno[keep], pred[keep]) 
       pval  <-   round (cortest $ p.value,  4 ) 
       Rval  <-   round (cortest $ estimate,  3 ) 
        
        #par(mfrow = c(1,2))  
        plot (noNA $ pheno[keep], pred[keep],  pch =   16 ,  col =   rgb ( 0 , 0 , 0 , 0.4 ),  
             main =   paste (  'garden ' , gard,  '  \n  ' ,  'R = ' , Rval,  '  |  pval = ' , pval,  sep =   '' ),  
             xlab =   "Height (actual)" ,  
             ylab =   "Height (predicted)" ) 
        abline ( 0 ,  1 ,  col =   'red' ,  lty =   2 )  # 1to1 line  
        abline ( lm (pred[keep]  ~  noNA $ pheno[keep]),  col =   'blue' )  # fit line  
        
       gards_r[gard]  <-  Rval 
        
     }   
    
   } 
    
    hist (gards_r) 
    hist (gards_r ^  2 ) 
    par ( mfrow =   c ( 1 , 1 ))    
   
       barplot (gards_r,  las =   2 )    
   
       # Each year separately  
    
   years  <-   unique (df.scaled $ year) 
    
    par ( mfrow =   c ( 1 , 2 )) 
   years_r  <-   rep ( NA ,  length (years)) 
    names (years_r)  <-  years 
    
    for (n  in   1  :  length (years)){ 
      
     year  <-  years[n] 
      
      # use only data with no NAs (lm removes this automatically)  
     noNA  <-  df.scaled[, c ( "pheno" ,  "garden_clim" ,  "garden_clim_2" ,  "home_clim" ,  "home_clim_2" ,  "genotype" ,  "garden" ,  "year" ,  "pc1" ,  "pc2" ,  "pc3" )] 
     noNA  <-  noNA[ complete.cases (noNA),] 
      
     keep  <-   which (noNA $ year  ==  year) 
      
      if ( length (keep)  >   0 ){ 
        
       cortest  <-   cor.test (noNA $ pheno[keep], pred[keep]) 
       pval  <-   round (cortest $ p.value,  4 ) 
       Rval  <-   round (cortest $ estimate,  3 ) 
        
        #par(mfrow = c(1,2))  
        plot (noNA $ pheno[keep], pred[keep],  pch =   16 ,  col =   rgb ( 0 , 0 , 0 , 0.4 ),  
             main =   paste (  'Year ' , year,  '  \n  ' ,  'R = ' , Rval,  '  |  pval = ' , pval,  sep =   '' ),  
             xlab =   "Height (actual)" ,  
             ylab =   "Height (predicted)" ) 
        abline ( 0 ,  1 ,  col =   'red' ,  lty =   2 )  # 1to1 line  
        abline ( lm (pred[keep]  ~  noNA $ pheno[keep]),  col =   'blue' )  # fit line  
        
       years_r[year]  <-  Rval 
        
     }   
    
   }    
   
       hist (years_r) 
    hist (years_r ^  2 )    
   
       par ( mfrow =   c ( 1 , 1 )) 
    barplot (years_r,  las =   2 )    
   
       # each garden + year separately  
    
   gards  <-   unique (df.scaled $ garden) 
    
    par ( mfrow =   c ( 4 , 5 )) 
    # gards_r <- rep(NA, length(gards))  
    # names(gards_r) <- gards  
    
    # nested loop - loop through gardens then years (some gardens not measured in some years)  
    for (n  in   1  :  length (gards)){ 
      
     gard  <-  gards[n] 
      
     years  <-   sort ( unique (df.scaled[df.scaled $ garden  ==  gard,  'year' ])) 
      
      for (y  in   1  :  length (years)){ 
        
       year  <-  years[y] 
        
        # use only data with no NAs (lm removes this automatically)  
       noNA  <-  df.scaled[, c ( "pheno" ,  "garden_clim" ,  "garden_clim_2" ,  "home_clim" ,  "home_clim_2" ,  "genotype" ,  "garden" ,  "year" ,  "pc1" ,  "pc2" ,  "pc3" )] 
       noNA  <-  noNA[ complete.cases (noNA),] 
        
       keep  <-   which (noNA $ garden  ==  gard  &  noNA $ year  ==  year) 
        
        if ( length (keep)  >   0 ){ 
          
         cortest  <-   cor.test (noNA $ pheno[keep], pred[keep]) 
         pval  <-   round (cortest $ p.value,  4 ) 
         Rval  <-   round (cortest $ estimate,  3 ) 
          
          #par(mfrow = c(1,2))  
          plot (noNA $ pheno[keep], pred[keep],  pch =   16 ,  col =   rgb ( 0 , 0 , 0 , 0.4 ),  
               main =   paste (gard,  ' ' , year,  '  \n  ' ,  'R = ' , Rval,  '  |  pval = ' , pval,  sep =   '' ),  
               xlab =   "Height (actual)" ,  
               ylab =   "Height (predicted)" ) 
          abline ( 0 ,  1 ,  col =   'red' ,  lty =   2 )  # 1to1 line  
          
          # skip plotting fit line if it can't run the correlation (eg SWMN 2022 are all dead)  
          # I haven't gotten this to work  
          #try(x <- abline(lm(pred[keep] ~ noNA$pheno[keep]), col = 'blue'), silent = T)  
          
          # gards_r[gard] <- Rval  
          
       }   
     } 
   }    
   
       # does ancestry affect how well the model predicts height?  
    # might expect hybrids to be less predictable  
   r_vs_pt  <-   data.frame ( cor_R =  genos_r,  cor_pval =  genos_p,   Pt =   NA ,  hybrid_index =   NA ) 
    
   r_vs_pt     
  ##     cor_R cor_pval Pt hybrid_index
## 206 0.827   0.0000 NA           NA
## 210 0.760   0.0000 NA           NA
## 218 0.792   0.0000 NA           NA
## 233 0.805   0.0000 NA           NA
## 255 0.740   0.0000 NA           NA
## 258 0.815   0.0000 NA           NA
## 307 0.879   0.0000 NA           NA
## 311 0.679   0.0000 NA           NA
## 317 0.677   0.0000 NA           NA
## 333 0.833   0.0000 NA           NA
## 334 0.866   0.0000 NA           NA
## 342 0.703   0.0000 NA           NA
## 353 0.690   0.0000 NA           NA
## 364 0.710   0.0000 NA           NA
## 374 0.852   0.0000 NA           NA
## 380 0.608   0.0000 NA           NA
## 381 0.779   0.0000 NA           NA
## 393    NA       NA NA           NA
## 405 0.642   0.0000 NA           NA
## 411 0.737   0.0000 NA           NA
## 416 0.474   0.0002 NA           NA
## 419 0.792   0.0000 NA           NA
## 423 0.751   0.0000 NA           NA
## 427 0.356   0.0150 NA           NA
## 432 0.556   0.0000 NA           NA
## 437 0.858   0.0000 NA           NA
## 443 0.842   0.0000 NA           NA
## 453 0.843   0.0000 NA           NA
## 463 0.805   0.0000 NA           NA
## 469 0.813   0.0000 NA           NA
## 522 0.842   0.0000 NA           NA
## 533 0.687   0.0000 NA           NA
## 543 0.764   0.0000 NA           NA
## 545 0.565   0.0000 NA           NA
## 564 0.828   0.0000 NA           NA
## 567 0.746   0.0000 NA           NA
## 572 0.560   0.0001 NA           NA
## 588 0.735   0.0000 NA           NA
## 590    NA       NA NA           NA
## 601 0.707   0.0000 NA           NA
## 808 0.748   0.0000 NA           NA
## 821 0.628   0.0000 NA           NA
## 827 0.835   0.0000 NA           NA
## 865 0.468   0.0035 NA           NA
## 947    NA       NA NA           NA
## 972 0.820   0.0000 NA           NA
## 973 0.800   0.0000 NA           NA  
       # get Pt (tricho ancestry)  
    for (n  in   1  :  nrow (r_vs_pt)){ 
      
     r_vs_pt[n,  'Pt' ]  <-  dat[ match ( rownames (r_vs_pt)[n], df $ genotype),  'Pt' ] 
     r_vs_pt[n,  'hybrid_index' ]  <-  dat[ match ( rownames (r_vs_pt)[n], df $ genotype),  'hybrid_index_Pb' ] 
      
   } 
    par ( mfrow =   c ( 1 , 2 ))    
   
       plot (r_vs_pt $ Pt, r_vs_pt $ cor_R) 
    plot (r_vs_pt $ hybrid_index, r_vs_pt $ cor_R)    
   
 
 
  6  Model validation using
subsets 
 Test model performance using leave-one-out cross-validation: Split
data into training/test sets by leaving one garden out, training the
model without that garden, then predict for that garden. 
 
  6.1  By garden 
 This section separates models by garden, but not by year - so both
years are included in the plots of actual vs predicted values. The MS
does not report values from this section, instead reporting years
separately as in the next code chunk. 
       # validate model by removing one garden to build model, then testing predictions for that garden  
    
    # plot full model for comparison  
    plot_model (mod,  type =   'std' ,  vline.color =   "black" ,  show.values =  T)  +  
       theme ( text=  element_text ( size=  16 ))  +   
      ggtitle ( 'Full model' )    
   
       # loop though gardens and leave each out  
    # not all of these models converge  
    
   gards  <-   sort ( unique (df.scaled $ garden)) 
    
   mods_test  <-   list () 
    
    for (n  in   1  :  length (gards)){ 
      
     gard  <-  gards[n] 
      
      # subset  
     df.scaled.sub  <-  df.scaled[df.scaled $ garden  !=  gard,] 
      
     mods_test[[n]]  <-   update (mod,  data =  df.scaled.sub) 
      
      summary (mods_test[[n]]) 
      
     p  <-   plot_model (mods_test[[n]],  type =   'std' ,  vline.color =   "black" ,  show.values =  T)  +  
        theme ( text=  element_text ( size=  16 ))  +   
        ggtitle ( paste ( 'without' , gard)) 
      plot (p) 
      
      
   }    
              
  ## Warning in finalizeTMB(TMBStruc, obj, fit, h, data.tmb.old): failed to invert
## Hessian from numDeriv::jacobian(), falling back to internal vcov estimate  
    
  ## Warning in finalizeTMB(TMBStruc, obj, fit, h, data.tmb.old): failed to invert
## Hessian from numDeriv::jacobian(), falling back to internal vcov estimate  
    
  ## Warning in (function (start, objective, gradient = NULL, hessian = NULL, :
## NA/NaN function evaluation  
  ## Warning in (function (start, objective, gradient = NULL, hessian = NULL, :
## NA/NaN function evaluation
## Warning in (function (start, objective, gradient = NULL, hessian = NULL, :
## NA/NaN function evaluation
## Warning in (function (start, objective, gradient = NULL, hessian = NULL, :
## NA/NaN function evaluation
## Warning in (function (start, objective, gradient = NULL, hessian = NULL, :
## NA/NaN function evaluation
## Warning in (function (start, objective, gradient = NULL, hessian = NULL, :
## NA/NaN function evaluation  
  ## Warning in finalizeTMB(TMBStruc, obj, fit, h, data.tmb.old): Model convergence
## problem; non-positive-definite Hessian matrix. See vignette('troubleshooting')  
  ## Warning in finalizeTMB(TMBStruc, obj, fit, h, data.tmb.old): Model convergence
## problem; false convergence (8). See vignette('troubleshooting'),
## help('diagnose')  
  ## Warning in sqrt(diag(vcovs)): NaNs produced
## Warning in sqrt(diag(vcovs)): NaNs produced  
   
       names (mods_test)  <-   paste ( 'without_' , gards,  sep =   '' ) 
    
    plot_models (mods_test,  std.est =   'std' ,  spacing =   0.8 ,  vline.color =   "black" ,  show.values =  F,  p.shape =  T,  m.labels =   names (mods_test),  colors =   c ( "#008856" ,   "#8DB600" ,  "#BE0032" ,  "#875692" ,  "#A1CAF1" ,  "#2B3D26" ,  "#C2B280" ,  "#F3C300" ,  "#848482" ,  "#0067A5" ,  "#E68FAC" ,  "#F99379" ,  "#604E97" ,  "#B3446C" ,  "#DCD300" ,  "#882D17" ,  "#E25822" ,  "#654522" ))    
  ## Warning in finalizeTMB(TMBStruc, obj, fit, h, data.tmb.old): failed to invert
## Hessian from numDeriv::jacobian(), falling back to internal vcov estimate  
  ## Warning in finalizeTMB(TMBStruc, obj, fit, h, data.tmb.old): failed to invert
## Hessian from numDeriv::jacobian(), falling back to internal vcov estimate  
   
       #ggsave(filename = paste('results/model_prediction/compare_models_leave_one_garden_out_', garden_clim_colname, '_vs_', pheno_colname, '.pdf', sep = ''), device = 'pdf', height = 12, width = 8)  
    
    ########  
    
    # residuals for each sub-model  
    #pdf(file = 'results/model_prediction/residuals_density_leave_garden_out.pdf', height = 6, width = 16)  
    #plot_resid(mods_test)  
    #dev.off()  
    
    #########  
    # plot predicted vs actual for the full dataset (all gardens) for each model  
    
    par ( mfrow =   c ( 2 , 2 )) 
    
    for (n  in   1  :  length (mods_test)){ 
      
     modN  <-  mods_test[[n]] 
      
          # overall model  
        plot_predicted_vs_actual ( model_R (modN,  re.form =   NULL ),  title =   names (mods_test)[n]) 
        plot_predicted_vs_actual ( model_R (modN,  re.form =   NA ),  title =   'no random effects' ) 
        # conditional model (just growth)  
        # with dead trees removed  
        plot_predicted_vs_actual ( model_R (modN,  re.form =   NULL ,  type =   'conditional' ),  title =   'conditional' ) 
        plot_predicted_vs_actual ( model_R (modN,  re.form =   NA ,  type =   'conditional' ),  title =   'no random effects' ) 
      
   }    
                   
       ##########  
    # predict for each garden left out of model  
    
    
    # function for predicting with new data - based on model_R() function defined above  
   model_R_new  <-   function (mod, newdata,  type =   'response' ,  re.form =   NULL ,  se.fit =   FALSE ){ 
      
     df  <-  newdata 
      
      # make new dataframe with no NA values  
     noNA  <-  df[ complete.cases (df),] 
      
      # predict phenotype (generally height)  
     noNA $ pred  <-   predict (mod,  newdata =  noNA,  type =  type,  re.form =  re.form,  se.fit =  se.fit,  allow.new.levels=  TRUE ) 
      
      # un-log-transform  
     noNA $ pred  <-   exp (noNA $ pred)  -   1  
      
      # if type = conditional, remove dead trees (which are modeled separately in zero-inflated model)  
      if (type  ==   'conditional' ){ 
       noNA  <-  noNA[ rownames (noNA)  %in%  alive,] 
     } 
      
      # calculate correlation  
     cor  <-   cor.test (noNA $ pheno, noNA $ pred) 
      
      return ( list ( actual =  noNA $ pheno,  predicted =  noNA $ pred,  cor =  cor,  data_noNAs =  noNA)) 
      
   } 
    
    
    # loop through each model and predict for garden that was left out  
    
    # objects to save results to  
   pred.overall_REs  <-   list () 
   pred.overall_noREs  <-   list () 
   pred.cond_REs  <-   list () 
   pred.cond_noREs  <-   list () 
    
    par ( mfrow =   c ( 2 , 2 )) 
    for (n  in   1  :  length (gards)){ 
      
     gard  <-  gards[n] 
     modN  <-  mods_test[[n]] 
      
     newdata  <-  df.scaled[df.scaled $ garden  ==  gard,] 
      
      # plot  
      # overall model  
      
     pred.overall_REs[[n]]  <-   model_R_new (modN,  newdata =   newdata,  re.form =   NULL ) 
      plot_predicted_vs_actual (pred.overall_REs[[n]],  title =   paste ( names (mods_test)[n],  'overall, with random effects' ,  sep =   '  \n  ' )) 
      
     pred.overall_noREs[[n]]  <-   model_R_new (modN,  newdata =  newdata,  re.form =   NA ) 
      plot_predicted_vs_actual (pred.overall_noREs[[n]],  title =   'overall, no random effects' ) 
      
      
      # conditional model  
     pred.cond_REs[[n]]  <-   model_R_new (modN,  type =   'conditional' ,  newdata =   newdata,  re.form =   NULL ) 
      plot_predicted_vs_actual (pred.cond_REs[[n]],  title =   'conditional, with random effects' ) 
      
     pred.cond_noREs[[n]]  <-   model_R_new (modN,  type =   'conditional' ,  newdata =  newdata,  re.form =   NA ) 
      plot_predicted_vs_actual (pred.cond_noREs[[n]],  title =   'conditional, no random effects' ) 
      
      
   }    
                   
       # remove "all" model from names  
   model_names  <-   names (mods_test)[ names (mods_test)  !=   'all' ] 
    names (pred.overall_REs)  <-  model_names 
    names (pred.overall_noREs)  <-  model_names 
    names (pred.cond_REs)  <-  model_names 
    names (pred.cond_noREs)  <-  model_names 
    
   preds.full  <-   list (pred.overall_REs, pred.overall_noREs, pred.cond_REs, pred.cond_noREs) 
    names (preds.full)  <-   c ( 'overall_REs' ,  'overall_noREs' ,  'cond_REs' ,  'cond_noREs' ) 
    
    # plot panel with just the conditional RE plots  
   gard_cols  <-   c ( "#008856" ,   "#8DB600" ,  "#BE0032" ,  "#875692" ,  "#A1CAF1" ,  "#2B3D26" ,  "#C2B280" ,  "#F3C300" ,  "#848482" ,  "#0067A5" ,  "#E68FAC" ,  "#F99379" ,  "#604E97" ,  "#B3446C" ,  "#DCD300" ,  "#882D17" ,  "#E25822" ,  "#654522" ) 
    
    
    #png(filename = paste('results/model_prediction/validation_leaveOneGardenOutAndPredict_', garden_clim_colname, '_vs_', pheno_colname, '.png', sep = ''), height = 7.2, width = 12.8, units = 'in', res = 300)  
    
    #pdf(file = paste('results/model_prediction/validation_leaveOneGardenOutAndPredict_', garden_clim_colname, '_vs_', pheno_colname, '.pdf', sep = ''), height = 8, width = 12)  
    par ( mfrow =   c ( 3 , 4 )) 
    
    for (n  in   1  :  length (pred.cond_noREs)){ 
      
     gard  <-   gsub ( 'without_' ,  '' ,  names (pred.cond_noREs)[n]) 
      plot_predicted_vs_actual (pred.cond_noREs[[n]],  
                               title =  gard,  
                               col =  gard_cols[n], 
                               col_1to1 =   'grey' , 
                               col_fit =  gard_cols[n]) 
      
   }    
   
       #dev.off()  
    
    # version ordered by mortality - use mean to get a proportion  
    # use as character so it's alphabetical to match 'gard' object  
   dead  <-   aggregate (dat $ Survival_09_2021,  by =   list ( as.character (dat $ MiniCG_Site)),  FUN =  mean,  na.rm =  T) 
    
   ord  <-   order (dead $ x,  decreasing =  T) 
    
    # proportion surviving in each garden (NOT proportion dead)  
   dead[ord,]    
  ##      Group.1         x
## 11        SU 1.0000000
## 12      SWMN 0.9787234
## 1  EVERGREEN 0.9574468
## 14        VA 0.9347826
## 10      PENN 0.9255319
## 16       WSU 0.9090909
## 2         ID 0.9042553
## 5        MSU 0.9042553
## 17       WYO 0.8191489
## 15        WI 0.8085106
## 7       NWMO 0.7127660
## 4     MORTON 0.6276596
## 13       UCM 0.5851064
## 9        OSU 0.5543478
## 3       LOCK 0.5425532
## 8       OLLU 0.5319149
## 6       NDSU 0.4255319  
      gard.sort  <-  dead $ Group .1 [ord] 
    
    #pdf(file = paste('results/model_prediction/validation_leaveOneGardenOutAndPredict_sortGardensByMortality', garden_clim_colname, '_vs_', pheno_colname, '.pdf', sep = ''), height = 8, width = 12)  
    
    par ( mfrow =   c ( 3 , 4 ))    
   
       for (n  in  ord){ 
      
      
     gard  <-  gards[n] 
      
      plot_predicted_vs_actual (pred.cond_noREs[[n]],  
                               title =  gard,  
                               col =  gard_cols[n], 
                               col_1to1 =   'grey' , 
                               col_fit =  gard_cols[n]) 
      
   }    
   
       #dev.off()  
    
    # png version for supplement  
    
    #png(filename = paste('results/model_prediction/validation_leaveOneGardenOutAndPredict_sortGardensByMortality', garden_clim_colname, '_vs_', pheno_colname, '.png', sep = ''), height = 7.2, width = 12.8, units = 'in', res = 300)  
    
    par ( mfrow =   c ( 3 , 6 ))    
   
       for (n  in  ord){ 
      
      
     gard  <-  gards[n] 
      
      plot_predicted_vs_actual (pred.cond_REs[[n]],  
                               title =  gard,  
                               col =  gard_cols[n], 
                               col_1to1 =   'grey' , 
                               col_fit =  gard_cols[n], 
                               legend =  F) 
      
   } 
    
    
    #dev.off()  
    
    # get all the R values  
   rvals  <-   as.data.frame ( matrix ( nrow =   length (preds.full $ overall_REs),  ncol =   4 )) 
    colnames (rvals)  <-   names (preds.full) 
    rownames (rvals)  <-   names (preds.full $ overall_REs) 
    
   rvals $ overall_REs  <-   as.numeric ( lapply ( 1  :  length (preds.full $ overall_REs),  function (x) preds.full $ overall_REs[[x]] $ cor $ estimate)) 
   rvals $ overall_noREs  <-   as.numeric ( lapply ( 1  :  length (preds.full $ overall_noREs),  function (x) preds.full $ overall_noREs[[x]] $ cor $ estimate)) 
   rvals $ cond_REs  <-   as.numeric ( lapply ( 1  :  length (preds.full $ cond_REs),  function (x) preds.full $ cond_REs[[x]] $ cor $ estimate)) 
   rvals $ cond_noREs  <-   as.numeric ( lapply ( 1  :  length (preds.full $ cond_noREs),  function (x) preds.full $ cond_noREs[[x]] $ cor $ estimate)) 
    
   rvals    
  ##                   overall_REs overall_noREs  cond_REs cond_noREs
## without_EVERGREEN  0.53344130   0.498068166 0.5444618  0.5057557
## without_ID         0.41004013   0.394889822 0.4402556  0.4232682
## without_LOCK       0.27532887   0.250420061 0.4592166  0.3265674
## without_MORTON     0.25569009   0.266308662 0.3069637  0.3403586
## without_MSU        0.47701103   0.436132365 0.5654567  0.5102041
## without_NDSU       0.29975506   0.280686281 0.4285887  0.4210366
## without_NWMO       0.34362366   0.314375707 0.5462739  0.5418791
## without_OLLU       0.14300101   0.132683574 0.2175649  0.1836277
## without_OSU        0.52950408   0.529416821 0.6076054  0.6032568
## without_PENN       0.60020092   0.538490197 0.6493221  0.5905257
## without_SU         0.46308374   0.440892298 0.5626802  0.5446174
## without_SWMN       0.20087909   0.219286134 0.3144451  0.2804939
## without_UCM        0.18270583   0.139429619 0.3310679  0.2213358
## without_VA         0.59116080   0.573018105 0.6329196  0.6028021
## without_WI         0.02189791   0.002079156 0.1385380  0.1126083
## without_WSU        0.57849694   0.527685983 0.5774656  0.5255068
## without_WYO        0.23401699  -0.159029899 0.2993616 -0.2664114  
       # plots  
    par ( mfrow =   c ( 2 , 2 ))    
   
       # xlim assumes no negative correlations  
    hist (rvals $ overall_REs,  xlim =   c ( 0 , 1 )) 
    hist (rvals $ overall_noREs,  xlim =   c ( 0 , 1 )) 
    hist (rvals $ cond_REs,  xlim =   c ( 0 , 1 )) 
    hist (rvals $ cond_noREs,  xlim =   c ( 0 , 1 ))    
   
       colMeans (rvals)    
  ##   overall_REs overall_noREs      cond_REs    cond_noREs 
##     0.3611669     0.3167549     0.4483640     0.3804372  
      rvals[ which.max (rvals $ cond_REs),]    
  ##              overall_REs overall_noREs  cond_REs cond_noREs
## without_PENN   0.6002009     0.5384902 0.6493221  0.5905257  
      rvals[ which.min (rvals $ cond_REs),]    
  ##            overall_REs overall_noREs cond_REs cond_noREs
## without_WI  0.02189791   0.002079156 0.138538  0.1126083  
 
 
  6.2  By garden + year 
 Predictions for each garden are made separately for each year, if
multiple years of data are available (some gardens were only measured
the first year). These are the results reported in the manuscript. 
       # loop through each model and predict for garden that was left out  
    # here, predictions are separated by year   
    # so model is trained without a garden, then predictions for that garden are made separately for each year  
    # use mods_test object from above chunk, with each garden removed   
    
   gard_cols  <-   c ( "#008856" ,   "#8DB600" ,  "#BE0032" ,  "#875692" ,  "#A1CAF1" ,  "#2B3D26" ,  "#C2B280" ,  "#F3C300" ,  "#848482" ,  "#0067A5" ,  "#E68FAC" ,  "#F99379" ,  "#604E97" ,  "#B3446C" ,  "#DCD300" ,  "#882D17" ,  "#E25822" ,  "#654522" ) 
    
    # objects to save results to  
   pred.overall_REs  <-   list () 
   pred.overall_noREs  <-   list () 
   pred.cond_REs  <-   list () 
   pred.cond_noREs  <-   list () 
    
   gards  <-   sort ( unique (df.scaled $ garden)) 
   cols.yr  <-   vector () 
    
    # check that garden names and model names are in the same order  
    cbind (gards,  names (mods_test))    
  ##       gards                          
##  [1,] "EVERGREEN" "without_EVERGREEN"
##  [2,] "ID"        "without_ID"       
##  [3,] "LOCK"      "without_LOCK"     
##  [4,] "MORTON"    "without_MORTON"   
##  [5,] "MSU"       "without_MSU"      
##  [6,] "NDSU"      "without_NDSU"     
##  [7,] "NWMO"      "without_NWMO"     
##  [8,] "OLLU"      "without_OLLU"     
##  [9,] "OSU"       "without_OSU"      
## [10,] "PENN"      "without_PENN"     
## [11,] "SU"        "without_SU"       
## [12,] "SWMN"      "without_SWMN"     
## [13,] "UCM"       "without_UCM"      
## [14,] "VA"        "without_VA"       
## [15,] "WI"        "without_WI"       
## [16,] "WSU"       "without_WSU"      
## [17,] "WYO"       "without_WYO"  
       par ( mfrow =   c ( 2 , 2 )) 
    # counter to keep track of which garden/year we're on  
   count  <-   1  
    for (n  in   1  :  length (gards)){ 
      
      # loop through each garden, use model trained without that garden  
     gard  <-  gards[n] 
     modN  <-  mods_test[[n]] 
      
      # subset data to this garden  
     df.scaled.sub  <-  df.scaled[df.scaled $ garden  ==  gard,] 
      
      # now loop through each year for this garden  
     years  <-   sort ( unique (df.scaled.sub $ year)) 
      
      for (y  in   1  :  length (years)){ 
        
       year  <-  years[y] 
        
        # subset data to this year  
       newdata  <-  df.scaled.sub[df.scaled.sub $ year  ==  year,] 
        
        # plot actual vs predicted for this garden and year, using model trained without this garden  
        
        # plots  
        # overall model  
        
       pred.overall_REs[[count]]  <-   model_R_new (modN,  newdata =   newdata,  re.form =   NULL ) 
        plot_predicted_vs_actual (pred.overall_REs[[count]],  title =   paste ( names (mods_test)[n],  ', predicted for ' , year,  '  \n  ' ,  'overall, with random effects' ,  sep =   '' )) 
        
       pred.overall_noREs[[count]]  <-   model_R_new (modN,  newdata =  newdata,  re.form =   NA ) 
        plot_predicted_vs_actual (pred.overall_noREs[[count]],  title =   'overall, no random effects' ) 
        
        
        # conditional model  
       pred.cond_REs[[count]]  <-   model_R_new (modN,  type =   'conditional' ,  newdata =   newdata,  re.form =   NULL ) 
        plot_predicted_vs_actual (pred.cond_REs[[count]],  title =   'conditional, with random effects' ) 
        
       pred.cond_noREs[[count]]  <-   model_R_new (modN,  type =   'conditional' ,  newdata =  newdata,  re.form =   NA ) 
        plot_predicted_vs_actual (pred.cond_noREs[[count]],  title =   'conditional, no random effects' ) 
        
        # name output  
       name  <-   paste (gard, year,  sep =   '_' ) 
        names (pred.overall_REs)[[count]]  <-  name 
        names (pred.overall_noREs)[[count]]  <-  name 
        names (pred.cond_REs)[[count]]  <-  name 
        names (pred.cond_noREs)[[count]]  <-  name 
        
        # set garden color and save to a vector  
       cols.yr[count]  <-  gard_cols[n] 
        
        # set counter  
       count  <-  count  +   1  
     } 
   }    
                                 
      preds.full  <-   list (pred.overall_REs, pred.overall_noREs, pred.cond_REs, pred.cond_noREs) 
    names (preds.full)  <-   c ( 'overall_REs' ,  'overall_noREs' ,  'cond_REs' ,  'cond_noREs' ) 
    
    # get yearly averages  
   pred .21   <-  pred.cond_REs[ grep ( '2021' ,  names (pred.cond_REs))] 
    mean ( sapply ( 1  :  length (pred .21 ),  function (x)  mean (pred .21 [[x]] $ cor $ estimate)))    
  ## [1] 0.4884965  
      pred .22   <-  pred.cond_REs[ grep ( '2022' ,  names (pred.cond_REs))] 
    mean ( sapply ( 1  :  length (pred .22 ),  function (x)  mean (pred .22 [[x]] $ cor $ estimate)))    
  ## [1] 0.3812715  
       # plot panel with just the conditional RE plots  
    
    
    #pdf(file = paste('results/model_prediction/validation_leaveOneGardenOutAndPredictYear_', garden_clim_colname, '_vs_', pheno_colname, '.pdf', sep = ''), height = 8, width = 12)  
    par ( mfrow =   c ( 3 , 4 )) 
    
    for (n  in   1  :  length (pred.cond_REs)){ 
      
      
     title  <-   names (pred.cond_REs)[n] 
      plot_predicted_vs_actual (pred.cond_REs[[n]],  
                               title =  title,  
                               col =  cols.yr[n], 
                               col_1to1 =   'grey' , 
                               col_fit =  cols.yr[n], 
                               legend =  F) 
      
   }    
    
       #dev.off()  
    
    #png(filename = paste('results/model_prediction/validation_leaveOneGardenOutAndPredictYear_', garden_clim_colname, '_vs_', pheno_colname, '.png', sep = ''), height = 16, width = 9.6, units = 'in', res = 300)  
    
    #par(mfrow = c(8,4))  
    par ( mfrow =   c ( 4 , 5 ))    
   
       for (n  in   1  :  length (pred.cond_REs)){ 
      
      
     title  <-   names (pred.cond_REs)[n] 
      plot_predicted_vs_actual (pred.cond_REs[[n]],  
                               title =  title,  
                               col =  cols.yr[n], 
                               col_1to1 =   'grey' , 
                               col_fit =  cols.yr[n], 
                               legend =  F) 
      
   }    
   
       #dev.off()  
    
    # get all the R values  
    #hist(as.numeric(lapply(1:length(preds.full$cond_REs), function(x) preds.full$cond_REs[[x]]$cor$estimate)))     
   
 
 
 
  7  Compare model
predictive ability when genetic and home climate information is
removed 
 Test what information is needed to predict each genotype’s response
to temperature. if home climate can serve as a proxy for locally
adaptive genetic variation, including genetic data may not be necessary
for predicting growth under future climates. 
       # Does genetics or home climate give better predictions of fitness?  
    # using same method as above, calculate R^2 values when leaving each garden out  
    # but compare full model to models with home climate or genetic structure removed  
    
    # only climate, no genetics  
    
   gards  <-   unique (df.scaled $ garden) 
    
   mods_test_no_gen  <-   list () 
    
    for (n  in   1  :  length (gards)){ 
      
     gard  <-  gards[n] 
      
      # subset  
     df.scaled.sub  <-  df.scaled[df.scaled $ garden  !=  gard,] 
      
      # run model  
     mods_test_no_gen[[n]]  <-    glmmTMB ( log (pheno  +   1 )  ~  garden_clim * home_clim  +  garden_clim_2 * home_clim_2   +  garden_clim_2 * home_clim  +  home_clim_2 * garden_clim  +  
                                         ( 1   |  genotype)  +  ( 1   |  garden / block)  +  ( 1   |  year)  +  ( 1   |  indiv), 
                                        data =  df.scaled.sub,  
                                        family =   gaussian (),  
                                        ziformula =   ~ .) 
      
    summary (mods_test_no_gen[[n]]) 
    
   p  <-   plot_model (mods_test_no_gen[[n]],  type =   'std' ,  vline.color =   "black" ,  show.values =  T)  +  
      theme ( text=  element_text ( size=  16 ))  +   
      ggtitle ( paste ( 'without' , gard)) 
    plot (p) 
      
      
   }    
      
  ## Warning in finalizeTMB(TMBStruc, obj, fit, h, data.tmb.old): Model convergence
## problem; non-positive-definite Hessian matrix. See vignette('troubleshooting')  
   
  ## Warning in finalizeTMB(TMBStruc, obj, fit, h, data.tmb.old): failed to invert
## Hessian from numDeriv::jacobian(), falling back to internal vcov estimate  
       
  ## Warning in finalizeTMB(TMBStruc, obj, fit, h, data.tmb.old): Model convergence
## problem; non-positive-definite Hessian matrix. See vignette('troubleshooting')  
   
  ## Warning in finalizeTMB(TMBStruc, obj, fit, h, data.tmb.old): Model convergence
## problem; non-positive-definite Hessian matrix. See vignette('troubleshooting')  
  ## Warning in finalizeTMB(TMBStruc, obj, fit, h, data.tmb.old): Model convergence
## problem; singular convergence (7). See vignette('troubleshooting'),
## help('diagnose')  
      
  ## Warning in finalizeTMB(TMBStruc, obj, fit, h, data.tmb.old): Model convergence
## problem; non-positive-definite Hessian matrix. See vignette('troubleshooting')  
    
       names (mods_test_no_gen)  <-   paste ( 'without_' , gards,  sep =   '' ) 
    
    # plot together  
    
    plot_models (mods_test_no_gen,  std.est =   'std' ,  spacing =   0.8 ,  vline.color =   "black" ,  show.values =  F,  p.shape =  T,  m.labels =   names (mods_test),  colors =   c ( "#008856" ,   "#8DB600" ,  "#BE0032" ,  "#875692" ,  "#A1CAF1" ,  "#2B3D26" ,  "#C2B280" ,  "#F3C300" ,  "#848482" ,  "#0067A5" ,  "#E68FAC" ,  "#F99379" ,  "#604E97" ,  "#B3446C" ,  "#DCD300" ,  "#882D17" ,  "#E25822" ,  "#654522" ))    
   
       #ggsave(filename = paste('results/model_prediction/compare_models_leave_one_garden_out_NO_GENETICS', garden_clim_colname, '_vs_', pheno_colname, '.pdf', sep = ''), device = 'pdf', height = 12, width = 8)  
    
    
    # loop through each model and predict for garden that was left out  
    
    # objects to save results to  
   pred.overall_REs  <-   list () 
   pred.overall_noREs  <-   list () 
   pred.cond_REs  <-   list () 
   pred.cond_noREs  <-   list () 
    
    par ( mfrow =   c ( 2 , 2 )) 
    
    # counter to keep track of which garden/year we're on  
   count  <-   1  
    for (n  in   1  :  length (gards)){ 
      
      # loop through each garden, use model trained without that garden  
     gard  <-  gards[n] 
     modN  <-  mods_test_no_gen[[n]] 
      
      # subset data to this garden  
     df.scaled.sub  <-  df.scaled[df.scaled $ garden  ==  gard,] 
      
      # now loop through each year for this garden  
     years  <-   sort ( unique (df.scaled.sub $ year)) 
      
      for (y  in   1  :  length (years)){ 
        
       year  <-  years[y] 
        
        # subset data to this year  
       newdata  <-  df.scaled.sub[df.scaled.sub $ year  ==  year,] 
        
        # plot actual vs predicted for this garden and year, using model trained without this garden  
        
        # plots  
        # overall model  
        
       pred.overall_REs[[count]]  <-   model_R_new (modN,  newdata =   newdata,  re.form =   NULL ) 
        plot_predicted_vs_actual (pred.overall_REs[[count]],  title =   paste ( names (mods_test)[n],  ', predicted for ' , year,  '  \n  ' ,  'overall, with random effects' ,  sep =   '' )) 
        
       pred.overall_noREs[[count]]  <-   model_R_new (modN,  newdata =  newdata,  re.form =   NA ) 
        plot_predicted_vs_actual (pred.overall_noREs[[count]],  title =   'overall, no random effects' ) 
        
        
        # conditional model  
       pred.cond_REs[[count]]  <-   model_R_new (modN,  type =   'conditional' ,  newdata =   newdata,  re.form =   NULL ) 
        plot_predicted_vs_actual (pred.cond_REs[[count]],  title =   'conditional, with random effects' ) 
        
       pred.cond_noREs[[count]]  <-   model_R_new (modN,  type =   'conditional' ,  newdata =  newdata,  re.form =   NA ) 
        plot_predicted_vs_actual (pred.cond_noREs[[count]],  title =   'conditional, no random effects' ) 
        
        # name output  
       name  <-   paste (gard, year,  sep =   '_' ) 
        names (pred.overall_REs)[[count]]  <-  name 
        names (pred.overall_noREs)[[count]]  <-  name 
        names (pred.cond_REs)[[count]]  <-  name 
        names (pred.cond_noREs)[[count]]  <-  name 
        
        # set garden color and save to a vector  
        #cols.yr[count] <- gard_cols[n]  
        
        # set counter  
       count  <-  count  +   1  
     } 
   }    
                                 
      preds.nogen  <-   list (pred.overall_REs, pred.overall_noREs, pred.cond_REs, pred.cond_noREs) 
    names (preds.nogen)  <-   c ( 'overall_REs' ,  'overall_noREs' ,  'cond_REs' ,  'cond_noREs' ) 
    
    
    
    # only genetics and garden climate, not home climate  
    
   gards  <-   unique (df.scaled $ garden) 
    
   mods_test_no_clim  <-   list () 
    
    for (n  in   1  :  length (gards)){ 
      
     gard  <-  gards[n] 
      
      # subset  
     df.scaled.sub  <-  df.scaled[df.scaled $ garden  !=  gard,] 
      
      # run model  
     mods_test_no_clim[[n]]  <-    glmmTMB ( log (pheno  +   1 )  ~  garden_clim  +  garden_clim_2   +  
                                          pc1  +  pc2  +  pc3  +   
                                          pc1 * garden_clim  +  pc2 * garden_clim  +  pc3 * garden_clim  +  pc1 * garden_clim_2  +  pc2 * garden_clim_2  +  pc3 * garden_clim_2  +  
                                          ( 1   |  genotype)  +  ( 1   |  garden / block)  +  ( 1   |  year)  +  ( 1   |  indiv), 
                                         data =  df.scaled.sub,  
                                         family =   gaussian (),  
                                         ziformula =   ~ .) 
      
      summary (mods_test_no_clim[[n]]) 
      
     p  <-   plot_model (mods_test_no_clim[[n]],  type =   'std' ,  vline.color =   "black" ,  show.values =  T)  +  
        theme ( text=  element_text ( size=  16 ))  +   
        ggtitle ( paste ( 'without' , gard)) 
      plot (p) 
      
      
   }    
    
  ## Warning in (function (start, objective, gradient = NULL, hessian = NULL, :
## NA/NaN function evaluation  
  ## Warning in (function (start, objective, gradient = NULL, hessian = NULL, :
## NA/NaN function evaluation
## Warning in (function (start, objective, gradient = NULL, hessian = NULL, :
## NA/NaN function evaluation
## Warning in (function (start, objective, gradient = NULL, hessian = NULL, :
## NA/NaN function evaluation  
  ## Warning in finalizeTMB(TMBStruc, obj, fit, h, data.tmb.old): Model convergence
## problem; non-positive-definite Hessian matrix. See vignette('troubleshooting')  
  ## Warning in finalizeTMB(TMBStruc, obj, fit, h, data.tmb.old): Model convergence
## problem; false convergence (8). See vignette('troubleshooting'),
## help('diagnose')  
    
  ## Warning in finalizeTMB(TMBStruc, obj, fit, h, data.tmb.old): Model convergence
## problem; non-positive-definite Hessian matrix. See vignette('troubleshooting')  
  ## Warning in finalizeTMB(TMBStruc, obj, fit, h, data.tmb.old): Model convergence
## problem; non-positive-definite Hessian matrix. See vignette('troubleshooting')  
               
       names (mods_test_no_clim)  <-   paste ( 'without_' , gards,  sep =   '' ) 
    
    # plot together  
    
    plot_models (mods_test_no_clim,  std.est =   'std' ,  spacing =   0.8 ,  vline.color =   "black" ,  show.values =  F,  p.shape =  T,  m.labels =   names (mods_test),  colors =   c ( "#008856" ,   "#8DB600" ,  "#BE0032" ,  "#875692" ,  "#A1CAF1" ,  "#2B3D26" ,  "#C2B280" ,  "#F3C300" ,  "#848482" ,  "#0067A5" ,  "#E68FAC" ,  "#F99379" ,  "#604E97" ,  "#B3446C" ,  "#DCD300" ,  "#882D17" ,  "#E25822" ,  "#654522" ))    
  ## Warning in (function (start, objective, gradient = NULL, hessian = NULL, :
## NA/NaN function evaluation  
  ## Warning in (function (start, objective, gradient = NULL, hessian = NULL, :
## NA/NaN function evaluation
## Warning in (function (start, objective, gradient = NULL, hessian = NULL, :
## NA/NaN function evaluation
## Warning in (function (start, objective, gradient = NULL, hessian = NULL, :
## NA/NaN function evaluation  
  ## Warning in finalizeTMB(TMBStruc, obj, fit, h, data.tmb.old): Model convergence
## problem; non-positive-definite Hessian matrix. See vignette('troubleshooting')  
  ## Warning in finalizeTMB(TMBStruc, obj, fit, h, data.tmb.old): Model convergence
## problem; false convergence (8). See vignette('troubleshooting'),
## help('diagnose')  
  ## Warning in finalizeTMB(TMBStruc, obj, fit, h, data.tmb.old): Model convergence
## problem; non-positive-definite Hessian matrix. See vignette('troubleshooting')  
   
       #ggsave(filename = paste('results/model_prediction/compare_models_leave_one_garden_out_NO_HOME_CLIMATE', garden_clim_colname, '_vs_', pheno_colname, '.pdf', sep = ''), device = 'pdf', height = 12, width = 8)  
    
    
    # loop through each model and predict for garden that was left out  
    # this uses the full model, no climate, and no genetics so they can be compared for each run without a garden  
    
    # objects to save results to  
   pred.overall_REs  <-   list () 
   pred.overall_noREs  <-   list () 
   pred.cond_REs  <-   list () 
   pred.cond_noREs  <-   list () 
    
    par ( mfrow =   c ( 2 , 2 )) 
    # counter to keep track of which garden/year we're on  
   count  <-   1  
    for (n  in   1  :  length (gards)){ 
      
      # loop through each garden, use model trained without that garden  
     gard  <-  gards[n] 
     modN  <-  mods_test_no_clim[[n]] 
      
      # subset data to this garden  
     df.scaled.sub  <-  df.scaled[df.scaled $ garden  ==  gard,] 
      
      # now loop through each year for this garden  
     years  <-   sort ( unique (df.scaled.sub $ year)) 
      
      for (y  in   1  :  length (years)){ 
        
       year  <-  years[y] 
        
        # subset data to this year  
       newdata  <-  df.scaled.sub[df.scaled.sub $ year  ==  year,] 
        
        # plot actual vs predicted for this garden and year, using model trained without this garden  
        
        # plots  
        # overall model  
        
       pred.overall_REs[[count]]  <-   model_R_new (modN,  newdata =   newdata,  re.form =   NULL ) 
        plot_predicted_vs_actual (pred.overall_REs[[count]],  title =   paste ( names (mods_test)[n],  ', predicted for ' , year,  '  \n  ' ,  'overall, with random effects' ,  sep =   '' )) 
        
       pred.overall_noREs[[count]]  <-   model_R_new (modN,  newdata =  newdata,  re.form =   NA ) 
        plot_predicted_vs_actual (pred.overall_noREs[[count]],  title =   'overall, no random effects' ) 
        
        
        # conditional model  
       pred.cond_REs[[count]]  <-   model_R_new (modN,  type =   'conditional' ,  newdata =   newdata,  re.form =   NULL ) 
        plot_predicted_vs_actual (pred.cond_REs[[count]],  title =   'conditional, with random effects' ) 
        
       pred.cond_noREs[[count]]  <-   model_R_new (modN,  type =   'conditional' ,  newdata =  newdata,  re.form =   NA ) 
        plot_predicted_vs_actual (pred.cond_noREs[[count]],  title =   'conditional, no random effects' ) 
        
        # name output  
       name  <-   paste (gard, year,  sep =   '_' ) 
        names (pred.overall_REs)[[count]]  <-  name 
        names (pred.overall_noREs)[[count]]  <-  name 
        names (pred.cond_REs)[[count]]  <-  name 
        names (pred.cond_noREs)[[count]]  <-  name 
        
        # set garden color and save to a vector  
       cols.yr[count]  <-  gard_cols[n] 
        
        # set counter  
       count  <-  count  +   1  
     } 
   }    
                                 
      preds.noclim  <-   list (pred.overall_REs, pred.overall_noREs, pred.cond_REs, pred.cond_noREs) 
    names (preds.noclim)  <-   c ( 'overall_REs' ,  'overall_noREs' ,  'cond_REs' ,  'cond_noREs' ) 
    
    ###########################  
    # compare R values of predictions among all three models  
    
    par ( mfrow =   c ( 1 , 3 )) 
    hist ( as.numeric ( lapply ( 1  :  length (preds.full $ cond_REs),  function (x) preds.full $ cond_REs[[x]] $ cor $ estimate))) 
    hist ( as.numeric ( lapply ( 1  :  length (preds.nogen $ cond_REs),  function (x) preds.nogen $ cond_REs[[x]] $ cor $ estimate))) 
    hist ( as.numeric ( lapply ( 1  :  length (preds.noclim $ cond_REs),  function (x) preds.noclim $ cond_REs[[x]] $ cor $ estimate)))    
   
       # put all R values into a dataframe, each row being a model with one garden excluded, a column for full/conditional models and with/without random effects  
    # I did this in a pretty ugly way, oh well  
    
   mods_test_R  <-   data.frame ( matrix ( nrow =   length (preds.full $ overall_noREs) *  3 ,  ncol =   1 )) 
    
    colnames (mods_test_R)  <-   'model'  
    
   mods_test_R $ model  <-   c ( 
      rep ( 'full' ,  length (preds.full $ cond_REs)), 
      rep ( 'no_genetics' ,  length (preds.nogen $ cond_REs)), 
      rep ( 'no_climate' ,  length (preds.noclim $ cond_REs)) 
   ) 
    
   mods_test_R $ test_data  <-   c ( 
      names (preds.full $ cond_REs), 
      names (preds.nogen $ cond_REs), 
      names (preds.noclim $ cond_REs) 
   ) 
    
    # add overall with REs  
   mods_test_R $ Rval_overall_REs  <-   c ( 
      as.numeric ( lapply ( 1  :  length (preds.full $ overall_REs),  function (x) preds.full $ overall_REs[[x]] $ cor $ estimate)), 
      as.numeric ( lapply ( 1  :  length (preds.nogen $ overall_REs),  function (x) preds.nogen $ overall_REs[[x]] $ cor $ estimate)), 
      as.numeric ( lapply ( 1  :  length (preds.noclim $ overall_REs),  function (x) preds.noclim $ overall_REs[[x]] $ cor $ estimate)) 
   ) 
    
    # add overall without REs  
   mods_test_R $ Rval_overall_noREs  <-   c ( 
      as.numeric ( lapply ( 1  :  length (preds.full $ overall_noREs),  function (x) preds.full $ overall_noREs[[x]] $ cor $ estimate)), 
      as.numeric ( lapply ( 1  :  length (preds.nogen $ overall_noREs),  function (x) preds.nogen $ overall_noREs[[x]] $ cor $ estimate)), 
      as.numeric ( lapply ( 1  :  length (preds.noclim $ overall_noREs),  function (x) preds.noclim $ overall_noREs[[x]] $ cor $ estimate)) 
   ) 
    
    # add conditional with REs  
   mods_test_R $ Rval_cond_REs  <-   c ( 
      as.numeric ( lapply ( 1  :  length (preds.full $ cond_REs),  function (x) preds.full $ cond_REs[[x]] $ cor $ estimate)), 
      as.numeric ( lapply ( 1  :  length (preds.nogen $ cond_REs),  function (x) preds.nogen $ cond_REs[[x]] $ cor $ estimate)), 
      as.numeric ( lapply ( 1  :  length (preds.noclim $ cond_REs),  function (x) preds.noclim $ cond_REs[[x]] $ cor $ estimate)) 
   ) 
    
    # add conditional no REs  
   mods_test_R $ Rval_cond_noREs  <-   c ( 
      as.numeric ( lapply ( 1  :  length (preds.full $ cond_noREs),  function (x) preds.full $ cond_noREs[[x]] $ cor $ estimate)), 
      as.numeric ( lapply ( 1  :  length (preds.nogen $ cond_noREs),  function (x) preds.nogen $ cond_noREs[[x]] $ cor $ estimate)), 
      as.numeric ( lapply ( 1  :  length (preds.noclim $ cond_noREs),  function (x) preds.noclim $ cond_noREs[[x]] $ cor $ estimate)) 
   ) 
    
    # add garden and year information for plotting  
   mods_test_R $ garden  <-    sapply ( 1  :  nrow (mods_test_R),  FUN =   function (x)  strsplit (mods_test_R $ test_data,  '_' )[[x]][ 1 ]) 
   mods_test_R $ year  <-    sapply ( 1  :  nrow (mods_test_R),  FUN =   function (x)  strsplit (mods_test_R $ test_data,  '_' )[[x]][ 2 ]) 
    
      
    # calculate averages for each type of R calculated for the full model  
    colMeans (mods_test_R[mods_test_R $ model  ==   'full' , 3  :  6 ])    
  ##   Rval_overall_REs Rval_overall_noREs      Rval_cond_REs    Rval_cond_noREs 
##          0.3817800          0.3338020          0.4400723          0.3801952  
       # how many have accuracy >0.5?  
    sum (mods_test_R $ Rval_cond_noREs[mods_test_R $ model  ==   'full' ]  >  0.5 )    
  ## [1] 14  
       # ggplot settings  
    theme_set ( theme_bw ( base_size =   18 )) 
    
   gard_cols  <-   c ( "#008856" ,   "#8DB600" ,  "#BE0032" ,  "#875692" ,  "#A1CAF1" ,  "#2B3D26" ,  "#C2B280" ,  "#F3C300" ,  "#848482" ,  "#0067A5" ,  "#E68FAC" ,  "#F99379" ,  "#604E97" ,  "#B3446C" ,  "#DCD300" ,  "#882D17" ,  "#E25822" ,  "#654522" ) 
    
    # use ggpubr to add pvalues:  
    #https://www.r-bloggers.com/2017/06/add-p-values-and-significance-levels-to-ggplots/  
   my_comparisons  <-   combn ( unique (mods_test_R $ model),  2 ,  simplify =  F) 
    
   p1  <-   ggplot (mods_test_R,  aes ( x =  model,  y =  Rval_overall_REs)) +  
      geom_hline ( yintercept =   0 )  +  
      geom_violin ( draw_quantiles =   c ( 0.5 ))  +  
      scale_color_manual ( values =  gard_cols,  name =   'Garden' )  +  
      geom_jitter ( height =   0 ,  width =   0.1 ,  size =   3 ,  aes ( color =  garden,  shape =  year))  +  
      scale_shape_manual ( values =   c ( 16 , 1 ),  name =   'Year' )  +  
      ylim ( -  1 ,  1.2 )  +  
      ylab ( 'r (Testing dataset)' )  +  
      ggtitle ( 'Overall model, with random effects' )  +  
      stat_compare_means ( comparisons =  my_comparisons,  label =   'p.format' ,  method =   'wilcox.test' ,  paired =  T) 
    plot (p1)    
   
      p2  <-   
      ggplot (mods_test_R,  aes ( x =  model,  y =  Rval_overall_noREs)) +  
      geom_hline ( yintercept =   0 )  +  
      geom_violin ( draw_quantiles =   c ( 0.5 ))  +  
      geom_jitter ( height =   0 ,  width =   0.1 ,  size =   3 ,  aes ( color =  garden,  shape =  year))  +  
      scale_color_manual ( values =  gard_cols,  name =   'Garden' )  +  
      scale_shape_manual ( values =   c ( 16 , 1 ),  name =   'Year' )  +  
      ylim ( -  1 ,  1.2 )  +  
      ylab ( 'r (Testing dataset)' )  +  
      ggtitle ( 'Overall model, no random effects' )  +  
      stat_compare_means ( comparisons =  my_comparisons,  label =   'p.format' ,  method =   'wilcox.test' ,  paired =  T) 
      
    plot (p2)    
   
      p3  <-   ggplot (mods_test_R,  aes ( x =  model,  y =  Rval_cond_REs)) +  
      geom_hline ( yintercept =   0 )  +  
      geom_violin ( draw_quantiles =   c ( 0.5 ))  +  
      geom_jitter ( height =   0 ,  width =   0.1 ,  size =   3 ,  aes ( color =  garden,  shape =  year))  +  
      scale_color_manual ( values =  gard_cols,  name =   'Garden' )  +  
      scale_shape_manual ( values =   c ( 16 , 1 ),  name =   'Year' )  +  
      ylim ( -  1 ,  1.2 )  +  
      ylab ( 'r (Testing dataset)' )  +  
      ggtitle ( 'Conditional model, with random effects' )  +  
      stat_compare_means ( comparisons =  my_comparisons,  label =   'p.format' ,  method =   'wilcox.test' ,  paired =  T) 
      
    plot (p3)    
   
      p4  <-   ggplot (mods_test_R,  aes ( x =  model,  y =  Rval_cond_noREs)) +  
      geom_hline ( yintercept =   0 )  +  
      geom_violin ( draw_quantiles =   c ( 0.5 ))  +  
      geom_jitter ( height =   0 ,  width =   0.1 ,  size =   3 ,  aes ( color =  garden,  shape =  year))  +  
      scale_color_manual ( values =  gard_cols,  name =   'Garden' )  +  
      scale_shape_manual ( values =   c ( 16 , 1 ),  name =   'Year' )  +  
      ylim ( -  1 ,  1.2 )  +  
      ylab ( 'r (Testing dataset)' )  +  
      ggtitle ( 'Conditional model, no random effects' )  +  
      stat_compare_means ( comparisons =  my_comparisons,  label =   'p.format' ,  method =   'wilcox.test' ,  paired =  T) 
    plot (p4)    
   
       wrap_plots (p1, p2, p3, p4)    
   
       # ggsave(filename = paste('results/model_prediction/Rvalues_predictWithoutOneGarden_compareClimateGeneticsModels_', garden_clim_colname, '_vs_', pheno_colname, '.pdf', sep = ''), device = 'pdf', height = 14, width = 16)  
    # ggsave(filename = paste('results/model_prediction/Rvalues_predictWithoutOneGarden_compareClimateGeneticsModels_', garden_clim_colname, '_vs_', pheno_colname, '.png', sep = ''), device = 'png', height = 14, width = 16)  
    
    # just the cond_noREs plot  
    ggplot (mods_test_R,  aes ( x =  model,  y =  Rval_cond_noREs)) +  
      geom_hline ( yintercept =   0 )  +  
      geom_violin ( draw_quantiles =   c ( 0.5 ))  +  
      geom_jitter ( height =   0 ,  width =   0.1 ,  size =   3 ,  aes ( color =  garden,  shape =  year))  +  
      scale_color_manual ( values =  gard_cols,  name =   'Garden' )  +  
      scale_shape_manual ( values =   c ( 16 , 1 ),  name =   'Year' )  +  
      ylim ( -  1 ,  1.2 )  +  
      ylab ( 'r (Testing dataset)' )  +  
      ggtitle ( 'Conditional model, no random effects' )  +  
    stat_compare_means ( comparisons =  my_comparisons,  label =   'p.signif' ,  method =   'wilcox.test' ,  paired =  T)    
   
       # ggsave(filename = paste('results/model_prediction/Rvalues_condNoREs_predictWithoutOneGarden_compareClimateGeneticsModels_', garden_clim_colname, '_vs_', pheno_colname, '.png', sep = ''), device = 'png', height = 6, width = 8)  
    
    
    ##########  
    # just compare 2021  
    
   my_comparisons  <-   combn ( unique (mods_test_R $ model),  2 ,  simplify =  F) 
    
   p1  <-   ggplot ( subset (mods_test_R, year  ==   '2021' ),  aes ( x =  model,  y =  Rval_overall_REs)) +  
      geom_hline ( yintercept =   0 )  +  
      geom_violin ( draw_quantiles =   c ( 0.5 ))  +  
      geom_jitter ( height =   0 ,  width =   0.1 ,  size =   3 ,  aes ( color =  garden,  shape =  year))  +  
      scale_color_manual ( values =  gard_cols,  name =   'Garden' )  +  
      scale_shape_manual ( values =   c ( 16 , 1 ),  name =   'Year' )  +  
      ylim ( -  1 ,  1.2 )  +  
      ylab ( 'r (Testing dataset)' )  +  
      ggtitle ( 'Overall model, with random effects' )  +  
      stat_compare_means ( comparisons =  my_comparisons,  label =   'p.format' ,  method =   'wilcox.test' ,  paired =  T) 
    plot (p1)    
   
      p2  <-   
      ggplot ( subset (mods_test_R, year  ==   '2021' ),  aes ( x =  model,  y =  Rval_overall_noREs)) +  
      geom_hline ( yintercept =   0 )  +  
      geom_violin ( draw_quantiles =   c ( 0.5 ))  +  
      geom_jitter ( height =   0 ,  width =   0.1 ,  size =   3 ,  aes ( color =  garden,  shape =  year))  +  
      scale_color_manual ( values =  gard_cols,  name =   'Garden' )  +  
      scale_shape_manual ( values =   c ( 16 , 1 ),  name =   'Year' )  +  
      ylim ( -  1 ,  1.2 )  +  
      ylab ( 'r (Testing dataset)' )  +  
      ggtitle ( 'Overall model, no random effects' )  +  
      stat_compare_means ( comparisons =  my_comparisons,  label =   'p.format' ,  method =   'wilcox.test' ,  paired =  T) 
      
    plot (p2)    
   
      p3  <-   ggplot ( subset (mods_test_R, year  ==   '2021' ),  aes ( x =  model,  y =  Rval_cond_REs)) +  
      geom_hline ( yintercept =   0 )  +  
      geom_violin ( draw_quantiles =   c ( 0.5 ))  +  
      geom_jitter ( height =   0 ,  width =   0.1 ,  size =   3 ,  aes ( color =  garden,  shape =  year))  +  
      scale_color_manual ( values =  gard_cols,  name =   'Garden' )  +  
      scale_shape_manual ( values =   c ( 16 , 1 ),  name =   'Year' )  +  
      ylim ( -  1 ,  1.2 )  +  
      ylab ( 'r (Testing dataset)' )  +  
      ggtitle ( 'Conditional model, with random effects' )  +  
      stat_compare_means ( comparisons =  my_comparisons,  label =   'p.format' ,  method =   'wilcox.test' ,  paired =  T) 
      
    plot (p3)    
   
      p4  <-   ggplot ( subset (mods_test_R, year  ==   '2021' ),  aes ( x =  model,  y =  Rval_cond_noREs)) +  
      geom_hline ( yintercept =   0 )  +  
      geom_violin ( draw_quantiles =   c ( 0.5 ))  +  
      geom_jitter ( height =   0 ,  width =   0.1 ,  size =   3 ,  aes ( color =  garden,  shape =  year))  +  
      scale_color_manual ( values =  gard_cols,  name =   'Garden' )  +  
      scale_shape_manual ( values =   c ( 16 , 1 ),  name =   'Year' )  +  
      ylim ( -  1 ,  1.2 )  +  
      ylab ( 'r (Testing dataset)' )  +  
      ggtitle ( 'Conditional model, no random effects' )  +  
      stat_compare_means ( comparisons =  my_comparisons,  label =   'p.format' ,  method =   'wilcox.test' ,  paired =  T) 
    plot (p4)    
   
       wrap_plots (p1, p2, p3, p4)    
   
       #ggsave(filename = paste('results/model_prediction/Rvalues_predictWithoutOneGarden_compareClimateGeneticsModels2021_', garden_clim_colname, '_vs_', pheno_colname, '.png', sep = ''), device = 'png', height = 14, width = 16)  
    
    #####################  
    # just compare 2022  
    
   my_comparisons  <-   combn ( unique (mods_test_R $ model),  2 ,  simplify =  F) 
    
   p1  <-   ggplot ( subset (mods_test_R, year  ==   '2022' ),  aes ( x =  model,  y =  Rval_overall_REs)) +  
      geom_hline ( yintercept =   0 )  +  
      geom_violin ( draw_quantiles =   c ( 0.5 ))  +  
      geom_jitter ( height =   0 ,  width =   0.1 ,  size =   3 ,  aes ( color =  garden,  shape =  year))  +  
      scale_color_manual ( values =  gard_cols,  name =   'Garden' )  +  
      scale_shape_manual ( values =   c ( 16 , 1 ),  name =   'Year' )  +  
      ylim ( -  1 ,  1.1 )  +  
      ylab ( 'R (Testing dataset)' )  +  
      ggtitle ( 'Overall model, with random effects' )  +  
      stat_compare_means ( comparisons =  my_comparisons,  label =   'p.format' ,  method =   'wilcox.test' ,  paired =  T) 
    plot (p1)    
   
      p2  <-   
      ggplot ( subset (mods_test_R, year  ==   '2022' ),  aes ( x =  model,  y =  Rval_overall_noREs)) +  
      geom_hline ( yintercept =   0 )  +  
      geom_violin ( draw_quantiles =   c ( 0.5 ))  +  
      geom_jitter ( height =   0 ,  width =   0.1 ,  size =   3 ,  aes ( color =  garden,  shape =  year))  +  
      scale_color_manual ( values =  gard_cols,  name =   'Garden' )  +  
      scale_shape_manual ( values =   c ( 16 , 1 ),  name =   'Year' )  +  
      ylim ( -  1 ,  1.2 )  +  
      ylab ( 'R (Testing dataset)' )  +  
      ggtitle ( 'Overall model, no random effects' )  +  
      stat_compare_means ( comparisons =  my_comparisons,  label =   'p.format' ,  method =   'wilcox.test' ,  paired =  T) 
      
    plot (p2)    
   
      p3  <-   ggplot ( subset (mods_test_R, year  ==   '2022' ),  aes ( x =  model,  y =  Rval_cond_REs)) +  
      geom_hline ( yintercept =   0 )  +  
      geom_violin ( draw_quantiles =   c ( 0.5 ))  +  
      geom_jitter ( height =   0 ,  width =   0.1 ,  size =   3 ,  aes ( color =  garden,  shape =  year))  +  
      scale_color_manual ( values =  gard_cols,  name =   'Garden' )  +  
      scale_shape_manual ( values =   c ( 16 , 1 ),  name =   'Year' )  +  
      ylim ( -  1 ,  1.2 )  +  
      ylab ( 'R (Testing dataset)' )  +  
      ggtitle ( 'Conditional model, with random effects' )  +  
      stat_compare_means ( comparisons =  my_comparisons,  label =   'p.format' ,  method =   'wilcox.test' ,  paired =  T) 
      
    plot (p3)    
   
      p4  <-   ggplot ( subset (mods_test_R, year  ==   '2022' ),  aes ( x =  model,  y =  Rval_cond_noREs)) +  
      geom_hline ( yintercept =   0 )  +  
      geom_violin ( draw_quantiles =   c ( 0.5 ))  +  
      geom_jitter ( height =   0 ,  width =   0.1 ,  size =   3 ,  aes ( color =  garden,  shape =  year))  +  
      scale_color_manual ( values =  gard_cols,  name =   'Garden' )  +  
      scale_shape_manual ( values =   c ( 16 , 1 ),  name =   'Year' )  +  
      ylim ( -  1 ,  1.2 )  +  
      ylab ( 'R (Testing dataset)' )  +  
      ggtitle ( 'Conditional model, no random effects' )  +  
      stat_compare_means ( comparisons =  my_comparisons,  label =   'p.format' ,  method =   'wilcox.test' ,  paired =  T) 
    plot (p4)    
   
       wrap_plots (p1, p2, p3, p4)    
   
       #ggsave(filename = paste('results/model_prediction/Rvalues_predictWithoutOneGarden_compareClimateGeneticsModels2022_', garden_clim_colname, '_vs_', pheno_colname, '.png', sep = ''), device = 'png', height = 14, width = 16)  
    
    ########  
    
    # run linear model to test significance  
    anova ( lm (Rval_overall_REs  ~  model  *  year,  data =  mods_test_R))    
  ## Analysis of Variance Table
## 
## Response: Rval_overall_REs
##            Df Sum Sq  Mean Sq F value Pr(>F)
## model       2 0.0098 0.004923  0.1014 0.9037
## year        1 0.0689 0.068883  1.4188 0.2368
## model:year  2 0.0000 0.000006  0.0001 0.9999
## Residuals  87 4.2240 0.048551  
       anova ( lm (Rval_overall_noREs  ~  model * year,  data =  mods_test_R))    
  ## Analysis of Variance Table
## 
## Response: Rval_overall_noREs
##            Df Sum Sq  Mean Sq F value Pr(>F)
## model       2 0.0190 0.009519  0.1892 0.8279
## year        1 0.0551 0.055132  1.0960 0.2980
## model:year  2 0.0002 0.000097  0.0019 0.9981
## Residuals  87 4.3764 0.050303  
       pairwise.t.test (mods_test_R $ Rval_overall_noREs, mods_test_R $ model)    
  ## 
##  Pairwise comparisons using t tests with pooled SD 
## 
## data:  mods_test_R$Rval_overall_noREs and mods_test_R$model 
## 
##             full no_climate
## no_climate  1    -         
## no_genetics 1    1         
## 
## P value adjustment method: holm  
       anova ( lm (Rval_cond_REs  ~  model,  data =  mods_test_R))    
  ## Analysis of Variance Table
## 
## Response: Rval_cond_REs
##           Df Sum Sq  Mean Sq F value Pr(>F)
## model      2 0.0137 0.006836  0.1105 0.8955
## Residuals 90 5.5695 0.061883  
       anova ( lm (Rval_cond_noREs  ~  model,  data =  mods_test_R))    
  ## Analysis of Variance Table
## 
## Response: Rval_cond_noREs
##           Df Sum Sq  Mean Sq F value Pr(>F)
## model      2 0.0606 0.030309  0.4519 0.6379
## Residuals 90 6.0365 0.067072  
       pairwise.t.test (mods_test_R $ Rval_cond_noREs, mods_test_R $ model)    
  ## 
##  Pairwise comparisons using t tests with pooled SD 
## 
## data:  mods_test_R$Rval_cond_noREs and mods_test_R$model 
## 
##             full no_climate
## no_climate  1    -         
## no_genetics 1    1         
## 
## P value adjustment method: holm  
 
 
  8  Predicting phenotypes
based on model 
 
  8.1  Function for
predicting the response to climate 
       # predict each genotype's response to a vector of climate (MCMT) values  
    # garden_clims is a vector  
    # can calculate standard error of predictions using se.fit = TRUE - this takes longer to run  
    
   predict_genotype  <-   function (model, 
                                 type =   'response' ,  # can also be conditional or zprob  
                                effects, 
                                home_clim, 
                                garden_clims, 
                                 se.fit =   FALSE ){ 
      
      # vector to save predictions and their standard errors  
     pred_height  <-   vector () 
     pred_se  <-   vector () 
      
      
      # loop through garden climates  
      for (g  in   1  :  length (garden_clims)){ 
        
       home  <-  home_clim 
       garden  <-  garden_clims[g] 
        
        # make newdata to give to predict()  
       new_data  <-   as.data.frame ( cbind (home, garden, home ^  2 , garden ^  2 )) 
        colnames (new_data)  <-   c ( 'home_clim' ,  'garden_clim' ,  'home_clim_2' ,  'garden_clim_2' ) 
        # add other random or fixed effects  
        for (n  in   1  :  length (effects)){ 
         new_data[ names (effects[n])]  <-  effects[n] 
       } 
        
        # first have to scale new_data to match the scaled data used in model  
        for (v  in   1  :  length (new_data)){ 
         var  <-   names (new_data)[v] 
          
          # only scale if var exists in scaling_factor - random effects like genotype are not in scaling factor  
          # IF VARIABLES ARE NAMED DIFFERENTLY HERE IN effects AND scaling_factor IT WILL SKIP THEM  
          if (var  %in%   names (scaling_factor)){ 
           new_data[var]  <-  new_data[var] * scaling_factor[[var]] 
         }  else  { 
            warning (var,  ' does not have a scaling factor, skipping' ) 
         } 
          
          
       } 
        
        # predict response for this new data  
       pred  <-   predict (mod, 
                        type =  type, 
                        newdata =  new_data, 
                        re.form =   NA , 
                        allow.new.levels=  TRUE , 
                        se.fit =  se.fit) 
        
        if (se.fit  ==   TRUE ) { 
         fit  <-  pred $ fit 
         se  <-  pred $ se.fit 
       }  else  { 
         fit  <-  pred 
         se  <-   NA  
       } 
        
        # back transform from log if we're predicting height  
        # zprob is probability of a zero, so don't transform  
        if (type  %in%   c ( 'response' ,  'conditional' )){ 
         fit  <-   exp (fit)  -   1  
          # if we calculated standard error, transform that too  
          if (se.fit  ==   TRUE ){ 
           se  <-   exp (se)  -   1  
         } 
       } 
       pred_height[g]  <-  fit 
       pred_se[g]  <-  se 
     } 
      # save  
     pred_height_df  <-   data.frame ( garden_clim =  garden_clims,  predicted_height =  pred_height,  pred_se =  pred_se) 
      
      return (pred_height_df) 
      
      # for troubleshooting - also return input data  
      # return(list(predictions = pred_height_df,  
      #             newdata = new_data))  
   }    
 
 
  8.2  Setup 
       # get info for each genotype for use in predictions  
   geno_info  <-   as.data.frame ( matrix ( nrow=   length ( unique (dat $ Genotype)),  ncol =   4 )) 
    colnames (geno_info)  <-   c ( 'pc1' ,  'pc2' ,  'pc3' ,  'home_clim' ) 
    
    rownames (geno_info)  <-   unique (dat $ Genotype) 
    
    for (n  in   1  :  nrow (geno_info)){ 
      
     geno  <-   rownames (geno_info)[n] 
      
      # get first row with info for this genotype  
     geno_row  <-  dat[ match (geno, dat $ Genotype),] 
      
     geno_info $ pc1[n]  <-  geno_row $ genetic_PC1 
     geno_info $ pc2[n]  <-  geno_row $ genetic_PC2 
     geno_info $ pc3[n]  <-  geno_row $ genetic_PC3 
     geno_info $ home_clim[n]  <-  geno_row[,home_clim_colname] 
     geno_info $ Pt[n]  <-  geno_row $ Pt 
      
   } 
    
    # remove the ones with missing data  
   geno_info  <-  geno_info[ complete.cases (geno_info),] 
    
    # how many predictions to make for each genotype? (higher = smoother curves, but takes longer to run)  
   n_clims  <-   100  
    
    #############################################################   
    #overall prediction (incorporating both height and mortality)  
    
    
    # range of climate to predict for  
    # use range of both gardens and home climates  
   pred_clim_min  <-   range ( c (garden_clim, home_clim),  na.rm =  T)[ 1 ] 
   pred_clim_max  <-   range ( c (garden_clim, home_clim),  na.rm =  T)[ 2 ]    
 
 
  8.3  Predict response to
climate by genotype 
       ###############################################  
    
    # predictions for overall model (incorporating both height and mortality probability - makes predictions for height in cm)  
    
    # loop through genotypes and predict genotype-specific responses across the climate gradient (MCMT)  
   preds  <-   list () 
    
    par ( mfrow =   c ( 3 , 4 )) 
    
    for (n  in   1  :  nrow (geno_info)){ 
      
     geno  <-   rownames (geno_info)[n] 
      
     g.pc1  <-  geno_info $ pc1[n] 
     g.pc2  <-  geno_info $ pc2[n] 
     g.pc3  <-  geno_info $ pc3[n] 
     g.home_clim  <-  geno_info $ home_clim[n] 
      
      
      
     preds[[n]]  <-   predict_genotype (mod,  
                                     effects =   list ( pc1 =  g.pc1,  
                                                    pc2 =  g.pc2, 
                                                    pc3 =  g.pc3, 
                                                    genotype =  geno),  
                                     home_clim =  g.home_clim,  
                                     garden_clims =   seq (pred_clim_min, pred_clim_max,  length.out =  n_clims), 
                                     se.fit =   FALSE ) 
      
      # plot each genotype when done predicting  
      # plot(preds[[n]]$garden_clim, preds[[n]]$predicted_height, main = rownames(geno_info)[n])  
      # # lines for SE  
      # lines(preds[[n]]$garden_clim, preds[[n]]$predicted_height+preds[[n]]$pred_se, col = 'blue')  
      # lines(preds[[n]]$garden_clim, preds[[n]]$predicted_height-preds[[n]]$pred_se, col = 'blue')  
      #   
      cat ( paste ( 'done with' , n,  '  \n  ' )) 
      
   }    
  ## done with 1  
  ## done with 2  
  ## done with 3  
  ## done with 4  
  ## done with 5  
  ## done with 6  
  ## done with 7  
  ## done with 8  
  ## done with 9  
  ## done with 10  
  ## done with 11  
  ## done with 12  
  ## done with 13  
  ## done with 14  
  ## done with 15  
  ## done with 16  
  ## done with 17  
  ## done with 18  
  ## done with 19  
  ## done with 20  
  ## done with 21  
  ## done with 22  
  ## done with 23  
  ## done with 24  
  ## done with 25  
  ## done with 26  
  ## done with 27  
  ## done with 28  
  ## done with 29  
  ## done with 30  
  ## done with 31  
  ## done with 32  
  ## done with 33  
  ## done with 34  
  ## done with 35  
  ## done with 36  
  ## done with 37  
  ## done with 38  
  ## done with 39  
  ## done with 40  
  ## done with 41  
  ## done with 42  
  ## done with 43  
  ## done with 44  
       names (preds)  <-   paste ( 'genotype' ,  rownames (geno_info),  sep =   '_' ) 
    
      
    ######################  
    # plot all genotypes on one graph  
    
   colf  <-   colorRamp2 ( breaks =   c ( 1 ,  0.5 ,  0 ),  colors =   c ( '#BCEE68' ,  '#333333' ,  '#1C86EE' )) 
    
    #png(file = paste('results/model_prediction/predicted_height_by_genotype_overall_response_', clim_label, '.png', sep = ''), height = 8, width = 10, res = 300, units = 'in')  
    par ( mfrow =   c ( 1 , 1 ),  cex.lab =   1.5 ) 
    plot ( 0 , 0 , 
         xlim =   c (pred_clim_min, pred_clim_max),  
         ylim =   c ( 0 ,  ceiling ( max ( sapply ( 1  :  length (preds),  function (n)  max (preds[[n]] $ predicted_height))))),  
         type =   'n' ,  
         xlab =  clim_label,  
         ylab =   paste (pheno_label,  '(cm)' )) 
    
    for (n  in   1  :  length (preds)){ 
      
      lines (preds[[n]] $ garden_clim,  
           preds[[n]] $ predicted_height,  
            col =   colf (geno_info[n,  'Pt' ]),  
            lwd =   2 ) 
      
   } 
    
    
    # plot range of home climates  
    #abline(v = range(geno_info$home_clim, na.rm = T), lty = 2)  
    points ( x =  geno_info $ home_clim,  y =   rep ( 0 ,  nrow (geno_info))) 
    # plot range of garden climates  
    # abline(v = range(garden_clim, na.rm = T), lty = 1)  
    points ( x =   unique (garden_clim),  y =   rep ( 0 ,  length ( unique (garden_clim))),  pch =   '|' ) 
    legend ( 'topright' ,  pch =  c ( '⭘' ,  '|' ),  legend =   c ( 'Home Climate' ,  'Garden Climate' ))    
   
       #dev.off()  
    
    # plot with transfer distance instead of garden climate  
    
    # calculate x axis limits  
   xmin  <-  pred_clim_min  -   max (dat[,home_clim_colname]) 
   xmax  <-  pred_clim_max  -   min (dat[,home_clim_colname]) 
    
    #png(file = paste('results/model_prediction/predicted_height_by_genotype_transfer_dist_overall_response_', clim_label, '.png', sep = ''), height = 8, width = 10, res = 300, units = 'in')  
    par ( mfrow =   c ( 1 , 1 ),  cex.lab =   1.5 ) 
    plot ( 0 , 0 , 
         xlim =   c (xmin, xmax),  
         ylim =   c ( 0 ,  ceiling ( max ( sapply ( 1  :  length (preds),  function (n)  max (preds[[n]] $ predicted_height))))),  
         type =   'n' ,  
         xlab =   paste (clim_label,  'Transfer Distance' ),  
         ylab =   paste (pheno_label,  '(cm)' )) 
    
    abline ( v =   0 ,  lty =   1 ,  col =   'grey40' ) 
    
    for (n  in   1  :  length (preds)){ 
      
      lines (preds[[n]] $ garden_clim  -  geno_info $ home_clim[n],  
           preds[[n]] $ predicted_height,  
            col =   colf (geno_info[n,  'Pt' ]),  
            lwd =   2 ) 
      
   }    
   
       #dev.off()  
    
    
    # plot each individual separately, with real data points  
    
    # set shape for each year  
   shapes  <-   c ( 19 ,  17 ,  15 ) 
   cols.yr  <-   c ( 'black' ,  'blue' ,  'red' ) 
    
    #pdf(file = paste('results/model_prediction/model_and_data_by_genotype_overall_', garden_clim_colname, '_', pheno_colname, '.pdf', sep = ''), height = 10, width = 12)  
    par ( mfrow =   c ( 3 , 4 )) 
    for (n  in   1  :  length (preds)){ 
    
      # get genotype  
     geno  <-   gsub ( 'genotype_' ,  '' ,  names (preds)[n]) 
      
      # get genotype info  
     sub  <-  df[df $ genotype  ==  geno,] 
      
      # get R and p-value (calculated in model_plots chunk)  
     Rval  <-  r_vs_pt[geno,  'cor_R' ] 
     pval  <-  r_vs_pt[geno,  'cor_pval' ] 
      
      plot (sub $ garden_clim, sub $ pheno,  
           pch =  shapes[ as.factor (sub $ year)],  
           col =  cols.yr[ as.factor (sub $ year)], 
           #col = dat[dat$Genotype == geno, 'color_Pt'][1],  
           xlab =   paste ( 'Garden' , clim_label), 
           ylab =  pheno_label) 
      title ( main =   paste ( 'Genotype ' , geno,  '  \n  ' ,  'R = ' , Rval,  '  |  p = ' , pval,  sep =   '' ),  adj =   0 ) 
      
      lines (preds[[n]] $ garden_clim,  
           preds[[n]] $ predicted_height,  
            col =   colf (geno_info[n,  'Pt' ]),  
            lwd =   2 ) 
      
      # SE lines  
      #   lines(preds[[n]]$garden_clim, preds[[n]]$predicted_height+preds[[n]]$pred_se, col = 'grey')  
      # lines(preds[[n]]$garden_clim, preds[[n]]$predicted_height-preds[[n]]$pred_se, col = 'grey')  
      
      # line for home climate  
      abline ( v =  sub $ home_clim[ 1 ],  lty =   2 ,  col =   'grey' ) 
      
    
      
   }    
     
       # dev.off()  
    
    # does each genotype outcompete others at its home site?  
    
    #pdf(file = paste('results/model_prediction/model_compare_genotype_performance_at_home_', garden_clim_colname, '_', pheno_colname, '.pdf', sep = ''), height = 10, width = 12)  
    #png(file = paste('results/model_prediction/model_compare_genotype_performance_at_home_onePage', garden_clim_colname, '_', pheno_colname, '.png', sep = ''), height = 24, width = 16, units = 'in', res = 600)  
    
    #par(mfrow = c(9,5))  
    par ( mfrow =   c ( 3 , 4 ))    
   
       for (n  in   1  :  length (preds)){ 
      
      # get genotype  
     geno  <-   gsub ( 'genotype_' ,  '' ,  names (preds)[n]) 
      
      # get genotype info  
     sub  <-  df[df $ genotype  ==  geno,] 
      
      plot (sub $ garden_clim, sub $ pheno,  
           type =   'n' , 
           #pch = shapes[as.factor(sub$year)],   
           #col = cols.yr[as.factor(sub$year)],  
           #col = dat[dat$Genotype == geno, 'color_Pt'][1],  
           xlab =   paste ( 'Garden' , clim_label), 
           ylab =  pheno_label, 
           xlim =   c (pred_clim_min, pred_clim_max), 
           ylim =   c ( 0 ,  ceiling ( max ( sapply ( 1  :  length (preds),  function (n)  max (preds[[n]] $ predicted_height)))))) 
      title ( main =   paste ( 'Genotype' , geno),  adj =   0 ) 
      
      # line for home climate  
      abline ( v =  sub $ home_clim[ 1 ],  lty =   2 ,  lwd =   2 ,  col =   'black' ) 
      
      # add other genotypes  
      for (g  in   1  :  length (preds)){ 
        
        lines (preds[[g]] $ garden_clim,  
             preds[[g]] $ predicted_height,  
              col =   colf (geno_info[g,  'Pt' ]),  
              lwd =   1 , 
              lty =   2 ) 
     } 
      
      # line for this genotype  
      lines (preds[[n]] $ garden_clim,  
           preds[[n]] $ predicted_height,  
            col =   colf (geno_info[n,  'Pt' ]),  
            lwd =   3 ) 
      
      
   }    
     
       #dev.off()  
    
    ###############################################  
    
    # predictions for conditional model (just height)  
    
    # loop through and predict  
   preds.cond  <-   list () 
    
    par ( mfrow =   c ( 3 , 4 ))    
   
       for (n  in   1  :  nrow (geno_info)){ 
      
     geno  <-   rownames (geno_info)[n] 
      
     g.pc1  <-  geno_info $ pc1[n] 
     g.pc2  <-  geno_info $ pc2[n] 
     g.pc3  <-  geno_info $ pc3[n] 
     g.home_clim  <-  geno_info $ home_clim[n] 
      
      
     preds.cond[[n]]  <-   predict_genotype (mod,  
                                          type =   'conditional' ,  
                                          effects =   list ( pc1 =  g.pc1,  
                                                         pc2 =  g.pc2,  
                                                         pc3 =  g.pc3,  
                                                         genotype =  geno),  
                                          home_clim =  g.home_clim,  
                                          garden_clims =   seq (pred_clim_min, pred_clim_max,  length.out =  n_clims, 
                                                             se.fit =   FALSE )) 
      
      #plot(preds.cond[[n]]$garden_clim, preds.cond[[n]]$predicted_height, main = rownames(geno_info)[n])  
      
      #cat(paste('done with', n, '\n'))  
      
   } 
    
    names (preds.cond)  <-   paste ( 'genotype' ,  rownames (geno_info),  sep =   '_' ) 
    
    # plot conditional model  
    
    
    #png(file = paste('results/model_prediction/predicted_height_by_genotype_conditional_model_', clim_label, '.png', sep = ''), height = 8, width = 10, res = 300, units = 'in')  
    par ( mfrow =   c ( 1 , 1 ),  cex.lab =   1.5 ) 
    
    plot ( 0 , 0 ,  xlim =   c (pred_clim_min, pred_clim_max),  
         ylim =   c ( 0 ,  ceiling ( max ( sapply ( 1  :  length (preds.cond),  function (n)  max (preds.cond[[n]] $ predicted_height))))),  
         type =   'n' ,  
         xlab =  clim_label, 
         ylab =   paste (pheno_label,  '(cm)' )) 
    
    for (n  in   1  :  length (preds.cond)){ 
      
      lines (preds.cond[[n]] $ garden_clim,  
           preds.cond[[n]] $ predicted_height,  
            col =   colf (geno_info[n,  'Pt' ]),  
            lwd =   2 ) 
      
   } 
    
    # plot range of home climates  
    #abline(v = range(geno_info$home_clim, na.rm = T), lty = 2)  
    points ( x =  geno_info $ home_clim,  y =   rep ( 0 ,  nrow (geno_info))) 
    # plot range of garden climates  
    #abline(v = range(garden_clim, na.rm = T), lty = 1)  
    points ( x =   unique (garden_clim),  y =   rep (  0 ,  length ( unique (garden_clim))),  pch =   '|' ) 
    legend ( 'topright' ,  pch =  c ( '⭘' ,  '|' ),  legend =   c ( 'Home Climate' ,  'Garden Climate' ))    
   
       #dev.off()  
    
    # plot with transfer distance instead of garden climate  
    
    # calculate x axis limits  
   xmin  <-  pred_clim_min  -   max (dat[,home_clim_colname]) 
   xmax  <-  pred_clim_max  -   min (dat[,home_clim_colname]) 
    
    #png(file = paste('results/model_prediction/predicted_height_by_genotype_transfer_dist_conditional_model_', clim_label, '.png', sep = ''), height = 8, width = 10, res = 300, units = 'in')  
    par ( mfrow =   c ( 1 , 1 ),  cex.lab =   1.5 ) 
    plot ( 0 , 0 , 
         xlim =   c (xmin, xmax),  
         ylim =   c ( 0 , ceiling ( max ( sapply ( 1  :  length (preds.cond),  function (n)  max (preds.cond[[n]] $ predicted_height))))),  
         type =   'n' ,  
         xlab =   paste (clim_label,  'Transfer Distance' ),  
         ylab =   paste (pheno_label,  '(cm)' )) 
    
    abline ( v =   0 ,  col =   'grey40' ) 
    
    for (n  in   1  :  length (preds.cond)){ 
      
      lines (preds[[n]] $ garden_clim  -  geno_info $ home_clim[n],  
           preds.cond[[n]] $ predicted_height,  
            col =   colf (geno_info[n,  'Pt' ]),  
            lwd =   2 ) 
      
   }    
   
       #dev.off()  
    
    
    # each genotype individually  
    
    #pdf(file = paste('results/model_prediction/model_and_data_by_genotype_conditional_', garden_clim_colname, '_', pheno_colname, '.pdf', sep = ''), height = 10, width = 12)  
    par ( mfrow =   c ( 3 , 4 )) 
    for (n  in   1  :  length (preds)){ 
    
      # get genotype  
     geno  <-   gsub ( 'genotype_' ,  '' ,  names (preds)[n]) 
      
      # get genotype info  
     sub  <-  df[df $ genotype  ==  geno,] 
      
      # get R and p-value (calculated in model_plots chunk)  
      # Rval <- r_vs_pt[geno, 'cor_R']  
      # pval <- r_vs_pt[geno, 'cor_pval']  
      
      plot (sub $ garden_clim, sub $ pheno,  
           #pch = 16,   
           col =  cols.yr[ as.factor (sub $ year)], 
           pch =  shapes[ as.factor (sub $ year)], 
           #col = sub$color_Pt,  
           xlab =   paste ( 'Garden' , clim_label), 
           ylab =  pheno_label, 
           main =   paste ( 'Genotype' , geno)) 
      # haven't calculated R and p values for the conditional model yet, so don't plot them  
      #title(main = paste('Genotype ', geno, '\n', 'R = ', Rval, '  |  p = ', pval, sep = ''), adj = 0)  
      
      lines (preds[[n]] $ garden_clim, preds[[n]] $ predicted_height,  col =   colf (geno_info[n,  'Pt' ]),  lwd =   2 ,  lty =   2 ) 
      lines (preds.cond[[n]] $ garden_clim, preds.cond[[n]] $ predicted_height,  col =   colf (geno_info[n,  'Pt' ]),  lwd =   2 ) 
      
      # line for home climate  
      abline ( v =  sub $ home_clim[ 1 ],  lty =   2 ,  col =   'grey' ) 
      
   }    
     
       #dev.off()  
    
    
    #############################################  
    # predict for zero inflated model (mortality)  
    
    # loop through and predict  
   preds.zi  <-   list () 
    
    par ( mfrow =   c ( 3 , 4 ))    
   
       for (n  in   1  :  nrow (geno_info)){ 
      
     geno  <-   rownames (geno_info)[n] 
      
     g.pc1  <-  geno_info $ pc1[n] 
     g.pc2  <-  geno_info $ pc2[n] 
     g.pc3  <-  geno_info $ pc3[n] 
     g.home_clim  <-  geno_info $ home_clim[n] 
      
     preds.zi[[n]]  <-   predict_genotype (mod,  
                                        type =   'zprob' ,  
                                        effects =   list ( pc1 =  g.pc1,  
                                                       pc2 =  g.pc2,  
                                                       pc3 =  g.pc3,  
                                                       genotype =  geno),  
                                        home_clim =  g.home_clim,  
                                        garden_clims =   seq (pred_clim_min, pred_clim_max,  length.out =  n_clims), 
                                        se.fit =   FALSE ) 
      
      #plot(preds.zi[[n]]$garden_clim, preds.zi[[n]]$predicted_height, main = rownames(geno_info)[n], ylim = c(0,1))  
      
      #cat(paste('done with', n, '\n'))  
      
   } 
    
    names (preds.zi)  <-   paste ( 'genotype' ,  rownames (geno_info),  sep =   '_' ) 
    
    # plot zero-inflated model  
    
    
    #png(file = paste('results/model_prediction/predicted_height_by_genotype_zero-inflated_', clim_label, '.png', sep = ''), height = 8, width = 10, res = 300, units = 'in')  
    par ( mfrow =   c ( 1 , 1 ),  cex.lab =   1.5 ) 
    plot ( 0 , 0 , 
         xlim =   c (pred_clim_min, pred_clim_max),  
         ylim =   c ( 0 , 1 ),  
         type =   'n' ,  
         xlab =  clim_label,  
         ylab =   'Probablility of Mortality' ) 
    
    for (n  in   1  :  length (preds.zi)){ 
      
      lines (preds.zi[[n]] $ garden_clim,  
           preds.zi[[n]] $ predicted_height,  
            col =   colf (geno_info[n,  'Pt' ]),  
            lwd =   2 ) 
      
   } 
    
    # plot range of home climates  
    #abline(v = range(geno_info$home_clim, na.rm = T), lty = 2)  
    points ( x =  geno_info $ home_clim,  y =   rep ( 0 ,  nrow (geno_info))) 
    # plot range of garden climates  
    #abline(v = range(garden_clim, na.rm = T), lty = 1)  
    points ( x =   unique (garden_clim),  y =   rep (  0 ,  length ( unique (garden_clim))),  pch =   '|' ) 
    
    legend ( 'topright' ,  pch =  c ( '⭘' ,  '|' ),  legend =   c ( 'Home Climate' ,  'Garden Climate' ))    
   
       #dev.off()  
    
    # plot with transfer distance instead of garden climate  
    
    # calculate x axis limits  
   xmin  <-  pred_clim_min  -   max (dat[,home_clim_colname]) 
   xmax  <-  pred_clim_max  -   min (dat[,home_clim_colname]) 
    
    #png(file = paste('results/model_prediction/predicted_height_by_genotype_transfer_dist_zi_response_', clim_label, '.png', sep = ''), height = 8, width = 10, res = 300, units = 'in')  
    par ( mfrow =   c ( 1 , 1 ),  cex.lab =   1.5 ) 
    plot ( 0 , 0 , 
         xlim =   c (xmin, xmax),  
         ylim =   c ( 0 , 1 ),  
         type =   'n' ,  
         xlab =   paste (clim_label,  'Transfer Distance' ),  
         ylab =   'Probability of Mortality' ) 
    
    abline ( v =   0 ,  col =   'grey40' ) 
    
    for (n  in   1  :  length (preds.zi)){ 
      
      lines (preds.zi[[n]] $ garden_clim  -  geno_info $ home_clim[n],  
           preds.zi[[n]] $ predicted_height,  
            col =   colf (geno_info[n,  'Pt' ]),  
            lwd =   2 ) 
      
   }    
   
       #dev.off()  
    
    
    #################################  
    # calculate optimal climate  
   geno_info $ optimal_clim  <-   NA  
    for (n  in   1  :  length (preds)){ 
      
     tmp  <-  preds[[n]] 
     geno_info $ optimal_clim[n]  <-  tmp[ which.max (tmp $ predicted_height),  'garden_clim' ] 
      
   } 
    
    plot (geno_info $ home_clim, geno_info $ optimal_clim,  col =   colf (geno_info $ Pt),  pch =   16 ,  xlab =   'home climate' ,  ylab =   'predicted optimal climate' ,  cex =   1.5 )    
   
       plot (geno_info $ home_clim, geno_info $ optimal_clim,  type =   'n' ,  xlab =   'home climate' ,  ylab =   'predicted optimal climate' ,  cex =   1.5 ) 
    text (geno_info $ home_clim, geno_info $ optimal_clim,  labels =   rownames (geno_info),  col =   colf (geno_info $ Pt)) 
    abline ( 0 , 1 , lty =   2 )    
   
       # save  
    
    #predictions <- list(overall = preds, conditional = preds.cond, zi = preds.zi, genotypes = geno_info)  
    #save(predictions, file = paste('results/model_prediction/predictedGrowth_acrossClimates_byGenotype_', garden_clim_colname, '.Rdata', sep = ''))     
       # to skip running the predictions, which take a long time, can load output file from the above chunk  
    #load(paste('results/model_prediction/predictedGrowth_acrossClimates_byGenotype_', garden_clim_colname, '.Rdata', sep = ''))  
    # preds <- predictions$overall  
    # preds.cond <- predictions$conditional  
    # preds.zi <- predictions$zi  
    
   colf  <-   colorRamp2 ( breaks =   c ( 1 ,  0.5 ,  0 ),  colors =   c ( '#BCEE68' ,  '#333333' ,  '#1C86EE' )) 
    
    #png(file = paste('results/model_prediction/predicted_height_by_genotype_panel_', garden_clim_colname, sep = ''), height = 7, width = 10, res = 300, units = 'in')  
    
    par ( mfrow =   c ( 2 , 3 ),  cex.lab =   1.5 ,  cex.main =   1.5 ) 
    
    # overall  
    plot ( 0 , 0 , 
         xlim =   c (pred_clim_min, pred_clim_max),  
         ylim =   c ( 0 ,  ceiling ( max ( sapply ( 1  :  length (preds.cond),  function (n)  max (preds.cond[[n]] $ predicted_height))))),  
         type =   'n' ,  
         xlab =   paste (clim_label,  '(°C)' ),  
         ylab =   paste (pheno_label,  '(cm)' )) 
    title ( 'A. Overall model' ,  adj =   0 ) 
    
    for (n  in   1  :  length (preds)){ 
      
      lines (preds[[n]] $ garden_clim,  
           preds[[n]] $ predicted_height,  
            col =   colf (geno_info[n,  'Pt' ]),  
            lwd =   2 ) 
      
   } 
    
    
    # plot range of home climates  
    #abline(v = range(geno_info$home_clim, na.rm = T), lty = 2)  
    points ( x =  geno_info $ home_clim,  y =   rep ( 0 ,  nrow (geno_info)),  pch =   '|' ) 
    # plot range of garden climates  
    # abline(v = range(garden_clim, na.rm = T), lty = 1)  
    points ( x =   unique (garden_clim),  y =   rep ( 0 ,  length ( unique (garden_clim))),  pch =   1 ) 
    legend ( 'topright' ,  pch =  c ( '|' ,  '⭘' ),  legend =   c ( 'Home Climate' ,  'Garden Climate' )) 
    
      
    
    
    # conditional  
    plot ( 0 , 0 ,  xlim =   c (pred_clim_min, pred_clim_max),  
         ylim =   c ( 0 ,  ceiling ( max ( sapply ( 1  :  length (preds.cond),  function (n)  max (preds.cond[[n]] $ predicted_height))))), 
         type =   'n' ,  
         xlab =   paste (clim_label,  '(°C)' ), 
         ylab =   paste (pheno_label,  '(cm)' )) 
    title ( 'B. Conditional model (growth)' ,  adj =   0 ) 
    
    for (n  in   1  :  length (preds.cond)){ 
      
      lines (preds.cond[[n]] $ garden_clim,  
           preds.cond[[n]] $ predicted_height,  
            col =   colf (geno_info[n,  'Pt' ]),  
            lwd =   2 ) 
      
   } 
    
    # plot range of home climates  
    #abline(v = range(geno_info$home_clim, na.rm = T), lty = 2)  
    points ( x =  geno_info $ home_clim,  y =   rep ( 0 ,  nrow (geno_info)),  pch =   '|' ) 
    # plot range of garden climates  
    # abline(v = range(garden_clim, na.rm = T), lty = 1)  
    points ( x =   unique (garden_clim),  y =   rep ( 0 ,  length ( unique (garden_clim))),  pch =   1 ) 
    
    #legend('topright', pch =c(1, 16), legend = c('Home Climate', 'Garden Climate'))  
    
    
    # zi  
    plot ( 0 , 0 , 
         xlim =   c (pred_clim_min, pred_clim_max),  
         ylim =   c ( 0 , 1 ),  
         type =   'n' ,  
         xlab =   paste (clim_label,  '(°C)' ),  
         ylab =   'Probablility of Mortality' ) 
    title ( 'C. Zero-inflated Model (mortality)' ,  adj =   0 ) 
    
    for (n  in   1  :  length (preds.cond)){ 
      
      lines (preds.zi[[n]] $ garden_clim,  
           preds.zi[[n]] $ predicted_height,  
            col =   colf (geno_info[n,  'Pt' ]),  
            lwd =   2 ) 
      
   } 
    
    # plot range of home climates  
    #abline(v = range(geno_info$home_clim, na.rm = T), lty = 2)  
    points ( x =  geno_info $ home_clim,  y =   rep ( 0 ,  nrow (geno_info)),  pch =   '|' ) 
    # plot range of garden climates  
    # abline(v = range(garden_clim, na.rm = T), lty = 1)  
    points ( x =   unique (garden_clim),  y =   rep ( 0 ,  length ( unique (garden_clim))),  pch =   1 ) 
    
    #legend('topright', pch =c(1, 16), legend = c('Home Climate', 'Garden Climate'))  
    
    #################  
    
    # plot with transfer distance instead of garden climate  
    
    # overall  
    # calculate x axis limits  
   xmin  <-  pred_clim_min  -   max (dat[,home_clim_colname]) 
   xmax  <-  pred_clim_max  -   min (dat[,home_clim_colname]) 
    
    plot ( 0 , 0 , 
         xlim =   c (xmin, xmax),  
         ylim =   c ( 0 , ceiling ( max ( sapply ( 1  :  length (preds.cond),  function (n)  max (preds.cond[[n]] $ predicted_height))))),  
         type =   'n' ,  
         xlab =   paste (clim_label,  'Transfer Distance (°C)' ),  
         ylab =   paste (pheno_label,  '(cm)' )) 
    title ( 'D.' ,  adj =   0 ) 
    
    abline ( v =   0 ,  lty =   1 ,  col =   'grey40' ) 
    
    for (n  in   1  :  length (preds)){ 
      
      lines (preds[[n]] $ garden_clim  -  geno_info $ home_clim[n],  
           preds[[n]] $ predicted_height,  
            col =   colf (geno_info[n,  'Pt' ]),  
            lwd =   2 ) 
      
   } 
    
    # plot with transfer distance instead of garden climate  
    
    # calculate x axis limits  
   xmin  <-  pred_clim_min  -   max (dat[,home_clim_colname]) 
   xmax  <-  pred_clim_max  -   min (dat[,home_clim_colname]) 
    
    
    plot ( 0 , 0 , 
         xlim =   c (xmin, xmax),  
         ylim =   c ( 0 ,  ceiling ( max ( sapply ( 1  :  length (preds.cond),  function (n)  max (preds.cond[[n]] $ predicted_height))))),  
         type =   'n' ,  
         xlab =   paste (clim_label,  'Transfer Distance (°C)' ),  
         ylab =   paste (pheno_label,  '(cm)' )) 
    
    title ( 'E.' ,  adj =   0 ) 
    
    abline ( v =   0 ,  col =   'grey40' ) 
    
    for (n  in   1  :  length (preds.cond)){ 
      
      lines (preds[[n]] $ garden_clim  -  geno_info $ home_clim[n],  
           preds.cond[[n]] $ predicted_height,  
            col =   colf (geno_info[n,  'Pt' ]),  
            lwd =   2 ) 
      
   } 
    
    # plot with transfer distance instead of garden climate  
    
    # calculate x axis limits  
   xmin  <-  pred_clim_min  -   max (dat[,home_clim_colname]) 
   xmax  <-  pred_clim_max  -   min (dat[,home_clim_colname]) 
    
    
    plot ( 0 , 0 , 
         xlim =   c (xmin, xmax),  
         ylim =   c ( 0 , 1 ),  
         type =   'n' ,  
         xlab =   paste (clim_label,  'Transfer Distance (°C)' ),  
         ylab =   'Probability of Mortality' ) 
    
    title ( 'F.' ,  adj =   0 ) 
    
    abline ( v =   0 ,  col =   'grey40' ) 
    
    for (n  in   1  :  length (preds.zi)){ 
      
      lines (preds.zi[[n]] $ garden_clim  -  geno_info $ home_clim[n],  
           preds.zi[[n]] $ predicted_height,  
            col =   colf (geno_info[n,  'Pt' ]),  
            lwd =   2 ) 
      
   }    
   
       #dev.off()     
 
 


 

 

 

 

 


 
 

 
 
